# Transcriptional Activation of Prostate Specific Homeobox Gene NKX3-1 in Subsets of T-Cell Lymphoblastic Leukemia (T-ALL)

**DOI:** 10.1371/journal.pone.0040747

**Published:** 2012-07-27

**Authors:** Stefan Nagel, Stefan Ehrentraut, Jürgen Tomasch, Stefan Lienenklaus, Björn Schneider, Robert Geffers, Corinna Meyer, Maren Kaufmann, Hans G. Drexler, Roderick A. F. MacLeod

**Affiliations:** 1 Department of Human and Animal Cell Lines, Leibniz-Institute DSMZ - German Collection of Microorganisms and Cell Cultures, Braunschweig, Germany; 2 Microbial Communication, Helmholtz Centre for Infection Research, Braunschweig, Germany; 3 Molecular Immunology, Helmholtz Centre for Infection Research, Braunschweig, Germany; 4 Institute of Pathology, University of Rostock, Rostock, Germany; 5 Research Group for Genome Analytics, Helmholtz Centre for Infection Research, Braunschweig, Germany; Carl-Gustav Carus Technical University-Dresden, Germany

## Abstract

Homeobox genes encode transcription factors impacting key developmental processes including embryogenesis, organogenesis, and cell differentiation. Reflecting their tight transcriptional control, homeobox genes are often embedded in large non-coding, cis-regulatory regions, containing tissue specific elements. In T-cell acute lymphoblastic leukemia (T-ALL) homeobox genes are frequently deregulated by chromosomal aberrations, notably translocations adding T-cell specific activatory elements. NKX3-1 is a prostate specific homeobox gene activated in T-ALL patients expressing oncogenic TAL1 or displaying immature T-cell characteristics. After investigating regulation of NKX3-1 in primary cells and cell lines, we report its ectopic expression in T-ALL cells independent of chromosomal rearrangements. Using siRNAs and expression profiling, we exploited NKX3-1 positive T-ALL cell lines as tools to investigate aberrant activatory mechanisms. Our data confirmed NKX3-1 activation by TAL1/GATA3/LMO and identified LYL1 as an alternative activator in immature T-ALL cells devoid of GATA3. Moreover, we showed that NKX3-1 is directly activated by early T-cell homeodomain factor MSX2. These activators were regulated by MLL and/or by IL7-, BMP4- and IGF2-signalling. Finally, we demonstrated homeobox gene SIX6 as a direct leukemic target of NKX3-1 in T-ALL. In conclusion, we identified three major mechanisms of NKX3-1 regulation in T-ALL cell lines which are represented by activators TAL1, LYL1 and MSX2, corresponding to particular T-ALL subtypes described in patients. These results may contribute to the understanding of leukemic transcriptional networks underlying disturbed T-cell differentiation in T-ALL.

## Introduction

T-cells derive from early progenitor cells originating from hematopoietic stem cells in the bone marrow. After emigrating, T-cell progenitors complete their development in the thymus as thymocytes. Several pathways are crucial for developmental processes of thymocytes: these include BMP4, IGF2, IL2, IL4, IL7, IL15, NOTCH, TGFb and WNT signalling pathways [1]–[5]. The course of T-cell differentiation is mainly regulated via transcriptional processes [6]. Accordingly, many families of transcription factors (TF) are involved in T-cell gene regulation, including basic helix-loop-helix (bHLH) proteins, GATA-factors and LIM-domain factors. These three families assemble a TF complex which varies in composition in different hematopoietic lineages [7]–[11]. BHLH proteins TAL1 and LYL1 are restricted to progenitor cells undergoing silencing at subsequent thymocytic stages [12]. GATA2 also represents a progenitor factor which is respectively substituted by GATA1 and GATA3 in the erythroid and T-cell lineages [13].

Homeodomain proteins regulate fundamental differentiation processes in embryogenesis and the adult. Members of the HOX-family (HOXA5, HOXA9) and of the NKL-family (MSX2, HHEX) are active in the development of T-cells [14]–[16]. MSX2 is regulated by the BMP4-pathway in several developing tissues including T-cells, highlighting the transcriptional impact of this signalling pathway [15], [17].

In T-cell acute lymphoblastic leukemia (T-ALL) thymocyte differentiation is disturbed, resulting in leukemic cells developmentally arrested at particular stages. These cells express certain oncogenes which subsequently serve as indicators for classification of T-ALL subtypes [18]. Oncogenes comprise several families of TFs including bHLH (e.g. TAL1, LYL1) and NKL homeobox genes (e.g. TLX1, TLX3, NKX2-5). Chromosomal rearrangement is the most prominent mechanism of oncogene deregulation in T-ALL [19]. Aberrations deregulating NKL homeobox genes include translocations of the T-cell receptor (TCR) genes activating TLX1 via t(10;14)(q24;q11) or other T-cell specific genes like BCL11B activating TLX3 or NKX2-5 via t(5;14)(q35;q32) [20]–[22].

Many oncogenes identified in T-ALL encode factors regulating early stage specific thymocyte development (TAL1, LYL1, LMO2, HOXA5), or ectopically activated factors (TLX1, TLX3) [19]. Accordingly, activities of early stage specific oncogenes may induce stem cell-like characteristics in leukemic cells, and ectopically activated oncogenes regulate downstream genes which might correspond to heterologous developmental signatures, e.g. activation of the heart (and B-cell) specific gene MEF2C by the heart specific homeodomain protein NKX2-5 [23].

NKX3-1 is a member of the NKL-family of homeobox genes and is physiologically expressed in developing and mature prostate [24]. Transcription of this gene in prostate cells is regulated by several signalling pathways and tissue specific TFs [25]. Expression of NKX3-1 in T-ALL patients has been reported previously, associated with TAL1 expression, MLL translocations or an immature phenotype [14]. Moreover, Kusy and colleages demonstrated direct regulation of NKX3-1 by oncogenic TF complex TAL1/GATA3/LMO in T-ALL cells [26].

Here, we analyzed the deregulated expression of homeobox gene NKX3-1 in T-ALL cell lines. The aim of the study was to identify upstream and downstream activities of leukemic NKX3-1. Our data indicate absence of chromosomal aberrations and of ectopic prostate-specific impacts and illustrate particular pathways and factors activating leukemic NKX3-1 transcription.

## Materials and Methods

### Cell Lines and Treatments

Cell lines are held by the DSMZ (Braunschweig, Germany) except PER-117 generously provided by Ursula Kees, Perth, Australia [27]. Cultivation was performed as described previously [28]. Lentiviral mediated gene transfer of TAL1, LMO2, STAT5A and MSX2 into T-ALL cell lines has been described previously [15], [29]. Expression constructs for LYL1, GATA2, GATA3, MSX2, and NKX3-1 have been cloned in vector pCMV6 and were obtained from Origene (Wiesbaden, Germany). SiRNA oligonucleotides (siFOXA1, siETS1, siSOX4, siFGF9, siLMO1, siLMO2, siTAL1, siLYL1, siGATA3, siGATA2, siMLL, siIGF2BP1, siMSX2 and AllStars negative Control siRNA, named here siCTR) were obtained from Qiagen (Hilden, Germany). Both, siRNAs and expression constructs, were transfected into the cells by electroporation using the EPI-2500 impulse generator (Fischer, Heidelberg, Germany) at 350 V for 10 ms. Cell stimulations were performed by treatment with recombinant human BMP4, FGF9, IGF2, IL2, IL4, IL7, IL13, IL15, or WNT5B for 16 h at concentrations of 20 ng/ml (R & D Systems, Wiesbaden, Germany). Stimulations with all trans-retinoic acid (ATRA) or γ-secretase inhibitor N-[N-(3,5-Difluorophenacetyl)-L-alanyl]-S-phenylglycine t-butyl ester (DAPT) (Sigma, Taufkirchen, Germany) were performed at concentrations of 1 µM for 16 h. For stimulations with anti-CD3 (clone 2Q1170, Santa Cruz, Heidelberg, Germany) we used concentrations of 10 µg/ml for 16 h.

### Chromosomal Analyses

Fluorescent in-situ-hybridization (FISH) analyses were performed as described previously [30]. RP11-BAC-clones were obtained from the Childreńs Hospital Oakland Research Institute (CA, USA), prepared using the Big BAC DNA Kit (Princeton Separations, Adelphia, NJ, USA) and directly labelled by nick translation with dUTP-fluors (Dyomics, Jena, Germany). RP11-304K15, RP11-325C22, and RP11-268I13 were labelled with Dy-495, Dy-590 and Dy-547, respectively. Fluorescence images were captured using an Axio-Imager microscope (Zeiss, Göttingen, Germany) and analyzed with special software and a Spectral Imaging FISH system (Applied Spectral Imaging, Neckarhausen,Germany).

### Genomic Array Analyses

For genome-wide copy number analysis of cell lines JURKAT and PER-117 we used the Affymetrix platform. The datasets for JURKAT (500 K array) were obtained from the National Cancer Institute (Bethesda, MD, USA), GSK Cancer Cell Line Genomic Profiling Data (https://cabig.nci.nih.gov/community/caArray_GSKdata/). The datasets for PER-117 (100 K array) were generated at the Helmholtz Centre for Infection Research (Research Group for Genome Analytics, Braunschweig, Germany). Genotyping and unpaired copy number analysis were performed using 30 reference data sets as normal control downloaded from Affymetrix. Copy number analyses were performed using the Affymetrix Genotyping Console GTC Software version 4.0 (Affymetrix, High Wycombe, UK) and visualized by the Affymetrix GTC-Browser program.

### Polymerase Chain-reaction (PCR) Analyses

Total RNA from primary cells or cell lines was extracted using TRIzol reagent (Invitrogen, Karlsruhe, Germany). Peripheral blood mononuclear cells (PBMC) were isolated from healthy volunteers (coauthors of this paper) using Lymphoprep (Axis Shield PoC, Oslo, Norway). Primary CD19-positive B-cells were prepared from PBMC using corresponding magnetic beads from Miltenyi Biotec (Bergisch Gladbach, Germany). Subsequently, we determined the purity of CD19-positive cells at 85% by flow cytometry. Primary CD3-positive T-cells were provided by Prof. Dr. Michaela Scherr (Medical School Hannover, Germany) isolated from healthy donors, using magnetic beads from Miltenyi Biotec. Total human RNA isolated from bone marrow, lymph nodes, thymus, prostate, and retina was obtained from Clontech (Saint-Germain-en-Laye, France). Murine tissues, including bone marrow, popliteal lymph node, spleen, thymus, prostate, and eye were prepared from wild type C57BL/6 mice (Harlan-Winkelmann, Borchen, Germany). cDNA was subsequently synthesized from 5 µg RNA by random priming, using Superscript II (Invitrogen).

Real-time quantitative expression analysis (RQ-PCR) was performed by the 7500 Fast Real-time System, using commercial buffer and primer sets (Applied Biosystems, Darmstadt, Germany) or oligonucleotides obtained from MWG Eurofins (Martinsried, Germany) as listed in **Table 1**. For normalization of expression levels we used TATA box binding protein (TBP). Quantitative analyses were performed in triplicate and repeated twice. The statistical significance of gene expression differences was calculated using the T-test and indicated by p-values in the figures.

**Table 1:** Oligonucleotides used for PCR and cloning.

Gene	Acc.No	Comment	Forward (3′–5′)	Reverse (3′–5′)
LYL1	NM_005583	ChIP	GAGGCCCGCGCTGCTAGGTC	CCCGGTTTCCTCCCTCTCAC
MSX2	XM_037646	RQ-PCR	AGATGGAGCGGCGTGGATGC	CTCTGCACGCTCTGCAATGG
NKX3-1	AF247704	ChIP	GGCATTTCTAAACATGTCCAGCTGC	CTACCTCTCCCAGGACTGGG
		reporter	CTAAGCTTGTCCTCTGAGTTGCCCAGTC	TTGGATCCGGGAGGATATTAATGTTCTGTTTC
SIX6	NM_007374	reporter	CAAAGCTTACAAATACAGAGGGCATTTGG	ATGGATCCCTGTTCCTTGCACAGTGCCTG
TBP	NM_001172085	RQ-PCR	CGGAGAGTTCTGGGATTGT	CACGAAGTGCAATGGTCTTT

Restriction sites for *Hind*III and *Bam*HI are underlined.

### Protein Analysis

Western blot analysis was performed by the semi-dry method. Proteins obtained from cell lysates were transferred onto nitrocellulose membranes (Bio-Rad, München, Germany) which were blocked with 5% dry milk powder dissolved in phosphate-buffered-saline buffer (PBS). The following antibodies were obtained from Invitrogen: anti-NKX3.1 (clone 3–9), from Santa Cruz Biotechnology: anti-ERK1 (K-23), GATA2 (H-116), GATA3 (HG3-31), and from Abnova (Aachen, Germany): anti-LYL1 (B01P). The secondary antibodies have been connected with peroxidase and were detected by Western Lightning-ECL (Perkin Elmer, Waltham, MA, USA).

### Chromatin Immuno-precipitation (ChIP)

ChIP analysis was performed with the ChIP Assay Kit (Millipore-Upstate, Schwalbach, Germany) as reported previously [31], isolating genomic DNA fragments generated by sonication, using antibody anti-GATA2 (H-116, Santa Cruz Biotechnology). The subsequent ChIP-PCR analysis was performed using taqpol (Qiagen), oligonucleotides as listed in **Table 1** (MWG Eurofins) and the thermocycler TGradient (Biometra, Göttingen, Germany).

### Reporter Gene Analysis

For creation of the reporter gene constructs we combined a reporter gene with genomic fragments, containing binding sites for MSX2 or NKX3-1. We cloned genomic PCR products (oligonucleotides are listed in **Table 1**) of the corresponding upstream regions (regulator) and of the HOXA9 gene, comprising exon1-intron1-exon2 (reporter), into the *Hind*III/*Bam*HI and *Eco*RI sites, respectively, of the expression vector pcDNA3 downstream of the CMV enhancer [31]. The constructs were validated by sequence analysis (MWG Eurofins). Taqman real-time PCR using a commercial HOXA9 assay quantified the spliced reporter-transcript, corresponding to promoter activity. A cotransfected luciferase construct served as transfection control, quantified by the Luciferase Assay System (Promega) using the luminometer Lumat LB9501 (Berthold Technologies, Bad Wildbad, Germany).

### Expression Profiling

For quantification of gene expression via profiling, we used gene chips HG U133 Plus 2.0 from Affymetrix (High Wycombe, UK). The datasets were generated at the University of Würzburg and generously provided by Prof. Andreas Rosenwald (Institute of Pathology, University of Würzburg, Germany) or obtained from the National Center for Biotechnology Information (NCBI) Gene Expression Omnibus (www.ncbi.nlm.nih.gov/gds). Analyses of expression data were performed using Microsoft Excel and online programs. For creation of heat maps we used CLUSTER version 2.11 and TREEVIEW version 1.60 (http://rana.lbl.gov/EisenSoftware.htm). For statistical analyses the primary expression data (CEL-files) were processed using the R-based Bioconductor package before comparing NKX3-1 positive and negative T-ALL cell lines using the LIMMA package [32]. Only those genes showing a p-value <0.003 were assumed to be differentially expressed in both groups of cell lines. Subsequent generation of heat maps was performed using the basic functions.

## Results

### Expression of NKX3-1 in T-ALL Cells

**Figure 1:**
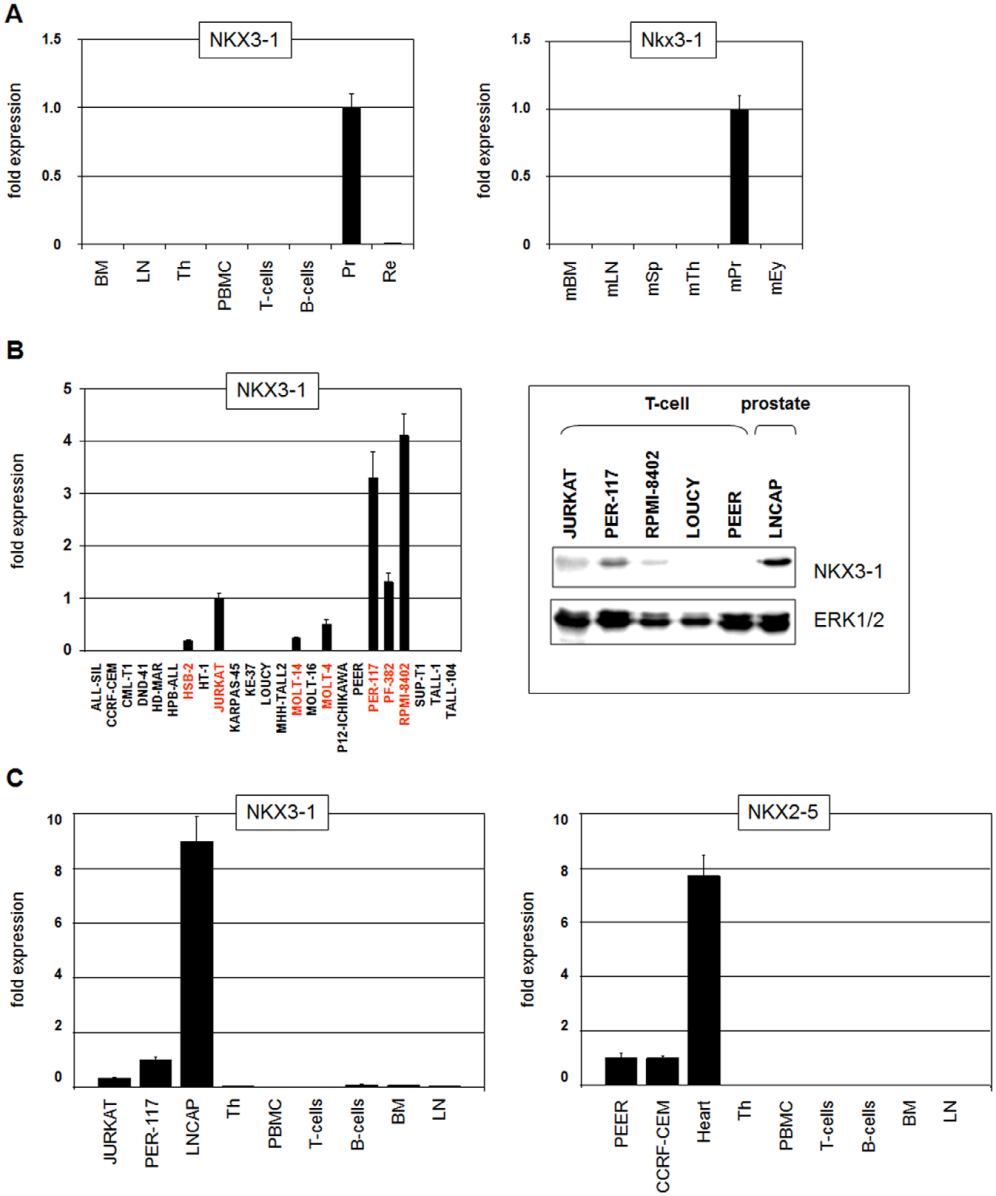
Expression of NKX3-1. (**A**) Expression analyses of human (left) and murine (right) NKX3-1 transcripts in primary tissues including bone marrow (BM), lymph node (LN), thymus (Th), peripheral blood mononuclear cells (PBMC), T-cells, B-cells, prostate (Pr), retina (Re), spleen (Sp) and eye (Ey) were performed by RQ-PCR. Note the data demonstrate exclusive NKX3-1 expression in prostate cells. (**B**) RQ-PCR analysis of NKX3-1 transcripts in 24 T-ALL cell lines revealed 7 positive cell lines indicated in red letters (left). Western blot analysis shows NKX3-1 protein expression in JURKAT, PER-117 and RPMI-8402 but not in LOUCY and PEER (right). Prostate cell line LNCAP served as positive control for NKX3-1 and ERK1/2 as loading control. (**C**) RQ-PCR analysis of NKX3-1 (left) and NKX2-5 (right) was performed in T-ALL cell lines in addition to primary tissues. The respective expression deficits of NKX3-1 and NKX2-5 in T-ALL versus tissue controls LNCAP (prostate) and heart were similar.

To examine the physiological expression of NKX3-1 we measured its RNA level in primary human cells of the prostate, retina and several hematopoietic tissues, including bone marrow, lymph node, thymus, PBMC, T- and B-cells, in addition to diverse primary murine cell types (**Fig. 1A**). Data from both species indicated exclusive expression of NKX3-1 in prostate cells and its absence in the hematopoietic compartment. For analysis of NKX3-1 expression in T-ALL we screened 24 human T-ALL cell lines by RQ-PCR (**Fig. 1B**). Seven T-ALL cell lines (HSB-2, JURKAT, MOLT-14, MOLT-4, PER-117, PF-382, RPMI-8402) demonstrated detectable NKX3-1 expression with different intensities. Western blot analysis showed significant NKX3-1 protein levels in JURKAT, PER-117 and RPMI-8402 as compared to the prostate cell line LNCAP (**Fig. 1B**). Of note, the t(X;11)(q13;q23) positive cell line KARPAS-45 expressing MLL-AFX (MLL-FOXO4) fusion protein showed no NKX3-1 expression, discounting direct activation by MLL fusion proteins.

Thereafter, NKX3-1 transcript levels of JURKAT and PER-117 were compared to that of the prostate cell line LNCAP, indicating about 9-fold higher expression in prostate cells than in T-ALL cells (**Fig. 1C**). Interestingly, heart cells expressed about 8-fold higher levels of homeobox gene NKX2-5 than t(5;14)(q35;q32) positive T-ALL cell lines CCRF-CEM and PEER (**Fig. 1C**). These data demonstrate aberrant and ectopic expression of both NKL homeobox genes in T-ALL cells at similar levels when compared to their physiological tissue controls.

### Absence of Chromosomal Aberrations at NKX3-1 in T-ALL

**Figure 2:**
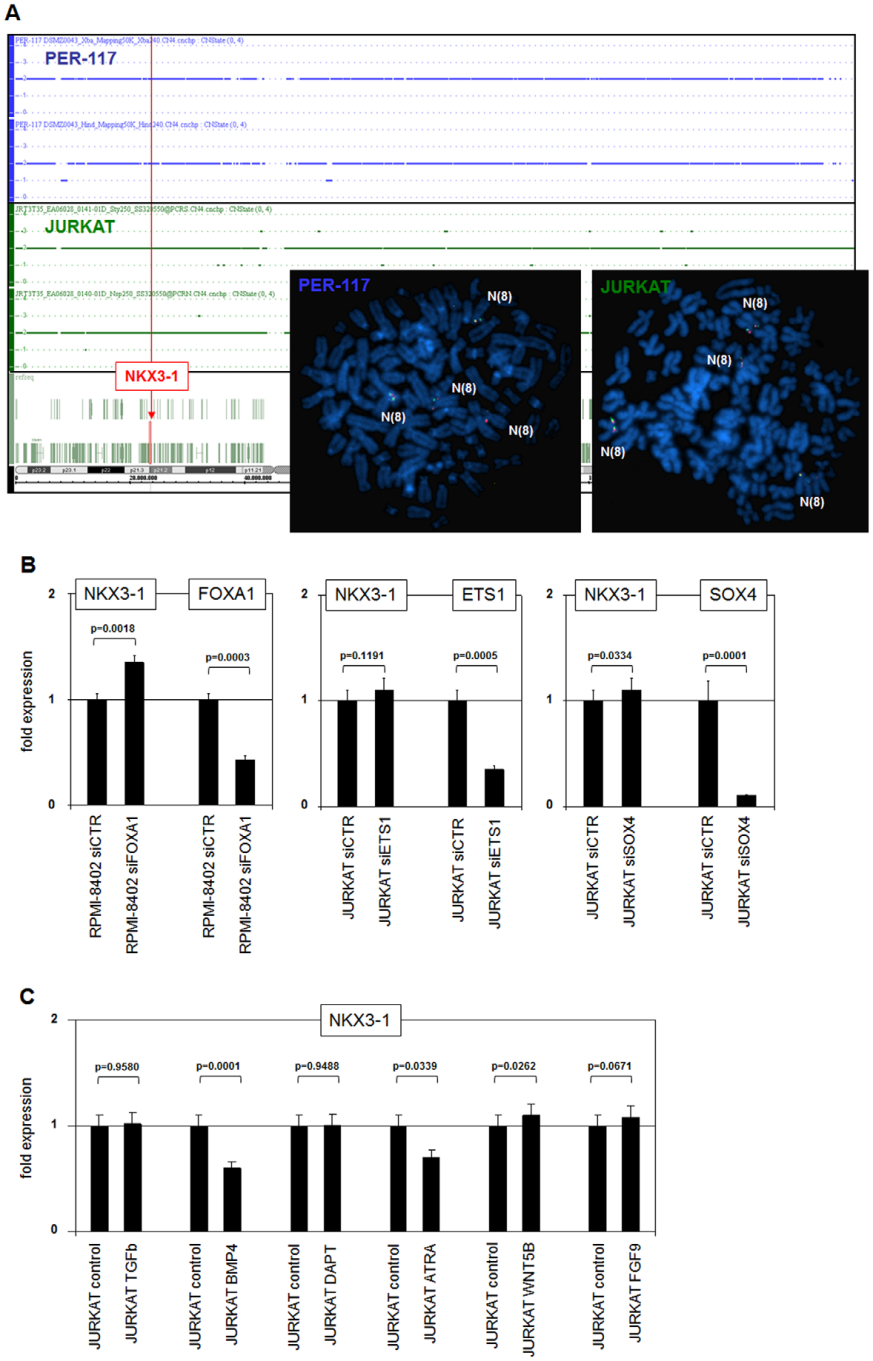
Analyses of NKX3-1 deregulation in T-ALL. (**A**) FISH analysis of NKX3-1 positive cell lines PER-117 and JURKAT was performed by two NKX3-1 flanking and one straddling probe (inserts). Genomic array analysis of T-ALL cell lines PER-117 and JURKAT shows no change of copy numbers along the chromosome 8 including locus NKX3-1 at 8p21 (marked by a red line). Together, these data demonstrate absence of rearrangements or copy number changes at NKX3-1. (**B**) RQ-PCR analyses of T-ALL cell lines treated with siRNAs directed against FOXA1, ETS1 and SOX4 demonstrate reduced expression of the targeted genes while sparing NKX3-1. (**C**) RQ-PCR analysis of NKX3-1 in JURKAT cells treated with several ligands for particular signalling pathways. Data show reduced NKX3-1 expression in cells treated with BMP4 or ATRA.

To investigate whether the ectopic expression of NKX3-1 in T-ALL cells was chromosomal in origin, we performed FISH analyses on metaphase chromosomes of all seven NKX3-1 positive T-ALL cell lines using flanking and straddling probes (**Fig. 2A** **, Fig. S1**). However, no chromosomal rearrangements were detected, indicating a wild type configuration throughout. We then analyzed copy number variations in JURKAT and PER-117 by genomic profiling. Again in both cell lines no changes in genomic copy number at the NKX3-1 locus at 8p21 were detected (**Fig. 2A**). We concluded that ectopic NKX3-1 expression in T-ALL cells is not chromosomally mediated contrasting with NKX2-5 and other leukemic NKL homeobox genes in T-ALL. Therefore, we postulated that deregulated expression of NKX3-1 in T-ALL might be due to aberrant activities of signalling pathways and/or TFs.

### Analyses of TFs and Signalling Pathways of Prostate and T-ALL

NKX3-1 is physiologically expressed and regulated in prostate cells [24]. To examine potential aberrant activities of prostate specific activatory TFs, we analyzed the roles of FOXA1, ETS1 and SOX4 in T-ALL cell lines [25]. Array data indicated significant expression levels of ETS1 and SOX4 in T-ALL cell lines while that of FOXA1 appeared inconspicuous (**Fig. S2A**). Nevertheless, the NKX3-1 positive cell line RPMI-8402 expressed the highest levels of FOXA1 as analyzed by RQ-PCR (**Fig. S2A**). SiRNA mediated knockdown of FOXA1, ETS1 and SOX4 in RPMI-8402 and JURKAT cells inhibited the targeted TFs, while NKX3-1 was spared (**Fig. 2B**). We concluded that these prostate specific TFs do not activate NKX3-1 transcription in T-ALL cells.

To examine aberrant activities of signalling pathways present in prostate cells we analyzed the potential effects of BMP-, FGF-, NOTCH-, TGFbeta-, WNT-pathways and steroid ligands [25]. Array data supported their possible involvement from expression of corresponding receptors and ligands in T-ALL cell lines (**Fig. S2B**). Accordingly, JURKAT cells were treated with BMP4, TGFbeta, FGF9, NOTCH-inhibitor DAPT, WNT5B, and ATRA. However, none of these succeeded in inducing any increase in NKX3-1 expression as analyzed by RQ-PCR. Indeed, BMP4 and ATRA reduced expression of NKX3-1 (**Fig. 2C**). Of note, excepting FGF9 these factors and pathways also play a role in T-cell development. Therefore, BMP4 and retinoic acid signalling may physiologically contribute to NKX3-1 silencing in developing T-cells.

### TAL1 and LYL1 Activate Expression of NKX3-1 in T-ALL in different Modes

Recently, Kusy and colleagues found in JURKAT cells that oncogene TAL1 activates NKX3-1 transcription in concert with GATA3 and LMO proteins [26]. Here, we analyzed if this TF constellation is generally responsible for NKX3-1 activation in T-ALL cells. Therefore, we screened the expression levels in T-ALL cell lines of major TFs constituting this transcription complex, comprising LMO1/2/4, TAL1/LYL1 and GATA2/3.

**Figure 3:**
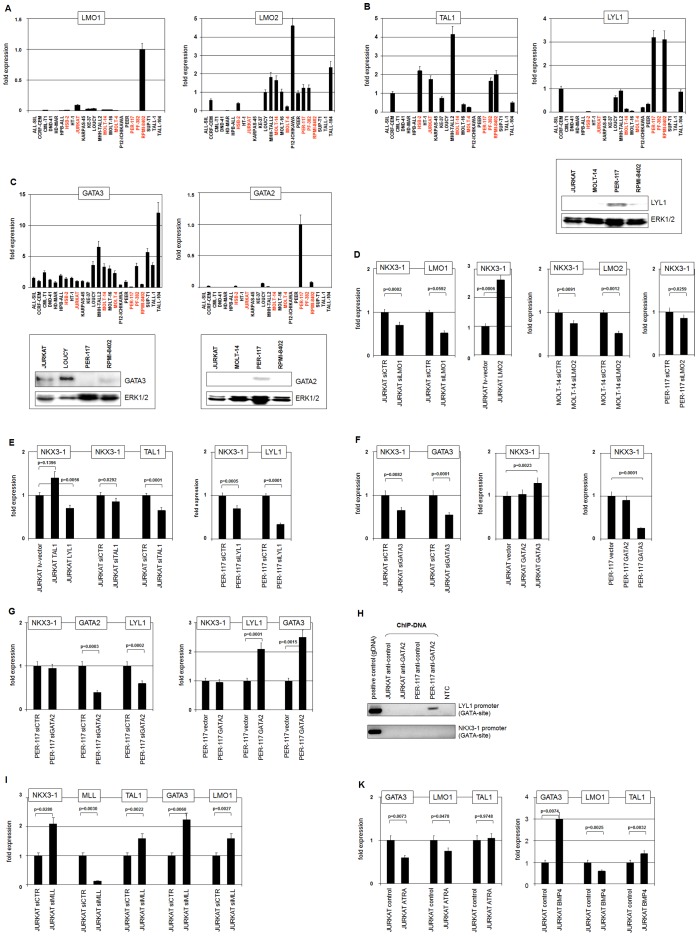
Analyses of LMO-, bHLH-, and GATA-factors in NKX3-1 deregulation. Quantification of gene expression by RQ-PCR in 24 T-ALL cell lines for (**A**) LMO1 (left) and LMO2 (right), (**B**) TAL1 (left) and LYL1 (right), (**C**) GATA3 (left) and GATA2 (right). NKX3-1 expressing cell lines are indicated in red letters. Additionally, Western blot analyses were performed for LYL1 (**B**), GATA3 and GATA2 (**C**). ERK1/2 served as loading control. The results of these expression analyses are given in Table 2. (**D**) RQ-PCR analyses of T-ALL cell lines treated with siRNAs directed against LMO1 or LMO2 demonstrate reduced expression of the targeted genes. The expression levels of NKX3-1 were decreased significantly in JURKAT and MOLT-14 but not in PER-117. Lentiviral mediated overexpression of LMO2 in JURKAT resulted in increased NKX3-1 expression. (**E**) JURKAT cells were treated for overexpression of TAL1 or LYL1 by lentiviral gene transfer and for knockdown of TAL1 by siRNA. NKX3-1 expression data indicate activation by TAL1 and inhibition by LYL1. In contrast, siRNA mediated knockdown of LYL1 in PER-117 indicate activation of NKX3-1 expression. Knockdowns of both TAL1 and LYL1 in JURKAT and PER-117, respectively, were confirmed by RQ-PCR. (**F**) JURKAT cells were treated for knockdown of GATA3 by siRNA, indicating activation of NKX3-1 expression by GATA3. Overexpression of GATA3 or GATA2 by electroporation of expression constructs showed no significant effect for GATA2 in JURKAT or in PER-117. However, GATA3 enhanced NKX3-1 expression in JURKAT while it was inhibited in PER-117, indicating differences in regulation. (**G**) PER-117 cells were treated for knockdown (left) and for overexpression of GATA2 (right). Collectively, the data demonstrate activation by GATA2 in LYL1 as well as in GATA3. (**H**) ChIP analysis was performed using anti-GATA2 for precipitation of genomic DNA fragments. Subsequent PCR analysis indicates direct binding of GATA2 at the LYL1 but not NKX3-1 promoter. NTC: no template control. (**I**) JURKAT cells were treated for siRNA mediated knockdown of MLL. RQ-PCR analyses demonstrate concomitant repression of MLL and enhancement of NKX3-1, TAL1, GATA3 and LMO1 transcription. (**K**) Stimulation of JURKAT cells with ATRA (left) or BMP4 (right) modulated expression levels of GATA3, LMO1 and TAL1. While ATRA stimulation inhibited expression of both GATA3 and LMO1, BMP4 stimulation enhanced GATA3 and TAL1 but inhibited LMO1.

LMO1 was prominently expressed in NKX3-1 positive cell lines JURKAT and RPMI-8402 which carries a chromosomal aberration, t(11;14)(p15;q11), activating LMO1 (**Fig. 3A**). LMO2 expression was detected in 12 T-ALL cell lines confirming a previous report [33], 5 of which also express NKX3-1 (**Fig. 3A**). Expression of LMO4 was ubiquitous, detected in 23/24 T-ALL cell lines, discounting any specific impact on NKX3-1 activation (**Fig. S3**). Expression of TAL1 was detected in 11 cell lines, 6 of which also expressed NKX3-1 (**Fig. 3B**). In 24 cell lines LYL1 transcripts were detected in 11 examples, four of which were NKX3-1 positive. The expression of LYL1 protein in PER-117 and RPMI-8402 was confirmed by Western blot analysis (**Fig. 3B**). However, the expression levels in RPMI-8402 do not correlate between RNA and protein, most likely indicating posttranscriptional regulation. GATA3 transcripts were detected in all 24 T-ALL cell lines. However, expression in PER-117 was barely detectable. Accordingly, GATA3 protein was not detectable in this cell line, while JURKAT, LOUCY and RPMI-8402 all tested positive (**Fig. 3C**). GATA2 was expressed prominently in PER-117 and moderately so in 5 additional cell lines. However, GATA2 protein was only detectable in PER-117, corresponding to its immature phenotype (**Fig. 3C**) [27]. **Table 2** summarizes these expression data for NKX3-1 positive cell lines, indicating two different TF combinations: (i) TAL1, GATA3, LMO1/2 and (ii) LYL1, GATA2, LMO2. Therefore, we focused on JURKAT and PER-117 as models of these two type classes, which posited to represent TAL1-positve and immature T-ALL, respectively, and might operate differently regarding NKX3-1 expression.

**Table 2:** Expression results for GATA/TAL1/LYL1/LMO transcription factors.

cell line	GATA2	GATA3	TAL1	LYL1	LMO1	LMO2	LMO4
HSB-2	−	+++	+++	−	−	+++	+++
**JURKAT**	−	+++	+++	−	+++	−	+++
MOLT-4	−	+++	+++	−	−	+++	+++
MOLT-14	−	+++	+++	+	−	+++	+++
**PER-117**	+++	+	−	+++	−	+++	+++
PF-382	−	+++	+++	−	−	+++	+++
RPMI-8402*	+	+	+++	+	+++	−	+++

+++ significant expression;

+ expression detectable;

- no expression.

* RPMI-8402 expressed LYL1 at high RNA level but only low at the protein level.

Using siRNA mediated knockdown of particular TFs and subsequent quantification of NKX3-1 expression by RQ-PCR we were able to measure their likely impact on transcriptional activity. Knockdown of LMO1 in JURKAT cells and of LMO2 in MOLT-14 cells resulted in reduced expression of LMO1/2 and NKX3-1, confirming the activatory impact of LMO proteins in TAL1 positive T-ALL cells (**Fig. 3D**). However, LMO2 knockdown in PER-117 showed only limited reduction of NKX3-1 expression, indicating differences between the immature and the TAL1-type in NKX3-1 activation (**Fig. 3D**). Overexpression and knockdown of TAL1 in JURKAT consistently demonstrated its activating impact on NKX3-1 expression as described previously (**Fig. 3E**) [26]. Interestingly, overexpressing LYL1 resulted in reduced expression of NKX3-1 in JURKAT, as did siRNA mediated knockdown of LYL1 in PER-117 (**Fig. 3E**). These results demonstrate contrasting activatory and inhibitory roles of LYL1 in PER-117 and JURKAT, respectively, betraying further differences in NKX3-1 regulation in these T-ALL subtypes.

Next we analyzed the role of GATA-factors in NKX3-1 regulation. SiRNA mediated knockdown and overexpression of GATA3 in JURKAT demonstrated an activatory role (**Fig. 3F**). Overexpression of GATA2 left NKX3-1 expression unperturbed, as well in JURKAT as in PER-117 (**Fig. 3F**). In contrast, overexpression of GATA3 in PER-117 was accompanied by conspicuously reduced NKX3-1 expression (**Fig. 3F**), contrasting the situation in JURKAT. SiRNA mediated knockdown of GATA2 in PER-117 reduced LYL1, while overexpression activated LYL1 (**Fig. 3G**), confirming the known regulatory role of GATA2 on this gene [34]. However, expression of NKX3-1 remained unmoved despite the activatory input of LYL1 on NKX3-1 expression (**Fig. 3E**). Interestingly, in PER-117 GATA2 overexpression was accompanied by increased expression of GATA3 (**Fig. 3G**), which in turn reduced NKX3-1 expression (**Fig. 3F**). Thus, overexpression of GATA2 showed opposing activities in NKX3-1 expression, stimulating both activatory LYL1 and inhibitory GATA3. ChIP-analysis of untreated PER-117 cells demonstrated binding of GATA2 to the LYL1 promoter but not to the reported regulatory GATA-site of NKX3-1 (**Fig. 3H**), highlighting its contribution to LYL1 expression at limited expression levels. Together, these data demonstrate that TAL1 together with GATA3 and LMO proteins activates NKX3-1 transcription as shown previously [26]. Alternatively, LYL1 activates NKX3-1 in the absence of GATA3. Moreover, the combination of LYL1 and GATA3 appears to inhibit transcription of NKX3-1.

Accordingly, siRNA mediated knockdown of MLL in JURKAT cells boosted expression of TAL1, GATA3, LMO1 and subsequently that of NKX3-1 (**Fig. 3I**). Collectively, these data chart the inhibitory impact of MLL on prominent leukemic activators of NKX3-1. MLL translocations are accompanied by reduced levels of wild type MLL which may thus contribute indirectly to the expression of NKX3-1. Furthermore, treatment of JURKAT with ATRA reduced expression of NKX3-1 activators GATA3 and LMO1 as well, proffering an explanation for the suppressive impact of this steroid ligand (**Fig. 3K**). In contrast, treatment with BMP4 yielded conflicting results: activation of GATA3 and TAL1 but inhibition of LMO1 (**Fig. 3K**).

### Identification of Novel Upstream Regulators of NKX3-1 in T-ALL

**Figure 4:**
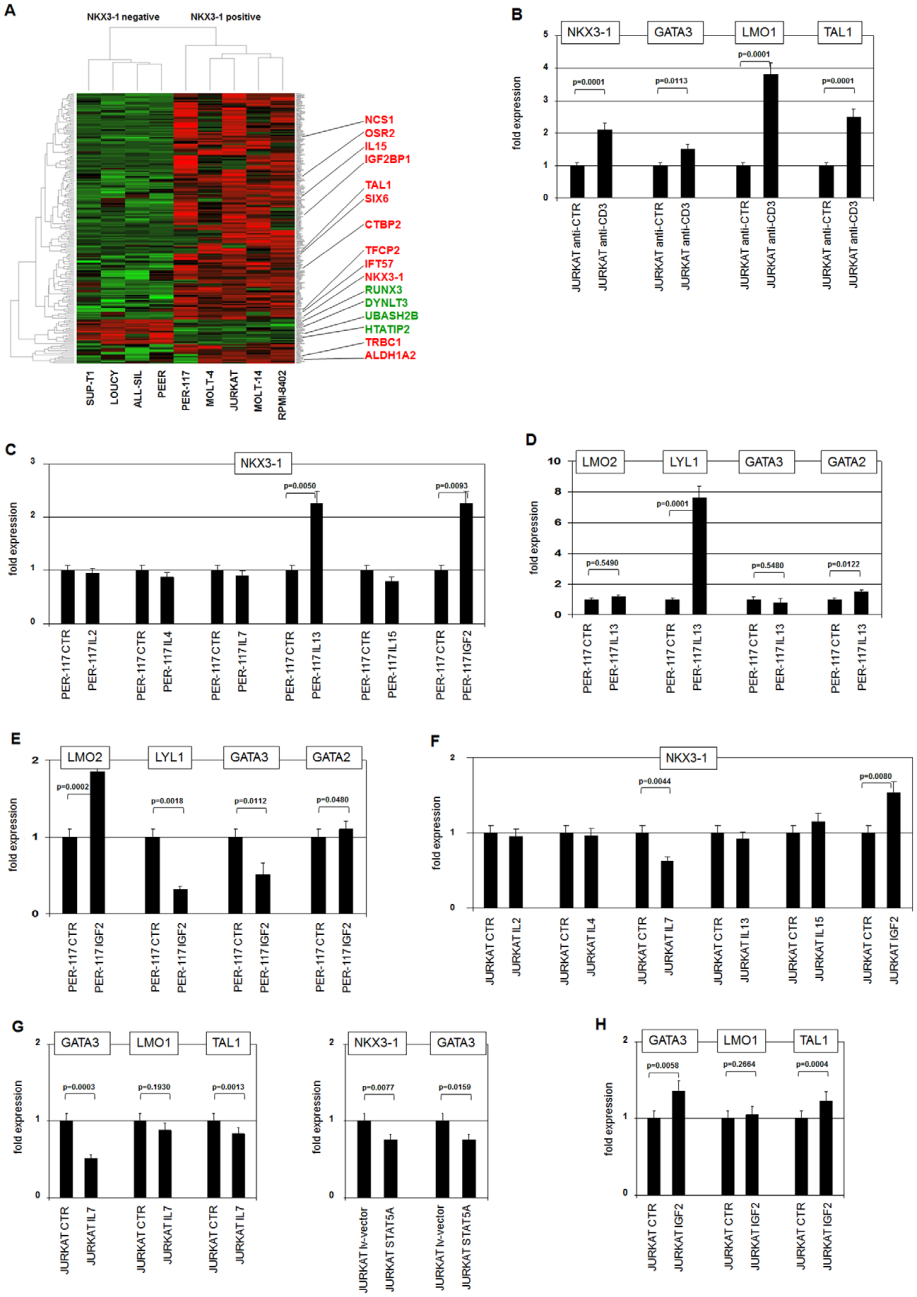
Identification of additional NKX3-1 regulators. (**A**) Heat map of 209 genes which displayed a significant differential expression between two groups of T-ALL cell lines concerning NKX3-1 expression, as analysed by the LIMMA package. Four T-ALL cell lines (SUP-T1, LOUCY, ALL-SIL, PEER) are NKX3-1 negative while five others (PER-117, MOLT-4, JURKAT, MOLT-14, RPMI-8402) are NKX3-1 positive. Red indicates strong expression levels, green low levels and black medium levels. Sixteen conspicuously expressed genes are highlighted. (**B**) RQ-PCR analysis of JURKAT cells treated with activating anti-CD3 antibody demonstrates concomitantly upregulation of NKX3-1, GATA3, LMO1 and TAL1. (**C**) Treatment of PER-117 cells with specific ligands demonstrates increased expression of NKX3-1 after stimulation with IL13 or IGF2. (**D**) Treatment of PER-117 cells with IL13 resulted in strong activation of LYL1 and slight activation of GATA2 expression, while that of LMO2 and GATA3 remained unperturbed. (**E**) Treatment of PER-117 cells with IGF2 resulted in differentially altered expression levels of LMO2, LYL1 and GATA3 while GATA2 remained unchanged. (**F**) Treatment of JURKAT cells with specific ligands demonstrates decreased expression of NKX3-1 after stimulation with IL7, while the expression increased after stimulation with IGF2. (**G**) Treatment of JURKAT cells with IL7 resulted in decreased expression of GATA3, LMO1 and TAL1 (left). Lentiviral overexpressed STAT5A in JURKAT cells resulted in decreased expression of both NKX3-1 and GATA3 (right). (**H**) Treatment of JURKAT cells with IGF2 resulted in marginally increased expression of GATA3 and TAL1 while no change was visible for LMO1.

To characterize additional upstream activators and to identify downstream targets of NKX3-1 we analyzed expression profiling data of T-ALL cell lines. Comparison of five NKX3-1 positive cell lines with four NKX3-1 negative ones using the R-based LIMMA package revealed 209 probe sets of differentially expressed genes (p<0.003). Consistently, the upregulated genes included NKX3-1 and TAL1 (**Table S1,** **Fig. 4A**). Selected gene candidates which may be involved in regulation of NKX3-1 expression or represent potential target genes are listed in **Table 3**. In the following we tested the impact of these identified genes.

**Table 3:** Selected genes differentially expressed in NKX3-1 positive cell lines.

gene	function	effect	category	reference
**upregulated genes in NKX3-1 positive cell lines:**				
TAL1	activates NKX3-1, TF-complex	differentiation	u	Kusy et al. 2010
TRBC1	T-cell receptor beta	TCR-signalling	u	
IFT57	TCR/CD3-complex	TCR-signalling	u	Finetti et al., 2011
NCS1	enhances Ca++ signaling	TCR-signalling	u	Nakamura et al., 2011
IL15	T/NK-cell differentiation	STAT3/5-signalling	u	Fehniger et al., 2001
CTBP2	inhibits IL2	STAT5-signalling	u	Wang et al., 2009
TFCP2	activates IL4 and IL13	STAT6-signalling	u	Casolaro et al., 2000
OSR2	inhibits BMP4-MSX-pathway	BMP4-signalling/MSX	u	Zhang et al., 2009
IGF2BP1	IGF2-binding	IGF2-signalling	u	
SIX6	retinal expression, TF	differentiation	d	Kumar, 2009
ALDH1A2	ALDH1A1 is TLX1-target	differentiation	d	Rice et al., 2008
**downregulated genes in NKX3-1 positive cell lines:**				
UBASH2B	inhibits TCR-signaling	TCR-signaling	u	Carpino et al., 2004
RUNX3	inhibits IL4 (IL13)	STAT6-signalling	u	Lee et al., 2009
	IL-7 activates RUNX3	STAT5-signalling	u	Park et al., 2010
DYNLT3	inhibits mitosis	proliferation	d	Lo et al., 2007
HTATIP2	pro-apoptotic	survival	d	Xiao et al., 2000

u: potential upstream regulator of NKX3-1; d: potential downstream target of NKX3-1.

TRBC1 (T-cell receptor beta constant 1), IFT57 (intraflagellar transport 57) and NCS1 (neuronal calcium sensor 1) were upregulated and UBASH2/STS1 (ubiquitin associated and SH3 domain containing 2) was downregulated in NKX3-1 positive cell lines and involved in TCR signalling [35]–[37]. To analyze the potency of this pathway in NKX3-1 expression we treated T-ALL cells with an activating antibody against TCR-coreceptor CD3. Of note, the immature T-cell line PER-117 does neither express TCR nor CD3 in contrast to JURKAT cells analyzed here (**Fig. S4A**). In comparison to the control, stimulation of CD3 resulted in enhanced NKX3-1 expression. Moreover, coactivation of GATA3, LMO1 and TAL1 indicates NKX3-1 regulation by TCR/CD3 via this TF complex (**Fig. 4B**).

IL15 (interleukin 15), CTBP2 (C-terminal binding protein 2), TFCP2 (transcription factor CP2) and IGF2BP1 (insulin-like growth factor 2 mRNA binding protein 1) are upregulated and RUNX3 (runt-related transcription factor 3) downregulated in NKX3-1 positive cell lines. These genes are involved in IL/JAK/STAT- and IGF2-pathways (**Table 3**) [2], [38]–[42]. Expression array data demonstrated components from these signalling pathways in T-ALL cell lines (**Fig. S4B**). Their potential impact on NKX3-1 expression was analyzed by treatment of PER-117 and JURKAT cells with IL2, IL4, IL7, IL13, IL15 and IGF2. In PER-117 IL13 and IGF2 treatments resulted in increased NKX3-1 expression while the other stimulations showed no effect (**Fig. 4C**). Coactivation of GATA2 and LYL1 by IL13 indicated NKX3-1 regulation via strongly activated LYL1 (**Fig. 4D**). However, IGF2 stimulation resulted in increased expression of LMO2 and decreased levels of GATA3 but also in decreased expression of LYL1 (**Fig. 4E**), suggesting the involvement of another mediator. The treatment of JURKAT cells by this panel of factors resulted in decreased NKX3-1 levels by IL7 and increased levels by IGF2 (**Fig. 4F**). IL7 inhibited expression of GATA3, LMO1 and TAL1 as well (**Fig. 4G**), indicating regulation of NKX3-1 expression via these TFs. Repressive IL7 signalling in T-cells may be mediated via STAT5 [43]. Accordingly, overexpression of STAT5A in JURKAT inhibited both NKX3-1 and GATA3 (**Fig. 4G**). In PER-117 STAT5A inhibited NKX3-1 as well, as demonstrated by siRNA mediated knockdown and subsequent expression analyses of NKX3-1, GATA2, GATA3 and LYL1 (**Fig. S4C**). Stimulation of JURKAT cells with IGF2 resulted in no significant change of GATA3, LMO1 or TAL1 expression (**Fig. 4H**), resembling PER-117. Taken together, regulation of NKX3-1 via TCR/CD3-, IL13- and IL7-signalling is mediated by TFs TAL1/LYL1 and GATA3/2, while these factors are not part of the IGF2-pathway.

**Figure 5:**
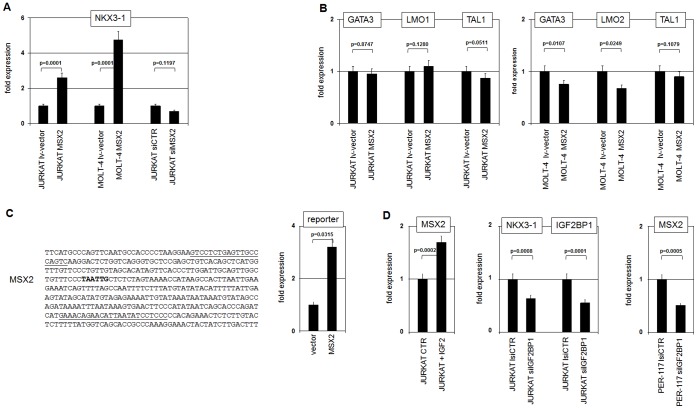
MSX2 activates NKX3-1 expression. (**A**) JURKAT and MOLT-4 cells were lentivirally transfected for overexpression of MSX2. Additionally, JURKAT were treated for knockdown of MSX2. Collectively, the data demonstrate the positive impact of MSX2 on NKX3-1 expression. (**B**) Overexpression of MSX2 in JURKAT cells showed no significant alteration in analyzed gene expression (left), while the same treatment in MOLT-4 cells indicated decreased expression of GATA3 and LMO2 but not of TAL1 (right). (**C**) Reporter gene assay of a genomic NKX3-1 upstream fragment (left) demonstrates stimulation by MSX2 as analyzed by RQ-PCR in PEER cells. The oligonucleotide sequences used for cloning of the reporter gene construct are underlined and the binding site for MSX2 is shown in bold. (**D**) IGF2 treatment resulted in increased expression of MSX2 in JURKAT cells (left). SiRNA mediated knockdown of IGF2BP1 in JURKAT cells resulted in reduced expression of NKX3-1, indicating a stimulatory input. SiRNA mediated knockdown of IGF2BP1 in PER-117 resulted in reduced expression of MSX2, indicating an activatory input of IGF2BP1 in IGF2-signalling.

MSX2 has been described as a target gene of IGF-signalling in odontoblasts and may play a role in T-cell development [15], [44]. Furthermore, OSR2 (odd-skipped related 2) is upregulated in NKX3-1 expressing cell lines and is a suppressor of the BMP/MSX-pathway (**Table 3**) [45]. Thus, to analyze the potential impact of MSX2 on NKX3-1 expression we measured NKX3-1 transcription in JURKAT and MOLT-4 cells subjected to MSX2 overexpression or knockdown. These data clearly demonstrate that MSX2 activates expression of NKX3-1 (**Fig. 5A**). Next we analyzed the role of MSX2, and showed the independence of GATA3, LMO1/2 and TAL1 expression from this factor (**Fig. 5B**). Sequence analysis of the NKX3-1 gene revealed a potential MSX2 binding site in the upstream region. Subsequent reporter gene assay using the corresponding genomic fragment demonstrated direct activation of NKX3-1 by MSX2 (**Fig. 5C**). Moreover, treatment of JURKAT cells with IGF2 resulted in enhanced expression of MSX2 (**Fig. 5D**) and siRNA mediated knockdown of IGF2BP1 decreased expression levels of both MSX2 and NKX3-1 (**Fig. 5D**). Thus, IGF2BP1 displayed an activating role for IGF2-signalling. Taken together, these data indicate that IGF2-signalling mediates enhancement of MSX2 expression which in turn directly activates transcription of NKX3-1. Of note, MSX2 has been described downstream of BMP4-signalling in T-cells as well [15]. Therefore, BMP4 inhibited the expression of NKX3-1 most likely by reduction of MSX2 as shown previously (**Fig. 2C**) [15].

### Identification of NKX3-1 Target Genes

**Figure 6:**
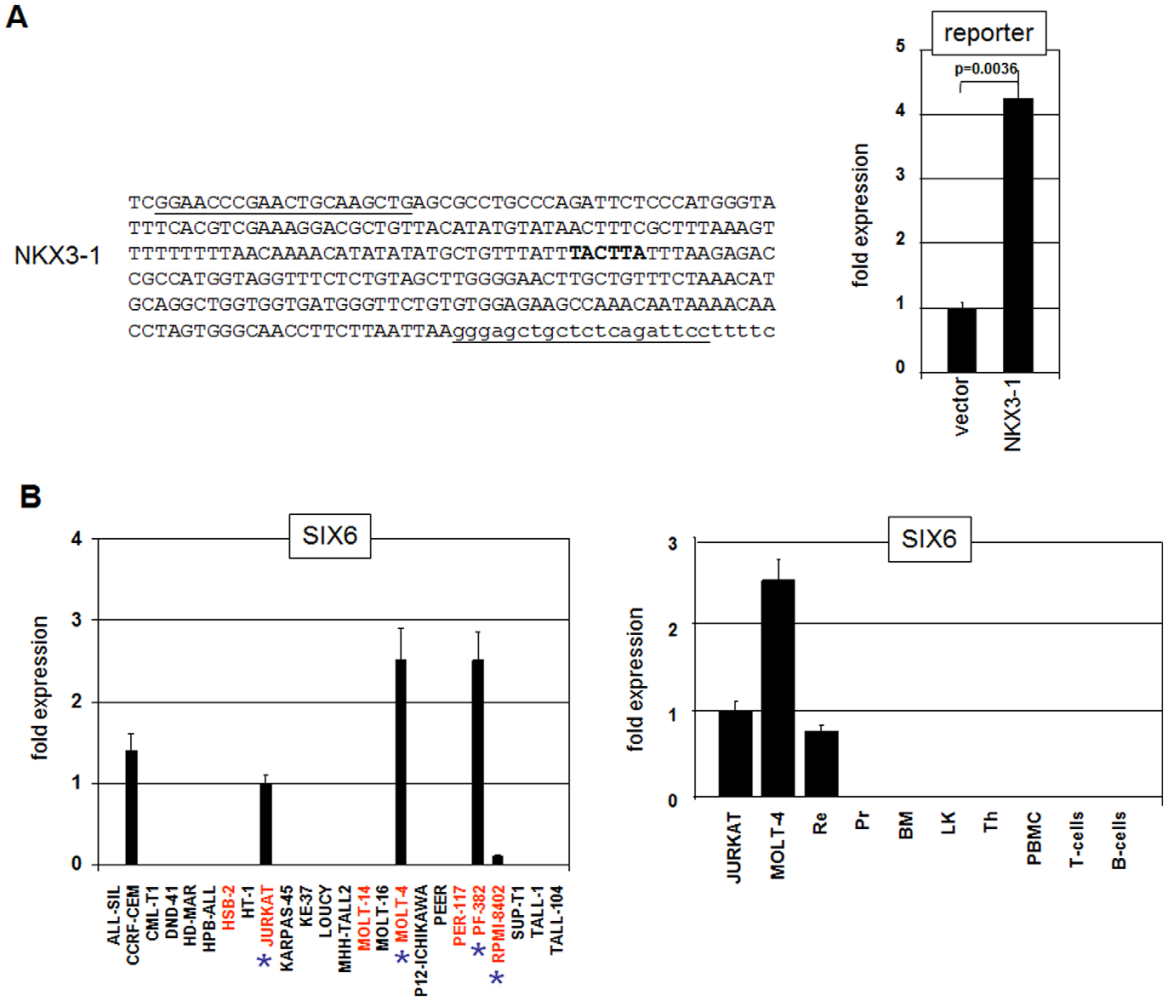
NKX3-1 activates SIX6 expression. (**A**) Reporter gene assay of a genomic SIX6 fragment located in exon 2 (left) demonstrates an activatory input of NKX3-1 as analyzed by RQ-PCR in PEER cells. The oligonucleotide sequences used for cloning of the reporter gene construct are underlined and the binding site for NKX3-1 is emboldened. (**B**) RQ-PCR analysis of SIX6 expression in T-ALL cell lines (left) and primary cells (right) demonstrates SIX6 transcripts in 5 cell lines and primary retina cells but not in hematopoietic cells. NKX3-1 positive cell lines are in red, coexpressing cell lines are marked with an asterix.

Finally, we analyzed the activity of NKX3-1 in regulating putative target genes. Expression profiling analysis revealed plausible candidate genes, including ALDH1A2, DYNLT3, HTATIP2/TIP30 and SIX6 (**Fig. 4A** **, Table S1,** **Table 3**). ALDH1A2 (aldehyde dehydrogenase 1 family, member A2) represents a paralog of ALDH1A1 which is involved in TLX1-positive T-ALL [46]. DYNLT3 (dynein light chain Tctex-type 3) and HTATIP2 (HIV-1 Tat interactive protein 2) inhibit proliferation and survival, respectively, suggesting tumor suppressor activity [47], [48]. SIX6 (SIX homeobox 6) encodes a TF which is involved in the development of retinal structures and has been detected in T-ALL patients coexpressing NKX3-1 [14], [49]. Interestingly, according to the UCSC genome browser (www.genome.cse.ucsc.edu), the SIX6 gene contains a potential binding site for NKX3-1 in exon 2. Subsequent reporter gene assay using the corresponding genomic fragment confirmed direct activation of SIX6 by NKX3-1 (**Fig. 6A**). The expression of SIX6 was detected in five T-ALL cell lines, three of which coexpressed NKX3-1 (**Fig. 6B**). The expression level of SIX6 in T-ALL cell lines JURKAT and MOLT-4 respectively matches or exceeds twofold or more primary cells of human retina (**Fig. 6B**). These data reveal prominent SIX6 transcripts in T-ALL cells. Furthermore, SIX6 transcripts were neither detected in the prostate nor in hematopoietic cells, demonstrating ectopic expression in T-ALL cells (**Fig. 6B**). Of note, NKX3-1 expression was equally silent in retinal cells (**Fig. 1A**), discounting their reciprocal activation under physiological conditions. Taken together, our results indicate that SIX6 represents a direct leukemic target gene of NKX3-1 in T-ALL cells. However, that merely 3/5 SIX6 positive T-ALL cell lines coexpress NKX3-1 and SIX6 suggests that additional factors regulate SIX6 expression.

## Discussion

Here, we analyzed mechanisms of aberrant NKX3-1 expression in T-ALL. We found no hint of genomic imbalances or chromosomal rearrangements which contrasts with other known NKL homeobox genes aberrantly expressed in T-ALL. Nevertheless, the expression level of leukemic NKX3-1 was similar to that of chromosomally activated NKX2-5 when set against their physiological tissue controls. NKX3-1 is physiologically expressed in prostate where it is activated by particular TFs and signalling pathways [24], [25]. Our data, however, yielded no hint for the activity of prostate specific TFs or pathways underlying aberrant NKX3-1 expression in T-ALL cells.

We confirmed the activating input of TAL1 in concert with GATA3 and LMO1/2, constituting a transcription factor complex which regulates fundamental processes in hematopoiesis [26]. The makeup of this complex depends on ontogenic cell status and cell type and comprises diverse permutations of bHLH proteins, GATA- and LMO-factors [7]–[11]. Interestingly, the bHLH protein LYL1 could not substitute the bHLH family member TAL1. LYL1 activated NKX3-1 transcription in the absence of GATA3 apparently without promotion of that TF complex. Moreover, LYL1 inhibited the expression of NKX3-1 in combination with GATA3, indicating fundamentally distinct and exclusive activation mechanisms. Furthermore, we confirmed the positive input of GATA2 in LYL1 expression as described previously [34], which was therefore deemed important for expression of NKX3-1 in immature T-ALL cells.

Profiling data on T-ALL patients have shown coexpression of NKX3-1 and TAL1 or MLL fusion proteins in addition to cases forming an immature type of T-ALL [14]. Accordingly, our data for T-ALL cell lines JURKAT (TAL1-positive) and PER-117 (LYL1-positive) may correspond to TAL1-positive and immature T-ALL subtypes, respectively. Our data concerning MLL and MLL fusions indicate that MLL inhibits basic NKX3-1 activators. Although MLL translocations leave NKX3-1 transcription unperturbed, but rather deplete wild type MLL, these rearrangements potentially play an indirect activatory role in NKX3-1 expression. T-ALL patients with MLL translocations consistently express enhanced levels of GATA3 as reported previously [50]. Similarly, signalling pathways activated by TCR/CD3, IL13 and IL7 mediated the expression levels of TAL1, LYL1, GATA3, LMO1, and LMO2, regulating NKX3-1 expression by modulation of direct activators.

Homeodomain protein MSX2 was identified as an additional factor for NKX3-1 activation. MSX2 binds the NKX3-1 upstream region, evidencing direct regulation. Furthermore, MSX2 was identified as a downstream target of IGF2-signalling in T-ALL cells in addition to BMP4-signalling as described previously [15]. IGF2 mediated activation of NKX3-1 transcription while BMP4 inhibited its expression. Our data indicate that the potency of IGF2 is enhanced by IGF2BP1. Both factors, IGF2 and BMP4, are physiologically expressed in the thymus and regulate early stages of developing T-cells [1], [2]. Accordingly, MSX2 has been proposed as a physiological NKL homeodomain-factor in early T-cell development [15]. Therefore, elevated MSX2 activity realized by enhanced IGF2-signalling and/or reduced BMP4-signalling may thus correlate with the immature type of T-ALL.

**Figure 7:**
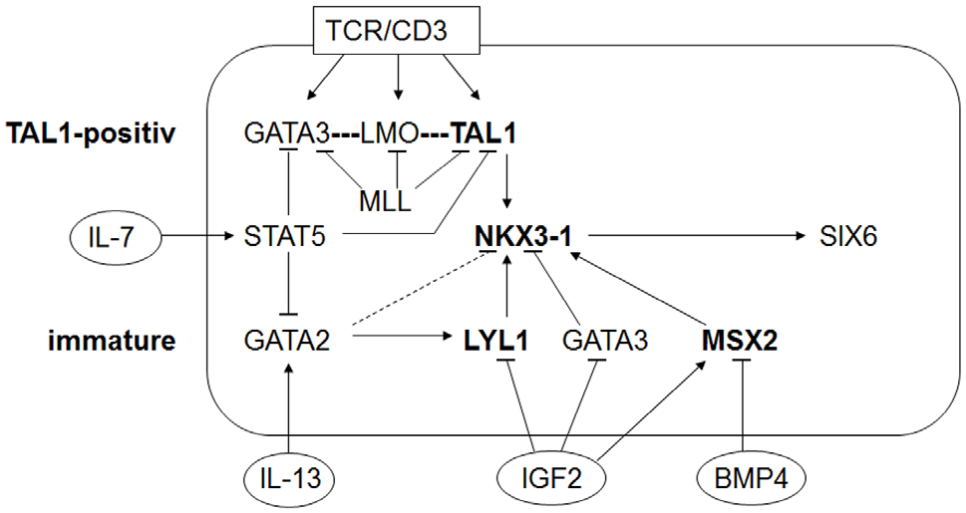
Leukemic network of NKX3-1. The figure summarizes the results of this study, highlighting the major activators of NKX3-1 in bold, comprising TAL1, LYL1 and MSX2, and their potential association to the T-ALL subtypes TAL1-positive and immature. T-ALL cell line JURKAT expresses TAL1, GATA3 and the TCR-complex while cell line PER-117 expresses LYL1 and GATA2. Signalling pathways BMP4 and IGF2 regulate expression of MSX2 and are associated to early stages of T-cell development.

Collectively, we have described three mechanisms of leukemic activation of homeobox gene NKX3-1 in T-ALL cells represented by TAL1, LYL1 and MSX2 as summarized in **Figure 7**. These mechanisms may correspond to the TAL1-positive and immature T-ALL subtypes, explaining the association of aberrant NKX3-1 expression with distinct types of T-ALL. Ectopic activation of NKX3-1 in leukemic cells is realized by aberrant TF activity. This kind of activation represents a novel type of homeobox gene deregulation in T-ALL. While TLX1, NKX2-5 and HOXA are activated by chromosomal juxtaposition to activatory elements [14], [20], [22], and PITX1 by chromosomal deletion of repressive elements [51], NKX3-1 appears to be activated without alteration of cis-regulatory regions.

Finally, we identified homeobox gene SIX6 as a direct target of NKX3-1 in T-ALL cells (**Fig. 7**). SIX6 regulates differentiation processes of the retina [49], but physiologically is neither expressed in hematopoietic nor in prostate cells. The presence of SIX6 in T-ALL patients has been described previously associated partly with NKX3-1 expression [14]. Our data may thus give a mechanistic explanation for this relationship. However, functional consequences of the deregulated expression of homeobox genes NKX3-1 and SIX6 in T-ALL remain elusive, although NKX3-1 has been described to regulate the miR-cluster 17∼92, and SIX6 the gene CDNK1B encoding cell cycle inhibitor p27 – both regulating proliferation [26], [52]. However, the involvement of NKX3-1 and SIX6 in developmental processes may suggest a deregulating role in thymocyte differentiation. Accordingly, SIX6 interacts with corepressor TLE/Groucho, contributing to suppression of non-retinal differentiation genes [49]. This interacting potential has been described for leukemic NKL homeodomain proteins as well which might represent, therefore, a basic pathologic trait in T-ALL [15], [53].

In conclusion, our data demonstrate three mechanisms for deregulating homeobox gene NKX3-1 and its subsequent target gene SIX6 in T-ALL. These mechanisms reflect TAL1-positve and immature T-ALL subtypes and may represent a novel type of homeobox gene deregulation in T-ALL, lacking cis-regulatory changes. Our results may contribute to the understanding of aberrant networks, their role in constitution of leukemic subtypes and the subsequent improvement of therapeutic protocols in T-ALL.

## Supporting Information

Figure S1 **Chromosomal analysis of NKX3-1 locus.** FISH analyses of additional NKX3-1 expressing T-ALL cell lines (MOLT-4, MOLT-14, PF-382, HSB-2 and RPMI-8402) using flanking and straddling probes of the NKX3-1 locus indicate wild type configurations.
(TIF)

Figure S2 **Expression analysis of TFs and signalling components.** (**A**) Data of expression analyses obtained by profiling for genes encoding TFs NKX3-1, FOXA1, SOX4 and ETS1 in T-ALL cell lines were transformed into a heat map (above). Red indicates high expression levels, black medium and green low levels. Expression analyses of FOXA1 by RQ-PCR in T-ALL cell lines (left) and in three prostate cell lines in comparison to RPMI-8402 (right) show elevated levels in RPMI-8402, which are, however, much lower when compared to prostate cells. (**B**) Heat map of expression profiling data from T-ALL cell lines demonstrates expression intensities of signalling-pathway components.
(TIF)

Figure S3 **LMO4 expression.** Quantification of gene expression for LMO4 in T-ALL cell lines by RQ-PCR. NKX3-1 expressing cell lines are indicated in red letters.
(TIF)

Figure S4 **Analysis of signalling components.** (**A**) Expression analyses of TCR and CD3 genes. The heat map (above) displays expression profiling data of T-ALL cell lines. Red indicates high expression levels, black medium and green low levels. Flow cytometry data (below) demonstrates significant CD3 protein expression in JURKAT (92%) but absent from PER-117. (**B**) The heat map shows expression profiling data of T-ALL cell lines for several IL/STAT- and IGF/IGFR-genes. (**C**) PER-117 cells were treated for siRNA mediated knockdown of STAT5A and STAT3. Collectively, the data indicate STAT5-mediated inhibition of GATA2, GATA3, LYL1 and NKX3-1.
(TIF)

Table S1 **Potential NKX3-1 regulator- and target-genes.**
(DOC)
