# Supplementary figures and images for: Transcriptional Activation of Prostate Specific Homeobox Gene NKX3-1 in Subsets of T-Cell Lymphoblastic Leukemia (T-ALL)
Source: PLoS One. 2012 Jul 27;7(7):e40747. doi: 10.1371/journal.pone.0040747 (PMC3407137; doi:10.1371/journal.pone.0040747)

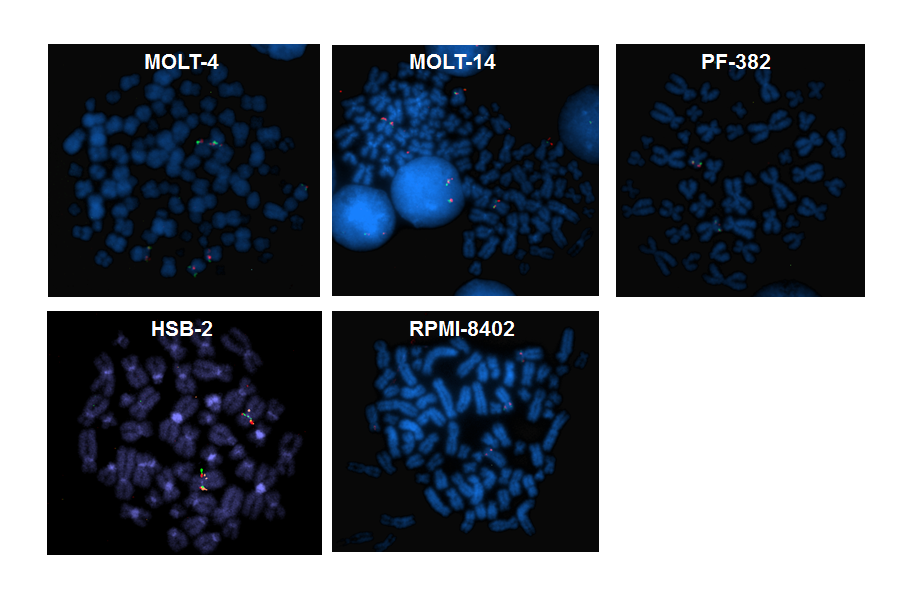

Supplement: Figure S1 — Chromosomal analysis of NKX3-1 locus. FISH analyses of additional NKX3-1 expressing T-ALL cell lines (MOLT-4, MOLT-14, PF-382, HSB-2 and RPMI-8402) using flanking and straddling probes of the NKX3-1 locus indicate wild type configurations. (TIF) [file pone.0040747.s001.tif]

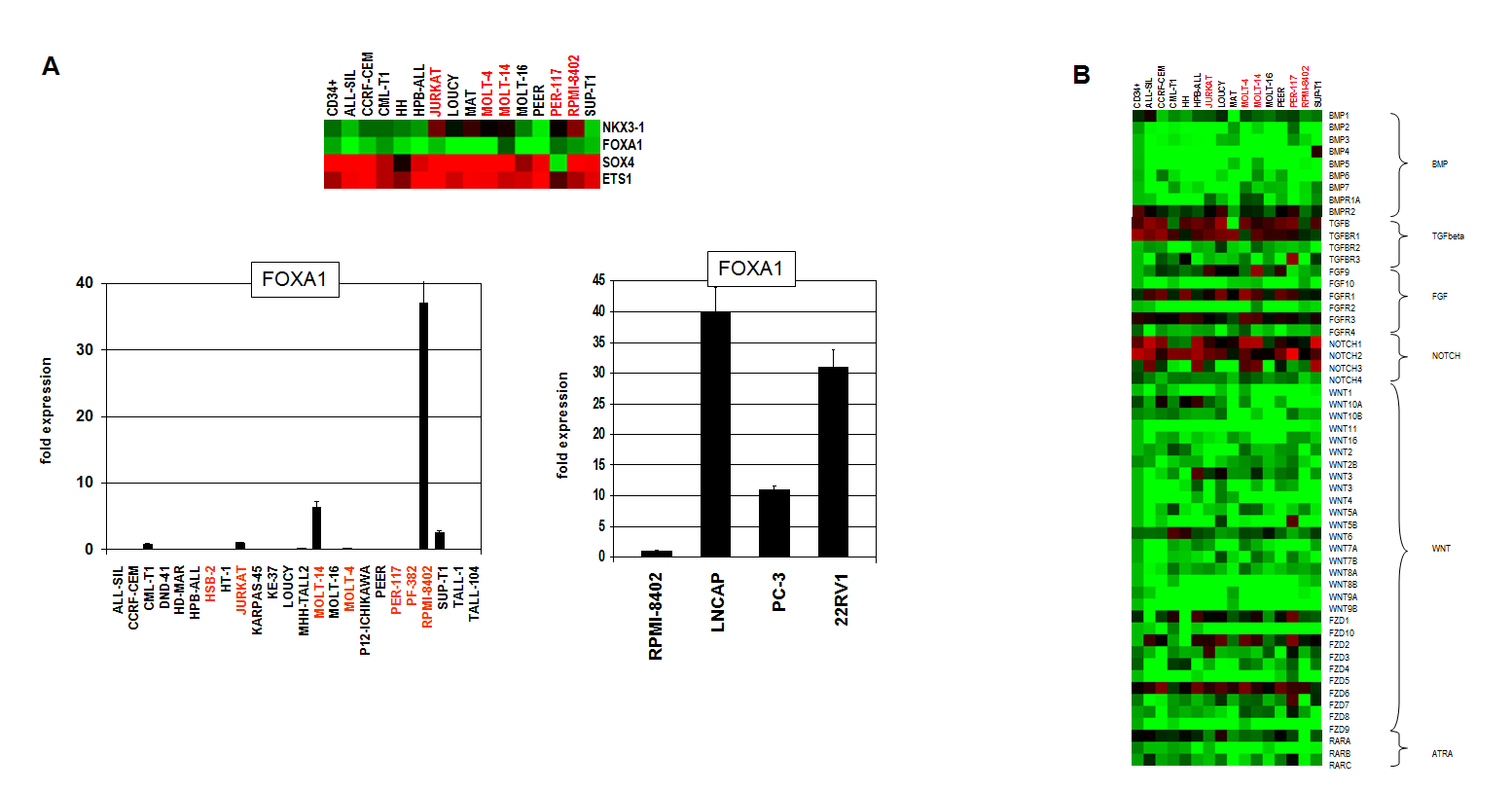

Supplement: Figure S2 — Expression analysis of TFs and signalling components. (A) Data of expression analyses obtained by profiling for genes encoding TFs NKX3-1, FOXA1, SOX4 and ETS1 in T-ALL cell lines were transformed into a heat map (above). Red indicates high expression levels, black medium and green low levels. Expression analyses of FOXA1 by RQ-PCR in T-ALL cell lines (left) and in three prostate cell lines in comparison to RPMI-8402 (right) show elevated levels in RPMI-8402, which are, however, much lower when compared to prostate cells. (B) Heat map of expression profiling data from T-ALL cell lines demonstrates expression intensities of signalling-pathway components. (TIF) [file pone.0040747.s002.tif]

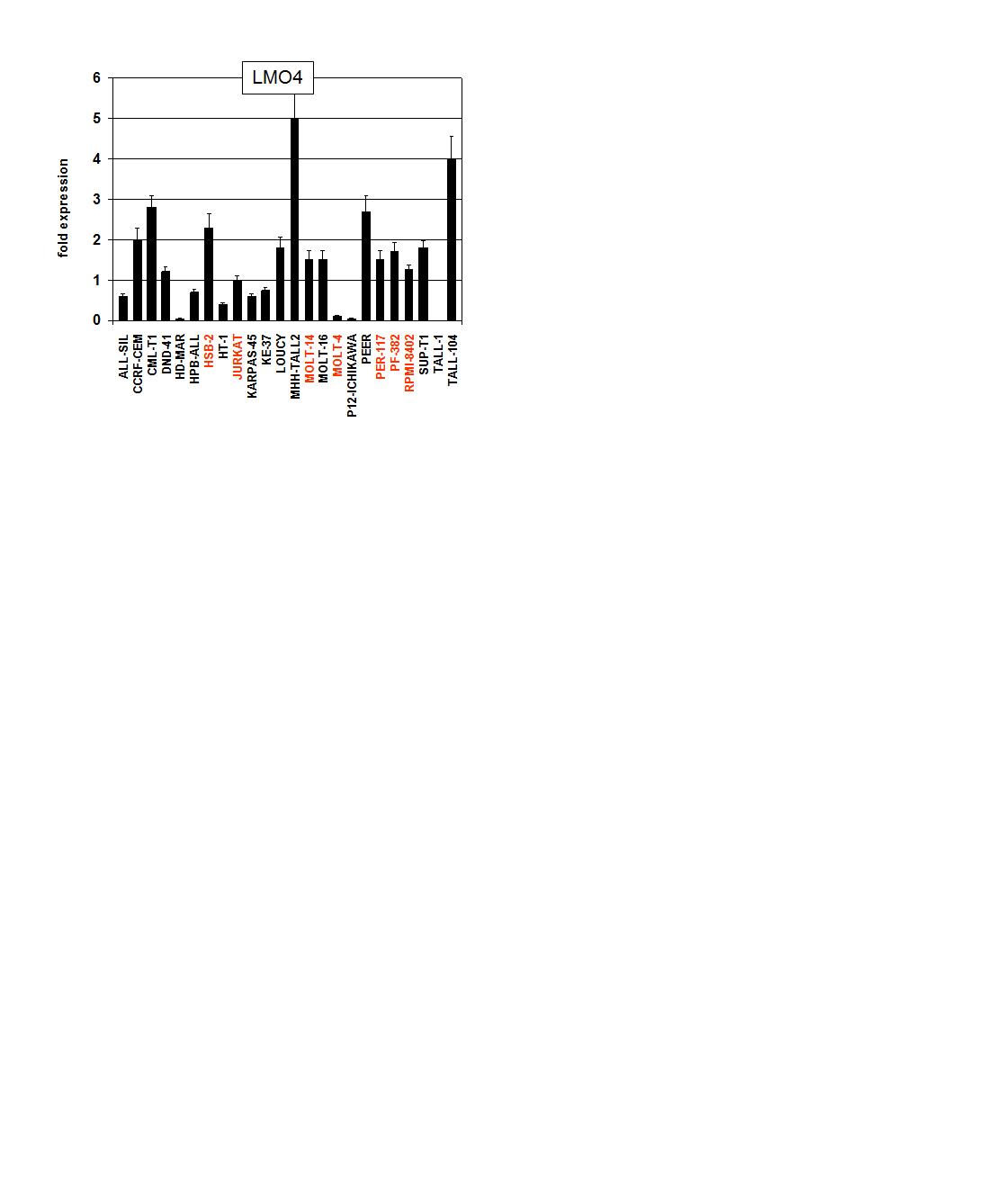

Supplement: Figure S3 — LMO4 expression. Quantification of gene expression for LMO4 in T-ALL cell lines by RQ-PCR. NKX3-1 expressing cell lines are indicated in red letters. (TIF) [file pone.0040747.s003.tif]

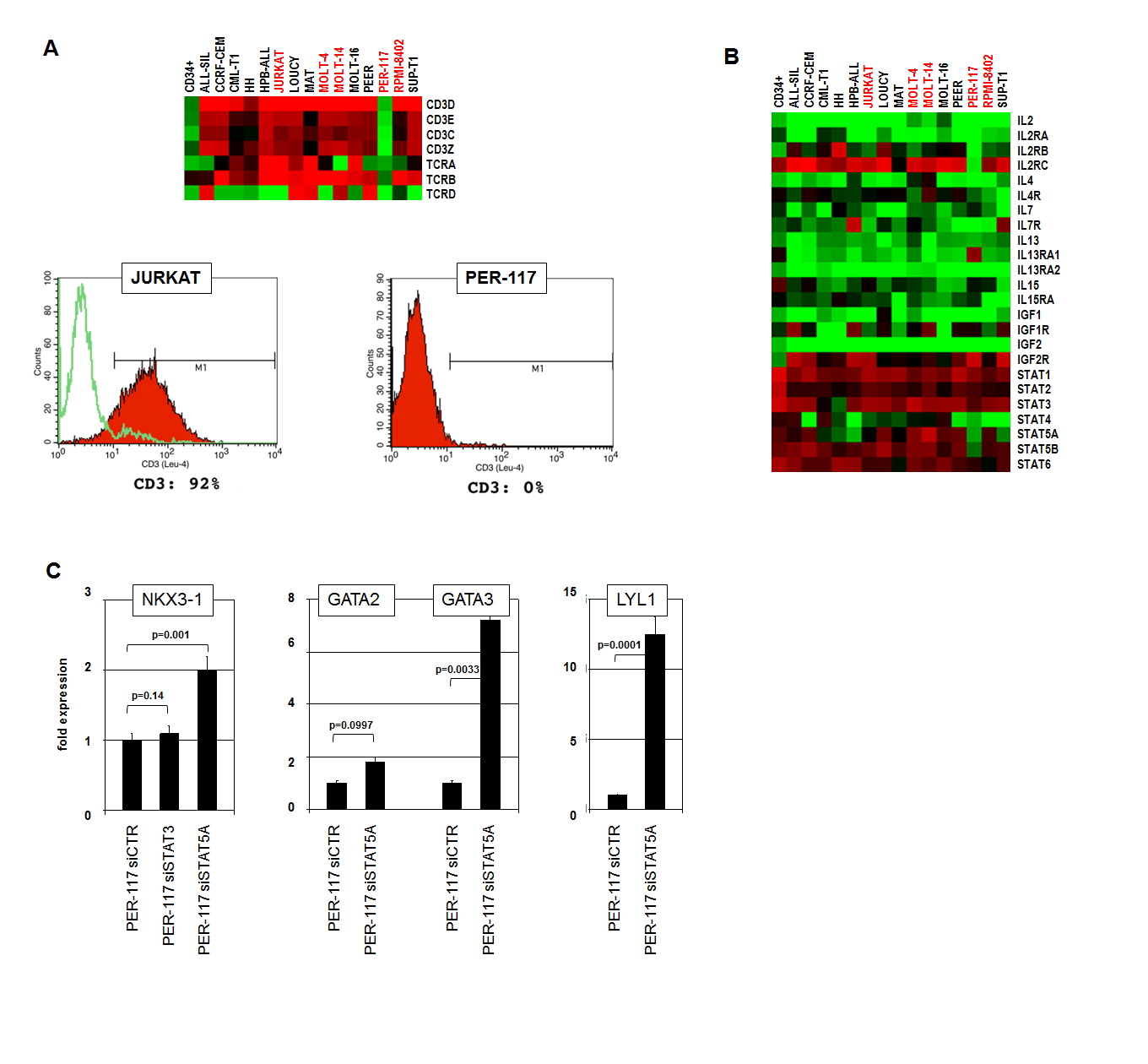

Supplement: Figure S4 — Analysis of signalling components. (A) Expression analyses of TCR and CD3 genes. The heat map (above) displays expression profiling data of T-ALL cell lines. Red indicates high expression levels, black medium and green low levels. Flow cytometry data (below) demonstrates significant CD3 protein expression in JURKAT (92%) but absent from PER-117. (B) The heat map shows expression profiling data of T-ALL cell lines for several IL/STAT- and IGF/IGFR-genes. (C) PER-117 cells were treated for siRNA mediated knockdown of STAT5A and STAT3. Collectively, the data indicate STAT5-mediated inhibition of GATA2, GATA3, LYL1 and NKX3-1. (TIF) [file pone.0040747.s004.tif]
