# Supplementary material for: Transcriptional Activation of Prostate Specific Homeobox Gene NKX3-1 in Subsets of T-Cell Lymphoblastic Leukemia (T-ALL)
Source: PLoS One. 2012 Jul 27;7(7):e40747. doi: 10.1371/journal.pone.0040747 (PMC3407137; doi:10.1371/journal.pone.0040747)
Supplement: Table S1 — Potential NKX3-1 regulator- and target-genes. (DOC) [file pone.0040747.s005.doc]

# Table S1: Potential NKX3-1 regulator- and target-genes

| **Probe** | **Symbol** | **Description** | **Chromosome** | **Chromosome Location** | **GenBank** | **Gene** | **Cytoband** | **UniGene** | **PubMed** | **Gene Ontology** | **Pathway** |
| --- | --- | --- | --- | --- | --- | --- | --- | --- | --- | --- | --- |
| [1007_s_at](https://www.affymetrix.com/LinkServlet?&probeset=1007_s_at) | DDR1 | discoidin domain receptor tyrosine kinase 1 | 6 | 30851860, 30852326, 30856464, 2363958, 2364424, 2368562, 2145962, 2146428, 2150566, 2199945, 2200411, 2204549, 2233817, 2234283, 2144847, 2145313, 2149451 | [U48705](http://www.ncbi.nlm.nih.gov/entrez/query.fcgi?cmd=search&db=nucleotide&term=U48705%5BACCN%5D&doptcmdl=GenBank) | [780](http://www.ncbi.nlm.nih.gov/sites/entrez?Db=gene&Cmd=DetailsSearch&Term=780) | [6p21.3](http://www.ncbi.nlm.nih.gov/mapview/map_search.cgi?direct=on&idtype=gene&id=780) | [Hs.631988](http://www.ncbi.nlm.nih.gov/UniGene/clust.cgi?ORG=Hs&CID=631988) | [74](http://www.ncbi.nih.gov/entrez/query.fcgi?tool=bioconductor&cmd=Retrieve&db=PubMed&list_uids=7774938%2C7789998%2C7834423%2C7848919%2C8139570%2C8226977%2C8302582%2C8390675%2C8622863%2C8682498%2C8796349%2C8977099%2C9396043%2C9492013%2C9659899%2C9659900%2C10681566%2C10783152%2C11126911%2C11133186%2C11344127%2C11472367%2C11606478%2C12477932%2C12935821%2C14574404%2C14764702%2C15111304%2C15136580%2C15146197%2C15213330%2C15240533%2C15489334%2C16169070%2C16337946%2C16440311%2C16702430%2C16774916%2C16912190%2C17001518%2C17027969%2C17101694%2C17299390%2C17440435%2C17440927%2C17704737%2C17721511%2C17970783%2C17982627%2C18023033%2C18065762%2C18190796%2C18270328%2C18362184%2C18593464%2C18836851%2C19262089%2C19401332%2C19752756%2C19837266%2C19851445%2C20007060%2C20041166%2C20182441%2C20219323%2C20353877%2C20372823%2C20380825%2C20487506%2C20587610%2C20596615%2C20799954%2C20920467%2C21170030) | [nucleotide binding](http://amigo.geneontology.org/cgi-bin/amigo/go.cgi?view=details&query=GO:0000166)  [regulation of cell growth](http://amigo.geneontology.org/cgi-bin/amigo/go.cgi?view=details&query=GO:0001558)  [regulation of cell-matrix adhesion](http://amigo.geneontology.org/cgi-bin/amigo/go.cgi?view=details&query=GO:0001952)  [transmembrane receptor protein tyrosine kinase activity](http://amigo.geneontology.org/cgi-bin/amigo/go.cgi?view=details&query=GO:0004714)  [receptor activity](http://amigo.geneontology.org/cgi-bin/amigo/go.cgi?view=details&query=GO:0004872)  [protein binding](http://amigo.geneontology.org/cgi-bin/amigo/go.cgi?view=details&query=GO:0005515)  [ATP binding](http://amigo.geneontology.org/cgi-bin/amigo/go.cgi?view=details&query=GO:0005524)  [extracellular region](http://amigo.geneontology.org/cgi-bin/amigo/go.cgi?view=details&query=GO:0005576)  [plasma membrane](http://amigo.geneontology.org/cgi-bin/amigo/go.cgi?view=details&query=GO:0005886)  [integral to plasma membrane](http://amigo.geneontology.org/cgi-bin/amigo/go.cgi?view=details&query=GO:0005887)  [cell adhesion](http://amigo.geneontology.org/cgi-bin/amigo/go.cgi?view=details&query=GO:0007155)  [transmembrane receptor protein tyrosine kinase signaling pathway](http://amigo.geneontology.org/cgi-bin/amigo/go.cgi?view=details&query=GO:0007169)  [embryo implantation](http://amigo.geneontology.org/cgi-bin/amigo/go.cgi?view=details&query=GO:0007566)  [negative regulation of cell proliferation](http://amigo.geneontology.org/cgi-bin/amigo/go.cgi?view=details&query=GO:0008285)  [basolateral plasma membrane](http://amigo.geneontology.org/cgi-bin/amigo/go.cgi?view=details&query=GO:0016323)  [transferase activity](http://amigo.geneontology.org/cgi-bin/amigo/go.cgi?view=details&query=GO:0016740)  [peptidyl-tyrosine phosphorylation](http://amigo.geneontology.org/cgi-bin/amigo/go.cgi?view=details&query=GO:0018108)  [organ regeneration](http://amigo.geneontology.org/cgi-bin/amigo/go.cgi?view=details&query=GO:0031100)  [ear development](http://amigo.geneontology.org/cgi-bin/amigo/go.cgi?view=details&query=GO:0043583)  [skin development](http://amigo.geneontology.org/cgi-bin/amigo/go.cgi?view=details&query=GO:0043588)  [response to protein stimulus](http://amigo.geneontology.org/cgi-bin/amigo/go.cgi?view=details&query=GO:0051789)  [branching involved in mammary gland duct morphogenesis](http://amigo.geneontology.org/cgi-bin/amigo/go.cgi?view=details&query=GO:0060444)  [mammary gland alveolus development](http://amigo.geneontology.org/cgi-bin/amigo/go.cgi?view=details&query=GO:0060749) |  |
| [1553972_a_at](https://www.affymetrix.com/LinkServlet?&probeset=1553972_a_at) | CBS | cystathionine-beta-synthase | 21 | -44473300, -44473300 | [BC007257](http://www.ncbi.nlm.nih.gov/entrez/query.fcgi?cmd=search&db=nucleotide&term=BC007257%5BACCN%5D&doptcmdl=GenBank) | [875](http://www.ncbi.nlm.nih.gov/sites/entrez?Db=gene&Cmd=DetailsSearch&Term=875) | [21q22.3](http://www.ncbi.nlm.nih.gov/mapview/map_search.cgi?direct=on&idtype=gene&id=875) | [Hs.533013](http://www.ncbi.nlm.nih.gov/UniGene/clust.cgi?ORG=Hs&CID=533013) | [239](http://www.ncbi.nih.gov/entrez/query.fcgi?tool=bioconductor&cmd=Retrieve&db=PubMed&list_uids=404147%2C681363%2C840498%2C1301198%2C2894761%2C7506602%2C7564249%2C7598711%2C7611293%2C7635485%2C7762555%2C7849717%2C7903580%2C7929220%2C7967489%2C7981678%2C8022826%2C8353501%2C8528202%2C8755636%2C8803779%2C8990018%2C9156316%2C9266356%2C9361025%2C9383285%2C9466992%2C9590298%2C9790750%2C9889017%2C10215408%2C10338090%2C10408774%2C10434301%2C10462600%2C10791559%2C10830953%2C11013450%2C11074524%2C11149614%2C11173483%2C11204591%2C11292330%2C11310576%2C11341749%2C11359213%2C11359462%2C11434706%2C11457468%2C11483494%2C11528503%2C11575217%2C11672761%2C11748855%2C11758232%2C11872884%2C12007221%2C12015064%2C12020105%2C12082592%2C12124992%2C12154064%2C12173932%2C12180146%2C12186157%2C12228232%2C12269827%2C12379655%2C12393509%2C12413583%2C12427542%2C12439143%2C12477932%2C12529702%2C12642343%2C12649066%2C12725044%2C12815602%2C12855221%2C12889841%2C14670973%2C14977639%2C15009965%2C15082224%2C15228193%2C15354395%2C15365998%2C15489334%2C15503105%2C15520012%2C15544339%2C15554031%2C15719048%2C15748616%2C15755387%2C15772012%2C15889417%2C15922487%2C15972722%2C15975077%2C16007597%2C16013960%2C16115349%2C16189514%2C16205833%2C16259797%2C16274669%2C16328059%2C16363792%2C16375773%2C16422253%2C16470595%2C16479318%2C16505479%2C16541333%2C16601865%2C16709328%2C16780588%2C16791140%2C16792904%2C16941496%2C16953589%2C17035141%2C17087506%2C17119116%2C17160942%2C17311259%2C17311260%2C17319270%2C17327360%2C17352495%2C17436311%2C17540596%2C17548676%2C17553479%2C17601350%2C17621169%2C17686644%2C17726616%2C17891500%2C17956124%2C17993766%2C18029348%2C18060852%2C18203168%2C18278872%2C18398434%2C18427977%2C18447718%2C18454451%2C18614746%2C18620331%2C18622257%2C18635682%2C18636124%2C18676680%2C18708589%2C18776696%2C18785313%2C18792976%2C18799873%2C18818748%2C18830263%2C18839533%2C18936436%2C18950795%2C18977241%2C18977990%2C18988749%2C18992148%2C19010420%2C19019082%2C19019335%2C19019492%2C19048631%2C19064578%2C19074437%2C19112534%2C19161160%2C19166826%2C19170196%2C19238444%2C19267073%2C19370759%2C19424622%2C19429038%2C19447967%2C19493349%2C19527514%2C19531479%2C19559392%2C19593657%2C19625176%2C19657138%2C19657388%2C19683694%2C19692168%2C19722721%2C19729796%2C19737740%2C19906435%2C19913121%2C19948975%2C20031554%2C20031578%2C20056620%2C20066033%2C20082058%2C20140262%2C20160465%2C20217437%2C20237949%2C20301697%2C20308073%2C20346360%2C20379614%2C20453000%2C20458436%2C20506325%2C20544798%2C20559280%2C20565774%2C20601281%2C20615890%2C20628086%2C20634891%2C20638879%2C20670920%2C20707729%2C20717043%2C20718043%2C20737570%2C20883119%2C20890573%2C20939734%2C20948192%2C21045269%2C21055808%2C21062078) | [endochondral ossification](http://amigo.geneontology.org/cgi-bin/amigo/go.cgi?view=details&query=GO:0001958)  [cystathionine beta-synthase activity](http://amigo.geneontology.org/cgi-bin/amigo/go.cgi?view=details&query=GO:0004122)  [protein binding](http://amigo.geneontology.org/cgi-bin/amigo/go.cgi?view=details&query=GO:0005515)  [soluble fraction](http://amigo.geneontology.org/cgi-bin/amigo/go.cgi?view=details&query=GO:0005625)  [nucleus](http://amigo.geneontology.org/cgi-bin/amigo/go.cgi?view=details&query=GO:0005634)  [nucleolus](http://amigo.geneontology.org/cgi-bin/amigo/go.cgi?view=details&query=GO:0005730)  [cytoplasm](http://amigo.geneontology.org/cgi-bin/amigo/go.cgi?view=details&query=GO:0005737)  [cytosol](http://amigo.geneontology.org/cgi-bin/amigo/go.cgi?view=details&query=GO:0005829)  [cysteine biosynthetic process from serine](http://amigo.geneontology.org/cgi-bin/amigo/go.cgi?view=details&query=GO:0006535)  [L-serine metabolic process](http://amigo.geneontology.org/cgi-bin/amigo/go.cgi?view=details&query=GO:0006563)  [L-serine catabolic process](http://amigo.geneontology.org/cgi-bin/amigo/go.cgi?view=details&query=GO:0006565)  [cellular amino acid biosynthetic process](http://amigo.geneontology.org/cgi-bin/amigo/go.cgi?view=details&query=GO:0008652)  [lyase activity](http://amigo.geneontology.org/cgi-bin/amigo/go.cgi?view=details&query=GO:0016829)  [cysteine biosynthetic process via cystathionine](http://amigo.geneontology.org/cgi-bin/amigo/go.cgi?view=details&query=GO:0019343)  [transsulfuration](http://amigo.geneontology.org/cgi-bin/amigo/go.cgi?view=details&query=GO:0019346)  [L-cysteine catabolic process](http://amigo.geneontology.org/cgi-bin/amigo/go.cgi?view=details&query=GO:0019448)  [enzyme binding](http://amigo.geneontology.org/cgi-bin/amigo/go.cgi?view=details&query=GO:0019899)  [heme binding](http://amigo.geneontology.org/cgi-bin/amigo/go.cgi?view=details&query=GO:0020037)  [heme binding](http://amigo.geneontology.org/cgi-bin/amigo/go.cgi?view=details&query=GO:0020037)  [pyridoxal phosphate binding](http://amigo.geneontology.org/cgi-bin/amigo/go.cgi?view=details&query=GO:0030170)  [ubiquitin protein ligase binding](http://amigo.geneontology.org/cgi-bin/amigo/go.cgi?view=details&query=GO:0031625)  [response to nutrient levels](http://amigo.geneontology.org/cgi-bin/amigo/go.cgi?view=details&query=GO:0031667)  [identical protein binding](http://amigo.geneontology.org/cgi-bin/amigo/go.cgi?view=details&query=GO:0042802)  [protein homodimerization activity](http://amigo.geneontology.org/cgi-bin/amigo/go.cgi?view=details&query=GO:0042803)  [intracellular membrane-bounded organelle](http://amigo.geneontology.org/cgi-bin/amigo/go.cgi?view=details&query=GO:0043231)  [homocysteine catabolic process](http://amigo.geneontology.org/cgi-bin/amigo/go.cgi?view=details&query=GO:0043418)  [regulation of JUN kinase activity](http://amigo.geneontology.org/cgi-bin/amigo/go.cgi?view=details&query=GO:0043506)  [metal ion binding](http://amigo.geneontology.org/cgi-bin/amigo/go.cgi?view=details&query=GO:0046872)  [homocysteine metabolic process](http://amigo.geneontology.org/cgi-bin/amigo/go.cgi?view=details&query=GO:0050667)  [response to folic acid](http://amigo.geneontology.org/cgi-bin/amigo/go.cgi?view=details&query=GO:0051593)  [hydrogen sulfide biosynthetic process](http://amigo.geneontology.org/cgi-bin/amigo/go.cgi?view=details&query=GO:0070814) | [Glycine, serine and threonine metabolism](http://www.genome.ad.jp/dbget-bin/show_pathway?MAP00260+4.2.1.22)  [Cysteine and methionine metabolism](http://www.genome.ad.jp/dbget-bin/show_pathway?MAP00270+4.2.1.22)  [Metabolic pathways](http://www.genome.ad.jp/dbget-bin/show_pathway?MAP01100+4.2.1.22) |
| [1554007_at](https://www.affymetrix.com/LinkServlet?&probeset=1554007_at) |  |  |  |  | [BC036488](http://www.ncbi.nlm.nih.gov/entrez/query.fcgi?cmd=search&db=nucleotide&term=BC036488%5BACCN%5D&doptcmdl=GenBank) |  |  |  |  |  |  |
| [1554076_s_at](https://www.affymetrix.com/LinkServlet?&probeset=1554076_s_at) | TMEM136 | transmembrane protein 136 | 11 | 120196015 | [BC015232](http://www.ncbi.nlm.nih.gov/entrez/query.fcgi?cmd=search&db=nucleotide&term=BC015232%5BACCN%5D&doptcmdl=GenBank) | [219902](http://www.ncbi.nlm.nih.gov/sites/entrez?Db=gene&Cmd=DetailsSearch&Term=219902) | [11q23.3](http://www.ncbi.nlm.nih.gov/mapview/map_search.cgi?direct=on&idtype=gene&id=219902) | [Hs.643516](http://www.ncbi.nlm.nih.gov/UniGene/clust.cgi?ORG=Hs&CID=643516) | [2](http://www.ncbi.nih.gov/entrez/query.fcgi?tool=bioconductor&cmd=Retrieve&db=PubMed&list_uids=12477932%2C16344560) | [membrane](http://amigo.geneontology.org/cgi-bin/amigo/go.cgi?view=details&query=GO:0016020)  [integral to membrane](http://amigo.geneontology.org/cgi-bin/amigo/go.cgi?view=details&query=GO:0016021) |  |
| [1554579_a_at](https://www.affymetrix.com/LinkServlet?&probeset=1554579_a_at) | MYO18B | myosin XVIIIB | 22 | 26138119 | [AB042648](http://www.ncbi.nlm.nih.gov/entrez/query.fcgi?cmd=search&db=nucleotide&term=AB042648%5BACCN%5D&doptcmdl=GenBank) | [84700](http://www.ncbi.nlm.nih.gov/sites/entrez?Db=gene&Cmd=DetailsSearch&Term=84700) | [22q12.1](http://www.ncbi.nlm.nih.gov/mapview/map_search.cgi?direct=on&idtype=gene&id=84700) | [Hs.417959](http://www.ncbi.nlm.nih.gov/UniGene/clust.cgi?ORG=Hs&CID=417959) | [15](http://www.ncbi.nih.gov/entrez/query.fcgi?tool=bioconductor&cmd=Retrieve&db=PubMed&list_uids=10591208%2C12209013%2C12477932%2C12547197%2C12741677%2C14702039%2C15305387%2C16499872%2C17081983%2C17294804%2C17474147%2C18029348%2C18519826%2C20379614%2C20713499) | [nucleotide binding](http://amigo.geneontology.org/cgi-bin/amigo/go.cgi?view=details&query=GO:0000166)  [motor activity](http://amigo.geneontology.org/cgi-bin/amigo/go.cgi?view=details&query=GO:0003774)  [actin binding](http://amigo.geneontology.org/cgi-bin/amigo/go.cgi?view=details&query=GO:0003779)  [protein binding](http://amigo.geneontology.org/cgi-bin/amigo/go.cgi?view=details&query=GO:0005515)  [ATP binding](http://amigo.geneontology.org/cgi-bin/amigo/go.cgi?view=details&query=GO:0005524)  [nucleus](http://amigo.geneontology.org/cgi-bin/amigo/go.cgi?view=details&query=GO:0005634)  [cytoplasm](http://amigo.geneontology.org/cgi-bin/amigo/go.cgi?view=details&query=GO:0005737)  [unconventional myosin complex](http://amigo.geneontology.org/cgi-bin/amigo/go.cgi?view=details&query=GO:0016461)  [sarcomere](http://amigo.geneontology.org/cgi-bin/amigo/go.cgi?view=details&query=GO:0030017) |  |
| [1556236_at](https://www.affymetrix.com/LinkServlet?&probeset=1556236_at) |  |  |  |  | [BC035154](http://www.ncbi.nlm.nih.gov/entrez/query.fcgi?cmd=search&db=nucleotide&term=BC035154%5BACCN%5D&doptcmdl=GenBank) |  |  |  |  |  |  |
| [1558077_s_at](https://www.affymetrix.com/LinkServlet?&probeset=1558077_s_at) | MDH1B | malate dehydrogenase 1B, NAD (soluble) | 2 | -207602489 | [BG202523](http://www.ncbi.nlm.nih.gov/entrez/query.fcgi?cmd=search&db=nucleotide&term=BG202523%5BACCN%5D&doptcmdl=GenBank) | [130752](http://www.ncbi.nlm.nih.gov/sites/entrez?Db=gene&Cmd=DetailsSearch&Term=130752) | [2q33.3](http://www.ncbi.nlm.nih.gov/mapview/map_search.cgi?direct=on&idtype=gene&id=130752) | [Hs.147816](http://www.ncbi.nlm.nih.gov/UniGene/clust.cgi?ORG=Hs&CID=147816) | [2](http://www.ncbi.nih.gov/entrez/query.fcgi?tool=bioconductor&cmd=Retrieve&db=PubMed&list_uids=12477932%2C20379614) | [binding](http://amigo.geneontology.org/cgi-bin/amigo/go.cgi?view=details&query=GO:0005488)  [carbohydrate metabolic process](http://amigo.geneontology.org/cgi-bin/amigo/go.cgi?view=details&query=GO:0005975)  [tricarboxylic acid cycle](http://amigo.geneontology.org/cgi-bin/amigo/go.cgi?view=details&query=GO:0006099)  [malate metabolic process](http://amigo.geneontology.org/cgi-bin/amigo/go.cgi?view=details&query=GO:0006108)  [oxidoreductase activity](http://amigo.geneontology.org/cgi-bin/amigo/go.cgi?view=details&query=GO:0016491)  [malate dehydrogenase activity](http://amigo.geneontology.org/cgi-bin/amigo/go.cgi?view=details&query=GO:0016615)  [oxidation-reduction process](http://amigo.geneontology.org/cgi-bin/amigo/go.cgi?view=details&query=GO:0055114) |  |
| [1560316_s_at](https://www.affymetrix.com/LinkServlet?&probeset=1560316_s_at) | GLCCI1 | glucocorticoid induced transcript 1 | 7 | 8008422 | [N32168](http://www.ncbi.nlm.nih.gov/entrez/query.fcgi?cmd=search&db=nucleotide&term=N32168%5BACCN%5D&doptcmdl=GenBank) | [113263](http://www.ncbi.nlm.nih.gov/sites/entrez?Db=gene&Cmd=DetailsSearch&Term=113263) | [7p21.3](http://www.ncbi.nlm.nih.gov/mapview/map_search.cgi?direct=on&idtype=gene&id=113263) | [Hs.131673](http://www.ncbi.nlm.nih.gov/UniGene/clust.cgi?ORG=Hs&CID=131673) | [10](http://www.ncbi.nih.gov/entrez/query.fcgi?tool=bioconductor&cmd=Retrieve&db=PubMed&list_uids=11591653%2C12477932%2C12557054%2C12690205%2C12853948%2C14702039%2C15489334%2C16964243%2C17081983%2C18029348) | [cytoplasm](http://amigo.geneontology.org/cgi-bin/amigo/go.cgi?view=details&query=GO:0005737) |  |
| [1561528_at](https://www.affymetrix.com/LinkServlet?&probeset=1561528_at) |  |  |  |  | [BC032874](http://www.ncbi.nlm.nih.gov/entrez/query.fcgi?cmd=search&db=nucleotide&term=BC032874%5BACCN%5D&doptcmdl=GenBank) |  |  |  |  |  |  |
| [200831_s_at](https://www.affymetrix.com/LinkServlet?&probeset=200831_s_at) | SCD | stearoyl-CoA desaturase (delta-9-desaturase) | 10 | 102106771 | [AA678241](http://www.ncbi.nlm.nih.gov/entrez/query.fcgi?cmd=search&db=nucleotide&term=AA678241%5BACCN%5D&doptcmdl=GenBank) | [6319](http://www.ncbi.nlm.nih.gov/sites/entrez?Db=gene&Cmd=DetailsSearch&Term=6319) | [10q24.31](http://www.ncbi.nlm.nih.gov/mapview/map_search.cgi?direct=on&idtype=gene&id=6319) | [Hs.558396](http://www.ncbi.nlm.nih.gov/UniGene/clust.cgi?ORG=Hs&CID=558396) [Hs.597496](http://www.ncbi.nlm.nih.gov/UniGene/clust.cgi?ORG=Hs&CID=597496) | [55](http://www.ncbi.nih.gov/entrez/query.fcgi?tool=bioconductor&cmd=Retrieve&db=PubMed&list_uids=21148%2C6102994%2C7909540%2C8125298%2C9362069%2C9373149%2C10229681%2C10922050%2C11181995%2C11397803%2C11415448%2C11677241%2C12061775%2C12401889%2C12477932%2C14683458%2C14967817%2C14967823%2C15164054%2C15489334%2C15609334%2C15610069%2C15662557%2C15708362%2C15851470%2C15855323%2C15907797%2C16213227%2C16385451%2C16723740%2C17614770%2C17636091%2C17852835%2C18029348%2C18030445%2C18286258%2C18340007%2C18499418%2C18660489%2C18697866%2C18813799%2C18832746%2C18952834%2C19130493%2C19154947%2C19478146%2C19710915%2C19913121%2C20032470%2C20395685%2C20565855%2C20579763%2C20599700%2C20628086%2C21045174) | [stearoyl-CoA 9-desaturase activity](http://amigo.geneontology.org/cgi-bin/amigo/go.cgi?view=details&query=GO:0004768)  [iron ion binding](http://amigo.geneontology.org/cgi-bin/amigo/go.cgi?view=details&query=GO:0005506)  [endoplasmic reticulum](http://amigo.geneontology.org/cgi-bin/amigo/go.cgi?view=details&query=GO:0005783)  [endoplasmic reticulum membrane](http://amigo.geneontology.org/cgi-bin/amigo/go.cgi?view=details&query=GO:0005789)  [fatty acid biosynthetic process](http://amigo.geneontology.org/cgi-bin/amigo/go.cgi?view=details&query=GO:0006633)  [membrane](http://amigo.geneontology.org/cgi-bin/amigo/go.cgi?view=details&query=GO:0016020)  [integral to membrane](http://amigo.geneontology.org/cgi-bin/amigo/go.cgi?view=details&query=GO:0016021)  [oxidoreductase activity](http://amigo.geneontology.org/cgi-bin/amigo/go.cgi?view=details&query=GO:0016491)  [oxidoreductase activity, acting on paired donors, with oxidation of a pair of donors resulting in the reduction of molecular oxygen to two molecules of water](http://amigo.geneontology.org/cgi-bin/amigo/go.cgi?view=details&query=GO:0016717)  [oxidation-reduction process](http://amigo.geneontology.org/cgi-bin/amigo/go.cgi?view=details&query=GO:0055114) | [Biosynthesis of unsaturated fatty acids](http://www.genome.ad.jp/dbget-bin/show_pathway?MAP01040+1.14.19.1)  [PPAR signaling pathway](http://www.genome.ad.jp/dbget-bin/show_pathway?MAP03320+1.14.19.1) |
| [201012_at](https://www.affymetrix.com/LinkServlet?&probeset=201012_at) | ANXA1 | annexin A1 | 9 | 75766780 | [NM_000700](http://www.ncbi.nlm.nih.gov/entrez/query.fcgi?cmd=search&db=nucleotide&term=NM_000700%5BACCN%5D&doptcmdl=GenBank) | [301](http://www.ncbi.nlm.nih.gov/sites/entrez?Db=gene&Cmd=DetailsSearch&Term=301) | [9q12-q21.2](http://www.ncbi.nlm.nih.gov/mapview/map_search.cgi?direct=on&idtype=gene&id=301) [9q21.13](http://www.ncbi.nlm.nih.gov/mapview/map_search.cgi?direct=on&idtype=gene&id=301) | [Hs.494173](http://www.ncbi.nlm.nih.gov/UniGene/clust.cgi?ORG=Hs&CID=494173) | [124](http://www.ncbi.nih.gov/entrez/query.fcgi?tool=bioconductor&cmd=Retrieve&db=PubMed&list_uids=1374236%2C1602151%2C1670773%2C1832554%2C2138016%2C2457390%2C2532504%2C2936963%2C2967291%2C2969496%2C2971450%2C3303336%2C8425544%2C8453382%2C8557678%2C8587144%2C8955167%2C9459484%2C9915835%2C10512675%2C10882119%2C10908733%2C11468004%2C11574426%2C11759108%2C12165536%2C12236584%2C12477932%2C12645011%2C12679902%2C12859969%2C14506282%2C14633604%2C14733945%2C15064349%2C15157173%2C15168732%2C15187149%2C15447985%2C15476183%2C15485879%2C15489334%2C15526283%2C15581623%2C15592455%2C15883023%2C15944914%2C16014420%2C16100712%2C16130169%2C16226712%2C16324197%2C16460738%2C16530434%2C16627980%2C16675446%2C16741918%2C16883066%2C16899607%2C16949910%2C16973129%2C16984915%2C17008549%2C17019707%2C17023068%2C17081983%2C17215481%2C17317721%2C17340616%2C17353931%2C17372018%2C17626739%2C17681950%2C17873281%2C17932043%2C17971499%2C17994624%2C18029348%2C18164291%2C18297688%2C18396684%2C18566442%2C18577758%2C18594025%2C18652761%2C18663355%2C18673418%2C18706208%2C18776816%2C18794547%2C19076685%2C19101730%2C19104500%2C19204938%2C19208747%2C19289595%2C19351789%2C19394292%2C19428102%2C19596235%2C19615732%2C19767728%2C19850308%2C19913121%2C20007579%2C20025479%2C20061392%2C20163912%2C20237496%2C20308542%2C20353277%2C20359522%2C20398702%2C20549082%2C20558817%2C20562859%2C20628086%2C20665809%2C20679535%2C20713499%2C20821804%2C20872967%2C20962261%2C20970165) | [cornified envelope](http://amigo.geneontology.org/cgi-bin/amigo/go.cgi?view=details&query=GO:0001533)  [phospholipase inhibitor activity](http://amigo.geneontology.org/cgi-bin/amigo/go.cgi?view=details&query=GO:0004859)  [receptor binding](http://amigo.geneontology.org/cgi-bin/amigo/go.cgi?view=details&query=GO:0005102)  [structural molecule activity](http://amigo.geneontology.org/cgi-bin/amigo/go.cgi?view=details&query=GO:0005198)  [calcium ion binding](http://amigo.geneontology.org/cgi-bin/amigo/go.cgi?view=details&query=GO:0005509)  [protein binding](http://amigo.geneontology.org/cgi-bin/amigo/go.cgi?view=details&query=GO:0005515)  [phospholipid binding](http://amigo.geneontology.org/cgi-bin/amigo/go.cgi?view=details&query=GO:0005543)  [calcium-dependent phospholipid binding](http://amigo.geneontology.org/cgi-bin/amigo/go.cgi?view=details&query=GO:0005544)  [extracellular region](http://amigo.geneontology.org/cgi-bin/amigo/go.cgi?view=details&query=GO:0005576)  [extracellular region](http://amigo.geneontology.org/cgi-bin/amigo/go.cgi?view=details&query=GO:0005576)  [nucleus](http://amigo.geneontology.org/cgi-bin/amigo/go.cgi?view=details&query=GO:0005634)  [cytoplasm](http://amigo.geneontology.org/cgi-bin/amigo/go.cgi?view=details&query=GO:0005737)  [cilium](http://amigo.geneontology.org/cgi-bin/amigo/go.cgi?view=details&query=GO:0005929)  [lipid metabolic process](http://amigo.geneontology.org/cgi-bin/amigo/go.cgi?view=details&query=GO:0006629)  [anti-apoptosis](http://amigo.geneontology.org/cgi-bin/amigo/go.cgi?view=details&query=GO:0006916)  [cellular component movement](http://amigo.geneontology.org/cgi-bin/amigo/go.cgi?view=details&query=GO:0006928)  [inflammatory response](http://amigo.geneontology.org/cgi-bin/amigo/go.cgi?view=details&query=GO:0006954)  [cell cycle](http://amigo.geneontology.org/cgi-bin/amigo/go.cgi?view=details&query=GO:0007049)  [signal transduction](http://amigo.geneontology.org/cgi-bin/amigo/go.cgi?view=details&query=GO:0007165)  [cell surface receptor linked signaling pathway](http://amigo.geneontology.org/cgi-bin/amigo/go.cgi?view=details&query=GO:0007166)  [basolateral plasma membrane](http://amigo.geneontology.org/cgi-bin/amigo/go.cgi?view=details&query=GO:0016323)  [peptide cross-linking](http://amigo.geneontology.org/cgi-bin/amigo/go.cgi?view=details&query=GO:0018149)  [phospholipase A2 inhibitor activity](http://amigo.geneontology.org/cgi-bin/amigo/go.cgi?view=details&query=GO:0019834)  [keratinocyte differentiation](http://amigo.geneontology.org/cgi-bin/amigo/go.cgi?view=details&query=GO:0030216)  [protein binding, bridging](http://amigo.geneontology.org/cgi-bin/amigo/go.cgi?view=details&query=GO:0030674)  [positive regulation of vesicle fusion](http://amigo.geneontology.org/cgi-bin/amigo/go.cgi?view=details&query=GO:0031340)  [regulation of cell proliferation](http://amigo.geneontology.org/cgi-bin/amigo/go.cgi?view=details&query=GO:0042127)  [sarcolemma](http://amigo.geneontology.org/cgi-bin/amigo/go.cgi?view=details&query=GO:0042383)  [alpha-beta T cell differentiation](http://amigo.geneontology.org/cgi-bin/amigo/go.cgi?view=details&query=GO:0046632)  [arachidonic acid secretion](http://amigo.geneontology.org/cgi-bin/amigo/go.cgi?view=details&query=GO:0050482) |  |
| [201218_at](https://www.affymetrix.com/LinkServlet?&probeset=201218_at) | CTBP2 | C-terminal binding protein 2 | 10 | -126676418, -126676418, -126676418 | [N23018](http://www.ncbi.nlm.nih.gov/entrez/query.fcgi?cmd=search&db=nucleotide&term=N23018%5BACCN%5D&doptcmdl=GenBank) | [1488](http://www.ncbi.nlm.nih.gov/sites/entrez?Db=gene&Cmd=DetailsSearch&Term=1488) | [10q26.13](http://www.ncbi.nlm.nih.gov/mapview/map_search.cgi?direct=on&idtype=gene&id=1488) | [Hs.501345](http://www.ncbi.nlm.nih.gov/UniGene/clust.cgi?ORG=Hs&CID=501345) | [54](http://www.ncbi.nih.gov/entrez/query.fcgi?tool=bioconductor&cmd=Retrieve&db=PubMed&list_uids=7479821%2C9479502%2C9724649%2C9858600%2C10359772%2C10438528%2C10567582%2C10756197%2C10764811%2C11163272%2C11504872%2C11864595%2C12477932%2C12535528%2C12556451%2C12711682%2C12714599%2C12867035%2C15060175%2C15146197%2C15489334%2C15542832%2C16189514%2C16356938%2C16385451%2C16702210%2C16787403%2C17023432%2C17546044%2C18184656%2C18264096%2C18794092%2C19240061%2C19318432%2C19366831%2C19423541%2C19486893%2C19506021%2C19549807%2C19668232%2C19754958%2C19798104%2C19866473%2C19900942%2C19902474%2C20379614%2C20450899%2C20460480%2C20523059%2C20564319%2C20690139%2C20717903%2C20878950%2C21071540) | [protein binding](http://amigo.geneontology.org/cgi-bin/amigo/go.cgi?view=details&query=GO:0005515)  [nucleus](http://amigo.geneontology.org/cgi-bin/amigo/go.cgi?view=details&query=GO:0005634)  [negative regulation of cell proliferation](http://amigo.geneontology.org/cgi-bin/amigo/go.cgi?view=details&query=GO:0008285)  [negative regulation of transcription](http://amigo.geneontology.org/cgi-bin/amigo/go.cgi?view=details&query=GO:0016481)  [oxidoreductase activity](http://amigo.geneontology.org/cgi-bin/amigo/go.cgi?view=details&query=GO:0016491)  [transcription repressor activity](http://amigo.geneontology.org/cgi-bin/amigo/go.cgi?view=details&query=GO:0016564)  [oxidoreductase activity, acting on the CH-OH group of donors, NAD or NADP as acceptor](http://amigo.geneontology.org/cgi-bin/amigo/go.cgi?view=details&query=GO:0016616)  [transcriptional repressor complex](http://amigo.geneontology.org/cgi-bin/amigo/go.cgi?view=details&query=GO:0017053)  [viral genome replication](http://amigo.geneontology.org/cgi-bin/amigo/go.cgi?view=details&query=GO:0019079)  [cell junction](http://amigo.geneontology.org/cgi-bin/amigo/go.cgi?view=details&query=GO:0030054)  [cell differentiation](http://amigo.geneontology.org/cgi-bin/amigo/go.cgi?view=details&query=GO:0030154)  [synapse](http://amigo.geneontology.org/cgi-bin/amigo/go.cgi?view=details&query=GO:0045202)  [cofactor binding](http://amigo.geneontology.org/cgi-bin/amigo/go.cgi?view=details&query=GO:0048037)  [white fat cell differentiation](http://amigo.geneontology.org/cgi-bin/amigo/go.cgi?view=details&query=GO:0050872)  [NAD binding](http://amigo.geneontology.org/cgi-bin/amigo/go.cgi?view=details&query=GO:0051287)  [oxidation-reduction process](http://amigo.geneontology.org/cgi-bin/amigo/go.cgi?view=details&query=GO:0055114) | [Wnt signaling pathway](http://www.genome.ad.jp/kegg/pathway/hsa/hsa04310.html)  [Notch signaling pathway](http://www.genome.ad.jp/kegg/pathway/hsa/hsa04330.html)  [Pathways in cancer](http://www.genome.ad.jp/kegg/pathway/hsa/hsa05200.html)  [Chronic myeloid leukemia](http://www.genome.ad.jp/kegg/pathway/hsa/hsa05220.html) |
| [201219_at](https://www.affymetrix.com/LinkServlet?&probeset=201219_at) |  |  |  |  | [AW269836](http://www.ncbi.nlm.nih.gov/entrez/query.fcgi?cmd=search&db=nucleotide&term=AW269836%5BACCN%5D&doptcmdl=GenBank) |  |  |  |  | [ubiquitin thiolesterase activity](http://amigo.geneontology.org/cgi-bin/amigo/go.cgi?view=details&query=GO:0004221)  [protein binding](http://amigo.geneontology.org/cgi-bin/amigo/go.cgi?view=details&query=GO:0005515)  [intracellular](http://amigo.geneontology.org/cgi-bin/amigo/go.cgi?view=details&query=GO:0005622)  [nucleus](http://amigo.geneontology.org/cgi-bin/amigo/go.cgi?view=details&query=GO:0005634)  [nucleus](http://amigo.geneontology.org/cgi-bin/amigo/go.cgi?view=details&query=GO:0005634)  [nucleolus](http://amigo.geneontology.org/cgi-bin/amigo/go.cgi?view=details&query=GO:0005730)  [cytoplasm](http://amigo.geneontology.org/cgi-bin/amigo/go.cgi?view=details&query=GO:0005737)  [centrosome](http://amigo.geneontology.org/cgi-bin/amigo/go.cgi?view=details&query=GO:0005813)  [peptidase activity](http://amigo.geneontology.org/cgi-bin/amigo/go.cgi?view=details&query=GO:0008233)  [cysteine-type peptidase activity](http://amigo.geneontology.org/cgi-bin/amigo/go.cgi?view=details&query=GO:0008234)  [zinc ion binding](http://amigo.geneontology.org/cgi-bin/amigo/go.cgi?view=details&query=GO:0008270)  [negative regulation of cell proliferation](http://amigo.geneontology.org/cgi-bin/amigo/go.cgi?view=details&query=GO:0008285)  [aggresome](http://amigo.geneontology.org/cgi-bin/amigo/go.cgi?view=details&query=GO:0016235)  [negative regulation of transcription](http://amigo.geneontology.org/cgi-bin/amigo/go.cgi?view=details&query=GO:0016481)  [oxidoreductase activity](http://amigo.geneontology.org/cgi-bin/amigo/go.cgi?view=details&query=GO:0016491)  [transcription repressor activity](http://amigo.geneontology.org/cgi-bin/amigo/go.cgi?view=details&query=GO:0016564)  [oxidoreductase activity, acting on the CH-OH group of donors, NAD or NADP as acceptor](http://amigo.geneontology.org/cgi-bin/amigo/go.cgi?view=details&query=GO:0016616)  [transcriptional repressor complex](http://amigo.geneontology.org/cgi-bin/amigo/go.cgi?view=details&query=GO:0017053)  [viral genome replication](http://amigo.geneontology.org/cgi-bin/amigo/go.cgi?view=details&query=GO:0019079)  [cell junction](http://amigo.geneontology.org/cgi-bin/amigo/go.cgi?view=details&query=GO:0030054)  [cell differentiation](http://amigo.geneontology.org/cgi-bin/amigo/go.cgi?view=details&query=GO:0030154)  [positive regulation of Wnt receptor signaling pathway](http://amigo.geneontology.org/cgi-bin/amigo/go.cgi?view=details&query=GO:0030177)  [intermediate filament cytoskeleton](http://amigo.geneontology.org/cgi-bin/amigo/go.cgi?view=details&query=GO:0045111)  [synapse](http://amigo.geneontology.org/cgi-bin/amigo/go.cgi?view=details&query=GO:0045202)  [metal ion binding](http://amigo.geneontology.org/cgi-bin/amigo/go.cgi?view=details&query=GO:0046872)  [cofactor binding](http://amigo.geneontology.org/cgi-bin/amigo/go.cgi?view=details&query=GO:0048037)  [white fat cell differentiation](http://amigo.geneontology.org/cgi-bin/amigo/go.cgi?view=details&query=GO:0050872)  [NAD binding](http://amigo.geneontology.org/cgi-bin/amigo/go.cgi?view=details&query=GO:0051287)  [oxidation-reduction process](http://amigo.geneontology.org/cgi-bin/amigo/go.cgi?view=details&query=GO:0055114)  [protein K63-linked deubiquitination](http://amigo.geneontology.org/cgi-bin/amigo/go.cgi?view=details&query=GO:0070536) |  |
| [201220_x_at](https://www.affymetrix.com/LinkServlet?&probeset=201220_x_at) | CTBP2 | C-terminal binding protein 2 | 10 | -126676418, -126676418, -126676418 | [NM_001329](http://www.ncbi.nlm.nih.gov/entrez/query.fcgi?cmd=search&db=nucleotide&term=NM_001329%5BACCN%5D&doptcmdl=GenBank) | [1488](http://www.ncbi.nlm.nih.gov/sites/entrez?Db=gene&Cmd=DetailsSearch&Term=1488) | [10q26.13](http://www.ncbi.nlm.nih.gov/mapview/map_search.cgi?direct=on&idtype=gene&id=1488) | [Hs.501345](http://www.ncbi.nlm.nih.gov/UniGene/clust.cgi?ORG=Hs&CID=501345) | [54](http://www.ncbi.nih.gov/entrez/query.fcgi?tool=bioconductor&cmd=Retrieve&db=PubMed&list_uids=7479821%2C9479502%2C9724649%2C9858600%2C10359772%2C10438528%2C10567582%2C10756197%2C10764811%2C11163272%2C11504872%2C11864595%2C12477932%2C12535528%2C12556451%2C12711682%2C12714599%2C12867035%2C15060175%2C15146197%2C15489334%2C15542832%2C16189514%2C16356938%2C16385451%2C16702210%2C16787403%2C17023432%2C17546044%2C18184656%2C18264096%2C18794092%2C19240061%2C19318432%2C19366831%2C19423541%2C19486893%2C19506021%2C19549807%2C19668232%2C19754958%2C19798104%2C19866473%2C19900942%2C19902474%2C20379614%2C20450899%2C20460480%2C20523059%2C20564319%2C20690139%2C20717903%2C20878950%2C21071540) | [protein binding](http://amigo.geneontology.org/cgi-bin/amigo/go.cgi?view=details&query=GO:0005515)  [nucleus](http://amigo.geneontology.org/cgi-bin/amigo/go.cgi?view=details&query=GO:0005634)  [negative regulation of cell proliferation](http://amigo.geneontology.org/cgi-bin/amigo/go.cgi?view=details&query=GO:0008285)  [negative regulation of transcription](http://amigo.geneontology.org/cgi-bin/amigo/go.cgi?view=details&query=GO:0016481)  [oxidoreductase activity](http://amigo.geneontology.org/cgi-bin/amigo/go.cgi?view=details&query=GO:0016491)  [transcription repressor activity](http://amigo.geneontology.org/cgi-bin/amigo/go.cgi?view=details&query=GO:0016564)  [oxidoreductase activity, acting on the CH-OH group of donors, NAD or NADP as acceptor](http://amigo.geneontology.org/cgi-bin/amigo/go.cgi?view=details&query=GO:0016616)  [transcriptional repressor complex](http://amigo.geneontology.org/cgi-bin/amigo/go.cgi?view=details&query=GO:0017053)  [viral genome replication](http://amigo.geneontology.org/cgi-bin/amigo/go.cgi?view=details&query=GO:0019079)  [cell junction](http://amigo.geneontology.org/cgi-bin/amigo/go.cgi?view=details&query=GO:0030054)  [cell differentiation](http://amigo.geneontology.org/cgi-bin/amigo/go.cgi?view=details&query=GO:0030154)  [synapse](http://amigo.geneontology.org/cgi-bin/amigo/go.cgi?view=details&query=GO:0045202)  [cofactor binding](http://amigo.geneontology.org/cgi-bin/amigo/go.cgi?view=details&query=GO:0048037)  [white fat cell differentiation](http://amigo.geneontology.org/cgi-bin/amigo/go.cgi?view=details&query=GO:0050872)  [NAD binding](http://amigo.geneontology.org/cgi-bin/amigo/go.cgi?view=details&query=GO:0051287)  [oxidation-reduction process](http://amigo.geneontology.org/cgi-bin/amigo/go.cgi?view=details&query=GO:0055114) | [Wnt signaling pathway](http://www.genome.ad.jp/kegg/pathway/hsa/hsa04310.html)  [Notch signaling pathway](http://www.genome.ad.jp/kegg/pathway/hsa/hsa04330.html)  [Pathways in cancer](http://www.genome.ad.jp/kegg/pathway/hsa/hsa05200.html)  [Chronic myeloid leukemia](http://www.genome.ad.jp/kegg/pathway/hsa/hsa05220.html) |
| [201848_s_at](https://www.affymetrix.com/LinkServlet?&probeset=201848_s_at) | BNIP3 | BCL2/adenovirus E1B 19kDa interacting protein 3 | 10 | -133781203 | [U15174](http://www.ncbi.nlm.nih.gov/entrez/query.fcgi?cmd=search&db=nucleotide&term=U15174%5BACCN%5D&doptcmdl=GenBank) | [664](http://www.ncbi.nlm.nih.gov/sites/entrez?Db=gene&Cmd=DetailsSearch&Term=664) | [10q26.3](http://www.ncbi.nlm.nih.gov/mapview/map_search.cgi?direct=on&idtype=gene&id=664) | [Hs.144873](http://www.ncbi.nlm.nih.gov/UniGene/clust.cgi?ORG=Hs&CID=144873) | [71](http://www.ncbi.nih.gov/entrez/query.fcgi?tool=bioconductor&cmd=Retrieve&db=PubMed&list_uids=7478990%2C7954800%2C8125298%2C9373149%2C9396766%2C9575197%2C9867803%2C9973195%2C10381623%2C10625696%2C10891486%2C12215374%2C12477932%2C12690108%2C12879018%2C15164054%2C15289340%2C15328198%2C15489334%2C15560738%2C15709167%2C15856026%2C16002567%2C16169070%2C16189514%2C16217754%2C16219518%2C16357180%2C16765911%2C16799636%2C16803523%2C17207965%2C17255267%2C17360363%2C17394490%2C17576382%2C17576813%2C17600828%2C17638546%2C17638890%2C17729412%2C17786027%2C17928295%2C18029348%2C18059169%2C18092960%2C18163427%2C18359286%2C18371312%2C18551130%2C18728663%2C18838203%2C19088195%2C19273585%2C19339613%2C19505343%2C19535684%2C19641497%2C19668230%2C19690192%2C19956881%2C20026130%2C20082478%2C20100468%2C20201926%2C20368736%2C20436456%2C20437871%2C20637734%2C20855536%2C20973794) | [response to hypoxia](http://amigo.geneontology.org/cgi-bin/amigo/go.cgi?view=details&query=GO:0001666)  [protein binding](http://amigo.geneontology.org/cgi-bin/amigo/go.cgi?view=details&query=GO:0005515)  [nucleus](http://amigo.geneontology.org/cgi-bin/amigo/go.cgi?view=details&query=GO:0005634)  [nucleus](http://amigo.geneontology.org/cgi-bin/amigo/go.cgi?view=details&query=GO:0005634)  [nuclear envelope](http://amigo.geneontology.org/cgi-bin/amigo/go.cgi?view=details&query=GO:0005635)  [nucleoplasm](http://amigo.geneontology.org/cgi-bin/amigo/go.cgi?view=details&query=GO:0005654)  [cytoplasm](http://amigo.geneontology.org/cgi-bin/amigo/go.cgi?view=details&query=GO:0005737)  [mitochondrion](http://amigo.geneontology.org/cgi-bin/amigo/go.cgi?view=details&query=GO:0005739)  [mitochondrion](http://amigo.geneontology.org/cgi-bin/amigo/go.cgi?view=details&query=GO:0005739)  [DNA fragmentation involved in apoptotic nuclear change](http://amigo.geneontology.org/cgi-bin/amigo/go.cgi?view=details&query=GO:0006309)  [chromatin remodeling](http://amigo.geneontology.org/cgi-bin/amigo/go.cgi?view=details&query=GO:0006338)  [apoptosis](http://amigo.geneontology.org/cgi-bin/amigo/go.cgi?view=details&query=GO:0006915)  [apoptosis](http://amigo.geneontology.org/cgi-bin/amigo/go.cgi?view=details&query=GO:0006915)  [anti-apoptosis](http://amigo.geneontology.org/cgi-bin/amigo/go.cgi?view=details&query=GO:0006916)  [induction of apoptosis](http://amigo.geneontology.org/cgi-bin/amigo/go.cgi?view=details&query=GO:0006917)  [cell death](http://amigo.geneontology.org/cgi-bin/amigo/go.cgi?view=details&query=GO:0008219)  [cell death](http://amigo.geneontology.org/cgi-bin/amigo/go.cgi?view=details&query=GO:0008219)  [negative regulation of survival gene product expression](http://amigo.geneontology.org/cgi-bin/amigo/go.cgi?view=details&query=GO:0008634)  [membrane](http://amigo.geneontology.org/cgi-bin/amigo/go.cgi?view=details&query=GO:0016020)  [integral to membrane](http://amigo.geneontology.org/cgi-bin/amigo/go.cgi?view=details&query=GO:0016021)  [dendrite](http://amigo.geneontology.org/cgi-bin/amigo/go.cgi?view=details&query=GO:0030425)  [integral to mitochondrial outer membrane](http://amigo.geneontology.org/cgi-bin/amigo/go.cgi?view=details&query=GO:0031307)  [mitochondrial membrane](http://amigo.geneontology.org/cgi-bin/amigo/go.cgi?view=details&query=GO:0031966)  [mitochondrial membrane](http://amigo.geneontology.org/cgi-bin/amigo/go.cgi?view=details&query=GO:0031966)  [identical protein binding](http://amigo.geneontology.org/cgi-bin/amigo/go.cgi?view=details&query=GO:0042802)  [protein homodimerization activity](http://amigo.geneontology.org/cgi-bin/amigo/go.cgi?view=details&query=GO:0042803)  [interspecies interaction between organisms](http://amigo.geneontology.org/cgi-bin/amigo/go.cgi?view=details&query=GO:0044419)  [negative regulation of membrane potential](http://amigo.geneontology.org/cgi-bin/amigo/go.cgi?view=details&query=GO:0045837)  [regulation of mitochondrial membrane permeability](http://amigo.geneontology.org/cgi-bin/amigo/go.cgi?view=details&query=GO:0046902)  [protein heterodimerization activity](http://amigo.geneontology.org/cgi-bin/amigo/go.cgi?view=details&query=GO:0046982)  [neuron apoptosis](http://amigo.geneontology.org/cgi-bin/amigo/go.cgi?view=details&query=GO:0051402)  [defense response to virus](http://amigo.geneontology.org/cgi-bin/amigo/go.cgi?view=details&query=GO:0051607) |  |
| [201849_at](https://www.affymetrix.com/LinkServlet?&probeset=201849_at) | BNIP3 | BCL2/adenovirus E1B 19kDa interacting protein 3 | 10 | -133781203 | [NM_004052](http://www.ncbi.nlm.nih.gov/entrez/query.fcgi?cmd=search&db=nucleotide&term=NM_004052%5BACCN%5D&doptcmdl=GenBank) | [664](http://www.ncbi.nlm.nih.gov/sites/entrez?Db=gene&Cmd=DetailsSearch&Term=664) | [10q26.3](http://www.ncbi.nlm.nih.gov/mapview/map_search.cgi?direct=on&idtype=gene&id=664) | [Hs.144873](http://www.ncbi.nlm.nih.gov/UniGene/clust.cgi?ORG=Hs&CID=144873) | [71](http://www.ncbi.nih.gov/entrez/query.fcgi?tool=bioconductor&cmd=Retrieve&db=PubMed&list_uids=7478990%2C7954800%2C8125298%2C9373149%2C9396766%2C9575197%2C9867803%2C9973195%2C10381623%2C10625696%2C10891486%2C12215374%2C12477932%2C12690108%2C12879018%2C15164054%2C15289340%2C15328198%2C15489334%2C15560738%2C15709167%2C15856026%2C16002567%2C16169070%2C16189514%2C16217754%2C16219518%2C16357180%2C16765911%2C16799636%2C16803523%2C17207965%2C17255267%2C17360363%2C17394490%2C17576382%2C17576813%2C17600828%2C17638546%2C17638890%2C17729412%2C17786027%2C17928295%2C18029348%2C18059169%2C18092960%2C18163427%2C18359286%2C18371312%2C18551130%2C18728663%2C18838203%2C19088195%2C19273585%2C19339613%2C19505343%2C19535684%2C19641497%2C19668230%2C19690192%2C19956881%2C20026130%2C20082478%2C20100468%2C20201926%2C20368736%2C20436456%2C20437871%2C20637734%2C20855536%2C20973794) | [response to hypoxia](http://amigo.geneontology.org/cgi-bin/amigo/go.cgi?view=details&query=GO:0001666)  [protein binding](http://amigo.geneontology.org/cgi-bin/amigo/go.cgi?view=details&query=GO:0005515)  [nucleus](http://amigo.geneontology.org/cgi-bin/amigo/go.cgi?view=details&query=GO:0005634)  [nucleus](http://amigo.geneontology.org/cgi-bin/amigo/go.cgi?view=details&query=GO:0005634)  [nuclear envelope](http://amigo.geneontology.org/cgi-bin/amigo/go.cgi?view=details&query=GO:0005635)  [nucleoplasm](http://amigo.geneontology.org/cgi-bin/amigo/go.cgi?view=details&query=GO:0005654)  [cytoplasm](http://amigo.geneontology.org/cgi-bin/amigo/go.cgi?view=details&query=GO:0005737)  [mitochondrion](http://amigo.geneontology.org/cgi-bin/amigo/go.cgi?view=details&query=GO:0005739)  [mitochondrion](http://amigo.geneontology.org/cgi-bin/amigo/go.cgi?view=details&query=GO:0005739)  [DNA fragmentation involved in apoptotic nuclear change](http://amigo.geneontology.org/cgi-bin/amigo/go.cgi?view=details&query=GO:0006309)  [chromatin remodeling](http://amigo.geneontology.org/cgi-bin/amigo/go.cgi?view=details&query=GO:0006338)  [apoptosis](http://amigo.geneontology.org/cgi-bin/amigo/go.cgi?view=details&query=GO:0006915)  [apoptosis](http://amigo.geneontology.org/cgi-bin/amigo/go.cgi?view=details&query=GO:0006915)  [anti-apoptosis](http://amigo.geneontology.org/cgi-bin/amigo/go.cgi?view=details&query=GO:0006916)  [induction of apoptosis](http://amigo.geneontology.org/cgi-bin/amigo/go.cgi?view=details&query=GO:0006917)  [cell death](http://amigo.geneontology.org/cgi-bin/amigo/go.cgi?view=details&query=GO:0008219)  [cell death](http://amigo.geneontology.org/cgi-bin/amigo/go.cgi?view=details&query=GO:0008219)  [negative regulation of survival gene product expression](http://amigo.geneontology.org/cgi-bin/amigo/go.cgi?view=details&query=GO:0008634)  [membrane](http://amigo.geneontology.org/cgi-bin/amigo/go.cgi?view=details&query=GO:0016020)  [integral to membrane](http://amigo.geneontology.org/cgi-bin/amigo/go.cgi?view=details&query=GO:0016021)  [dendrite](http://amigo.geneontology.org/cgi-bin/amigo/go.cgi?view=details&query=GO:0030425)  [integral to mitochondrial outer membrane](http://amigo.geneontology.org/cgi-bin/amigo/go.cgi?view=details&query=GO:0031307)  [mitochondrial membrane](http://amigo.geneontology.org/cgi-bin/amigo/go.cgi?view=details&query=GO:0031966)  [mitochondrial membrane](http://amigo.geneontology.org/cgi-bin/amigo/go.cgi?view=details&query=GO:0031966)  [identical protein binding](http://amigo.geneontology.org/cgi-bin/amigo/go.cgi?view=details&query=GO:0042802)  [protein homodimerization activity](http://amigo.geneontology.org/cgi-bin/amigo/go.cgi?view=details&query=GO:0042803)  [interspecies interaction between organisms](http://amigo.geneontology.org/cgi-bin/amigo/go.cgi?view=details&query=GO:0044419)  [negative regulation of membrane potential](http://amigo.geneontology.org/cgi-bin/amigo/go.cgi?view=details&query=GO:0045837)  [regulation of mitochondrial membrane permeability](http://amigo.geneontology.org/cgi-bin/amigo/go.cgi?view=details&query=GO:0046902)  [protein heterodimerization activity](http://amigo.geneontology.org/cgi-bin/amigo/go.cgi?view=details&query=GO:0046982)  [neuron apoptosis](http://amigo.geneontology.org/cgi-bin/amigo/go.cgi?view=details&query=GO:0051402)  [defense response to virus](http://amigo.geneontology.org/cgi-bin/amigo/go.cgi?view=details&query=GO:0051607) |  |
| [202201_at](https://www.affymetrix.com/LinkServlet?&probeset=202201_at) | BLVRB | biliverdin reductase B (flavin reductase (NADPH)) | 19 | -40953690 | [NM_000713](http://www.ncbi.nlm.nih.gov/entrez/query.fcgi?cmd=search&db=nucleotide&term=NM_000713%5BACCN%5D&doptcmdl=GenBank) | [645](http://www.ncbi.nlm.nih.gov/sites/entrez?Db=gene&Cmd=DetailsSearch&Term=645) | [19q13.1-q13.2](http://www.ncbi.nlm.nih.gov/mapview/map_search.cgi?direct=on&idtype=gene&id=645) | [Hs.515785](http://www.ncbi.nlm.nih.gov/UniGene/clust.cgi?ORG=Hs&CID=515785) | [15](http://www.ncbi.nih.gov/entrez/query.fcgi?tool=bioconductor&cmd=Retrieve&db=PubMed&list_uids=1286669%2C7656592%2C7929092%2C8117274%2C8280170%2C8313871%2C8687377%2C8799475%2C11224564%2C12477932%2C12626517%2C12909459%2C15489334%2C18241201%2C19027726) | [biliverdin reductase activity](http://amigo.geneontology.org/cgi-bin/amigo/go.cgi?view=details&query=GO:0004074)  [biliverdin reductase activity](http://amigo.geneontology.org/cgi-bin/amigo/go.cgi?view=details&query=GO:0004074)  [binding](http://amigo.geneontology.org/cgi-bin/amigo/go.cgi?view=details&query=GO:0005488)  [cytoplasm](http://amigo.geneontology.org/cgi-bin/amigo/go.cgi?view=details&query=GO:0005737)  [cytosol](http://amigo.geneontology.org/cgi-bin/amigo/go.cgi?view=details&query=GO:0005829)  [cytosol](http://amigo.geneontology.org/cgi-bin/amigo/go.cgi?view=details&query=GO:0005829)  [porphyrin metabolic process](http://amigo.geneontology.org/cgi-bin/amigo/go.cgi?view=details&query=GO:0006778)  [oxidoreductase activity](http://amigo.geneontology.org/cgi-bin/amigo/go.cgi?view=details&query=GO:0016491)  [heme catabolic process](http://amigo.geneontology.org/cgi-bin/amigo/go.cgi?view=details&query=GO:0042167)  [heme catabolic process](http://amigo.geneontology.org/cgi-bin/amigo/go.cgi?view=details&query=GO:0042167)  [flavin reductase activity](http://amigo.geneontology.org/cgi-bin/amigo/go.cgi?view=details&query=GO:0042602)  [oxidation-reduction process](http://amigo.geneontology.org/cgi-bin/amigo/go.cgi?view=details&query=GO:0055114) | [Porphyrin and chlorophyll metabolism](http://www.genome.ad.jp/dbget-bin/show_pathway?MAP00860+1.3.1.24) |
| [202825_at](https://www.affymetrix.com/LinkServlet?&probeset=202825_at) | SLC25A4 | solute carrier family 25 (mitochondrial carrier; adenine nucleotide translocator), member 4 | 4 | 186064416 | [NM_001151](http://www.ncbi.nlm.nih.gov/entrez/query.fcgi?cmd=search&db=nucleotide&term=NM_001151%5BACCN%5D&doptcmdl=GenBank) | [291](http://www.ncbi.nlm.nih.gov/sites/entrez?Db=gene&Cmd=DetailsSearch&Term=291) | [4q35](http://www.ncbi.nlm.nih.gov/mapview/map_search.cgi?direct=on&idtype=gene&id=291) | [Hs.246506](http://www.ncbi.nlm.nih.gov/UniGene/clust.cgi?ORG=Hs&CID=246506) | [71](http://www.ncbi.nih.gov/entrez/query.fcgi?tool=bioconductor&cmd=Retrieve&db=PubMed&list_uids=1582253%2C2541251%2C2547778%2C2823266%2C2829183%2C8103757%2C8479824%2C8619474%2C8644740%2C9110174%2C9748162%2C9874241%2C10364542%2C10620603%2C10926541%2C11175251%2C11181702%2C11193032%2C11287411%2C11756592%2C11756613%2C11809823%2C12039962%2C12112115%2C12140186%2C12149099%2C12450408%2C12477932%2C12565915%2C12663490%2C12707443%2C12750393%2C12750404%2C14729611%2C15033717%2C15142377%2C15231833%2C15489334%2C15551024%2C15638722%2C15725353%2C15792871%2C15817944%2C15832179%2C16020522%2C16107323%2C16120388%2C16155110%2C16226712%2C16354571%2C16429131%2C16492162%2C16507998%2C16511342%2C16556444%2C16887100%2C17420318%2C18504126%2C18575922%2C18852887%2C19232058%2C19425506%2C19965780%2C20007455%2C20160640%2C20181062%2C20504995%2C20528917%2C20698827%2C20843780%2C20877624) | [mitochondrial genome maintenance](http://amigo.geneontology.org/cgi-bin/amigo/go.cgi?view=details&query=GO:0000002)  [transporter activity](http://amigo.geneontology.org/cgi-bin/amigo/go.cgi?view=details&query=GO:0005215)  [ATP:ADP antiporter activity](http://amigo.geneontology.org/cgi-bin/amigo/go.cgi?view=details&query=GO:0005471)  [protein binding](http://amigo.geneontology.org/cgi-bin/amigo/go.cgi?view=details&query=GO:0005515)  [mitochondrion](http://amigo.geneontology.org/cgi-bin/amigo/go.cgi?view=details&query=GO:0005739)  [mitochondrial outer membrane](http://amigo.geneontology.org/cgi-bin/amigo/go.cgi?view=details&query=GO:0005741)  [mitochondrial inner membrane](http://amigo.geneontology.org/cgi-bin/amigo/go.cgi?view=details&query=GO:0005743)  [mitochondrial inner membrane](http://amigo.geneontology.org/cgi-bin/amigo/go.cgi?view=details&query=GO:0005743)  [integral to plasma membrane](http://amigo.geneontology.org/cgi-bin/amigo/go.cgi?view=details&query=GO:0005887)  [generation of precursor metabolites and energy](http://amigo.geneontology.org/cgi-bin/amigo/go.cgi?view=details&query=GO:0006091)  [energy reserve metabolic process](http://amigo.geneontology.org/cgi-bin/amigo/go.cgi?view=details&query=GO:0006112)  [transport](http://amigo.geneontology.org/cgi-bin/amigo/go.cgi?view=details&query=GO:0006810)  [adenine transmembrane transporter activity](http://amigo.geneontology.org/cgi-bin/amigo/go.cgi?view=details&query=GO:0015207)  [adenine transport](http://amigo.geneontology.org/cgi-bin/amigo/go.cgi?view=details&query=GO:0015853)  [ADP transport](http://amigo.geneontology.org/cgi-bin/amigo/go.cgi?view=details&query=GO:0015866)  [ATP transport](http://amigo.geneontology.org/cgi-bin/amigo/go.cgi?view=details&query=GO:0015867)  [membrane](http://amigo.geneontology.org/cgi-bin/amigo/go.cgi?view=details&query=GO:0016020)  [viral reproduction](http://amigo.geneontology.org/cgi-bin/amigo/go.cgi?view=details&query=GO:0016032)  [interspecies interaction between organisms](http://amigo.geneontology.org/cgi-bin/amigo/go.cgi?view=details&query=GO:0044419)  [regulation of insulin secretion](http://amigo.geneontology.org/cgi-bin/amigo/go.cgi?view=details&query=GO:0050796)  [glutamate uptake involved in synaptic transmission](http://amigo.geneontology.org/cgi-bin/amigo/go.cgi?view=details&query=GO:0051935)  [transmembrane transport](http://amigo.geneontology.org/cgi-bin/amigo/go.cgi?view=details&query=GO:0055085)  [negative regulation of necrotic cell death](http://amigo.geneontology.org/cgi-bin/amigo/go.cgi?view=details&query=GO:0060547) | [Calcium signaling pathway](http://www.genome.ad.jp/kegg/pathway/hsa/hsa04020.html)  [Parkinson's disease](http://www.genome.ad.jp/kegg/pathway/hsa/hsa05012.html)  [Huntington's disease](http://www.genome.ad.jp/kegg/pathway/hsa/hsa05016.html) |
| [202893_at](https://www.affymetrix.com/LinkServlet?&probeset=202893_at) | UNC13B | unc-13 homolog B (C. elegans) | 9 | 35161988 | [NM_006377](http://www.ncbi.nlm.nih.gov/entrez/query.fcgi?cmd=search&db=nucleotide&term=NM_006377%5BACCN%5D&doptcmdl=GenBank) | [10497](http://www.ncbi.nlm.nih.gov/sites/entrez?Db=gene&Cmd=DetailsSearch&Term=10497) | [9p13.3](http://www.ncbi.nlm.nih.gov/mapview/map_search.cgi?direct=on&idtype=gene&id=10497) | [Hs.493791](http://www.ncbi.nlm.nih.gov/UniGene/clust.cgi?ORG=Hs&CID=493791) | [22](http://www.ncbi.nih.gov/entrez/query.fcgi?tool=bioconductor&cmd=Retrieve&db=PubMed&list_uids=8999968%2C9195900%2C9607201%2C9704016%2C9736751%2C10233166%2C10488064%2C11343654%2C11797009%2C12163476%2C12477932%2C12871971%2C14593078%2C15342556%2C15466010%2C16138900%2C16169070%2C18633107%2C19492809%2C19615732%2C19641095%2C20379614) | [signal transducer activity](http://amigo.geneontology.org/cgi-bin/amigo/go.cgi?view=details&query=GO:0004871)  [receptor activity](http://amigo.geneontology.org/cgi-bin/amigo/go.cgi?view=details&query=GO:0004872)  [cytoplasm](http://amigo.geneontology.org/cgi-bin/amigo/go.cgi?view=details&query=GO:0005737)  [Golgi apparatus](http://amigo.geneontology.org/cgi-bin/amigo/go.cgi?view=details&query=GO:0005794)  [plasma membrane](http://amigo.geneontology.org/cgi-bin/amigo/go.cgi?view=details&query=GO:0005886)  [exocytosis](http://amigo.geneontology.org/cgi-bin/amigo/go.cgi?view=details&query=GO:0006887)  [induction of apoptosis](http://amigo.geneontology.org/cgi-bin/amigo/go.cgi?view=details&query=GO:0006917)  [signal transduction](http://amigo.geneontology.org/cgi-bin/amigo/go.cgi?view=details&query=GO:0007165)  [excretion](http://amigo.geneontology.org/cgi-bin/amigo/go.cgi?view=details&query=GO:0007588)  [cell junction](http://amigo.geneontology.org/cgi-bin/amigo/go.cgi?view=details&query=GO:0030054)  [intracellular signal transduction](http://amigo.geneontology.org/cgi-bin/amigo/go.cgi?view=details&query=GO:0035556)  [synapse](http://amigo.geneontology.org/cgi-bin/amigo/go.cgi?view=details&query=GO:0045202)  [metal ion binding](http://amigo.geneontology.org/cgi-bin/amigo/go.cgi?view=details&query=GO:0046872) |  |
| [203178_at](https://www.affymetrix.com/LinkServlet?&probeset=203178_at) | GATM | glycine amidinotransferase (L-arginine:glycine amidinotransferase) | 15 | -45653323 | [NM_001482](http://www.ncbi.nlm.nih.gov/entrez/query.fcgi?cmd=search&db=nucleotide&term=NM_001482%5BACCN%5D&doptcmdl=GenBank) | [2628](http://www.ncbi.nlm.nih.gov/sites/entrez?Db=gene&Cmd=DetailsSearch&Term=2628) | [15q21.1](http://www.ncbi.nlm.nih.gov/mapview/map_search.cgi?direct=on&idtype=gene&id=2628) | [Hs.75335](http://www.ncbi.nlm.nih.gov/UniGene/clust.cgi?ORG=Hs&CID=75335) | [27](http://www.ncbi.nih.gov/entrez/query.fcgi?tool=bioconductor&cmd=Retrieve&db=PubMed&list_uids=3800397%2C8125298%2C8313955%2C9148748%2C9165070%2C9218780%2C9266688%2C9373149%2C9915841%2C10893433%2C11555793%2C12324495%2C12468279%2C12477932%2C12701824%2C12709373%2C14702039%2C15489334%2C15978539%2C16614068%2C16769397%2C16820567%2C17101918%2C19430482%2C20383146%2C20682460%2C20877624) | [protein binding](http://amigo.geneontology.org/cgi-bin/amigo/go.cgi?view=details&query=GO:0005515)  [cytoplasm](http://amigo.geneontology.org/cgi-bin/amigo/go.cgi?view=details&query=GO:0005737)  [mitochondrion](http://amigo.geneontology.org/cgi-bin/amigo/go.cgi?view=details&query=GO:0005739)  [mitochondrial inner membrane](http://amigo.geneontology.org/cgi-bin/amigo/go.cgi?view=details&query=GO:0005743)  [mitochondrial intermembrane space](http://amigo.geneontology.org/cgi-bin/amigo/go.cgi?view=details&query=GO:0005758)  [mitochondrial intermembrane space](http://amigo.geneontology.org/cgi-bin/amigo/go.cgi?view=details&query=GO:0005758)  [creatine metabolic process](http://amigo.geneontology.org/cgi-bin/amigo/go.cgi?view=details&query=GO:0006600)  [creatine biosynthetic process](http://amigo.geneontology.org/cgi-bin/amigo/go.cgi?view=details&query=GO:0006601)  [glycine amidinotransferase activity](http://amigo.geneontology.org/cgi-bin/amigo/go.cgi?view=details&query=GO:0015068)  [glycine amidinotransferase activity](http://amigo.geneontology.org/cgi-bin/amigo/go.cgi?view=details&query=GO:0015068)  [membrane](http://amigo.geneontology.org/cgi-bin/amigo/go.cgi?view=details&query=GO:0016020)  [transferase activity](http://amigo.geneontology.org/cgi-bin/amigo/go.cgi?view=details&query=GO:0016740)  [cellular nitrogen compound metabolic process](http://amigo.geneontology.org/cgi-bin/amigo/go.cgi?view=details&query=GO:0034641) | [Glycine, serine and threonine metabolism](http://www.genome.ad.jp/dbget-bin/show_pathway?MAP00260+2.1.4.1)  [Arginine and proline metabolism](http://www.genome.ad.jp/dbget-bin/show_pathway?MAP00330+2.1.4.1)  [Metabolic pathways](http://www.genome.ad.jp/dbget-bin/show_pathway?MAP01100+2.1.4.1) |
| [203215_s_at](https://www.affymetrix.com/LinkServlet?&probeset=203215_s_at) | MYO6 | myosin VI | 6 | 76458908 | [AA877789](http://www.ncbi.nlm.nih.gov/entrez/query.fcgi?cmd=search&db=nucleotide&term=AA877789%5BACCN%5D&doptcmdl=GenBank) | [4646](http://www.ncbi.nlm.nih.gov/sites/entrez?Db=gene&Cmd=DetailsSearch&Term=4646) | [6q13](http://www.ncbi.nlm.nih.gov/mapview/map_search.cgi?direct=on&idtype=gene&id=4646) | [Hs.149387](http://www.ncbi.nlm.nih.gov/UniGene/clust.cgi?ORG=Hs&CID=149387) | [59](http://www.ncbi.nih.gov/entrez/query.fcgi?tool=bioconductor&cmd=Retrieve&db=PubMed&list_uids=7493015%2C7929586%2C8022818%2C9205841%2C9259267%2C9852149%2C10198040%2C10519557%2C11447109%2C11468689%2C11517222%2C11707568%2C11728438%2C11906161%2C11967127%2C12050163%2C12477932%2C12687499%2C12857860%2C12893809%2C14574404%2C14702039%2C15044955%2C15060111%2C15123708%2C15146066%2C15231748%2C15247260%2C15837803%2C16169070%2C16344560%2C16499958%2C16507995%2C16908842%2C16948370%2C16949370%2C17071605%2C17187061%2C17353931%2C17635994%2C17683200%2C18029400%2C18212818%2C18311135%2C18348273%2C18429820%2C18543251%2C19615732%2C19855435%2C19893302%2C19913121%2C20201926%2C20353999%2C20379614%2C20576604%2C20604900%2C20628086%2C20850010%2C20936779) | [nucleotide binding](http://amigo.geneontology.org/cgi-bin/amigo/go.cgi?view=details&query=GO:0000166)  [ruffle](http://amigo.geneontology.org/cgi-bin/amigo/go.cgi?view=details&query=GO:0001726)  [motor activity](http://amigo.geneontology.org/cgi-bin/amigo/go.cgi?view=details&query=GO:0003774)  [actin binding](http://amigo.geneontology.org/cgi-bin/amigo/go.cgi?view=details&query=GO:0003779)  [protein binding](http://amigo.geneontology.org/cgi-bin/amigo/go.cgi?view=details&query=GO:0005515)  [calmodulin binding](http://amigo.geneontology.org/cgi-bin/amigo/go.cgi?view=details&query=GO:0005516)  [calmodulin binding](http://amigo.geneontology.org/cgi-bin/amigo/go.cgi?view=details&query=GO:0005516)  [ATP binding](http://amigo.geneontology.org/cgi-bin/amigo/go.cgi?view=details&query=GO:0005524)  [nucleus](http://amigo.geneontology.org/cgi-bin/amigo/go.cgi?view=details&query=GO:0005634)  [nucleoplasm](http://amigo.geneontology.org/cgi-bin/amigo/go.cgi?view=details&query=GO:0005654)  [cytoplasm](http://amigo.geneontology.org/cgi-bin/amigo/go.cgi?view=details&query=GO:0005737)  [cytoplasm](http://amigo.geneontology.org/cgi-bin/amigo/go.cgi?view=details&query=GO:0005737)  [Golgi apparatus](http://amigo.geneontology.org/cgi-bin/amigo/go.cgi?view=details&query=GO:0005794)  [cytosol](http://amigo.geneontology.org/cgi-bin/amigo/go.cgi?view=details&query=GO:0005829)  [plasma membrane](http://amigo.geneontology.org/cgi-bin/amigo/go.cgi?view=details&query=GO:0005886)  [coated pit](http://amigo.geneontology.org/cgi-bin/amigo/go.cgi?view=details&query=GO:0005905)  [cell cortex](http://amigo.geneontology.org/cgi-bin/amigo/go.cgi?view=details&query=GO:0005938)  [intracellular protein transport](http://amigo.geneontology.org/cgi-bin/amigo/go.cgi?view=details&query=GO:0006886)  [endocytosis](http://amigo.geneontology.org/cgi-bin/amigo/go.cgi?view=details&query=GO:0006897)  [endocytosis](http://amigo.geneontology.org/cgi-bin/amigo/go.cgi?view=details&query=GO:0006897)  [synaptic transmission](http://amigo.geneontology.org/cgi-bin/amigo/go.cgi?view=details&query=GO:0007268)  [sensory perception of sound](http://amigo.geneontology.org/cgi-bin/amigo/go.cgi?view=details&query=GO:0007605)  [cytoplasmic membrane-bounded vesicle](http://amigo.geneontology.org/cgi-bin/amigo/go.cgi?view=details&query=GO:0016023)  [unconventional myosin complex](http://amigo.geneontology.org/cgi-bin/amigo/go.cgi?view=details&query=GO:0016461)  [DNA-directed RNA polymerase II, holoenzyme](http://amigo.geneontology.org/cgi-bin/amigo/go.cgi?view=details&query=GO:0016591)  [actin filament-based movement](http://amigo.geneontology.org/cgi-bin/amigo/go.cgi?view=details&query=GO:0030048)  [actin filament-based movement](http://amigo.geneontology.org/cgi-bin/amigo/go.cgi?view=details&query=GO:0030048)  [endocytic vesicle](http://amigo.geneontology.org/cgi-bin/amigo/go.cgi?view=details&query=GO:0030139)  [DNA damage response, signal transduction by p53 class mediator](http://amigo.geneontology.org/cgi-bin/amigo/go.cgi?view=details&query=GO:0030330)  [clathrin coated vesicle membrane](http://amigo.geneontology.org/cgi-bin/amigo/go.cgi?view=details&query=GO:0030665)  [filamentous actin](http://amigo.geneontology.org/cgi-bin/amigo/go.cgi?view=details&query=GO:0031941)  [filamentous actin](http://amigo.geneontology.org/cgi-bin/amigo/go.cgi?view=details&query=GO:0031941)  [nuclear membrane](http://amigo.geneontology.org/cgi-bin/amigo/go.cgi?view=details&query=GO:0031965)  [ruffle membrane](http://amigo.geneontology.org/cgi-bin/amigo/go.cgi?view=details&query=GO:0032587)  [ADP binding](http://amigo.geneontology.org/cgi-bin/amigo/go.cgi?view=details&query=GO:0043531)  [clathrin-coated endocytic vesicle](http://amigo.geneontology.org/cgi-bin/amigo/go.cgi?view=details&query=GO:0045334)  [positive regulation of transcription from RNA polymerase II promoter](http://amigo.geneontology.org/cgi-bin/amigo/go.cgi?view=details&query=GO:0045944)  [perinuclear region of cytoplasm](http://amigo.geneontology.org/cgi-bin/amigo/go.cgi?view=details&query=GO:0048471)  [perinuclear region of cytoplasm](http://amigo.geneontology.org/cgi-bin/amigo/go.cgi?view=details&query=GO:0048471)  [actin filament binding](http://amigo.geneontology.org/cgi-bin/amigo/go.cgi?view=details&query=GO:0051015)  [actin filament binding](http://amigo.geneontology.org/cgi-bin/amigo/go.cgi?view=details&query=GO:0051015)  [regulation of secretion](http://amigo.geneontology.org/cgi-bin/amigo/go.cgi?view=details&query=GO:0051046)  [minus-end directed microfilament motor activity](http://amigo.geneontology.org/cgi-bin/amigo/go.cgi?view=details&query=GO:0060001) |  |
| [203216_s_at](https://www.affymetrix.com/LinkServlet?&probeset=203216_s_at) | MYO6 | myosin VI | 6 | 76458908 | [NM_004999](http://www.ncbi.nlm.nih.gov/entrez/query.fcgi?cmd=search&db=nucleotide&term=NM_004999%5BACCN%5D&doptcmdl=GenBank) | [4646](http://www.ncbi.nlm.nih.gov/sites/entrez?Db=gene&Cmd=DetailsSearch&Term=4646) | [6q13](http://www.ncbi.nlm.nih.gov/mapview/map_search.cgi?direct=on&idtype=gene&id=4646) | [Hs.149387](http://www.ncbi.nlm.nih.gov/UniGene/clust.cgi?ORG=Hs&CID=149387) | [59](http://www.ncbi.nih.gov/entrez/query.fcgi?tool=bioconductor&cmd=Retrieve&db=PubMed&list_uids=7493015%2C7929586%2C8022818%2C9205841%2C9259267%2C9852149%2C10198040%2C10519557%2C11447109%2C11468689%2C11517222%2C11707568%2C11728438%2C11906161%2C11967127%2C12050163%2C12477932%2C12687499%2C12857860%2C12893809%2C14574404%2C14702039%2C15044955%2C15060111%2C15123708%2C15146066%2C15231748%2C15247260%2C15837803%2C16169070%2C16344560%2C16499958%2C16507995%2C16908842%2C16948370%2C16949370%2C17071605%2C17187061%2C17353931%2C17635994%2C17683200%2C18029400%2C18212818%2C18311135%2C18348273%2C18429820%2C18543251%2C19615732%2C19855435%2C19893302%2C19913121%2C20201926%2C20353999%2C20379614%2C20576604%2C20604900%2C20628086%2C20850010%2C20936779) | [nucleotide binding](http://amigo.geneontology.org/cgi-bin/amigo/go.cgi?view=details&query=GO:0000166)  [ruffle](http://amigo.geneontology.org/cgi-bin/amigo/go.cgi?view=details&query=GO:0001726)  [motor activity](http://amigo.geneontology.org/cgi-bin/amigo/go.cgi?view=details&query=GO:0003774)  [actin binding](http://amigo.geneontology.org/cgi-bin/amigo/go.cgi?view=details&query=GO:0003779)  [protein binding](http://amigo.geneontology.org/cgi-bin/amigo/go.cgi?view=details&query=GO:0005515)  [calmodulin binding](http://amigo.geneontology.org/cgi-bin/amigo/go.cgi?view=details&query=GO:0005516)  [calmodulin binding](http://amigo.geneontology.org/cgi-bin/amigo/go.cgi?view=details&query=GO:0005516)  [ATP binding](http://amigo.geneontology.org/cgi-bin/amigo/go.cgi?view=details&query=GO:0005524)  [nucleus](http://amigo.geneontology.org/cgi-bin/amigo/go.cgi?view=details&query=GO:0005634)  [nucleoplasm](http://amigo.geneontology.org/cgi-bin/amigo/go.cgi?view=details&query=GO:0005654)  [cytoplasm](http://amigo.geneontology.org/cgi-bin/amigo/go.cgi?view=details&query=GO:0005737)  [cytoplasm](http://amigo.geneontology.org/cgi-bin/amigo/go.cgi?view=details&query=GO:0005737)  [Golgi apparatus](http://amigo.geneontology.org/cgi-bin/amigo/go.cgi?view=details&query=GO:0005794)  [cytosol](http://amigo.geneontology.org/cgi-bin/amigo/go.cgi?view=details&query=GO:0005829)  [plasma membrane](http://amigo.geneontology.org/cgi-bin/amigo/go.cgi?view=details&query=GO:0005886)  [coated pit](http://amigo.geneontology.org/cgi-bin/amigo/go.cgi?view=details&query=GO:0005905)  [cell cortex](http://amigo.geneontology.org/cgi-bin/amigo/go.cgi?view=details&query=GO:0005938)  [intracellular protein transport](http://amigo.geneontology.org/cgi-bin/amigo/go.cgi?view=details&query=GO:0006886)  [endocytosis](http://amigo.geneontology.org/cgi-bin/amigo/go.cgi?view=details&query=GO:0006897)  [endocytosis](http://amigo.geneontology.org/cgi-bin/amigo/go.cgi?view=details&query=GO:0006897)  [synaptic transmission](http://amigo.geneontology.org/cgi-bin/amigo/go.cgi?view=details&query=GO:0007268)  [sensory perception of sound](http://amigo.geneontology.org/cgi-bin/amigo/go.cgi?view=details&query=GO:0007605)  [cytoplasmic membrane-bounded vesicle](http://amigo.geneontology.org/cgi-bin/amigo/go.cgi?view=details&query=GO:0016023)  [unconventional myosin complex](http://amigo.geneontology.org/cgi-bin/amigo/go.cgi?view=details&query=GO:0016461)  [DNA-directed RNA polymerase II, holoenzyme](http://amigo.geneontology.org/cgi-bin/amigo/go.cgi?view=details&query=GO:0016591)  [actin filament-based movement](http://amigo.geneontology.org/cgi-bin/amigo/go.cgi?view=details&query=GO:0030048)  [actin filament-based movement](http://amigo.geneontology.org/cgi-bin/amigo/go.cgi?view=details&query=GO:0030048)  [endocytic vesicle](http://amigo.geneontology.org/cgi-bin/amigo/go.cgi?view=details&query=GO:0030139)  [DNA damage response, signal transduction by p53 class mediator](http://amigo.geneontology.org/cgi-bin/amigo/go.cgi?view=details&query=GO:0030330)  [clathrin coated vesicle membrane](http://amigo.geneontology.org/cgi-bin/amigo/go.cgi?view=details&query=GO:0030665)  [filamentous actin](http://amigo.geneontology.org/cgi-bin/amigo/go.cgi?view=details&query=GO:0031941)  [filamentous actin](http://amigo.geneontology.org/cgi-bin/amigo/go.cgi?view=details&query=GO:0031941)  [nuclear membrane](http://amigo.geneontology.org/cgi-bin/amigo/go.cgi?view=details&query=GO:0031965)  [ruffle membrane](http://amigo.geneontology.org/cgi-bin/amigo/go.cgi?view=details&query=GO:0032587)  [ADP binding](http://amigo.geneontology.org/cgi-bin/amigo/go.cgi?view=details&query=GO:0043531)  [clathrin-coated endocytic vesicle](http://amigo.geneontology.org/cgi-bin/amigo/go.cgi?view=details&query=GO:0045334)  [positive regulation of transcription from RNA polymerase II promoter](http://amigo.geneontology.org/cgi-bin/amigo/go.cgi?view=details&query=GO:0045944)  [perinuclear region of cytoplasm](http://amigo.geneontology.org/cgi-bin/amigo/go.cgi?view=details&query=GO:0048471)  [perinuclear region of cytoplasm](http://amigo.geneontology.org/cgi-bin/amigo/go.cgi?view=details&query=GO:0048471)  [actin filament binding](http://amigo.geneontology.org/cgi-bin/amigo/go.cgi?view=details&query=GO:0051015)  [actin filament binding](http://amigo.geneontology.org/cgi-bin/amigo/go.cgi?view=details&query=GO:0051015)  [regulation of secretion](http://amigo.geneontology.org/cgi-bin/amigo/go.cgi?view=details&query=GO:0051046)  [minus-end directed microfilament motor activity](http://amigo.geneontology.org/cgi-bin/amigo/go.cgi?view=details&query=GO:0060001) |  |
| [203303_at](https://www.affymetrix.com/LinkServlet?&probeset=203303_at) | DYNLT3 | dynein, light chain, Tctex-type 3 | X | -37698089 | [NM_006520](http://www.ncbi.nlm.nih.gov/entrez/query.fcgi?cmd=search&db=nucleotide&term=NM_006520%5BACCN%5D&doptcmdl=GenBank) | [6990](http://www.ncbi.nlm.nih.gov/sites/entrez?Db=gene&Cmd=DetailsSearch&Term=6990) | [Xp21](http://www.ncbi.nlm.nih.gov/mapview/map_search.cgi?direct=on&idtype=gene&id=6990) | [Hs.446392](http://www.ncbi.nlm.nih.gov/UniGene/clust.cgi?ORG=Hs&CID=446392) | [18](http://www.ncbi.nih.gov/entrez/query.fcgi?tool=bioconductor&cmd=Retrieve&db=PubMed&list_uids=8004092%2C9692886%2C9790665%2C11425878%2C11746667%2C11751937%2C12009301%2C12475239%2C12477932%2C15117959%2C15489334%2C16079286%2C16189514%2C16344560%2C17289665%2C17676955%2C18029348%2C20668116) | [kinetochore](http://amigo.geneontology.org/cgi-bin/amigo/go.cgi?view=details&query=GO:0000776)  [condensed chromosome kinetochore](http://amigo.geneontology.org/cgi-bin/amigo/go.cgi?view=details&query=GO:0000777)  [motor activity](http://amigo.geneontology.org/cgi-bin/amigo/go.cgi?view=details&query=GO:0003774)  [protein binding](http://amigo.geneontology.org/cgi-bin/amigo/go.cgi?view=details&query=GO:0005515)  [nucleus](http://amigo.geneontology.org/cgi-bin/amigo/go.cgi?view=details&query=GO:0005634)  [cytoplasm](http://amigo.geneontology.org/cgi-bin/amigo/go.cgi?view=details&query=GO:0005737)  [cytoskeleton](http://amigo.geneontology.org/cgi-bin/amigo/go.cgi?view=details&query=GO:0005856)  [cytoplasmic dynein complex](http://amigo.geneontology.org/cgi-bin/amigo/go.cgi?view=details&query=GO:0005868)  [microtubule](http://amigo.geneontology.org/cgi-bin/amigo/go.cgi?view=details&query=GO:0005874)  [plasma membrane](http://amigo.geneontology.org/cgi-bin/amigo/go.cgi?view=details&query=GO:0005886)  [transport](http://amigo.geneontology.org/cgi-bin/amigo/go.cgi?view=details&query=GO:0006810)  [cell cycle](http://amigo.geneontology.org/cgi-bin/amigo/go.cgi?view=details&query=GO:0007049)  [mitosis](http://amigo.geneontology.org/cgi-bin/amigo/go.cgi?view=details&query=GO:0007067)  [regulation of mitotic cell cycle](http://amigo.geneontology.org/cgi-bin/amigo/go.cgi?view=details&query=GO:0007346)  [cell division](http://amigo.geneontology.org/cgi-bin/amigo/go.cgi?view=details&query=GO:0051301) |  |
| [203410_at](https://www.affymetrix.com/LinkServlet?&probeset=203410_at) | AP3M2 | adaptor-related protein complex 3, mu 2 subunit | 8 | 42010463 | [NM_006803](http://www.ncbi.nlm.nih.gov/entrez/query.fcgi?cmd=search&db=nucleotide&term=NM_006803%5BACCN%5D&doptcmdl=GenBank) | [10947](http://www.ncbi.nlm.nih.gov/sites/entrez?Db=gene&Cmd=DetailsSearch&Term=10947) | [8p11.2](http://www.ncbi.nlm.nih.gov/mapview/map_search.cgi?direct=on&idtype=gene&id=10947) | [Hs.654529](http://www.ncbi.nlm.nih.gov/UniGene/clust.cgi?ORG=Hs&CID=654529) | [8](http://www.ncbi.nih.gov/entrez/query.fcgi?tool=bioconductor&cmd=Retrieve&db=PubMed&list_uids=1602151%2C7601449%2C8076832%2C9400603%2C12477932%2C15489334%2C17293072%2C19481122) | [Golgi apparatus](http://amigo.geneontology.org/cgi-bin/amigo/go.cgi?view=details&query=GO:0005794)  [intracellular protein transport](http://amigo.geneontology.org/cgi-bin/amigo/go.cgi?view=details&query=GO:0006886)  [vesicle-mediated transport](http://amigo.geneontology.org/cgi-bin/amigo/go.cgi?view=details&query=GO:0016192)  [AP-type membrane coat adaptor complex](http://amigo.geneontology.org/cgi-bin/amigo/go.cgi?view=details&query=GO:0030119)  [clathrin adaptor complex](http://amigo.geneontology.org/cgi-bin/amigo/go.cgi?view=details&query=GO:0030131) | [Lysosome](http://www.genome.ad.jp/kegg/pathway/hsa/hsa04142.html) |
| [203438_at](https://www.affymetrix.com/LinkServlet?&probeset=203438_at) | STC2 | stanniocalcin 2 | 5 | -172741725 | [AI435828](http://www.ncbi.nlm.nih.gov/entrez/query.fcgi?cmd=search&db=nucleotide&term=AI435828%5BACCN%5D&doptcmdl=GenBank) | [8614](http://www.ncbi.nlm.nih.gov/sites/entrez?Db=gene&Cmd=DetailsSearch&Term=8614) | [5q35.1](http://www.ncbi.nlm.nih.gov/mapview/map_search.cgi?direct=on&idtype=gene&id=8614) | [Hs.233160](http://www.ncbi.nlm.nih.gov/UniGene/clust.cgi?ORG=Hs&CID=233160) | [26](http://www.ncbi.nih.gov/entrez/query.fcgi?tool=bioconductor&cmd=Retrieve&db=PubMed&list_uids=9723890%2C9753616%2C10022771%2C10450831%2C10947959%2C12477932%2C14702039%2C15302935%2C15367391%2C15486227%2C15489334%2C16169070%2C16303743%2C17545519%2C17909264%2C18355956%2C18394600%2C18492817%2C19298603%2C19415750%2C19582875%2C19786016%2C20174869%2C20422456%2C20424473%2C20619259) | [hormone activity](http://amigo.geneontology.org/cgi-bin/amigo/go.cgi?view=details&query=GO:0005179)  [extracellular region](http://amigo.geneontology.org/cgi-bin/amigo/go.cgi?view=details&query=GO:0005576)  [cell surface receptor linked signaling pathway](http://amigo.geneontology.org/cgi-bin/amigo/go.cgi?view=details&query=GO:0007166)  [cell-cell signaling](http://amigo.geneontology.org/cgi-bin/amigo/go.cgi?view=details&query=GO:0007267)  [embryo implantation](http://amigo.geneontology.org/cgi-bin/amigo/go.cgi?view=details&query=GO:0007566)  [response to nutrient](http://amigo.geneontology.org/cgi-bin/amigo/go.cgi?view=details&query=GO:0007584)  [response to vitamin D](http://amigo.geneontology.org/cgi-bin/amigo/go.cgi?view=details&query=GO:0033280)  [response to peptide hormone stimulus](http://amigo.geneontology.org/cgi-bin/amigo/go.cgi?view=details&query=GO:0043434)  [decidualization](http://amigo.geneontology.org/cgi-bin/amigo/go.cgi?view=details&query=GO:0046697)  [cellular response to hypoxia](http://amigo.geneontology.org/cgi-bin/amigo/go.cgi?view=details&query=GO:0071456) |  |
| [203636_at](https://www.affymetrix.com/LinkServlet?&probeset=203636_at) | MID1 | midline 1 (Opitz/BBB syndrome) | X | -10473381, -10437305, -10413349, -10413349, -10413349, -10413349 | [BE967532](http://www.ncbi.nlm.nih.gov/entrez/query.fcgi?cmd=search&db=nucleotide&term=BE967532%5BACCN%5D&doptcmdl=GenBank) | [4281](http://www.ncbi.nlm.nih.gov/sites/entrez?Db=gene&Cmd=DetailsSearch&Term=4281) | [Xp22](http://www.ncbi.nlm.nih.gov/mapview/map_search.cgi?direct=on&idtype=gene&id=4281) | [Hs.27695](http://www.ncbi.nlm.nih.gov/UniGene/clust.cgi?ORG=Hs&CID=27695) [Hs.689953](http://www.ncbi.nlm.nih.gov/UniGene/clust.cgi?ORG=Hs&CID=689953) | [41](http://www.ncbi.nih.gov/entrez/query.fcgi?tool=bioconductor&cmd=Retrieve&db=PubMed&list_uids=7493033%2C9354791%2C9425238%2C9718340%2C9722948%2C10077590%2C10400985%2C11030761%2C11331580%2C11371618%2C11685209%2C11806752%2C12408967%2C12411602%2C12477932%2C12545276%2C12798296%2C12833403%2C15057556%2C15070402%2C15489334%2C15558842%2C16344560%2C16378742%2C16498413%2C16529770%2C17043407%2C17081983%2C17221865%2C17428496%2C17438131%2C17672918%2C18005432%2C18172692%2C18220417%2C18360914%2C18697196%2C18949047%2C19049519%2C19549727%2C20301502) | [microtubule cytoskeleton organization](http://amigo.geneontology.org/cgi-bin/amigo/go.cgi?view=details&query=GO:0000226)  [intracellular](http://amigo.geneontology.org/cgi-bin/amigo/go.cgi?view=details&query=GO:0005622)  [cytoplasm](http://amigo.geneontology.org/cgi-bin/amigo/go.cgi?view=details&query=GO:0005737)  [spindle](http://amigo.geneontology.org/cgi-bin/amigo/go.cgi?view=details&query=GO:0005819)  [microtubule associated complex](http://amigo.geneontology.org/cgi-bin/amigo/go.cgi?view=details&query=GO:0005875)  [cytoplasmic microtubule](http://amigo.geneontology.org/cgi-bin/amigo/go.cgi?view=details&query=GO:0005881)  [negative regulation of microtubule depolymerization](http://amigo.geneontology.org/cgi-bin/amigo/go.cgi?view=details&query=GO:0007026)  [pattern specification process](http://amigo.geneontology.org/cgi-bin/amigo/go.cgi?view=details&query=GO:0007389)  [zinc ion binding](http://amigo.geneontology.org/cgi-bin/amigo/go.cgi?view=details&query=GO:0008270)  [ligase activity](http://amigo.geneontology.org/cgi-bin/amigo/go.cgi?view=details&query=GO:0016874)  [ubiquitin protein ligase binding](http://amigo.geneontology.org/cgi-bin/amigo/go.cgi?view=details&query=GO:0031625)  [positive regulation of stress-activated MAPK cascade](http://amigo.geneontology.org/cgi-bin/amigo/go.cgi?view=details&query=GO:0032874)  [metal ion binding](http://amigo.geneontology.org/cgi-bin/amigo/go.cgi?view=details&query=GO:0046872) | [Ubiquitin mediated proteolysis](http://www.genome.ad.jp/dbget-bin/show_pathway?MAP04120+6.3.2.19) |
| [203637_s_at](https://www.affymetrix.com/LinkServlet?&probeset=203637_s_at) | MID1 | midline 1 (Opitz/BBB syndrome) | X | -10473381, -10437305, -10413349, -10413349, -10413349, -10413349 | [NM_000381](http://www.ncbi.nlm.nih.gov/entrez/query.fcgi?cmd=search&db=nucleotide&term=NM_000381%5BACCN%5D&doptcmdl=GenBank) | [4281](http://www.ncbi.nlm.nih.gov/sites/entrez?Db=gene&Cmd=DetailsSearch&Term=4281) | [Xp22](http://www.ncbi.nlm.nih.gov/mapview/map_search.cgi?direct=on&idtype=gene&id=4281) | [Hs.27695](http://www.ncbi.nlm.nih.gov/UniGene/clust.cgi?ORG=Hs&CID=27695) [Hs.689953](http://www.ncbi.nlm.nih.gov/UniGene/clust.cgi?ORG=Hs&CID=689953) | [41](http://www.ncbi.nih.gov/entrez/query.fcgi?tool=bioconductor&cmd=Retrieve&db=PubMed&list_uids=7493033%2C9354791%2C9425238%2C9718340%2C9722948%2C10077590%2C10400985%2C11030761%2C11331580%2C11371618%2C11685209%2C11806752%2C12408967%2C12411602%2C12477932%2C12545276%2C12798296%2C12833403%2C15057556%2C15070402%2C15489334%2C15558842%2C16344560%2C16378742%2C16498413%2C16529770%2C17043407%2C17081983%2C17221865%2C17428496%2C17438131%2C17672918%2C18005432%2C18172692%2C18220417%2C18360914%2C18697196%2C18949047%2C19049519%2C19549727%2C20301502) | [microtubule cytoskeleton organization](http://amigo.geneontology.org/cgi-bin/amigo/go.cgi?view=details&query=GO:0000226)  [intracellular](http://amigo.geneontology.org/cgi-bin/amigo/go.cgi?view=details&query=GO:0005622)  [cytoplasm](http://amigo.geneontology.org/cgi-bin/amigo/go.cgi?view=details&query=GO:0005737)  [spindle](http://amigo.geneontology.org/cgi-bin/amigo/go.cgi?view=details&query=GO:0005819)  [microtubule associated complex](http://amigo.geneontology.org/cgi-bin/amigo/go.cgi?view=details&query=GO:0005875)  [cytoplasmic microtubule](http://amigo.geneontology.org/cgi-bin/amigo/go.cgi?view=details&query=GO:0005881)  [negative regulation of microtubule depolymerization](http://amigo.geneontology.org/cgi-bin/amigo/go.cgi?view=details&query=GO:0007026)  [pattern specification process](http://amigo.geneontology.org/cgi-bin/amigo/go.cgi?view=details&query=GO:0007389)  [zinc ion binding](http://amigo.geneontology.org/cgi-bin/amigo/go.cgi?view=details&query=GO:0008270)  [ligase activity](http://amigo.geneontology.org/cgi-bin/amigo/go.cgi?view=details&query=GO:0016874)  [ubiquitin protein ligase binding](http://amigo.geneontology.org/cgi-bin/amigo/go.cgi?view=details&query=GO:0031625)  [positive regulation of stress-activated MAPK cascade](http://amigo.geneontology.org/cgi-bin/amigo/go.cgi?view=details&query=GO:0032874)  [metal ion binding](http://amigo.geneontology.org/cgi-bin/amigo/go.cgi?view=details&query=GO:0046872) | [Ubiquitin mediated proteolysis](http://www.genome.ad.jp/dbget-bin/show_pathway?MAP04120+6.3.2.19) |
| [203695_s_at](https://www.affymetrix.com/LinkServlet?&probeset=203695_s_at) | DFNA5 | deafness, autosomal dominant 5 | 7 | -24737973, -24737973 | [NM_004403](http://www.ncbi.nlm.nih.gov/entrez/query.fcgi?cmd=search&db=nucleotide&term=NM_004403%5BACCN%5D&doptcmdl=GenBank) | [1687](http://www.ncbi.nlm.nih.gov/sites/entrez?Db=gene&Cmd=DetailsSearch&Term=1687) | [7p15](http://www.ncbi.nlm.nih.gov/mapview/map_search.cgi?direct=on&idtype=gene&id=1687) | [Hs.520708](http://www.ncbi.nlm.nih.gov/UniGene/clust.cgi?ORG=Hs&CID=520708) | [26](http://www.ncbi.nih.gov/entrez/query.fcgi?tool=bioconductor&cmd=Retrieve&db=PubMed&list_uids=8589696%2C8619474%2C9110174%2C9450185%2C9523727%2C9771715%2C11058868%2C12461698%2C12477932%2C12853124%2C12853948%2C14559215%2C14676472%2C14702039%2C15489334%2C16169070%2C16344560%2C16897187%2C17427029%2C17616391%2C17868390%2C18223688%2C18346456%2C19911014%2C20379614%2C20403915) | [sensory perception of sound](http://amigo.geneontology.org/cgi-bin/amigo/go.cgi?view=details&query=GO:0007605) |  |
| [204042_at](https://www.affymetrix.com/LinkServlet?&probeset=204042_at) | WASF3 | WAS protein family, member 3 | 13 | 27131839 | [AB020707](http://www.ncbi.nlm.nih.gov/entrez/query.fcgi?cmd=search&db=nucleotide&term=AB020707%5BACCN%5D&doptcmdl=GenBank) | [10810](http://www.ncbi.nlm.nih.gov/sites/entrez?Db=gene&Cmd=DetailsSearch&Term=10810) | [13q12](http://www.ncbi.nlm.nih.gov/mapview/map_search.cgi?direct=on&idtype=gene&id=10810) | [Hs.635221](http://www.ncbi.nlm.nih.gov/UniGene/clust.cgi?ORG=Hs&CID=635221) | [21](http://www.ncbi.nih.gov/entrez/query.fcgi?tool=bioconductor&cmd=Retrieve&db=PubMed&list_uids=8137235%2C8889548%2C9732292%2C10048485%2C10381382%2C11073096%2C11146629%2C11246027%2C12168954%2C12185600%2C12477932%2C12856283%2C15057823%2C15280206%2C15670045%2C15752430%2C15826941%2C15907837%2C17623672%2C19395286%2C19801681) | [actin binding](http://amigo.geneontology.org/cgi-bin/amigo/go.cgi?view=details&query=GO:0003779)  [cytoplasm](http://amigo.geneontology.org/cgi-bin/amigo/go.cgi?view=details&query=GO:0005737)  [cytoskeleton](http://amigo.geneontology.org/cgi-bin/amigo/go.cgi?view=details&query=GO:0005856)  [protein complex assembly](http://amigo.geneontology.org/cgi-bin/amigo/go.cgi?view=details&query=GO:0006461)  [actin filament polymerization](http://amigo.geneontology.org/cgi-bin/amigo/go.cgi?view=details&query=GO:0030041) | [Adherens junction](http://www.genome.ad.jp/kegg/pathway/hsa/hsa04520.html)  [Fc gamma R-mediated phagocytosis](http://www.genome.ad.jp/kegg/pathway/hsa/hsa04666.html) |
| [204197_s_at](https://www.affymetrix.com/LinkServlet?&probeset=204197_s_at) | RUNX3 | runt-related transcription factor 3 | 1 | -25226002, -25226002 | [NM_004350](http://www.ncbi.nlm.nih.gov/entrez/query.fcgi?cmd=search&db=nucleotide&term=NM_004350%5BACCN%5D&doptcmdl=GenBank) | [864](http://www.ncbi.nlm.nih.gov/sites/entrez?Db=gene&Cmd=DetailsSearch&Term=864) | [1p36](http://www.ncbi.nlm.nih.gov/mapview/map_search.cgi?direct=on&idtype=gene&id=864) | [Hs.170019](http://www.ncbi.nlm.nih.gov/UniGene/clust.cgi?ORG=Hs&CID=170019) | [133](http://www.ncbi.nih.gov/entrez/query.fcgi?tool=bioconductor&cmd=Retrieve&db=PubMed&list_uids=7607690%2C7622058%2C7835892%2C8437866%2C9751710%2C10228168%2C10531362%2C11733147%2C11955451%2C12477932%2C12824905%2C12855590%2C12875960%2C12907736%2C14702039%2C14715269%2C14760761%2C15051926%2C15138260%2C15273736%2C15386419%2C15489334%2C15688019%2C15728469%2C15778373%2C15780064%2C15819721%2C15824739%2C16080503%2C16091737%2C16135801%2C16140942%2C16142337%2C16155404%2C16234815%2C16328045%2C16344560%2C16367921%2C16373335%2C16442267%2C16582583%2C16627973%2C16652147%2C16684349%2C16710414%2C16767156%2C16818622%2C16887969%2C16984612%2C17195845%2C17353931%2C17380460%2C17384682%2C17470130%2C17471240%2C17584746%2C17591800%2C17591929%2C17606310%2C17914577%2C17923751%2C17956589%2C18029348%2C18058463%2C18097595%2C18256927%2C18259121%2C18288406%2C18323800%2C18349282%2C18426645%2C18430739%2C18475302%2C18494051%2C18500170%2C18572225%2C18580070%2C18636364%2C18639281%2C18663147%2C18668679%2C18676844%2C18684727%2C18717361%2C18772112%2C18850007%2C18937968%2C18949360%2C18953836%2C19012242%2C19015875%2C19159630%2C19174785%2C19223906%2C19290488%2C19336521%2C19403666%2C19470943%2C19521519%2C19552756%2C19571605%2C19596937%2C19603429%2C19645591%2C19695681%2C19706291%2C19728008%2C19800882%2C19827872%2C19886737%2C19917773%2C19933870%2C20100835%2C20160714%2C20190752%2C20211142%2C20228843%2C20306685%2C20353948%2C20392673%2C20442291%2C20492341%2C20599712%2C20615577%2C20631058%2C20676134%2C20677014%2C20682997%2C20801098%2C20887385%2C20955380%2C21088106%2C21105967) | [DNA binding](http://amigo.geneontology.org/cgi-bin/amigo/go.cgi?view=details&query=GO:0003677)  [sequence-specific DNA binding transcription factor activity](http://amigo.geneontology.org/cgi-bin/amigo/go.cgi?view=details&query=GO:0003700)  [protein binding](http://amigo.geneontology.org/cgi-bin/amigo/go.cgi?view=details&query=GO:0005515)  [ATP binding](http://amigo.geneontology.org/cgi-bin/amigo/go.cgi?view=details&query=GO:0005524)  [nucleus](http://amigo.geneontology.org/cgi-bin/amigo/go.cgi?view=details&query=GO:0005634)  [cytoplasm](http://amigo.geneontology.org/cgi-bin/amigo/go.cgi?view=details&query=GO:0005737)  [regulation of transcription, DNA-dependent](http://amigo.geneontology.org/cgi-bin/amigo/go.cgi?view=details&query=GO:0006355)  [transcription from RNA polymerase II promoter](http://amigo.geneontology.org/cgi-bin/amigo/go.cgi?view=details&query=GO:0006366)  [induction of apoptosis](http://amigo.geneontology.org/cgi-bin/amigo/go.cgi?view=details&query=GO:0006917)  [cell proliferation](http://amigo.geneontology.org/cgi-bin/amigo/go.cgi?view=details&query=GO:0008283)  [negative regulation of cell cycle](http://amigo.geneontology.org/cgi-bin/amigo/go.cgi?view=details&query=GO:0045786)  [negative regulation of epithelial cell proliferation](http://amigo.geneontology.org/cgi-bin/amigo/go.cgi?view=details&query=GO:0050680) |  |
| [204198_s_at](https://www.affymetrix.com/LinkServlet?&probeset=204198_s_at) | RUNX3 | runt-related transcription factor 3 | 1 | -25226002, -25226002 | [AA541630](http://www.ncbi.nlm.nih.gov/entrez/query.fcgi?cmd=search&db=nucleotide&term=AA541630%5BACCN%5D&doptcmdl=GenBank) | [864](http://www.ncbi.nlm.nih.gov/sites/entrez?Db=gene&Cmd=DetailsSearch&Term=864) | [1p36](http://www.ncbi.nlm.nih.gov/mapview/map_search.cgi?direct=on&idtype=gene&id=864) | [Hs.170019](http://www.ncbi.nlm.nih.gov/UniGene/clust.cgi?ORG=Hs&CID=170019) | [133](http://www.ncbi.nih.gov/entrez/query.fcgi?tool=bioconductor&cmd=Retrieve&db=PubMed&list_uids=7607690%2C7622058%2C7835892%2C8437866%2C9751710%2C10228168%2C10531362%2C11733147%2C11955451%2C12477932%2C12824905%2C12855590%2C12875960%2C12907736%2C14702039%2C14715269%2C14760761%2C15051926%2C15138260%2C15273736%2C15386419%2C15489334%2C15688019%2C15728469%2C15778373%2C15780064%2C15819721%2C15824739%2C16080503%2C16091737%2C16135801%2C16140942%2C16142337%2C16155404%2C16234815%2C16328045%2C16344560%2C16367921%2C16373335%2C16442267%2C16582583%2C16627973%2C16652147%2C16684349%2C16710414%2C16767156%2C16818622%2C16887969%2C16984612%2C17195845%2C17353931%2C17380460%2C17384682%2C17470130%2C17471240%2C17584746%2C17591800%2C17591929%2C17606310%2C17914577%2C17923751%2C17956589%2C18029348%2C18058463%2C18097595%2C18256927%2C18259121%2C18288406%2C18323800%2C18349282%2C18426645%2C18430739%2C18475302%2C18494051%2C18500170%2C18572225%2C18580070%2C18636364%2C18639281%2C18663147%2C18668679%2C18676844%2C18684727%2C18717361%2C18772112%2C18850007%2C18937968%2C18949360%2C18953836%2C19012242%2C19015875%2C19159630%2C19174785%2C19223906%2C19290488%2C19336521%2C19403666%2C19470943%2C19521519%2C19552756%2C19571605%2C19596937%2C19603429%2C19645591%2C19695681%2C19706291%2C19728008%2C19800882%2C19827872%2C19886737%2C19917773%2C19933870%2C20100835%2C20160714%2C20190752%2C20211142%2C20228843%2C20306685%2C20353948%2C20392673%2C20442291%2C20492341%2C20599712%2C20615577%2C20631058%2C20676134%2C20677014%2C20682997%2C20801098%2C20887385%2C20955380%2C21088106%2C21105967) | [DNA binding](http://amigo.geneontology.org/cgi-bin/amigo/go.cgi?view=details&query=GO:0003677)  [sequence-specific DNA binding transcription factor activity](http://amigo.geneontology.org/cgi-bin/amigo/go.cgi?view=details&query=GO:0003700)  [protein binding](http://amigo.geneontology.org/cgi-bin/amigo/go.cgi?view=details&query=GO:0005515)  [ATP binding](http://amigo.geneontology.org/cgi-bin/amigo/go.cgi?view=details&query=GO:0005524)  [nucleus](http://amigo.geneontology.org/cgi-bin/amigo/go.cgi?view=details&query=GO:0005634)  [cytoplasm](http://amigo.geneontology.org/cgi-bin/amigo/go.cgi?view=details&query=GO:0005737)  [regulation of transcription, DNA-dependent](http://amigo.geneontology.org/cgi-bin/amigo/go.cgi?view=details&query=GO:0006355)  [transcription from RNA polymerase II promoter](http://amigo.geneontology.org/cgi-bin/amigo/go.cgi?view=details&query=GO:0006366)  [induction of apoptosis](http://amigo.geneontology.org/cgi-bin/amigo/go.cgi?view=details&query=GO:0006917)  [cell proliferation](http://amigo.geneontology.org/cgi-bin/amigo/go.cgi?view=details&query=GO:0008283)  [negative regulation of cell cycle](http://amigo.geneontology.org/cgi-bin/amigo/go.cgi?view=details&query=GO:0045786)  [negative regulation of epithelial cell proliferation](http://amigo.geneontology.org/cgi-bin/amigo/go.cgi?view=details&query=GO:0050680) |  |
| [204301_at](https://www.affymetrix.com/LinkServlet?&probeset=204301_at) | KBTBD11 | kelch repeat and BTB (POZ) domain containing 11 | 8 | 1922043 | [NM_014867](http://www.ncbi.nlm.nih.gov/entrez/query.fcgi?cmd=search&db=nucleotide&term=NM_014867%5BACCN%5D&doptcmdl=GenBank) | [9920](http://www.ncbi.nlm.nih.gov/sites/entrez?Db=gene&Cmd=DetailsSearch&Term=9920) | [8p23.3](http://www.ncbi.nlm.nih.gov/mapview/map_search.cgi?direct=on&idtype=gene&id=9920) | [Hs.5333](http://www.ncbi.nlm.nih.gov/UniGene/clust.cgi?ORG=Hs&CID=5333) | [4](http://www.ncbi.nih.gov/entrez/query.fcgi?tool=bioconductor&cmd=Retrieve&db=PubMed&list_uids=9314494%2C9872452%2C14508709%2C15592455) |  |  |
| [204417_at](https://www.affymetrix.com/LinkServlet?&probeset=204417_at) | GALC | galactosylceramidase | 14 | -88429657, -88399359 | [NM_000153](http://www.ncbi.nlm.nih.gov/entrez/query.fcgi?cmd=search&db=nucleotide&term=NM_000153%5BACCN%5D&doptcmdl=GenBank) | [2581](http://www.ncbi.nlm.nih.gov/sites/entrez?Db=gene&Cmd=DetailsSearch&Term=2581) | [14q31](http://www.ncbi.nlm.nih.gov/mapview/map_search.cgi?direct=on&idtype=gene&id=2581) | [Hs.513439](http://www.ncbi.nlm.nih.gov/UniGene/clust.cgi?ORG=Hs&CID=513439) | [33](http://www.ncbi.nih.gov/entrez/query.fcgi?tool=bioconductor&cmd=Retrieve&db=PubMed&list_uids=3278379%2C6811701%2C7417782%2C7581365%2C7601472%2C8162701%2C8281145%2C8297359%2C8399327%2C8595408%2C8786069%2C8889548%2C8940268%2C9272171%2C9338580%2C9434153%2C10234611%2C10477434%2C10833326%2C11814461%2C12477932%2C15489334%2C15657896%2C16344560%2C16607461%2C17458901%2C17579360%2C20301416%2C20410102%2C20800603%2C20877624%2C20886637%2C21102463) | [galactosylceramidase activity](http://amigo.geneontology.org/cgi-bin/amigo/go.cgi?view=details&query=GO:0004336)  [lysosome](http://amigo.geneontology.org/cgi-bin/amigo/go.cgi?view=details&query=GO:0005764)  [carbohydrate metabolic process](http://amigo.geneontology.org/cgi-bin/amigo/go.cgi?view=details&query=GO:0005975)  [galactosylceramide catabolic process](http://amigo.geneontology.org/cgi-bin/amigo/go.cgi?view=details&query=GO:0006683)  [hydrolase activity, acting on glycosyl bonds](http://amigo.geneontology.org/cgi-bin/amigo/go.cgi?view=details&query=GO:0016798)  [cation binding](http://amigo.geneontology.org/cgi-bin/amigo/go.cgi?view=details&query=GO:0043169) | [Sphingolipid metabolism](http://www.genome.ad.jp/dbget-bin/show_pathway?MAP00600+3.2.1.46)  [Metabolic pathways](http://www.genome.ad.jp/dbget-bin/show_pathway?MAP01100+3.2.1.46)  [Lysosome](http://www.genome.ad.jp/dbget-bin/show_pathway?MAP04142+3.2.1.46) |
| [204604_at](https://www.affymetrix.com/LinkServlet?&probeset=204604_at) | CDK14 | cyclin-dependent kinase 14 | 7 | 90338711 | [NM_012395](http://www.ncbi.nlm.nih.gov/entrez/query.fcgi?cmd=search&db=nucleotide&term=NM_012395%5BACCN%5D&doptcmdl=GenBank) | [5218](http://www.ncbi.nlm.nih.gov/sites/entrez?Db=gene&Cmd=DetailsSearch&Term=5218) | [7q21-q22](http://www.ncbi.nlm.nih.gov/mapview/map_search.cgi?direct=on&idtype=gene&id=5218) | [Hs.258576](http://www.ncbi.nlm.nih.gov/UniGene/clust.cgi?ORG=Hs&CID=258576) | [17](http://www.ncbi.nih.gov/entrez/query.fcgi?tool=bioconductor&cmd=Retrieve&db=PubMed&list_uids=9202329%2C10048485%2C11313143%2C12098780%2C12477932%2C12690205%2C12853948%2C15761153%2C16775625%2C17353931%2C17517622%2C17567994%2C18029348%2C19058789%2C19524571%2C19884882%2C20379614) | [G2/M transition of mitotic cell cycle](http://amigo.geneontology.org/cgi-bin/amigo/go.cgi?view=details&query=GO:0000086)  [nucleotide binding](http://amigo.geneontology.org/cgi-bin/amigo/go.cgi?view=details&query=GO:0000166)  [cytoplasmic cyclin-dependent protein kinase holoenzyme complex](http://amigo.geneontology.org/cgi-bin/amigo/go.cgi?view=details&query=GO:0000308)  [cyclin-dependent protein kinase activity](http://amigo.geneontology.org/cgi-bin/amigo/go.cgi?view=details&query=GO:0004693)  [protein binding](http://amigo.geneontology.org/cgi-bin/amigo/go.cgi?view=details&query=GO:0005515)  [ATP binding](http://amigo.geneontology.org/cgi-bin/amigo/go.cgi?view=details&query=GO:0005524)  [nucleus](http://amigo.geneontology.org/cgi-bin/amigo/go.cgi?view=details&query=GO:0005634)  [cytoplasm](http://amigo.geneontology.org/cgi-bin/amigo/go.cgi?view=details&query=GO:0005737)  [cytosol](http://amigo.geneontology.org/cgi-bin/amigo/go.cgi?view=details&query=GO:0005829)  [plasma membrane](http://amigo.geneontology.org/cgi-bin/amigo/go.cgi?view=details&query=GO:0005886)  [protein phosphorylation](http://amigo.geneontology.org/cgi-bin/amigo/go.cgi?view=details&query=GO:0006468)  [cell cycle](http://amigo.geneontology.org/cgi-bin/amigo/go.cgi?view=details&query=GO:0007049)  [Wnt receptor signaling pathway](http://amigo.geneontology.org/cgi-bin/amigo/go.cgi?view=details&query=GO:0016055)  [transferase activity](http://amigo.geneontology.org/cgi-bin/amigo/go.cgi?view=details&query=GO:0016740)  [cyclin binding](http://amigo.geneontology.org/cgi-bin/amigo/go.cgi?view=details&query=GO:0030332)  [cell division](http://amigo.geneontology.org/cgi-bin/amigo/go.cgi?view=details&query=GO:0051301)  [regulation of canonical Wnt receptor signaling pathway](http://amigo.geneontology.org/cgi-bin/amigo/go.cgi?view=details&query=GO:0060828) |  |
| [204678_s_at](https://www.affymetrix.com/LinkServlet?&probeset=204678_s_at) | KCNK1 | potassium channel, subfamily K, member 1 | 1 | 233749749 | [U90065](http://www.ncbi.nlm.nih.gov/entrez/query.fcgi?cmd=search&db=nucleotide&term=U90065%5BACCN%5D&doptcmdl=GenBank) | [3775](http://www.ncbi.nlm.nih.gov/sites/entrez?Db=gene&Cmd=DetailsSearch&Term=3775) | [1q42-q43](http://www.ncbi.nlm.nih.gov/mapview/map_search.cgi?direct=on&idtype=gene&id=3775) | [Hs.208544](http://www.ncbi.nlm.nih.gov/UniGene/clust.cgi?ORG=Hs&CID=208544) | [18](http://www.ncbi.nih.gov/entrez/query.fcgi?tool=bioconductor&cmd=Retrieve&db=PubMed&list_uids=8605869%2C8661042%2C8978667%2C9362344%2C9462864%2C9832487%2C11053038%2C11165377%2C11256078%2C12477932%2C12855359%2C14702039%2C15489334%2C15820677%2C16382106%2C19571146%2C19948975%2C19959478) | [inward rectifier potassium channel activity](http://amigo.geneontology.org/cgi-bin/amigo/go.cgi?view=details&query=GO:0005242)  [voltage-gated ion channel activity](http://amigo.geneontology.org/cgi-bin/amigo/go.cgi?view=details&query=GO:0005244)  [potassium channel activity](http://amigo.geneontology.org/cgi-bin/amigo/go.cgi?view=details&query=GO:0005267)  [ion transport](http://amigo.geneontology.org/cgi-bin/amigo/go.cgi?view=details&query=GO:0006811)  [potassium ion transport](http://amigo.geneontology.org/cgi-bin/amigo/go.cgi?view=details&query=GO:0006813)  [voltage-gated potassium channel complex](http://amigo.geneontology.org/cgi-bin/amigo/go.cgi?view=details&query=GO:0008076)  [membrane](http://amigo.geneontology.org/cgi-bin/amigo/go.cgi?view=details&query=GO:0016020)  [integral to membrane](http://amigo.geneontology.org/cgi-bin/amigo/go.cgi?view=details&query=GO:0016021) |  |
| [204679_at](https://www.affymetrix.com/LinkServlet?&probeset=204679_at) | KCNK1 | potassium channel, subfamily K, member 1 | 1 | 233749749 | [NM_002245](http://www.ncbi.nlm.nih.gov/entrez/query.fcgi?cmd=search&db=nucleotide&term=NM_002245%5BACCN%5D&doptcmdl=GenBank) | [3775](http://www.ncbi.nlm.nih.gov/sites/entrez?Db=gene&Cmd=DetailsSearch&Term=3775) | [1q42-q43](http://www.ncbi.nlm.nih.gov/mapview/map_search.cgi?direct=on&idtype=gene&id=3775) | [Hs.208544](http://www.ncbi.nlm.nih.gov/UniGene/clust.cgi?ORG=Hs&CID=208544) | [18](http://www.ncbi.nih.gov/entrez/query.fcgi?tool=bioconductor&cmd=Retrieve&db=PubMed&list_uids=8605869%2C8661042%2C8978667%2C9362344%2C9462864%2C9832487%2C11053038%2C11165377%2C11256078%2C12477932%2C12855359%2C14702039%2C15489334%2C15820677%2C16382106%2C19571146%2C19948975%2C19959478) | [inward rectifier potassium channel activity](http://amigo.geneontology.org/cgi-bin/amigo/go.cgi?view=details&query=GO:0005242)  [voltage-gated ion channel activity](http://amigo.geneontology.org/cgi-bin/amigo/go.cgi?view=details&query=GO:0005244)  [potassium channel activity](http://amigo.geneontology.org/cgi-bin/amigo/go.cgi?view=details&query=GO:0005267)  [ion transport](http://amigo.geneontology.org/cgi-bin/amigo/go.cgi?view=details&query=GO:0006811)  [potassium ion transport](http://amigo.geneontology.org/cgi-bin/amigo/go.cgi?view=details&query=GO:0006813)  [voltage-gated potassium channel complex](http://amigo.geneontology.org/cgi-bin/amigo/go.cgi?view=details&query=GO:0008076)  [membrane](http://amigo.geneontology.org/cgi-bin/amigo/go.cgi?view=details&query=GO:0016020)  [integral to membrane](http://amigo.geneontology.org/cgi-bin/amigo/go.cgi?view=details&query=GO:0016021) |  |
| [204832_s_at](https://www.affymetrix.com/LinkServlet?&probeset=204832_s_at) | BMPR1A | bone morphogenetic protein receptor, type IA | 10 | 88516395 | [NM_004329](http://www.ncbi.nlm.nih.gov/entrez/query.fcgi?cmd=search&db=nucleotide&term=NM_004329%5BACCN%5D&doptcmdl=GenBank) | [657](http://www.ncbi.nlm.nih.gov/sites/entrez?Db=gene&Cmd=DetailsSearch&Term=657) | [10q22.3](http://www.ncbi.nlm.nih.gov/mapview/map_search.cgi?direct=on&idtype=gene&id=657) | [Hs.524477](http://www.ncbi.nlm.nih.gov/UniGene/clust.cgi?ORG=Hs&CID=524477) | [87](http://www.ncbi.nih.gov/entrez/query.fcgi?tool=bioconductor&cmd=Retrieve&db=PubMed&list_uids=7644468%2C7791754%2C8006002%2C8397373%2C8592941%2C8605097%2C8702914%2C8707881%2C9389648%2C9525338%2C9547239%2C9663660%2C9730621%2C9738003%2C9950587%2C10051328%2C10504300%2C10692589%2C10712517%2C10850425%2C10880444%2C10881198%2C11139569%2C11241215%2C11263668%2C11278302%2C11282024%2C11381269%2C11401330%2C11438941%2C11536076%2C11580864%2C12065756%2C12135884%2C12136244%2C12417513%2C12477932%2C12620973%2C12630959%2C15064755%2C15148321%2C15235019%2C15252450%2C15351706%2C15489334%2C15621726%2C15657086%2C15940369%2C16226113%2C16341674%2C16385451%2C16436528%2C16436638%2C16525031%2C16613856%2C16672363%2C16886151%2C17081983%2C17101085%2C17356069%2C17513295%2C17573831%2C17624341%2C17873119%2C18160401%2C18178612%2C18184661%2C18262054%2C18436533%2C18510548%2C18667463%2C18823382%2C18937504%2C19244313%2C19438883%2C19453261%2C19463221%2C19502417%2C19773747%2C20301642%2C20346360%2C20587070%2C20634891%2C20693682%2C20734064%2C20927405%2C21152263) | [nucleotide binding](http://amigo.geneontology.org/cgi-bin/amigo/go.cgi?view=details&query=GO:0000166)  [protein serine/threonine kinase activity](http://amigo.geneontology.org/cgi-bin/amigo/go.cgi?view=details&query=GO:0004674)  [receptor activity](http://amigo.geneontology.org/cgi-bin/amigo/go.cgi?view=details&query=GO:0004872)  [transforming growth factor beta receptor activity](http://amigo.geneontology.org/cgi-bin/amigo/go.cgi?view=details&query=GO:0005024)  [protein binding](http://amigo.geneontology.org/cgi-bin/amigo/go.cgi?view=details&query=GO:0005515)  [ATP binding](http://amigo.geneontology.org/cgi-bin/amigo/go.cgi?view=details&query=GO:0005524)  [plasma membrane](http://amigo.geneontology.org/cgi-bin/amigo/go.cgi?view=details&query=GO:0005886)  [plasma membrane](http://amigo.geneontology.org/cgi-bin/amigo/go.cgi?view=details&query=GO:0005886)  [plasma membrane](http://amigo.geneontology.org/cgi-bin/amigo/go.cgi?view=details&query=GO:0005886)  [caveola](http://amigo.geneontology.org/cgi-bin/amigo/go.cgi?view=details&query=GO:0005901)  [protein phosphorylation](http://amigo.geneontology.org/cgi-bin/amigo/go.cgi?view=details&query=GO:0006468)  [immune response](http://amigo.geneontology.org/cgi-bin/amigo/go.cgi?view=details&query=GO:0006955)  [transforming growth factor beta receptor signaling pathway](http://amigo.geneontology.org/cgi-bin/amigo/go.cgi?view=details&query=GO:0007179)  [positive regulation of pathway-restricted SMAD protein phosphorylation](http://amigo.geneontology.org/cgi-bin/amigo/go.cgi?view=details&query=GO:0010862)  [integral to membrane](http://amigo.geneontology.org/cgi-bin/amigo/go.cgi?view=details&query=GO:0016021)  [positive regulation of bone mineralization](http://amigo.geneontology.org/cgi-bin/amigo/go.cgi?view=details&query=GO:0030501)  [BMP signaling pathway](http://amigo.geneontology.org/cgi-bin/amigo/go.cgi?view=details&query=GO:0030509)  [BMP signaling pathway](http://amigo.geneontology.org/cgi-bin/amigo/go.cgi?view=details&query=GO:0030509)  [protein homodimerization activity](http://amigo.geneontology.org/cgi-bin/amigo/go.cgi?view=details&query=GO:0042803)  [positive regulation of osteoblast differentiation](http://amigo.geneontology.org/cgi-bin/amigo/go.cgi?view=details&query=GO:0045669)  [SMAD binding](http://amigo.geneontology.org/cgi-bin/amigo/go.cgi?view=details&query=GO:0046332)  [metal ion binding](http://amigo.geneontology.org/cgi-bin/amigo/go.cgi?view=details&query=GO:0046872)  [positive regulation of SMAD protein import into nucleus](http://amigo.geneontology.org/cgi-bin/amigo/go.cgi?view=details&query=GO:0060391) | [Cytokine-cytokine receptor interaction](http://www.genome.ad.jp/dbget-bin/show_pathway?MAP04060+2.7.11.30)  [TGF-beta signaling pathway](http://www.genome.ad.jp/dbget-bin/show_pathway?MAP04350+2.7.11.30) |
| [205160_at](https://www.affymetrix.com/LinkServlet?&probeset=205160_at) | PEX11A | peroxisomal biogenesis factor 11 alpha | 15 | -90226288 | [AL360141](http://www.ncbi.nlm.nih.gov/entrez/query.fcgi?cmd=search&db=nucleotide&term=AL360141%5BACCN%5D&doptcmdl=GenBank) | [8800](http://www.ncbi.nlm.nih.gov/sites/entrez?Db=gene&Cmd=DetailsSearch&Term=8800) | [15q26.1](http://www.ncbi.nlm.nih.gov/mapview/map_search.cgi?direct=on&idtype=gene&id=8800) | [Hs.31034](http://www.ncbi.nlm.nih.gov/UniGene/clust.cgi?ORG=Hs&CID=31034) | [10](http://www.ncbi.nih.gov/entrez/query.fcgi?tool=bioconductor&cmd=Retrieve&db=PubMed&list_uids=9714566%2C9792670%2C9922452%2C10704444%2C12477932%2C14702039%2C15489334%2C16567422%2C17220199%2C20826455) | [peroxisome](http://amigo.geneontology.org/cgi-bin/amigo/go.cgi?view=details&query=GO:0005777)  [peroxisomal membrane](http://amigo.geneontology.org/cgi-bin/amigo/go.cgi?view=details&query=GO:0005778)  [integral to peroxisomal membrane](http://amigo.geneontology.org/cgi-bin/amigo/go.cgi?view=details&query=GO:0005779)  [peroxisome organization](http://amigo.geneontology.org/cgi-bin/amigo/go.cgi?view=details&query=GO:0007031)  [signal transduction](http://amigo.geneontology.org/cgi-bin/amigo/go.cgi?view=details&query=GO:0007165)  [membrane](http://amigo.geneontology.org/cgi-bin/amigo/go.cgi?view=details&query=GO:0016020)  [integral to membrane](http://amigo.geneontology.org/cgi-bin/amigo/go.cgi?view=details&query=GO:0016021)  [peroxisome membrane biogenesis](http://amigo.geneontology.org/cgi-bin/amigo/go.cgi?view=details&query=GO:0016557)  [peroxisome fission](http://amigo.geneontology.org/cgi-bin/amigo/go.cgi?view=details&query=GO:0016559)  [cellular lipid metabolic process](http://amigo.geneontology.org/cgi-bin/amigo/go.cgi?view=details&query=GO:0044255)  [brown fat cell differentiation](http://amigo.geneontology.org/cgi-bin/amigo/go.cgi?view=details&query=GO:0050873) | [Peroxisome](http://www.genome.ad.jp/kegg/pathway/hsa/hsa04146.html) |
| [205413_at](https://www.affymetrix.com/LinkServlet?&probeset=205413_at) | MPPED2 | metallophosphoesterase domain containing 2 | 11 | -30431618, -30406039 | [NM_001584](http://www.ncbi.nlm.nih.gov/entrez/query.fcgi?cmd=search&db=nucleotide&term=NM_001584%5BACCN%5D&doptcmdl=GenBank) | [744](http://www.ncbi.nlm.nih.gov/sites/entrez?Db=gene&Cmd=DetailsSearch&Term=744) | [11p13](http://www.ncbi.nlm.nih.gov/mapview/map_search.cgi?direct=on&idtype=gene&id=744) | [Hs.289795](http://www.ncbi.nlm.nih.gov/UniGene/clust.cgi?ORG=Hs&CID=289795) | [10](http://www.ncbi.nih.gov/entrez/query.fcgi?tool=bioconductor&cmd=Retrieve&db=PubMed&list_uids=7527372%2C8666403%2C8889549%2C9266672%2C12477932%2C15489334%2C16344560%2C17207965%2C19240061%2C20379614) | [nervous system development](http://amigo.geneontology.org/cgi-bin/amigo/go.cgi?view=details&query=GO:0007399)  [hydrolase activity](http://amigo.geneontology.org/cgi-bin/amigo/go.cgi?view=details&query=GO:0016787)  [metal ion binding](http://amigo.geneontology.org/cgi-bin/amigo/go.cgi?view=details&query=GO:0046872) |  |
| [205414_s_at](https://www.affymetrix.com/LinkServlet?&probeset=205414_s_at) | ARHGAP44 | Rho GTPase activating protein 44 | 17 | 12692828 | [NM_014859](http://www.ncbi.nlm.nih.gov/entrez/query.fcgi?cmd=search&db=nucleotide&term=NM_014859%5BACCN%5D&doptcmdl=GenBank) | [9912](http://www.ncbi.nlm.nih.gov/sites/entrez?Db=gene&Cmd=DetailsSearch&Term=9912) | [17p12](http://www.ncbi.nlm.nih.gov/mapview/map_search.cgi?direct=on&idtype=gene&id=9912) | [Hs.499758](http://www.ncbi.nlm.nih.gov/UniGene/clust.cgi?ORG=Hs&CID=499758) | [8](http://www.ncbi.nih.gov/entrez/query.fcgi?tool=bioconductor&cmd=Retrieve&db=PubMed&list_uids=9734811%2C11431473%2C12477932%2C14702039%2C15489334%2C16169070%2C16344560%2C19273615) | [GTPase activator activity](http://amigo.geneontology.org/cgi-bin/amigo/go.cgi?view=details&query=GO:0005096)  [intracellular](http://amigo.geneontology.org/cgi-bin/amigo/go.cgi?view=details&query=GO:0005622)  [cytoplasm](http://amigo.geneontology.org/cgi-bin/amigo/go.cgi?view=details&query=GO:0005737)  [cytosol](http://amigo.geneontology.org/cgi-bin/amigo/go.cgi?view=details&query=GO:0005829)  [signal transduction](http://amigo.geneontology.org/cgi-bin/amigo/go.cgi?view=details&query=GO:0007165)  [small GTPase mediated signal transduction](http://amigo.geneontology.org/cgi-bin/amigo/go.cgi?view=details&query=GO:0007264)  [regulation of small GTPase mediated signal transduction](http://amigo.geneontology.org/cgi-bin/amigo/go.cgi?view=details&query=GO:0051056) |  |
| [205794_s_at](https://www.affymetrix.com/LinkServlet?&probeset=205794_s_at) | NOVA1 | neuro-oncological ventral antigen 1 | 14 | -26939324, -26915089 | [NM_002515](http://www.ncbi.nlm.nih.gov/entrez/query.fcgi?cmd=search&db=nucleotide&term=NM_002515%5BACCN%5D&doptcmdl=GenBank) | [4857](http://www.ncbi.nlm.nih.gov/sites/entrez?Db=gene&Cmd=DetailsSearch&Term=4857) | [14q](http://www.ncbi.nlm.nih.gov/mapview/map_search.cgi?direct=on&idtype=gene&id=4857) | [Hs.31588](http://www.ncbi.nlm.nih.gov/UniGene/clust.cgi?ORG=Hs&CID=31588) | [18](http://www.ncbi.nih.gov/entrez/query.fcgi?tool=bioconductor&cmd=Retrieve&db=PubMed&list_uids=8398153%2C8558240%2C9154818%2C10368286%2C10719891%2C10737800%2C12477932%2C12808107%2C15146197%2C15489334%2C16041372%2C16169070%2C16713569%2C18029348%2C18218628%2C18822086%2C19584346%2C19680635) | [nuclear mRNA splicing, via spliceosome](http://amigo.geneontology.org/cgi-bin/amigo/go.cgi?view=details&query=GO:0000398)  [RNA binding](http://amigo.geneontology.org/cgi-bin/amigo/go.cgi?view=details&query=GO:0003723)  [mRNA binding](http://amigo.geneontology.org/cgi-bin/amigo/go.cgi?view=details&query=GO:0003729)  [nucleus](http://amigo.geneontology.org/cgi-bin/amigo/go.cgi?view=details&query=GO:0005634)  [RNA processing](http://amigo.geneontology.org/cgi-bin/amigo/go.cgi?view=details&query=GO:0006396)  [synaptic transmission](http://amigo.geneontology.org/cgi-bin/amigo/go.cgi?view=details&query=GO:0007268)  [locomotory behavior](http://amigo.geneontology.org/cgi-bin/amigo/go.cgi?view=details&query=GO:0007626)  [RNA splicing](http://amigo.geneontology.org/cgi-bin/amigo/go.cgi?view=details&query=GO:0008380) |  |
| [205831_at](https://www.affymetrix.com/LinkServlet?&probeset=205831_at) | CD2 | CD2 molecule | 1 | 117297085 | [NM_001767](http://www.ncbi.nlm.nih.gov/entrez/query.fcgi?cmd=search&db=nucleotide&term=NM_001767%5BACCN%5D&doptcmdl=GenBank) | [914](http://www.ncbi.nlm.nih.gov/sites/entrez?Db=gene&Cmd=DetailsSearch&Term=914) | [1p13.1](http://www.ncbi.nlm.nih.gov/mapview/map_search.cgi?direct=on&idtype=gene&id=914) | [Hs.523500](http://www.ncbi.nlm.nih.gov/UniGene/clust.cgi?ORG=Hs&CID=523500) | [77](http://www.ncbi.nih.gov/entrez/query.fcgi?tool=bioconductor&cmd=Retrieve&db=PubMed&list_uids=1346273%2C1351089%2C1377404%2C1385321%2C1832084%2C1970422%2C1976695%2C2111780%2C2437578%2C2444890%2C2471997%2C2883656%2C2894031%2C2901953%2C3490670%2C7517794%2C7539755%2C7544493%2C7589092%2C7686927%2C7915183%2C7933095%2C7994575%2C8125298%2C8551220%2C8809126%2C8977276%2C9270771%2C9373149%2C9475352%2C9677430%2C9741631%2C9743208%2C9843987%2C9857189%2C10380930%2C10404223%2C10510361%2C10575274%2C10642604%2C10722370%2C11090067%2C11152963%2C11376005%2C11544295%2C11575926%2C11591762%2C11602341%2C11932928%2C12032326%2C12356317%2C12369898%2C12426371%2C12477932%2C12530983%2C12618476%2C12690097%2C12714509%2C12731040%2C15489334%2C15528362%2C16000308%2C16049493%2C16710414%2C16803907%2C17085486%2C17168569%2C18318997%2C18431797%2C19109405%2C19237575%2C19494291%2C19502238%2C19586919%2C19898481%2C20813844%2C21048031) | [membrane raft polarization](http://amigo.geneontology.org/cgi-bin/amigo/go.cgi?view=details&query=GO:0001766)  [receptor activity](http://amigo.geneontology.org/cgi-bin/amigo/go.cgi?view=details&query=GO:0004872)  [protein binding](http://amigo.geneontology.org/cgi-bin/amigo/go.cgi?view=details&query=GO:0005515)  [extracellular region](http://amigo.geneontology.org/cgi-bin/amigo/go.cgi?view=details&query=GO:0005576)  [plasma membrane](http://amigo.geneontology.org/cgi-bin/amigo/go.cgi?view=details&query=GO:0005886)  [integral to plasma membrane](http://amigo.geneontology.org/cgi-bin/amigo/go.cgi?view=details&query=GO:0005887)  [induction of apoptosis](http://amigo.geneontology.org/cgi-bin/amigo/go.cgi?view=details&query=GO:0006917)  [cell surface receptor linked signaling pathway](http://amigo.geneontology.org/cgi-bin/amigo/go.cgi?view=details&query=GO:0007166)  [blood coagulation](http://amigo.geneontology.org/cgi-bin/amigo/go.cgi?view=details&query=GO:0007596)  [external side of plasma membrane](http://amigo.geneontology.org/cgi-bin/amigo/go.cgi?view=details&query=GO:0009897)  [internal side of plasma membrane](http://amigo.geneontology.org/cgi-bin/amigo/go.cgi?view=details&query=GO:0009898)  [cell-cell adhesion](http://amigo.geneontology.org/cgi-bin/amigo/go.cgi?view=details&query=GO:0016337)  [natural killer cell activation](http://amigo.geneontology.org/cgi-bin/amigo/go.cgi?view=details&query=GO:0030101)  [positive regulation of myeloid dendritic cell activation](http://amigo.geneontology.org/cgi-bin/amigo/go.cgi?view=details&query=GO:0030887)  [T cell activation](http://amigo.geneontology.org/cgi-bin/amigo/go.cgi?view=details&query=GO:0042110)  [protein homodimerization activity](http://amigo.geneontology.org/cgi-bin/amigo/go.cgi?view=details&query=GO:0042803)  [eukaryotic cell surface binding](http://amigo.geneontology.org/cgi-bin/amigo/go.cgi?view=details&query=GO:0043499)  [regulation of T cell differentiation](http://amigo.geneontology.org/cgi-bin/amigo/go.cgi?view=details&query=GO:0045580)  [anchored to plasma membrane](http://amigo.geneontology.org/cgi-bin/amigo/go.cgi?view=details&query=GO:0046658)  [leukocyte migration](http://amigo.geneontology.org/cgi-bin/amigo/go.cgi?view=details&query=GO:0050900) | [Cell adhesion molecules (CAMs)](http://www.genome.ad.jp/kegg/pathway/hsa/hsa04514.html)  [Hematopoietic cell lineage](http://www.genome.ad.jp/kegg/pathway/hsa/hsa04640.html) |
| [205992_s_at](https://www.affymetrix.com/LinkServlet?&probeset=205992_s_at) | IL15 | interleukin 15 | 4 | 142557753 | [NM_000585](http://www.ncbi.nlm.nih.gov/entrez/query.fcgi?cmd=search&db=nucleotide&term=NM_000585%5BACCN%5D&doptcmdl=GenBank) | [3600](http://www.ncbi.nlm.nih.gov/sites/entrez?Db=gene&Cmd=DetailsSearch&Term=3600) | [4q31](http://www.ncbi.nlm.nih.gov/mapview/map_search.cgi?direct=on&idtype=gene&id=3600) | [Hs.602618](http://www.ncbi.nlm.nih.gov/UniGene/clust.cgi?ORG=Hs&CID=602618) [Hs.654378](http://www.ncbi.nlm.nih.gov/UniGene/clust.cgi?ORG=Hs&CID=654378) | [226](http://www.ncbi.nih.gov/entrez/query.fcgi?tool=bioconductor&cmd=Retrieve&db=PubMed&list_uids=7568001%2C7641685%2C7759105%2C8026467%2C8178155%2C8568232%2C8668345%2C8932977%2C8977197%2C9176231%2C9405632%2C10388525%2C10784451%2C10851076%2C10869346%2C11122241%2C11134140%2C11134366%2C11168804%2C11287118%2C11369758%2C11581322%2C11680634%2C11702064%2C11746271%2C11751999%2C11807773%2C11814312%2C11830476%2C11876762%2C11919076%2C11972069%2C12089333%2C12089714%2C12114302%2C12115611%2C12147628%2C12165497%2C12237122%2C12297341%2C12379638%2C12391205%2C12393488%2C12401478%2C12421959%2C12453470%2C12477932%2C12542496%2C12571184%2C12598649%2C12623850%2C12734346%2C12747456%2C12757260%2C12759422%2C12805064%2C12826231%2C12847234%2C12857602%2C12865812%2C12960322%2C14499665%2C14581351%2C14607929%2C14607946%2C14617758%2C14648805%2C14662842%2C14684414%2C14702039%2C14718574%2C14762166%2C14967307%2C14982947%2C14984938%2C15034035%2C15039446%2C15131572%2C15225634%2C15284244%2C15328153%2C15347678%2C15353479%2C15358647%2C15489334%2C15531573%2C15536127%2C15557159%2C15563472%2C15598425%2C15710591%2C15713701%2C15770395%2C15778359%2C15943034%2C15976176%2C15993713%2C16024007%2C16060678%2C16098919%2C16109314%2C16136475%2C16148110%2C16273100%2C16284400%2C16333313%2C16427155%2C16474399%2C16482511%2C16507118%2C16606671%2C16629787%2C16670327%2C16772281%2C16791279%2C17001647%2C17077296%2C17110377%2C17142786%2C17158609%2C17324400%2C17363780%2C17537415%2C17554368%2C17616399%2C17635814%2C17655329%2C17703412%2C17938255%2C17947730%2C17964972%2C17982063%2C17986095%2C18086532%2C18275895%2C18280748%2C18295891%2C18311795%2C18348982%2C18365236%2C18419254%2C18424079%2C18431516%2C18482208%2C18493613%2C18493981%2C18505820%2C18509087%2C18514540%2C18523245%2C18524812%2C18580966%2C18583567%2C18633131%2C18656487%2C18676680%2C18697873%2C18706446%2C18782269%2C18818748%2C18840707%2C18981132%2C19019335%2C19047106%2C19088135%2C19133918%2C19170196%2C19176441%2C19188921%2C19233474%2C19237603%2C19240061%2C19247692%2C19258923%2C19325034%2C19406127%2C19414768%2C19414770%2C19414780%2C19505916%2C19527514%2C19546878%2C19570824%2C19573080%2C19592095%2C19593212%2C19625176%2C19657406%2C19692168%2C19696432%2C19703339%2C19710458%2C19756346%2C19773279%2C19796965%2C19835475%2C19913121%2C19949092%2C20007590%2C20043070%2C20083555%2C20140262%2C20142427%2C20153259%2C20166880%2C20190192%2C20237496%2C20331472%2C20381491%2C20414345%2C20430958%2C20444155%2C20453000%2C20483740%2C20503287%2C20538758%2C20568250%2C20622171%2C20628086%2C20631381%2C20670353%2C20671116%2C20732850%2C20736143%2C21048031%2C21049047) | [NK T cell proliferation](http://amigo.geneontology.org/cgi-bin/amigo/go.cgi?view=details&query=GO:0001866)  [signal transducer activity](http://amigo.geneontology.org/cgi-bin/amigo/go.cgi?view=details&query=GO:0004871)  [cytokine activity](http://amigo.geneontology.org/cgi-bin/amigo/go.cgi?view=details&query=GO:0005125)  [cytokine receptor binding](http://amigo.geneontology.org/cgi-bin/amigo/go.cgi?view=details&query=GO:0005126)  [protein binding](http://amigo.geneontology.org/cgi-bin/amigo/go.cgi?view=details&query=GO:0005515)  [extracellular region](http://amigo.geneontology.org/cgi-bin/amigo/go.cgi?view=details&query=GO:0005576)  [extracellular space](http://amigo.geneontology.org/cgi-bin/amigo/go.cgi?view=details&query=GO:0005615)  [membrane fraction](http://amigo.geneontology.org/cgi-bin/amigo/go.cgi?view=details&query=GO:0005624)  [nucleus](http://amigo.geneontology.org/cgi-bin/amigo/go.cgi?view=details&query=GO:0005634)  [cytoplasm](http://amigo.geneontology.org/cgi-bin/amigo/go.cgi?view=details&query=GO:0005737)  [endosome](http://amigo.geneontology.org/cgi-bin/amigo/go.cgi?view=details&query=GO:0005768)  [Golgi apparatus](http://amigo.geneontology.org/cgi-bin/amigo/go.cgi?view=details&query=GO:0005794)  [integral to plasma membrane](http://amigo.geneontology.org/cgi-bin/amigo/go.cgi?view=details&query=GO:0005887)  [immune response](http://amigo.geneontology.org/cgi-bin/amigo/go.cgi?view=details&query=GO:0006955)  [signal transduction](http://amigo.geneontology.org/cgi-bin/amigo/go.cgi?view=details&query=GO:0007165)  [cell-cell signaling](http://amigo.geneontology.org/cgi-bin/amigo/go.cgi?view=details&query=GO:0007267)  [aging](http://amigo.geneontology.org/cgi-bin/amigo/go.cgi?view=details&query=GO:0007568)  [positive regulation of cell proliferation](http://amigo.geneontology.org/cgi-bin/amigo/go.cgi?view=details&query=GO:0008284)  [positive regulation of interleukin-17 production](http://amigo.geneontology.org/cgi-bin/amigo/go.cgi?view=details&query=GO:0032740)  [positive regulation of natural killer cell proliferation](http://amigo.geneontology.org/cgi-bin/amigo/go.cgi?view=details&query=GO:0032819)  [positive regulation of natural killer cell differentiation](http://amigo.geneontology.org/cgi-bin/amigo/go.cgi?view=details&query=GO:0032825)  [positive regulation of tissue remodeling](http://amigo.geneontology.org/cgi-bin/amigo/go.cgi?view=details&query=GO:0034105)  [positive regulation of T cell proliferation](http://amigo.geneontology.org/cgi-bin/amigo/go.cgi?view=details&query=GO:0042102)  [extrathymic T cell selection](http://amigo.geneontology.org/cgi-bin/amigo/go.cgi?view=details&query=GO:0045062)  [regulation of T cell differentiation](http://amigo.geneontology.org/cgi-bin/amigo/go.cgi?view=details&query=GO:0045580)  [lymph node development](http://amigo.geneontology.org/cgi-bin/amigo/go.cgi?view=details&query=GO:0048535)  [negative regulation of smooth muscle cell proliferation](http://amigo.geneontology.org/cgi-bin/amigo/go.cgi?view=details&query=GO:0048662)  [regulation of defense response to virus by host](http://amigo.geneontology.org/cgi-bin/amigo/go.cgi?view=details&query=GO:0050691)  [positive regulation of inflammatory response](http://amigo.geneontology.org/cgi-bin/amigo/go.cgi?view=details&query=GO:0050729)  [positive regulation of immune response](http://amigo.geneontology.org/cgi-bin/amigo/go.cgi?view=details&query=GO:0050778) | [Cytokine-cytokine receptor interaction](http://www.genome.ad.jp/kegg/pathway/hsa/hsa04060.html)  [Jak-STAT signaling pathway](http://www.genome.ad.jp/kegg/pathway/hsa/hsa04630.html)  [Intestinal immune network for IgA production](http://www.genome.ad.jp/kegg/pathway/hsa/hsa04672.html)  [Rheumatoid arthritis](http://www.genome.ad.jp/kegg/pathway/hsa/hsa05323.html) |
| [206255_at](https://www.affymetrix.com/LinkServlet?&probeset=206255_at) | BLK | B lymphoid tyrosine kinase | 8 | 11351520 | [NM_001715](http://www.ncbi.nlm.nih.gov/entrez/query.fcgi?cmd=search&db=nucleotide&term=NM_001715%5BACCN%5D&doptcmdl=GenBank) | [640](http://www.ncbi.nlm.nih.gov/sites/entrez?Db=gene&Cmd=DetailsSearch&Term=640) | [8p23-p22](http://www.ncbi.nlm.nih.gov/mapview/map_search.cgi?direct=on&idtype=gene&id=640) | [Hs.146591](http://www.ncbi.nlm.nih.gov/UniGene/clust.cgi?ORG=Hs&CID=146591) | [37](http://www.ncbi.nih.gov/entrez/query.fcgi?tool=bioconductor&cmd=Retrieve&db=PubMed&list_uids=2404338%2C7592787%2C7592958%2C7822795%2C7845672%2C8083187%2C8395016%2C8621719%2C8636124%2C8756631%2C9177269%2C9525867%2C10449731%2C11896452%2C12406557%2C12477932%2C14970218%2C18204098%2C19165918%2C19180478%2C19225526%2C19351960%2C19503088%2C19644876%2C19667185%2C19740902%2C19796918%2C19838193%2C20130895%2C20131239%2C20131273%2C20156505%2C20453440%2C20881011%2C20962850%2C21068098%2C21225715) | [nucleotide binding](http://amigo.geneontology.org/cgi-bin/amigo/go.cgi?view=details&query=GO:0000166)  [protein tyrosine kinase activity](http://amigo.geneontology.org/cgi-bin/amigo/go.cgi?view=details&query=GO:0004713)  [non-membrane spanning protein tyrosine kinase activity](http://amigo.geneontology.org/cgi-bin/amigo/go.cgi?view=details&query=GO:0004715)  [ATP binding](http://amigo.geneontology.org/cgi-bin/amigo/go.cgi?view=details&query=GO:0005524)  [protein phosphorylation](http://amigo.geneontology.org/cgi-bin/amigo/go.cgi?view=details&query=GO:0006468)  [intracellular protein kinase cascade](http://amigo.geneontology.org/cgi-bin/amigo/go.cgi?view=details&query=GO:0007243)  [positive regulation of insulin secretion](http://amigo.geneontology.org/cgi-bin/amigo/go.cgi?view=details&query=GO:0032024) |  |
| [206283_s_at](https://www.affymetrix.com/LinkServlet?&probeset=206283_s_at) | TAL1 | T-cell acute lymphocytic leukemia 1 | 1 | -47681962 | [NM_003189](http://www.ncbi.nlm.nih.gov/entrez/query.fcgi?cmd=search&db=nucleotide&term=NM_003189%5BACCN%5D&doptcmdl=GenBank) | [6886](http://www.ncbi.nlm.nih.gov/sites/entrez?Db=gene&Cmd=DetailsSearch&Term=6886) | [1p32](http://www.ncbi.nlm.nih.gov/mapview/map_search.cgi?direct=on&idtype=gene&id=6886) | [Hs.705618](http://www.ncbi.nlm.nih.gov/UniGene/clust.cgi?ORG=Hs&CID=705618) | [78](http://www.ncbi.nih.gov/entrez/query.fcgi?tool=bioconductor&cmd=Retrieve&db=PubMed&list_uids=1311214%2C1396592%2C1450410%2C1453013%2C1886719%2C1964581%2C2230650%2C2247063%2C2303035%2C2602361%2C2740341%2C7568177%2C7833471%2C7957052%2C8078932%2C8084606%2C8159721%2C8414504%2C8423803%2C8437851%2C8576241%2C9111058%2C9171354%2C9209374%2C9242638%2C9507011%2C9819382%2C9824680%2C10383400%2C10490830%2C10688671%2C11118214%2C11904294%2C12010791%2C12091340%2C12239153%2C12477932%2C12867998%2C14651981%2C14715640%2C14729962%2C14970264%2C15086455%2C15383276%2C15677454%2C15923636%2C15961517%2C16007160%2C16298389%2C16407974%2C16621969%2C16710414%2C16763211%2C16778171%2C16849639%2C17460775%2C17474147%2C17503961%2C17507663%2C17878155%2C17910069%2C17962192%2C18187418%2C18436863%2C18439091%2C18495761%2C18550854%2C19406989%2C19497860%2C19527627%2C19587703%2C19785037%2C19865112%2C19913121%2C20028976%2C20140202%2C20628086%2C20855495) | [histone deacetylase complex](http://amigo.geneontology.org/cgi-bin/amigo/go.cgi?view=details&query=GO:0000118)  [nuclear chromatin](http://amigo.geneontology.org/cgi-bin/amigo/go.cgi?view=details&query=GO:0000790)  [DNA binding](http://amigo.geneontology.org/cgi-bin/amigo/go.cgi?view=details&query=GO:0003677)  [sequence-specific DNA binding transcription factor activity](http://amigo.geneontology.org/cgi-bin/amigo/go.cgi?view=details&query=GO:0003700)  [protein binding](http://amigo.geneontology.org/cgi-bin/amigo/go.cgi?view=details&query=GO:0005515)  [nucleus](http://amigo.geneontology.org/cgi-bin/amigo/go.cgi?view=details&query=GO:0005634)  [transcription, DNA-dependent](http://amigo.geneontology.org/cgi-bin/amigo/go.cgi?view=details&query=GO:0006351)  [multicellular organismal development](http://amigo.geneontology.org/cgi-bin/amigo/go.cgi?view=details&query=GO:0007275)  [cell proliferation](http://amigo.geneontology.org/cgi-bin/amigo/go.cgi?view=details&query=GO:0008283)  [regulation of gene-specific transcription from RNA polymerase II promoter](http://amigo.geneontology.org/cgi-bin/amigo/go.cgi?view=details&query=GO:0010551)  [positive regulation of gene-specific transcription from RNA polymerase II promoter](http://amigo.geneontology.org/cgi-bin/amigo/go.cgi?view=details&query=GO:0010552)  [promoter binding](http://amigo.geneontology.org/cgi-bin/amigo/go.cgi?view=details&query=GO:0010843)  [transcription activator activity](http://amigo.geneontology.org/cgi-bin/amigo/go.cgi?view=details&query=GO:0016563)  [enzyme binding](http://amigo.geneontology.org/cgi-bin/amigo/go.cgi?view=details&query=GO:0019899)  [hemopoiesis](http://amigo.geneontology.org/cgi-bin/amigo/go.cgi?view=details&query=GO:0030097)  [erythrocyte differentiation](http://amigo.geneontology.org/cgi-bin/amigo/go.cgi?view=details&query=GO:0030218)  [positive regulation of protein complex assembly](http://amigo.geneontology.org/cgi-bin/amigo/go.cgi?view=details&query=GO:0031334)  [Lsd1/2 complex](http://amigo.geneontology.org/cgi-bin/amigo/go.cgi?view=details&query=GO:0033193)  [embryonic hemopoiesis](http://amigo.geneontology.org/cgi-bin/amigo/go.cgi?view=details&query=GO:0035162)  [histone deacetylase binding](http://amigo.geneontology.org/cgi-bin/amigo/go.cgi?view=details&query=GO:0042826)  [cell fate commitment](http://amigo.geneontology.org/cgi-bin/amigo/go.cgi?view=details&query=GO:0045165)  [positive regulation of erythrocyte differentiation](http://amigo.geneontology.org/cgi-bin/amigo/go.cgi?view=details&query=GO:0045648)  [positive regulation of erythrocyte differentiation](http://amigo.geneontology.org/cgi-bin/amigo/go.cgi?view=details&query=GO:0045648)  [positive regulation of chromatin assembly or disassembly](http://amigo.geneontology.org/cgi-bin/amigo/go.cgi?view=details&query=GO:0045799)  [positive regulation of mitotic cell cycle](http://amigo.geneontology.org/cgi-bin/amigo/go.cgi?view=details&query=GO:0045931)  [positive regulation of cell division](http://amigo.geneontology.org/cgi-bin/amigo/go.cgi?view=details&query=GO:0051781)  [E-box binding](http://amigo.geneontology.org/cgi-bin/amigo/go.cgi?view=details&query=GO:0070888) |  |
| [206546_at](https://www.affymetrix.com/LinkServlet?&probeset=206546_at) | SYCP2 | synaptonemal complex protein 2 | 20 | -58438618 | [NM_014258](http://www.ncbi.nlm.nih.gov/entrez/query.fcgi?cmd=search&db=nucleotide&term=NM_014258%5BACCN%5D&doptcmdl=GenBank) | [10388](http://www.ncbi.nlm.nih.gov/sites/entrez?Db=gene&Cmd=DetailsSearch&Term=10388) | [20q13.33](http://www.ncbi.nlm.nih.gov/mapview/map_search.cgi?direct=on&idtype=gene&id=10388) | [Hs.202676](http://www.ncbi.nlm.nih.gov/UniGene/clust.cgi?ORG=Hs&CID=202676) | [5](http://www.ncbi.nih.gov/entrez/query.fcgi?tool=bioconductor&cmd=Retrieve&db=PubMed&list_uids=9592139%2C10341103%2C11780052%2C12477932%2C20378615) | [synaptonemal complex](http://amigo.geneontology.org/cgi-bin/amigo/go.cgi?view=details&query=GO:0000795)  [lateral element](http://amigo.geneontology.org/cgi-bin/amigo/go.cgi?view=details&query=GO:0000800)  [DNA binding](http://amigo.geneontology.org/cgi-bin/amigo/go.cgi?view=details&query=GO:0003677)  [nucleus](http://amigo.geneontology.org/cgi-bin/amigo/go.cgi?view=details&query=GO:0005634)  [apoptosis](http://amigo.geneontology.org/cgi-bin/amigo/go.cgi?view=details&query=GO:0006915)  [cell cycle](http://amigo.geneontology.org/cgi-bin/amigo/go.cgi?view=details&query=GO:0007049)  [meiotic prophase I](http://amigo.geneontology.org/cgi-bin/amigo/go.cgi?view=details&query=GO:0007128)  [synaptonemal complex assembly](http://amigo.geneontology.org/cgi-bin/amigo/go.cgi?view=details&query=GO:0007130)  [male meiosis](http://amigo.geneontology.org/cgi-bin/amigo/go.cgi?view=details&query=GO:0007140)  [female meiosis](http://amigo.geneontology.org/cgi-bin/amigo/go.cgi?view=details&query=GO:0007143)  [fertilization](http://amigo.geneontology.org/cgi-bin/amigo/go.cgi?view=details&query=GO:0009566)  [organ morphogenesis](http://amigo.geneontology.org/cgi-bin/amigo/go.cgi?view=details&query=GO:0009887)  [protein heterodimerization activity](http://amigo.geneontology.org/cgi-bin/amigo/go.cgi?view=details&query=GO:0046982)  [male genitalia morphogenesis](http://amigo.geneontology.org/cgi-bin/amigo/go.cgi?view=details&query=GO:0048808)  [cell division](http://amigo.geneontology.org/cgi-bin/amigo/go.cgi?view=details&query=GO:0051301) |  |
| [206940_s_at](https://www.affymetrix.com/LinkServlet?&probeset=206940_s_at) | POU4F1 | POU class 4 homeobox 1 | 13 | -79173231 | [NM_006237](http://www.ncbi.nlm.nih.gov/entrez/query.fcgi?cmd=search&db=nucleotide&term=NM_006237%5BACCN%5D&doptcmdl=GenBank) | [5457](http://www.ncbi.nlm.nih.gov/sites/entrez?Db=gene&Cmd=DetailsSearch&Term=5457) | [13q31.1](http://www.ncbi.nlm.nih.gov/mapview/map_search.cgi?direct=on&idtype=gene&id=5457) | [Hs.654522](http://www.ncbi.nlm.nih.gov/UniGene/clust.cgi?ORG=Hs&CID=654522) | [23](http://www.ncbi.nih.gov/entrez/query.fcgi?tool=bioconductor&cmd=Retrieve&db=PubMed&list_uids=1357630%2C7623109%2C8234287%2C8248179%2C8889548%2C8941380%2C8995448%2C9448000%2C12427558%2C12432261%2C12441296%2C12477932%2C12810599%2C12893201%2C12911730%2C12934100%2C15021903%2C15272315%2C16247485%2C16276351%2C20348952%2C20376082%2C21116278) | [suckling behavior](http://amigo.geneontology.org/cgi-bin/amigo/go.cgi?view=details&query=GO:0001967)  [sequence-specific DNA binding transcription factor activity](http://amigo.geneontology.org/cgi-bin/amigo/go.cgi?view=details&query=GO:0003700)  [nucleus](http://amigo.geneontology.org/cgi-bin/amigo/go.cgi?view=details&query=GO:0005634)  [transcription, DNA-dependent](http://amigo.geneontology.org/cgi-bin/amigo/go.cgi?view=details&query=GO:0006351)  [regulation of transcription from RNA polymerase II promoter](http://amigo.geneontology.org/cgi-bin/amigo/go.cgi?view=details&query=GO:0006357)  [multicellular organismal development](http://amigo.geneontology.org/cgi-bin/amigo/go.cgi?view=details&query=GO:0007275)  [axonogenesis](http://amigo.geneontology.org/cgi-bin/amigo/go.cgi?view=details&query=GO:0007409)  [synapse assembly](http://amigo.geneontology.org/cgi-bin/amigo/go.cgi?view=details&query=GO:0007416)  [mesoderm development](http://amigo.geneontology.org/cgi-bin/amigo/go.cgi?view=details&query=GO:0007498)  [positive regulation of gene-specific transcription from RNA polymerase II promoter](http://amigo.geneontology.org/cgi-bin/amigo/go.cgi?view=details&query=GO:0010552)  [negative regulation of gene-specific transcription from RNA polymerase II promoter](http://amigo.geneontology.org/cgi-bin/amigo/go.cgi?view=details&query=GO:0010553)  [cell migration in hindbrain](http://amigo.geneontology.org/cgi-bin/amigo/go.cgi?view=details&query=GO:0021535)  [central nervous system neuron differentiation](http://amigo.geneontology.org/cgi-bin/amigo/go.cgi?view=details&query=GO:0021953)  [transcription regulator activity](http://amigo.geneontology.org/cgi-bin/amigo/go.cgi?view=details&query=GO:0030528)  [positive regulation of apoptosis](http://amigo.geneontology.org/cgi-bin/amigo/go.cgi?view=details&query=GO:0043065)  [sequence-specific DNA binding](http://amigo.geneontology.org/cgi-bin/amigo/go.cgi?view=details&query=GO:0043565)  [peripheral nervous system neuron differentiation](http://amigo.geneontology.org/cgi-bin/amigo/go.cgi?view=details&query=GO:0048934)  [regulation of neurogenesis](http://amigo.geneontology.org/cgi-bin/amigo/go.cgi?view=details&query=GO:0050767)  [proprioception involved in equilibrioception](http://amigo.geneontology.org/cgi-bin/amigo/go.cgi?view=details&query=GO:0051355) |  |
| [207015_s_at](https://www.affymetrix.com/LinkServlet?&probeset=207015_s_at) | ALDH1A2 | aldehyde dehydrogenase 1 family, member A2 | 15 | -58245627, -58245627 | [NM_003888](http://www.ncbi.nlm.nih.gov/entrez/query.fcgi?cmd=search&db=nucleotide&term=NM_003888%5BACCN%5D&doptcmdl=GenBank) | [8854](http://www.ncbi.nlm.nih.gov/sites/entrez?Db=gene&Cmd=DetailsSearch&Term=8854) | [15q21.3](http://www.ncbi.nlm.nih.gov/mapview/map_search.cgi?direct=on&idtype=gene&id=8854) | [Hs.643455](http://www.ncbi.nlm.nih.gov/UniGene/clust.cgi?ORG=Hs&CID=643455) | [23](http://www.ncbi.nih.gov/entrez/query.fcgi?tool=bioconductor&cmd=Retrieve&db=PubMed&list_uids=8663198%2C8797830%2C9819382%2C10192400%2C11953746%2C12477932%2C14702039%2C14718574%2C15489334%2C16166285%2C16237707%2C16368932%2C18029348%2C18495959%2C19343046%2C19478994%2C19609347%2C19703508%2C19886994%2C20308937%2C20375987%2C20379614%2C20450613) | [retinal dehydrogenase activity](http://amigo.geneontology.org/cgi-bin/amigo/go.cgi?view=details&query=GO:0001758)  [3-chloroallyl aldehyde dehydrogenase activity](http://amigo.geneontology.org/cgi-bin/amigo/go.cgi?view=details&query=GO:0004028)  [nucleus](http://amigo.geneontology.org/cgi-bin/amigo/go.cgi?view=details&query=GO:0005634)  [cytoplasm](http://amigo.geneontology.org/cgi-bin/amigo/go.cgi?view=details&query=GO:0005737)  [vitamin A metabolic process](http://amigo.geneontology.org/cgi-bin/amigo/go.cgi?view=details&query=GO:0006776)  [negative regulation of cell proliferation](http://amigo.geneontology.org/cgi-bin/amigo/go.cgi?view=details&query=GO:0008285)  [oxidoreductase activity](http://amigo.geneontology.org/cgi-bin/amigo/go.cgi?view=details&query=GO:0016491)  [retinal binding](http://amigo.geneontology.org/cgi-bin/amigo/go.cgi?view=details&query=GO:0016918)  [neural tube development](http://amigo.geneontology.org/cgi-bin/amigo/go.cgi?view=details&query=GO:0021915)  [response to cytokine stimulus](http://amigo.geneontology.org/cgi-bin/amigo/go.cgi?view=details&query=GO:0034097)  [retinoic acid metabolic process](http://amigo.geneontology.org/cgi-bin/amigo/go.cgi?view=details&query=GO:0042573)  [oxidation-reduction process](http://amigo.geneontology.org/cgi-bin/amigo/go.cgi?view=details&query=GO:0055114) | [Retinol metabolism](http://www.genome.ad.jp/dbget-bin/show_pathway?MAP00830+1.2.1.36)  [Metabolic pathways](http://www.genome.ad.jp/dbget-bin/show_pathway?MAP01100+1.2.1.36) |
| [207016_s_at](https://www.affymetrix.com/LinkServlet?&probeset=207016_s_at) | ALDH1A2 | aldehyde dehydrogenase 1 family, member A2 | 15 | -58245627, -58245627 | [AB015228](http://www.ncbi.nlm.nih.gov/entrez/query.fcgi?cmd=search&db=nucleotide&term=AB015228%5BACCN%5D&doptcmdl=GenBank) | [8854](http://www.ncbi.nlm.nih.gov/sites/entrez?Db=gene&Cmd=DetailsSearch&Term=8854) | [15q21.3](http://www.ncbi.nlm.nih.gov/mapview/map_search.cgi?direct=on&idtype=gene&id=8854) | [Hs.643455](http://www.ncbi.nlm.nih.gov/UniGene/clust.cgi?ORG=Hs&CID=643455) | [23](http://www.ncbi.nih.gov/entrez/query.fcgi?tool=bioconductor&cmd=Retrieve&db=PubMed&list_uids=8663198%2C8797830%2C9819382%2C10192400%2C11953746%2C12477932%2C14702039%2C14718574%2C15489334%2C16166285%2C16237707%2C16368932%2C18029348%2C18495959%2C19343046%2C19478994%2C19609347%2C19703508%2C19886994%2C20308937%2C20375987%2C20379614%2C20450613) | [retinal dehydrogenase activity](http://amigo.geneontology.org/cgi-bin/amigo/go.cgi?view=details&query=GO:0001758)  [3-chloroallyl aldehyde dehydrogenase activity](http://amigo.geneontology.org/cgi-bin/amigo/go.cgi?view=details&query=GO:0004028)  [nucleus](http://amigo.geneontology.org/cgi-bin/amigo/go.cgi?view=details&query=GO:0005634)  [cytoplasm](http://amigo.geneontology.org/cgi-bin/amigo/go.cgi?view=details&query=GO:0005737)  [vitamin A metabolic process](http://amigo.geneontology.org/cgi-bin/amigo/go.cgi?view=details&query=GO:0006776)  [negative regulation of cell proliferation](http://amigo.geneontology.org/cgi-bin/amigo/go.cgi?view=details&query=GO:0008285)  [oxidoreductase activity](http://amigo.geneontology.org/cgi-bin/amigo/go.cgi?view=details&query=GO:0016491)  [retinal binding](http://amigo.geneontology.org/cgi-bin/amigo/go.cgi?view=details&query=GO:0016918)  [neural tube development](http://amigo.geneontology.org/cgi-bin/amigo/go.cgi?view=details&query=GO:0021915)  [response to cytokine stimulus](http://amigo.geneontology.org/cgi-bin/amigo/go.cgi?view=details&query=GO:0034097)  [retinoic acid metabolic process](http://amigo.geneontology.org/cgi-bin/amigo/go.cgi?view=details&query=GO:0042573)  [oxidation-reduction process](http://amigo.geneontology.org/cgi-bin/amigo/go.cgi?view=details&query=GO:0055114) | [Retinol metabolism](http://www.genome.ad.jp/dbget-bin/show_pathway?MAP00830+1.2.1.36)  [Metabolic pathways](http://www.genome.ad.jp/dbget-bin/show_pathway?MAP01100+1.2.1.36) |
| [207250_at](https://www.affymetrix.com/LinkServlet?&probeset=207250_at) | SIX6 | SIX homeobox 6 | 14 | 60975937 | [NM_007374](http://www.ncbi.nlm.nih.gov/entrez/query.fcgi?cmd=search&db=nucleotide&term=NM_007374%5BACCN%5D&doptcmdl=GenBank) | [4990](http://www.ncbi.nlm.nih.gov/sites/entrez?Db=gene&Cmd=DetailsSearch&Term=4990) | [14q23.1](http://www.ncbi.nlm.nih.gov/mapview/map_search.cgi?direct=on&idtype=gene&id=4990) | [Hs.194756](http://www.ncbi.nlm.nih.gov/UniGene/clust.cgi?ORG=Hs&CID=194756) | [13](http://www.ncbi.nih.gov/entrez/query.fcgi?tool=bioconductor&cmd=Retrieve&db=PubMed&list_uids=10381575%2C10512683%2C11493467%2C12441302%2C12477932%2C15266624%2C15505031%2C18029348%2C18293925%2C18666230%2C20057906%2C20211142%2C20881960) | [sequence-specific DNA binding transcription factor activity](http://amigo.geneontology.org/cgi-bin/amigo/go.cgi?view=details&query=GO:0003700)  [nucleus](http://amigo.geneontology.org/cgi-bin/amigo/go.cgi?view=details&query=GO:0005634)  [regulation of transcription, DNA-dependent](http://amigo.geneontology.org/cgi-bin/amigo/go.cgi?view=details&query=GO:0006355)  [multicellular organismal development](http://amigo.geneontology.org/cgi-bin/amigo/go.cgi?view=details&query=GO:0007275)  [visual perception](http://amigo.geneontology.org/cgi-bin/amigo/go.cgi?view=details&query=GO:0007601)  [organ morphogenesis](http://amigo.geneontology.org/cgi-bin/amigo/go.cgi?view=details&query=GO:0009887)  [sequence-specific DNA binding](http://amigo.geneontology.org/cgi-bin/amigo/go.cgi?view=details&query=GO:0043565) |  |
| [208029_s_at](https://www.affymetrix.com/LinkServlet?&probeset=208029_s_at) | LAPTM4B | lysosomal protein transmembrane 4 beta | 8 | 98787808 | [NM_018407](http://www.ncbi.nlm.nih.gov/entrez/query.fcgi?cmd=search&db=nucleotide&term=NM_018407%5BACCN%5D&doptcmdl=GenBank) | [55353](http://www.ncbi.nlm.nih.gov/sites/entrez?Db=gene&Cmd=DetailsSearch&Term=55353) | [8q22.1](http://www.ncbi.nlm.nih.gov/mapview/map_search.cgi?direct=on&idtype=gene&id=55353) | [Hs.492314](http://www.ncbi.nlm.nih.gov/UniGene/clust.cgi?ORG=Hs&CID=492314) | [24](http://www.ncbi.nih.gov/entrez/query.fcgi?tool=bioconductor&cmd=Retrieve&db=PubMed&list_uids=11076863%2C11230166%2C12477932%2C12640618%2C12902989%2C15162524%2C15489334%2C15489336%2C15911104%2C15968325%2C16303743%2C16381901%2C16769693%2C17074969%2C17965115%2C18334282%2C18949404%2C19690886%2C19843073%2C20358632%2C20379614%2C20583413%2C20711237%2C20881850) | [protein binding](http://amigo.geneontology.org/cgi-bin/amigo/go.cgi?view=details&query=GO:0005515)  [transport](http://amigo.geneontology.org/cgi-bin/amigo/go.cgi?view=details&query=GO:0006810)  [endomembrane system](http://amigo.geneontology.org/cgi-bin/amigo/go.cgi?view=details&query=GO:0012505)  [membrane](http://amigo.geneontology.org/cgi-bin/amigo/go.cgi?view=details&query=GO:0016020)  [integral to membrane](http://amigo.geneontology.org/cgi-bin/amigo/go.cgi?view=details&query=GO:0016021) | [Lysosome](http://www.genome.ad.jp/kegg/pathway/hsa/hsa04142.html) |
| [208165_s_at](https://www.affymetrix.com/LinkServlet?&probeset=208165_s_at) | PRSS16 | protease, serine, 16 (thymus) | 6 | 27215501 | [NM_005865](http://www.ncbi.nlm.nih.gov/entrez/query.fcgi?cmd=search&db=nucleotide&term=NM_005865%5BACCN%5D&doptcmdl=GenBank) | [10279](http://www.ncbi.nlm.nih.gov/sites/entrez?Db=gene&Cmd=DetailsSearch&Term=10279) | [6p21](http://www.ncbi.nlm.nih.gov/mapview/map_search.cgi?direct=on&idtype=gene&id=10279) | [Hs.274407](http://www.ncbi.nlm.nih.gov/UniGene/clust.cgi?ORG=Hs&CID=274407) | [10](http://www.ncbi.nih.gov/entrez/query.fcgi?tool=bioconductor&cmd=Retrieve&db=PubMed&list_uids=8812418%2C10527559%2C12140752%2C14574404%2C14702039%2C15592422%2C17584581%2C19295542%2C19571808%2C19571809) | [cellular_component](http://amigo.geneontology.org/cgi-bin/amigo/go.cgi?view=details&query=GO:0005575)  [proteolysis](http://amigo.geneontology.org/cgi-bin/amigo/go.cgi?view=details&query=GO:0006508)  [peptidase activity](http://amigo.geneontology.org/cgi-bin/amigo/go.cgi?view=details&query=GO:0008233)  [serine-type peptidase activity](http://amigo.geneontology.org/cgi-bin/amigo/go.cgi?view=details&query=GO:0008236)  [cytoplasmic membrane-bounded vesicle](http://amigo.geneontology.org/cgi-bin/amigo/go.cgi?view=details&query=GO:0016023)  [protein catabolic process](http://amigo.geneontology.org/cgi-bin/amigo/go.cgi?view=details&query=GO:0030163) |  |
| [208498_s_at](https://www.affymetrix.com/LinkServlet?&probeset=208498_s_at) |  |  |  |  | [NM_004038](http://www.ncbi.nlm.nih.gov/entrez/query.fcgi?cmd=search&db=nucleotide&term=NM_004038%5BACCN%5D&doptcmdl=GenBank) |  |  |  |  | [alpha-amylase activity](http://amigo.geneontology.org/cgi-bin/amigo/go.cgi?view=details&query=GO:0004556)  [alpha-amylase activity](http://amigo.geneontology.org/cgi-bin/amigo/go.cgi?view=details&query=GO:0004556)  [alpha-amylase activity](http://amigo.geneontology.org/cgi-bin/amigo/go.cgi?view=details&query=GO:0004556)  [calcium ion binding](http://amigo.geneontology.org/cgi-bin/amigo/go.cgi?view=details&query=GO:0005509)  [protein binding](http://amigo.geneontology.org/cgi-bin/amigo/go.cgi?view=details&query=GO:0005515)  [extracellular region](http://amigo.geneontology.org/cgi-bin/amigo/go.cgi?view=details&query=GO:0005576)  [extracellular region](http://amigo.geneontology.org/cgi-bin/amigo/go.cgi?view=details&query=GO:0005576)  [extracellular space](http://amigo.geneontology.org/cgi-bin/amigo/go.cgi?view=details&query=GO:0005615)  [carbohydrate metabolic process](http://amigo.geneontology.org/cgi-bin/amigo/go.cgi?view=details&query=GO:0005975)  [carbohydrate metabolic process](http://amigo.geneontology.org/cgi-bin/amigo/go.cgi?view=details&query=GO:0005975)  [digestion](http://amigo.geneontology.org/cgi-bin/amigo/go.cgi?view=details&query=GO:0007586)  [carbohydrate catabolic process](http://amigo.geneontology.org/cgi-bin/amigo/go.cgi?view=details&query=GO:0016052)  [hydrolase activity, acting on glycosyl bonds](http://amigo.geneontology.org/cgi-bin/amigo/go.cgi?view=details&query=GO:0016798)  [chloride ion binding](http://amigo.geneontology.org/cgi-bin/amigo/go.cgi?view=details&query=GO:0031404)  [polysaccharide digestion](http://amigo.geneontology.org/cgi-bin/amigo/go.cgi?view=details&query=GO:0044245)  [metal ion binding](http://amigo.geneontology.org/cgi-bin/amigo/go.cgi?view=details&query=GO:0046872) |  |
| [209031_at](https://www.affymetrix.com/LinkServlet?&probeset=209031_at) | CADM1 | cell adhesion molecule 1 | 11 | -115044345 | [AL519710](http://www.ncbi.nlm.nih.gov/entrez/query.fcgi?cmd=search&db=nucleotide&term=AL519710%5BACCN%5D&doptcmdl=GenBank) | [23705](http://www.ncbi.nlm.nih.gov/sites/entrez?Db=gene&Cmd=DetailsSearch&Term=23705) | [11q23.2](http://www.ncbi.nlm.nih.gov/mapview/map_search.cgi?direct=on&idtype=gene&id=23705) | [Hs.370510](http://www.ncbi.nlm.nih.gov/UniGene/clust.cgi?ORG=Hs&CID=370510) | [72](http://www.ncbi.nih.gov/entrez/query.fcgi?tool=bioconductor&cmd=Retrieve&db=PubMed&list_uids=10610705%2C11279526%2C12050160%2C12079507%2C12112527%2C12202822%2C12234973%2C12432281%2C12477932%2C12716461%2C12759359%2C12826663%2C12920246%2C12925956%2C12942568%2C12973698%2C12975309%2C13679854%2C14559819%2C14633730%2C14639656%2C14970278%2C15184878%2C15231748%2C15471956%2C15535129%2C15741237%2C15781451%2C15811952%2C15905536%2C16083501%2C16108829%2C16205641%2C16303743%2C16311015%2C16394014%2C17018592%2C17130425%2C17260099%2C17300670%2C17326163%2C18084322%2C18332875%2C18471525%2C18498117%2C18559103%2C18684968%2C18726896%2C18922876%2C18957284%2C19115211%2C19371721%2C19561085%2C19643986%2C19653122%2C19783739%2C19835597%2C19854157%2C19913121%2C19995226%2C20190806%2C20215110%2C20340131%2C20375924%2C20379614%2C20570966%2C20628086%2C20677014%2C20709797%2C20838585%2C21081044%2C21329006) | [liver development](http://amigo.geneontology.org/cgi-bin/amigo/go.cgi?view=details&query=GO:0001889)  [receptor binding](http://amigo.geneontology.org/cgi-bin/amigo/go.cgi?view=details&query=GO:0005102)  [receptor binding](http://amigo.geneontology.org/cgi-bin/amigo/go.cgi?view=details&query=GO:0005102)  [plasma membrane](http://amigo.geneontology.org/cgi-bin/amigo/go.cgi?view=details&query=GO:0005886)  [plasma membrane](http://amigo.geneontology.org/cgi-bin/amigo/go.cgi?view=details&query=GO:0005886)  [plasma membrane](http://amigo.geneontology.org/cgi-bin/amigo/go.cgi?view=details&query=GO:0005886)  [cell-cell junction](http://amigo.geneontology.org/cgi-bin/amigo/go.cgi?view=details&query=GO:0005911)  [apoptosis](http://amigo.geneontology.org/cgi-bin/amigo/go.cgi?view=details&query=GO:0006915)  [cell adhesion](http://amigo.geneontology.org/cgi-bin/amigo/go.cgi?view=details&query=GO:0007155)  [homophilic cell adhesion](http://amigo.geneontology.org/cgi-bin/amigo/go.cgi?view=details&query=GO:0007156)  [heterophilic cell-cell adhesion](http://amigo.geneontology.org/cgi-bin/amigo/go.cgi?view=details&query=GO:0007157)  [multicellular organismal development](http://amigo.geneontology.org/cgi-bin/amigo/go.cgi?view=details&query=GO:0007275)  [spermatogenesis](http://amigo.geneontology.org/cgi-bin/amigo/go.cgi?view=details&query=GO:0007283)  [synapse assembly](http://amigo.geneontology.org/cgi-bin/amigo/go.cgi?view=details&query=GO:0007416)  [synaptic vesicle](http://amigo.geneontology.org/cgi-bin/amigo/go.cgi?view=details&query=GO:0008021)  [protein C-terminus binding](http://amigo.geneontology.org/cgi-bin/amigo/go.cgi?view=details&query=GO:0008022)  [cell recognition](http://amigo.geneontology.org/cgi-bin/amigo/go.cgi?view=details&query=GO:0008037)  [cell recognition](http://amigo.geneontology.org/cgi-bin/amigo/go.cgi?view=details&query=GO:0008037)  [integral to membrane](http://amigo.geneontology.org/cgi-bin/amigo/go.cgi?view=details&query=GO:0016021)  [basolateral plasma membrane](http://amigo.geneontology.org/cgi-bin/amigo/go.cgi?view=details&query=GO:0016323)  [calcium-independent cell-cell adhesion](http://amigo.geneontology.org/cgi-bin/amigo/go.cgi?view=details&query=GO:0016338)  [synaptosome](http://amigo.geneontology.org/cgi-bin/amigo/go.cgi?view=details&query=GO:0019717)  [cell differentiation](http://amigo.geneontology.org/cgi-bin/amigo/go.cgi?view=details&query=GO:0030154)  [PDZ domain binding](http://amigo.geneontology.org/cgi-bin/amigo/go.cgi?view=details&query=GO:0030165)  [axon](http://amigo.geneontology.org/cgi-bin/amigo/go.cgi?view=details&query=GO:0030424)  [dendrite](http://amigo.geneontology.org/cgi-bin/amigo/go.cgi?view=details&query=GO:0030425)  [cell junction assembly](http://amigo.geneontology.org/cgi-bin/amigo/go.cgi?view=details&query=GO:0034329)  [adherens junction organization](http://amigo.geneontology.org/cgi-bin/amigo/go.cgi?view=details&query=GO:0034332)  [susceptibility to natural killer cell mediated cytotoxicity](http://amigo.geneontology.org/cgi-bin/amigo/go.cgi?view=details&query=GO:0042271)  [susceptibility to natural killer cell mediated cytotoxicity](http://amigo.geneontology.org/cgi-bin/amigo/go.cgi?view=details&query=GO:0042271)  [protein homodimerization activity](http://amigo.geneontology.org/cgi-bin/amigo/go.cgi?view=details&query=GO:0042803)  [synapse](http://amigo.geneontology.org/cgi-bin/amigo/go.cgi?view=details&query=GO:0045202)  [cell-cell junction organization](http://amigo.geneontology.org/cgi-bin/amigo/go.cgi?view=details&query=GO:0045216)  [positive regulation of natural killer cell mediated cytotoxicity](http://amigo.geneontology.org/cgi-bin/amigo/go.cgi?view=details&query=GO:0045954)  [positive regulation of natural killer cell mediated cytotoxicity](http://amigo.geneontology.org/cgi-bin/amigo/go.cgi?view=details&query=GO:0045954)  [positive regulation of cytokine secretion](http://amigo.geneontology.org/cgi-bin/amigo/go.cgi?view=details&query=GO:0050715)  [detection of stimulus](http://amigo.geneontology.org/cgi-bin/amigo/go.cgi?view=details&query=GO:0051606)  [detection of stimulus](http://amigo.geneontology.org/cgi-bin/amigo/go.cgi?view=details&query=GO:0051606) | [Cell adhesion molecules (CAMs)](http://www.genome.ad.jp/kegg/pathway/hsa/hsa04514.html) |
| [209035_at](https://www.affymetrix.com/LinkServlet?&probeset=209035_at) | MDK | midkine (neurite growth-promoting factor 2) | 11 | 46402617, 46403218, 46403302 | [M69148](http://www.ncbi.nlm.nih.gov/entrez/query.fcgi?cmd=search&db=nucleotide&term=M69148%5BACCN%5D&doptcmdl=GenBank) | [4192](http://www.ncbi.nlm.nih.gov/sites/entrez?Db=gene&Cmd=DetailsSearch&Term=4192) | [11p11.2](http://www.ncbi.nlm.nih.gov/mapview/map_search.cgi?direct=on&idtype=gene&id=4192) | [Hs.82045](http://www.ncbi.nlm.nih.gov/UniGene/clust.cgi?ORG=Hs&CID=82045) | [85](http://www.ncbi.nih.gov/entrez/query.fcgi?tool=bioconductor&cmd=Retrieve&db=PubMed&list_uids=1639750%2C1701366%2C1768439%2C1883381%2C2025291%2C7835084%2C8406506%2C8471163%2C8621465%2C8694802%2C9089390%2C9384573%2C9452495%2C10212223%2C10706604%2C10772929%2C11048798%2C11925507%2C12077357%2C12122009%2C12127679%2C12147681%2C12175547%2C12477932%2C12579281%2C12841873%2C14970216%2C15050737%2C15138367%2C15146197%2C15146411%2C15197188%2C15201962%2C15340161%2C15355893%2C15489334%2C15734764%2C15781266%2C15897897%2C16169070%2C16341674%2C16713569%2C16895951%2C17066487%2C17171794%2C17267033%2C17368428%2C17379400%2C17451201%2C17493173%2C17607302%2C17845207%2C17931612%2C17971413%2C18176965%2C18195496%2C18329695%2C18422745%2C18469519%2C18657127%2C18682710%2C18698021%2C18712601%2C18851943%2C18956201%2C18985819%2C19016768%2C19060126%2C19112919%2C19250738%2C19409372%2C19538527%2C19698107%2C19728850%2C20308059%2C20350697%2C20442752%2C20447063%2C20511550%2C20525245%2C20544404%2C20694011%2C20811700%2C21094842%2C21208277) | [extracellular region](http://amigo.geneontology.org/cgi-bin/amigo/go.cgi?view=details&query=GO:0005576)  [signal transduction](http://amigo.geneontology.org/cgi-bin/amigo/go.cgi?view=details&query=GO:0007165)  [multicellular organismal development](http://amigo.geneontology.org/cgi-bin/amigo/go.cgi?view=details&query=GO:0007275)  [nervous system development](http://amigo.geneontology.org/cgi-bin/amigo/go.cgi?view=details&query=GO:0007399)  [growth factor activity](http://amigo.geneontology.org/cgi-bin/amigo/go.cgi?view=details&query=GO:0008083)  [heparin binding](http://amigo.geneontology.org/cgi-bin/amigo/go.cgi?view=details&query=GO:0008201)  [response to wounding](http://amigo.geneontology.org/cgi-bin/amigo/go.cgi?view=details&query=GO:0009611)  [cell differentiation](http://amigo.geneontology.org/cgi-bin/amigo/go.cgi?view=details&query=GO:0030154)  [adrenal gland development](http://amigo.geneontology.org/cgi-bin/amigo/go.cgi?view=details&query=GO:0030325)  [positive regulation of cell division](http://amigo.geneontology.org/cgi-bin/amigo/go.cgi?view=details&query=GO:0051781) |  |
| [209228_x_at](https://www.affymetrix.com/LinkServlet?&probeset=209228_x_at) | TUSC3 | tumor suppressor candidate 3 | 8 | 15397729 | [U42349](http://www.ncbi.nlm.nih.gov/entrez/query.fcgi?cmd=search&db=nucleotide&term=U42349%5BACCN%5D&doptcmdl=GenBank) | [7991](http://www.ncbi.nlm.nih.gov/sites/entrez?Db=gene&Cmd=DetailsSearch&Term=7991) | [8p22](http://www.ncbi.nlm.nih.gov/mapview/map_search.cgi?direct=on&idtype=gene&id=7991) | [Hs.591845](http://www.ncbi.nlm.nih.gov/UniGene/clust.cgi?ORG=Hs&CID=591845) [Hs.613699](http://www.ncbi.nlm.nih.gov/UniGene/clust.cgi?ORG=Hs&CID=613699) | [15](http://www.ncbi.nih.gov/entrez/query.fcgi?tool=bioconductor&cmd=Retrieve&db=PubMed&list_uids=8661104%2C9778121%2C10097140%2C12477932%2C12887896%2C14718574%2C15231748%2C15489334%2C15835887%2C16270321%2C16344560%2C17641416%2C18452889%2C18455129%2C19717468) | [dolichyl-diphosphooligosaccharide-protein glycotransferase activity](http://amigo.geneontology.org/cgi-bin/amigo/go.cgi?view=details&query=GO:0004579)  [endoplasmic reticulum](http://amigo.geneontology.org/cgi-bin/amigo/go.cgi?view=details&query=GO:0005783)  [endoplasmic reticulum membrane](http://amigo.geneontology.org/cgi-bin/amigo/go.cgi?view=details&query=GO:0005789)  [oligosaccharyltransferase complex](http://amigo.geneontology.org/cgi-bin/amigo/go.cgi?view=details&query=GO:0008250)  [membrane](http://amigo.geneontology.org/cgi-bin/amigo/go.cgi?view=details&query=GO:0016020)  [integral to membrane](http://amigo.geneontology.org/cgi-bin/amigo/go.cgi?view=details&query=GO:0016021)  [protein N-linked glycosylation via asparagine](http://amigo.geneontology.org/cgi-bin/amigo/go.cgi?view=details&query=GO:0018279)  [post-translational protein modification](http://amigo.geneontology.org/cgi-bin/amigo/go.cgi?view=details&query=GO:0043687)  [cellular protein metabolic process](http://amigo.geneontology.org/cgi-bin/amigo/go.cgi?view=details&query=GO:0044267)  [cell redox homeostasis](http://amigo.geneontology.org/cgi-bin/amigo/go.cgi?view=details&query=GO:0045454) | [N-Glycan biosynthesis](http://www.genome.ad.jp/kegg/pathway/hsa/hsa00510.html)  [Metabolic pathways](http://www.genome.ad.jp/kegg/pathway/hsa/hsa01100.html)  [Protein processing in endoplasmic reticulum](http://www.genome.ad.jp/kegg/pathway/hsa/hsa04141.html) |
| [209338_at](https://www.affymetrix.com/LinkServlet?&probeset=209338_at) | TFCP2 | transcription factor CP2 | 12 | -51487540 | [U03494](http://www.ncbi.nlm.nih.gov/entrez/query.fcgi?cmd=search&db=nucleotide&term=U03494%5BACCN%5D&doptcmdl=GenBank) | [7024](http://www.ncbi.nlm.nih.gov/sites/entrez?Db=gene&Cmd=DetailsSearch&Term=7024) | [12q13](http://www.ncbi.nlm.nih.gov/mapview/map_search.cgi?direct=on&idtype=gene&id=7024) | [Hs.48849](http://www.ncbi.nlm.nih.gov/UniGene/clust.cgi?ORG=Hs&CID=48849) | [44](http://www.ncbi.nih.gov/entrez/query.fcgi?tool=bioconductor&cmd=Retrieve&db=PubMed&list_uids=1732747%2C7828600%2C8035790%2C8114710%2C8157699%2C8289393%2C8586452%2C9371597%2C9685356%2C10455131%2C10888618%2C11001930%2C11003662%2C11283204%2C11574690%2C11865070%2C11884454%2C11940654%2C12393799%2C12477932%2C12555245%2C12661759%2C15489334%2C15857981%2C16169070%2C16263792%2C16272261%2C16344560%2C16648487%2C16710089%2C16713569%2C16973241%2C17456737%2C17902044%2C18164103%2C18307033%2C18629613%2C18787404%2C19889475%2C19902333%2C20211142%2C20404171%2C20562859%2C20661472) | [DNA binding](http://amigo.geneontology.org/cgi-bin/amigo/go.cgi?view=details&query=GO:0003677)  [sequence-specific DNA binding transcription factor activity](http://amigo.geneontology.org/cgi-bin/amigo/go.cgi?view=details&query=GO:0003700)  [protein binding](http://amigo.geneontology.org/cgi-bin/amigo/go.cgi?view=details&query=GO:0005515)  [cellular_component](http://amigo.geneontology.org/cgi-bin/amigo/go.cgi?view=details&query=GO:0005575)  [nucleus](http://amigo.geneontology.org/cgi-bin/amigo/go.cgi?view=details&query=GO:0005634)  [transcription, DNA-dependent](http://amigo.geneontology.org/cgi-bin/amigo/go.cgi?view=details&query=GO:0006351)  [regulation of transcription from RNA polymerase II promoter](http://amigo.geneontology.org/cgi-bin/amigo/go.cgi?view=details&query=GO:0006357)  [regulation of transcription](http://amigo.geneontology.org/cgi-bin/amigo/go.cgi?view=details&query=GO:0045449) |  |
| [209448_at](https://www.affymetrix.com/LinkServlet?&probeset=209448_at) | HTATIP2 | HIV-1 Tat interactive protein 2, 30kDa | 11 | 20385230, 20385246, 20385288, 20385402, 20385686 | [BC002439](http://www.ncbi.nlm.nih.gov/entrez/query.fcgi?cmd=search&db=nucleotide&term=BC002439%5BACCN%5D&doptcmdl=GenBank) | [10553](http://www.ncbi.nlm.nih.gov/sites/entrez?Db=gene&Cmd=DetailsSearch&Term=10553) | [11p15.1](http://www.ncbi.nlm.nih.gov/mapview/map_search.cgi?direct=on&idtype=gene&id=10553) | [Hs.90753](http://www.ncbi.nlm.nih.gov/UniGene/clust.cgi?ORG=Hs&CID=90753) | [35](http://www.ncbi.nih.gov/entrez/query.fcgi?tool=bioconductor&cmd=Retrieve&db=PubMed&list_uids=8125298%2C9174052%2C9373149%2C9482853%2C10395547%2C10611237%2C10698937%2C10892349%2C11313954%2C12477932%2C14695192%2C15073177%2C15124103%2C15282309%2C15342556%2C15489334%2C15493507%2C15633220%2C15728189%2C16615932%2C16799960%2C17097132%2C17997990%2C18029348%2C18519672%2C18528861%2C18537194%2C18972434%2C19010857%2C19027726%2C19104151%2C19349353%2C19798571%2C19839715%2C20374651) | [RNA polymerase II transcription factor activity](http://amigo.geneontology.org/cgi-bin/amigo/go.cgi?view=details&query=GO:0003702)  [transcription coactivator activity](http://amigo.geneontology.org/cgi-bin/amigo/go.cgi?view=details&query=GO:0003713)  [protein binding](http://amigo.geneontology.org/cgi-bin/amigo/go.cgi?view=details&query=GO:0005515)  [nucleus](http://amigo.geneontology.org/cgi-bin/amigo/go.cgi?view=details&query=GO:0005634)  [nuclear envelope](http://amigo.geneontology.org/cgi-bin/amigo/go.cgi?view=details&query=GO:0005635)  [cytoplasm](http://amigo.geneontology.org/cgi-bin/amigo/go.cgi?view=details&query=GO:0005737)  [regulation of transcription from RNA polymerase II promoter](http://amigo.geneontology.org/cgi-bin/amigo/go.cgi?view=details&query=GO:0006357)  [cellular amino acid metabolic process](http://amigo.geneontology.org/cgi-bin/amigo/go.cgi?view=details&query=GO:0006520)  [anti-apoptosis](http://amigo.geneontology.org/cgi-bin/amigo/go.cgi?view=details&query=GO:0006916)  [induction of apoptosis](http://amigo.geneontology.org/cgi-bin/amigo/go.cgi?view=details&query=GO:0006917)  [multicellular organismal development](http://amigo.geneontology.org/cgi-bin/amigo/go.cgi?view=details&query=GO:0007275)  [oxidoreductase activity](http://amigo.geneontology.org/cgi-bin/amigo/go.cgi?view=details&query=GO:0016491)  [oxidoreductase activity, acting on the aldehyde or oxo group of donors, NAD or NADP as acceptor](http://amigo.geneontology.org/cgi-bin/amigo/go.cgi?view=details&query=GO:0016620)  [cell differentiation](http://amigo.geneontology.org/cgi-bin/amigo/go.cgi?view=details&query=GO:0030154)  [regulation of apoptosis](http://amigo.geneontology.org/cgi-bin/amigo/go.cgi?view=details&query=GO:0042981)  [interspecies interaction between organisms](http://amigo.geneontology.org/cgi-bin/amigo/go.cgi?view=details&query=GO:0044419)  [regulation of angiogenesis](http://amigo.geneontology.org/cgi-bin/amigo/go.cgi?view=details&query=GO:0045765)  [positive regulation of transcription](http://amigo.geneontology.org/cgi-bin/amigo/go.cgi?view=details&query=GO:0045941)  [nuclear import](http://amigo.geneontology.org/cgi-bin/amigo/go.cgi?view=details&query=GO:0051170)  [NAD binding](http://amigo.geneontology.org/cgi-bin/amigo/go.cgi?view=details&query=GO:0051287)  [oxidation-reduction process](http://amigo.geneontology.org/cgi-bin/amigo/go.cgi?view=details&query=GO:0055114) |  |
| [209522_s_at](https://www.affymetrix.com/LinkServlet?&probeset=209522_s_at) | CRAT | carnitine O-acetyltransferase | 9 | -131857074 | [BC000723](http://www.ncbi.nlm.nih.gov/entrez/query.fcgi?cmd=search&db=nucleotide&term=BC000723%5BACCN%5D&doptcmdl=GenBank) | [1384](http://www.ncbi.nlm.nih.gov/sites/entrez?Db=gene&Cmd=DetailsSearch&Term=1384) | [9q34.1](http://www.ncbi.nlm.nih.gov/mapview/map_search.cgi?direct=on&idtype=gene&id=1384) | [Hs.12068](http://www.ncbi.nlm.nih.gov/UniGene/clust.cgi?ORG=Hs&CID=12068) | [16](http://www.ncbi.nih.gov/entrez/query.fcgi?tool=bioconductor&cmd=Retrieve&db=PubMed&list_uids=1456745%2C7829107%2C7945262%2C11001805%2C12077440%2C12477932%2C12526798%2C12562770%2C15099582%2C15489334%2C16756494%2C19553674%2C19913121%2C20628086%2C20677014%2C20877624) | [carnitine O-acetyltransferase activity](http://amigo.geneontology.org/cgi-bin/amigo/go.cgi?view=details&query=GO:0004092)  [mitochondrion](http://amigo.geneontology.org/cgi-bin/amigo/go.cgi?view=details&query=GO:0005739)  [mitochondrial inner membrane](http://amigo.geneontology.org/cgi-bin/amigo/go.cgi?view=details&query=GO:0005743)  [peroxisome](http://amigo.geneontology.org/cgi-bin/amigo/go.cgi?view=details&query=GO:0005777)  [peroxisomal matrix](http://amigo.geneontology.org/cgi-bin/amigo/go.cgi?view=details&query=GO:0005782)  [endoplasmic reticulum](http://amigo.geneontology.org/cgi-bin/amigo/go.cgi?view=details&query=GO:0005783)  [generation of precursor metabolites and energy](http://amigo.geneontology.org/cgi-bin/amigo/go.cgi?view=details&query=GO:0006091)  [fatty acid metabolic process](http://amigo.geneontology.org/cgi-bin/amigo/go.cgi?view=details&query=GO:0006631)  [transport](http://amigo.geneontology.org/cgi-bin/amigo/go.cgi?view=details&query=GO:0006810)  [acyltransferase activity](http://amigo.geneontology.org/cgi-bin/amigo/go.cgi?view=details&query=GO:0008415)  [energy derivation by oxidation of organic compounds](http://amigo.geneontology.org/cgi-bin/amigo/go.cgi?view=details&query=GO:0015980)  [membrane](http://amigo.geneontology.org/cgi-bin/amigo/go.cgi?view=details&query=GO:0016020)  [transferase activity](http://amigo.geneontology.org/cgi-bin/amigo/go.cgi?view=details&query=GO:0016740)  [fatty acid beta-oxidation using acyl-CoA oxidase](http://amigo.geneontology.org/cgi-bin/amigo/go.cgi?view=details&query=GO:0033540)  [fatty acid beta-oxidation using acyl-CoA oxidase](http://amigo.geneontology.org/cgi-bin/amigo/go.cgi?view=details&query=GO:0033540)  [cellular lipid metabolic process](http://amigo.geneontology.org/cgi-bin/amigo/go.cgi?view=details&query=GO:0044255) | [Peroxisome](http://www.genome.ad.jp/dbget-bin/show_pathway?MAP04146+2.3.1.7) |
| [209550_at](https://www.affymetrix.com/LinkServlet?&probeset=209550_at) | NDN | necdin homolog (mouse) | 15 | -23930561 | [U35139](http://www.ncbi.nlm.nih.gov/entrez/query.fcgi?cmd=search&db=nucleotide&term=U35139%5BACCN%5D&doptcmdl=GenBank) | [4692](http://www.ncbi.nlm.nih.gov/sites/entrez?Db=gene&Cmd=DetailsSearch&Term=4692) | [15q11.2-q12](http://www.ncbi.nlm.nih.gov/mapview/map_search.cgi?direct=on&idtype=gene&id=4692) | [Hs.50130](http://www.ncbi.nlm.nih.gov/UniGene/clust.cgi?ORG=Hs&CID=50130) | [32](http://www.ncbi.nih.gov/entrez/query.fcgi?tool=bioconductor&cmd=Retrieve&db=PubMed&list_uids=1394972%2C9302265%2C9354807%2C9422723%2C9630521%2C10347180%2C10915798%2C10965153%2C11439287%2C11813259%2C11959851%2C12198120%2C12414813%2C12477932%2C12716928%2C12913118%2C14593116%2C15247330%2C15489334%2C15978586%2C16169070%2C17207965%2C17353931%2C18272695%2C18660489%2C19058789%2C19386232%2C19517793%2C19626646%2C20029029%2C20538960%2C20665884) | [neuron migration](http://amigo.geneontology.org/cgi-bin/amigo/go.cgi?view=details&query=GO:0001764)  [DNA binding](http://amigo.geneontology.org/cgi-bin/amigo/go.cgi?view=details&query=GO:0003677)  [protein binding](http://amigo.geneontology.org/cgi-bin/amigo/go.cgi?view=details&query=GO:0005515)  [nucleus](http://amigo.geneontology.org/cgi-bin/amigo/go.cgi?view=details&query=GO:0005634)  [cytoplasm](http://amigo.geneontology.org/cgi-bin/amigo/go.cgi?view=details&query=GO:0005737)  [centrosome](http://amigo.geneontology.org/cgi-bin/amigo/go.cgi?view=details&query=GO:0005813)  [regulation of transcription, DNA-dependent](http://amigo.geneontology.org/cgi-bin/amigo/go.cgi?view=details&query=GO:0006355)  [nervous system development](http://amigo.geneontology.org/cgi-bin/amigo/go.cgi?view=details&query=GO:0007399)  [axonal fasciculation](http://amigo.geneontology.org/cgi-bin/amigo/go.cgi?view=details&query=GO:0007413)  [central nervous system development](http://amigo.geneontology.org/cgi-bin/amigo/go.cgi?view=details&query=GO:0007417)  [respiratory gaseous exchange](http://amigo.geneontology.org/cgi-bin/amigo/go.cgi?view=details&query=GO:0007585)  [negative regulation of cell proliferation](http://amigo.geneontology.org/cgi-bin/amigo/go.cgi?view=details&query=GO:0008285)  [glial cell migration](http://amigo.geneontology.org/cgi-bin/amigo/go.cgi?view=details&query=GO:0008347)  [sensory perception of pain](http://amigo.geneontology.org/cgi-bin/amigo/go.cgi?view=details&query=GO:0019233)  [regulation of growth](http://amigo.geneontology.org/cgi-bin/amigo/go.cgi?view=details&query=GO:0040008)  [gamma-tubulin binding](http://amigo.geneontology.org/cgi-bin/amigo/go.cgi?view=details&query=GO:0043015)  [perikaryon](http://amigo.geneontology.org/cgi-bin/amigo/go.cgi?view=details&query=GO:0043204)  [nerve growth factor receptor signaling pathway](http://amigo.geneontology.org/cgi-bin/amigo/go.cgi?view=details&query=GO:0048011)  [neuron development](http://amigo.geneontology.org/cgi-bin/amigo/go.cgi?view=details&query=GO:0048666)  [axon extension involved in development](http://amigo.geneontology.org/cgi-bin/amigo/go.cgi?view=details&query=GO:0048676) |  |
| [209598_at](https://www.affymetrix.com/LinkServlet?&probeset=209598_at) | PNMA2 | paraneoplastic antigen MA2 | 8 | -26362195 | [AB020690](http://www.ncbi.nlm.nih.gov/entrez/query.fcgi?cmd=search&db=nucleotide&term=AB020690%5BACCN%5D&doptcmdl=GenBank) | [10687](http://www.ncbi.nlm.nih.gov/sites/entrez?Db=gene&Cmd=DetailsSearch&Term=10687) | [8p21.2](http://www.ncbi.nlm.nih.gov/mapview/map_search.cgi?direct=on&idtype=gene&id=10687) | [Hs.591838](http://www.ncbi.nlm.nih.gov/UniGene/clust.cgi?ORG=Hs&CID=591838) | [14](http://www.ncbi.nih.gov/entrez/query.fcgi?tool=bioconductor&cmd=Retrieve&db=PubMed&list_uids=8889549%2C10048485%2C10050892%2C10362822%2C11558790%2C12477932%2C14702039%2C15489334%2C16214224%2C16344560%2C17207965%2C17353931%2C17474147%2C19596235) | [protein binding](http://amigo.geneontology.org/cgi-bin/amigo/go.cgi?view=details&query=GO:0005515)  [nucleus](http://amigo.geneontology.org/cgi-bin/amigo/go.cgi?view=details&query=GO:0005634)  [nucleolus](http://amigo.geneontology.org/cgi-bin/amigo/go.cgi?view=details&query=GO:0005730)  [apoptosis](http://amigo.geneontology.org/cgi-bin/amigo/go.cgi?view=details&query=GO:0006915) |  |
| [209706_at](https://www.affymetrix.com/LinkServlet?&probeset=209706_at) | NKX3-1 | NK3 homeobox 1 | 8 | -23536206 | [AF247704](http://www.ncbi.nlm.nih.gov/entrez/query.fcgi?cmd=search&db=nucleotide&term=AF247704%5BACCN%5D&doptcmdl=GenBank) | [4824](http://www.ncbi.nlm.nih.gov/sites/entrez?Db=gene&Cmd=DetailsSearch&Term=4824) | [8p21](http://www.ncbi.nlm.nih.gov/mapview/map_search.cgi?direct=on&idtype=gene&id=4824) | [Hs.55999](http://www.ncbi.nlm.nih.gov/UniGene/clust.cgi?ORG=Hs&CID=55999) | [62](http://www.ncbi.nih.gov/entrez/query.fcgi?tool=bioconductor&cmd=Retrieve&db=PubMed&list_uids=9226374%2C9377551%2C9537602%2C10559189%2C10993896%2C11085535%2C11137288%2C11809674%2C11980664%2C12450213%2C12477932%2C12661036%2C14633588%2C14648854%2C15311057%2C15489334%2C15523673%2C15691383%2C15734999%2C15880262%2C16201967%2C16382041%2C16397218%2C16413692%2C16442598%2C16519150%2C16581776%2C16697957%2C16814806%2C16817226%2C16845664%2C17108105%2C17202838%2C17234752%2C17311278%2C17486276%2C18077445%2C18296735%2C18360715%2C18454873%2C18757402%2C18794125%2C18974119%2C19258508%2C19263243%2C19266349%2C19453261%2C19462257%2C19767753%2C19780584%2C19797053%2C19886863%2C19915572%2C20363913%2C20395202%2C20479932%2C20564319%2C20588175%2C20676098%2C20716579%2C20842667%2C20855495) | [transcription regulatory region sequence-specific DNA binding](http://amigo.geneontology.org/cgi-bin/amigo/go.cgi?view=details&query=GO:0000976)  [sequence-specific DNA binding transcription factor activity](http://amigo.geneontology.org/cgi-bin/amigo/go.cgi?view=details&query=GO:0003700)  [sequence-specific DNA binding transcription factor activity](http://amigo.geneontology.org/cgi-bin/amigo/go.cgi?view=details&query=GO:0003700)  [protein binding](http://amigo.geneontology.org/cgi-bin/amigo/go.cgi?view=details&query=GO:0005515)  [nucleus](http://amigo.geneontology.org/cgi-bin/amigo/go.cgi?view=details&query=GO:0005634)  [nucleus](http://amigo.geneontology.org/cgi-bin/amigo/go.cgi?view=details&query=GO:0005634)  [transcription, DNA-dependent](http://amigo.geneontology.org/cgi-bin/amigo/go.cgi?view=details&query=GO:0006351)  [transcription, DNA-dependent](http://amigo.geneontology.org/cgi-bin/amigo/go.cgi?view=details&query=GO:0006351)  [regulation of transcription, DNA-dependent](http://amigo.geneontology.org/cgi-bin/amigo/go.cgi?view=details&query=GO:0006355)  [multicellular organismal development](http://amigo.geneontology.org/cgi-bin/amigo/go.cgi?view=details&query=GO:0007275)  [salivary gland development](http://amigo.geneontology.org/cgi-bin/amigo/go.cgi?view=details&query=GO:0007431)  [transcription factor binding](http://amigo.geneontology.org/cgi-bin/amigo/go.cgi?view=details&query=GO:0008134)  [positive regulation of gene-specific transcription from RNA polymerase II promoter](http://amigo.geneontology.org/cgi-bin/amigo/go.cgi?view=details&query=GO:0010552)  [transcription activator activity](http://amigo.geneontology.org/cgi-bin/amigo/go.cgi?view=details&query=GO:0016563)  [transcription repressor activity](http://amigo.geneontology.org/cgi-bin/amigo/go.cgi?view=details&query=GO:0016564)  [estrogen receptor activity](http://amigo.geneontology.org/cgi-bin/amigo/go.cgi?view=details&query=GO:0030284)  [estrogen receptor binding](http://amigo.geneontology.org/cgi-bin/amigo/go.cgi?view=details&query=GO:0030331)  [estrogen receptor signaling pathway](http://amigo.geneontology.org/cgi-bin/amigo/go.cgi?view=details&query=GO:0030520)  [negative regulation of gene-specific transcription](http://amigo.geneontology.org/cgi-bin/amigo/go.cgi?view=details&query=GO:0032582)  [negative regulation of insulin-like growth factor receptor signaling pathway](http://amigo.geneontology.org/cgi-bin/amigo/go.cgi?view=details&query=GO:0043569)  [positive regulation of mitotic cell cycle](http://amigo.geneontology.org/cgi-bin/amigo/go.cgi?view=details&query=GO:0045931)  [branching morphogenesis of a tube](http://amigo.geneontology.org/cgi-bin/amigo/go.cgi?view=details&query=GO:0048754)  [positive regulation of cell division](http://amigo.geneontology.org/cgi-bin/amigo/go.cgi?view=details&query=GO:0051781)  [branching involved in prostate gland morphogenesis](http://amigo.geneontology.org/cgi-bin/amigo/go.cgi?view=details&query=GO:0060442)  [epithelial cell proliferation involved in salivary gland morphogenesis](http://amigo.geneontology.org/cgi-bin/amigo/go.cgi?view=details&query=GO:0060664)  [negative regulation of epithelial cell proliferation involved in prostate gland development](http://amigo.geneontology.org/cgi-bin/amigo/go.cgi?view=details&query=GO:0060770)  [negative regulation of estrogen receptor binding](http://amigo.geneontology.org/cgi-bin/amigo/go.cgi?view=details&query=GO:0071899) | [Pathways in cancer](http://www.genome.ad.jp/kegg/pathway/hsa/hsa05200.html)  [Prostate cancer](http://www.genome.ad.jp/kegg/pathway/hsa/hsa05215.html) |
| [209771_x_at](https://www.affymetrix.com/LinkServlet?&probeset=209771_x_at) | CD24 | CD24 molecule | 6 | -21152525 | [AA761181](http://www.ncbi.nlm.nih.gov/entrez/query.fcgi?cmd=search&db=nucleotide&term=AA761181%5BACCN%5D&doptcmdl=GenBank) | [100133941](http://www.ncbi.nlm.nih.gov/sites/entrez?Db=gene&Cmd=DetailsSearch&Term=100133941) | [6q21](http://www.ncbi.nlm.nih.gov/mapview/map_search.cgi?direct=on&idtype=gene&id=100133941) | [Hs.644105](http://www.ncbi.nlm.nih.gov/UniGene/clust.cgi?ORG=Hs&CID=644105) [Hs.721443](http://www.ncbi.nlm.nih.gov/UniGene/clust.cgi?ORG=Hs&CID=721443) | [90](http://www.ncbi.nih.gov/entrez/query.fcgi?tool=bioconductor&cmd=Retrieve&db=PubMed&list_uids=1327504%2C1831224%2C2153173%2C7553654%2C7736776%2C7959762%2C8213086%2C8223854%2C8753773%2C8928617%2C9129046%2C10037815%2C10575223%2C11272271%2C11313396%2C12218294%2C12368195%2C12447971%2C12477932%2C12496407%2C12610508%2C12829373%2C14657362%2C14702039%2C15174142%2C15489334%2C15493995%2C15616015%2C15633604%2C16125303%2C16164042%2C16166435%2C16288985%2C16390867%2C16532032%2C16621031%2C16631259%2C16681720%2C16890615%2C16892043%2C16900767%2C16930538%2C17411341%2C17475624%2C17540049%2C17700640%2C17763438%2C17900673%2C17944116%2C17950993%2C17980703%2C18381780%2C18384848%2C18404683%2C18413748%2C18417991%2C18495204%2C18559090%2C18566397%2C18632604%2C18752058%2C19043399%2C19050962%2C19072375%2C19130400%2C19243896%2C19610054%2C19706825%2C19725119%2C19786366%2C19787233%2C19794958%2C19860845%2C19896210%2C19906290%2C19946098%2C19998456%2C20019840%2C20177845%2C20199686%2C20230526%2C20336055%2C20354454%2C20378664%2C20491779%2C20619441%2C20621328%2C20628624%2C21041728%2C21086907) | [response to hypoxia](http://amigo.geneontology.org/cgi-bin/amigo/go.cgi?view=details&query=GO:0001666)  [cell activation](http://amigo.geneontology.org/cgi-bin/amigo/go.cgi?view=details&query=GO:0001775)  [regulation of cytokine-mediated signaling pathway](http://amigo.geneontology.org/cgi-bin/amigo/go.cgi?view=details&query=GO:0001959)  [response to molecule of bacterial origin](http://amigo.geneontology.org/cgi-bin/amigo/go.cgi?view=details&query=GO:0002237)  [immune response-regulating cell surface receptor signaling pathway](http://amigo.geneontology.org/cgi-bin/amigo/go.cgi?view=details&query=GO:0002768)  [signal transducer activity](http://amigo.geneontology.org/cgi-bin/amigo/go.cgi?view=details&query=GO:0004871)  [protein binding](http://amigo.geneontology.org/cgi-bin/amigo/go.cgi?view=details&query=GO:0005515)  [plasma membrane](http://amigo.geneontology.org/cgi-bin/amigo/go.cgi?view=details&query=GO:0005886)  [elevation of cytosolic calcium ion concentration](http://amigo.geneontology.org/cgi-bin/amigo/go.cgi?view=details&query=GO:0007204)  [axon guidance](http://amigo.geneontology.org/cgi-bin/amigo/go.cgi?view=details&query=GO:0007411)  [induction of apoptosis by intracellular signals](http://amigo.geneontology.org/cgi-bin/amigo/go.cgi?view=details&query=GO:0008629)  [cell surface](http://amigo.geneontology.org/cgi-bin/amigo/go.cgi?view=details&query=GO:0009986)  [membrane](http://amigo.geneontology.org/cgi-bin/amigo/go.cgi?view=details&query=GO:0016020)  [Wnt receptor signaling pathway](http://amigo.geneontology.org/cgi-bin/amigo/go.cgi?view=details&query=GO:0016055)  [cell-cell adhesion](http://amigo.geneontology.org/cgi-bin/amigo/go.cgi?view=details&query=GO:0016337)  [cell migration](http://amigo.geneontology.org/cgi-bin/amigo/go.cgi?view=details&query=GO:0016477)  [protein kinase binding](http://amigo.geneontology.org/cgi-bin/amigo/go.cgi?view=details&query=GO:0019901)  [protein tyrosine kinase activator activity](http://amigo.geneontology.org/cgi-bin/amigo/go.cgi?view=details&query=GO:0030296)  [regulation of epithelial cell differentiation](http://amigo.geneontology.org/cgi-bin/amigo/go.cgi?view=details&query=GO:0030856)  [anchored to membrane](http://amigo.geneontology.org/cgi-bin/amigo/go.cgi?view=details&query=GO:0031225)  [T cell costimulation](http://amigo.geneontology.org/cgi-bin/amigo/go.cgi?view=details&query=GO:0031295)  [B cell receptor transport into membrane raft](http://amigo.geneontology.org/cgi-bin/amigo/go.cgi?view=details&query=GO:0032597)  [chemokine receptor transport out of membrane raft](http://amigo.geneontology.org/cgi-bin/amigo/go.cgi?view=details&query=GO:0032600)  [negative regulation of transforming growth factor-beta3 production](http://amigo.geneontology.org/cgi-bin/amigo/go.cgi?view=details&query=GO:0032913)  [positive regulation of activated T cell proliferation](http://amigo.geneontology.org/cgi-bin/amigo/go.cgi?view=details&query=GO:0042104)  [regulation of phosphorylation](http://amigo.geneontology.org/cgi-bin/amigo/go.cgi?view=details&query=GO:0042325)  [cholesterol homeostasis](http://amigo.geneontology.org/cgi-bin/amigo/go.cgi?view=details&query=GO:0042632)  [positive regulation of MAP kinase activity](http://amigo.geneontology.org/cgi-bin/amigo/go.cgi?view=details&query=GO:0043406)  [regulation of MAPKKK cascade](http://amigo.geneontology.org/cgi-bin/amigo/go.cgi?view=details&query=GO:0043408)  [response to estrogen stimulus](http://amigo.geneontology.org/cgi-bin/amigo/go.cgi?view=details&query=GO:0043627)  [membrane raft](http://amigo.geneontology.org/cgi-bin/amigo/go.cgi?view=details&query=GO:0045121)  [respiratory burst](http://amigo.geneontology.org/cgi-bin/amigo/go.cgi?view=details&query=GO:0045730)  [positive regulation of protein tyrosine kinase activity](http://amigo.geneontology.org/cgi-bin/amigo/go.cgi?view=details&query=GO:0061098) | [Hematopoietic cell lineage](http://www.genome.ad.jp/kegg/pathway/hsa/hsa04640.html) |
| [210074_at](https://www.affymetrix.com/LinkServlet?&probeset=210074_at) | CTSL2 | cathepsin L2 | 9 | -99794939 | [AF070448](http://www.ncbi.nlm.nih.gov/entrez/query.fcgi?cmd=search&db=nucleotide&term=AF070448%5BACCN%5D&doptcmdl=GenBank) | [1515](http://www.ncbi.nlm.nih.gov/sites/entrez?Db=gene&Cmd=DetailsSearch&Term=1515) | [9q22.2](http://www.ncbi.nlm.nih.gov/mapview/map_search.cgi?direct=on&idtype=gene&id=1515) | [Hs.610096](http://www.ncbi.nlm.nih.gov/UniGene/clust.cgi?ORG=Hs&CID=610096) [Hs.621851](http://www.ncbi.nlm.nih.gov/UniGene/clust.cgi?ORG=Hs&CID=621851) | [19](http://www.ncbi.nih.gov/entrez/query.fcgi?tool=bioconductor&cmd=Retrieve&db=PubMed&list_uids=9563472%2C9727401%2C10029531%2C10382972%2C11027133%2C12477932%2C12648222%2C12925692%2C15164053%2C15192101%2C15489334%2C15679121%2C16565075%2C17516850%2C17869649%2C17889653%2C18163891%2C20347002%2C20536394) | [aminopeptidase activity](http://amigo.geneontology.org/cgi-bin/amigo/go.cgi?view=details&query=GO:0004177)  [cysteine-type endopeptidase activity](http://amigo.geneontology.org/cgi-bin/amigo/go.cgi?view=details&query=GO:0004197)  [protein binding](http://amigo.geneontology.org/cgi-bin/amigo/go.cgi?view=details&query=GO:0005515)  [soluble fraction](http://amigo.geneontology.org/cgi-bin/amigo/go.cgi?view=details&query=GO:0005625)  [cytoplasm](http://amigo.geneontology.org/cgi-bin/amigo/go.cgi?view=details&query=GO:0005737)  [lysosome](http://amigo.geneontology.org/cgi-bin/amigo/go.cgi?view=details&query=GO:0005764)  [vacuole](http://amigo.geneontology.org/cgi-bin/amigo/go.cgi?view=details&query=GO:0005773)  [microvillus](http://amigo.geneontology.org/cgi-bin/amigo/go.cgi?view=details&query=GO:0005902)  [proteolysis](http://amigo.geneontology.org/cgi-bin/amigo/go.cgi?view=details&query=GO:0006508)  [cell communication](http://amigo.geneontology.org/cgi-bin/amigo/go.cgi?view=details&query=GO:0007154)  [spermatogenesis](http://amigo.geneontology.org/cgi-bin/amigo/go.cgi?view=details&query=GO:0007283)  [peptidase activity](http://amigo.geneontology.org/cgi-bin/amigo/go.cgi?view=details&query=GO:0008233)  [cysteine-type peptidase activity](http://amigo.geneontology.org/cgi-bin/amigo/go.cgi?view=details&query=GO:0008234)  [cellular response to starvation](http://amigo.geneontology.org/cgi-bin/amigo/go.cgi?view=details&query=GO:0009267)  [response to glucose stimulus](http://amigo.geneontology.org/cgi-bin/amigo/go.cgi?view=details&query=GO:0009749)  [external side of plasma membrane](http://amigo.geneontology.org/cgi-bin/amigo/go.cgi?view=details&query=GO:0009897)  [multicellular organismal aging](http://amigo.geneontology.org/cgi-bin/amigo/go.cgi?view=details&query=GO:0010259)  [response to organic cyclic compound](http://amigo.geneontology.org/cgi-bin/amigo/go.cgi?view=details&query=GO:0014070)  [nerve development](http://amigo.geneontology.org/cgi-bin/amigo/go.cgi?view=details&query=GO:0021675)  [stored secretory granule](http://amigo.geneontology.org/cgi-bin/amigo/go.cgi?view=details&query=GO:0030141)  [kininogen binding](http://amigo.geneontology.org/cgi-bin/amigo/go.cgi?view=details&query=GO:0030984)  [protein complex binding](http://amigo.geneontology.org/cgi-bin/amigo/go.cgi?view=details&query=GO:0032403)  [response to gonadotropin stimulus](http://amigo.geneontology.org/cgi-bin/amigo/go.cgi?view=details&query=GO:0034698)  [peptide binding](http://amigo.geneontology.org/cgi-bin/amigo/go.cgi?view=details&query=GO:0042277)  [neuron projection](http://amigo.geneontology.org/cgi-bin/amigo/go.cgi?view=details&query=GO:0043005)  [perikaryon](http://amigo.geneontology.org/cgi-bin/amigo/go.cgi?view=details&query=GO:0043204)  [apical part of cell](http://amigo.geneontology.org/cgi-bin/amigo/go.cgi?view=details&query=GO:0045177)  [decidualization](http://amigo.geneontology.org/cgi-bin/amigo/go.cgi?view=details&query=GO:0046697)  [autophagic cell death](http://amigo.geneontology.org/cgi-bin/amigo/go.cgi?view=details&query=GO:0048102)  [response to glucocorticoid stimulus](http://amigo.geneontology.org/cgi-bin/amigo/go.cgi?view=details&query=GO:0051384)  [response to protein stimulus](http://amigo.geneontology.org/cgi-bin/amigo/go.cgi?view=details&query=GO:0051789)  [Sertoli cell differentiation](http://amigo.geneontology.org/cgi-bin/amigo/go.cgi?view=details&query=GO:0060008) | [Lysosome](http://www.genome.ad.jp/dbget-bin/show_pathway?MAP04142+3.4.22.43) |
| [210253_at](https://www.affymetrix.com/LinkServlet?&probeset=210253_at) | HTATIP2 | HIV-1 Tat interactive protein 2, 30kDa | 11 | 20385230, 20385246, 20385288, 20385402, 20385686 | [AF092095](http://www.ncbi.nlm.nih.gov/entrez/query.fcgi?cmd=search&db=nucleotide&term=AF092095%5BACCN%5D&doptcmdl=GenBank) | [10553](http://www.ncbi.nlm.nih.gov/sites/entrez?Db=gene&Cmd=DetailsSearch&Term=10553) | [11p15.1](http://www.ncbi.nlm.nih.gov/mapview/map_search.cgi?direct=on&idtype=gene&id=10553) | [Hs.90753](http://www.ncbi.nlm.nih.gov/UniGene/clust.cgi?ORG=Hs&CID=90753) | [35](http://www.ncbi.nih.gov/entrez/query.fcgi?tool=bioconductor&cmd=Retrieve&db=PubMed&list_uids=8125298%2C9174052%2C9373149%2C9482853%2C10395547%2C10611237%2C10698937%2C10892349%2C11313954%2C12477932%2C14695192%2C15073177%2C15124103%2C15282309%2C15342556%2C15489334%2C15493507%2C15633220%2C15728189%2C16615932%2C16799960%2C17097132%2C17997990%2C18029348%2C18519672%2C18528861%2C18537194%2C18972434%2C19010857%2C19027726%2C19104151%2C19349353%2C19798571%2C19839715%2C20374651) | [RNA polymerase II transcription factor activity](http://amigo.geneontology.org/cgi-bin/amigo/go.cgi?view=details&query=GO:0003702)  [transcription coactivator activity](http://amigo.geneontology.org/cgi-bin/amigo/go.cgi?view=details&query=GO:0003713)  [protein binding](http://amigo.geneontology.org/cgi-bin/amigo/go.cgi?view=details&query=GO:0005515)  [nucleus](http://amigo.geneontology.org/cgi-bin/amigo/go.cgi?view=details&query=GO:0005634)  [nuclear envelope](http://amigo.geneontology.org/cgi-bin/amigo/go.cgi?view=details&query=GO:0005635)  [cytoplasm](http://amigo.geneontology.org/cgi-bin/amigo/go.cgi?view=details&query=GO:0005737)  [regulation of transcription from RNA polymerase II promoter](http://amigo.geneontology.org/cgi-bin/amigo/go.cgi?view=details&query=GO:0006357)  [cellular amino acid metabolic process](http://amigo.geneontology.org/cgi-bin/amigo/go.cgi?view=details&query=GO:0006520)  [anti-apoptosis](http://amigo.geneontology.org/cgi-bin/amigo/go.cgi?view=details&query=GO:0006916)  [induction of apoptosis](http://amigo.geneontology.org/cgi-bin/amigo/go.cgi?view=details&query=GO:0006917)  [multicellular organismal development](http://amigo.geneontology.org/cgi-bin/amigo/go.cgi?view=details&query=GO:0007275)  [oxidoreductase activity](http://amigo.geneontology.org/cgi-bin/amigo/go.cgi?view=details&query=GO:0016491)  [oxidoreductase activity, acting on the aldehyde or oxo group of donors, NAD or NADP as acceptor](http://amigo.geneontology.org/cgi-bin/amigo/go.cgi?view=details&query=GO:0016620)  [cell differentiation](http://amigo.geneontology.org/cgi-bin/amigo/go.cgi?view=details&query=GO:0030154)  [regulation of apoptosis](http://amigo.geneontology.org/cgi-bin/amigo/go.cgi?view=details&query=GO:0042981)  [interspecies interaction between organisms](http://amigo.geneontology.org/cgi-bin/amigo/go.cgi?view=details&query=GO:0044419)  [regulation of angiogenesis](http://amigo.geneontology.org/cgi-bin/amigo/go.cgi?view=details&query=GO:0045765)  [positive regulation of transcription](http://amigo.geneontology.org/cgi-bin/amigo/go.cgi?view=details&query=GO:0045941)  [nuclear import](http://amigo.geneontology.org/cgi-bin/amigo/go.cgi?view=details&query=GO:0051170)  [NAD binding](http://amigo.geneontology.org/cgi-bin/amigo/go.cgi?view=details&query=GO:0051287)  [oxidation-reduction process](http://amigo.geneontology.org/cgi-bin/amigo/go.cgi?view=details&query=GO:0055114) |  |
| [210432_s_at](https://www.affymetrix.com/LinkServlet?&probeset=210432_s_at) | SCN3A | sodium channel, voltage-gated, type III, alpha subunit | 2 | -165944031 | [AF225986](http://www.ncbi.nlm.nih.gov/entrez/query.fcgi?cmd=search&db=nucleotide&term=AF225986%5BACCN%5D&doptcmdl=GenBank) | [6328](http://www.ncbi.nlm.nih.gov/sites/entrez?Db=gene&Cmd=DetailsSearch&Term=6328) | [2q24](http://www.ncbi.nlm.nih.gov/mapview/map_search.cgi?direct=on&idtype=gene&id=6328) | [Hs.435274](http://www.ncbi.nlm.nih.gov/UniGene/clust.cgi?ORG=Hs&CID=435274) | [19](http://www.ncbi.nih.gov/entrez/query.fcgi?tool=bioconductor&cmd=Retrieve&db=PubMed&list_uids=8159690%2C8889548%2C9589372%2C10718198%2C10827173%2C11122339%2C11245985%2C11566500%2C12610651%2C15317864%2C16029190%2C16052353%2C16344560%2C16382098%2C17544618%2C18784617%2C19699781%2C20346423%2C20379614) | [voltage-gated sodium channel complex](http://amigo.geneontology.org/cgi-bin/amigo/go.cgi?view=details&query=GO:0001518)  [voltage-gated ion channel activity](http://amigo.geneontology.org/cgi-bin/amigo/go.cgi?view=details&query=GO:0005244)  [voltage-gated sodium channel activity](http://amigo.geneontology.org/cgi-bin/amigo/go.cgi?view=details&query=GO:0005248)  [ion transport](http://amigo.geneontology.org/cgi-bin/amigo/go.cgi?view=details&query=GO:0006811)  [sodium ion transport](http://amigo.geneontology.org/cgi-bin/amigo/go.cgi?view=details&query=GO:0006814)  [membrane](http://amigo.geneontology.org/cgi-bin/amigo/go.cgi?view=details&query=GO:0016020)  [integral to membrane](http://amigo.geneontology.org/cgi-bin/amigo/go.cgi?view=details&query=GO:0016021)  [transmembrane transport](http://amigo.geneontology.org/cgi-bin/amigo/go.cgi?view=details&query=GO:0055085) |  |
| [210835_s_at](https://www.affymetrix.com/LinkServlet?&probeset=210835_s_at) | CTBP2 | C-terminal binding protein 2 | 10 | -126676418, -126676418, -126676418 | [AF222711](http://www.ncbi.nlm.nih.gov/entrez/query.fcgi?cmd=search&db=nucleotide&term=AF222711%5BACCN%5D&doptcmdl=GenBank) | [1488](http://www.ncbi.nlm.nih.gov/sites/entrez?Db=gene&Cmd=DetailsSearch&Term=1488) | [10q26.13](http://www.ncbi.nlm.nih.gov/mapview/map_search.cgi?direct=on&idtype=gene&id=1488) | [Hs.501345](http://www.ncbi.nlm.nih.gov/UniGene/clust.cgi?ORG=Hs&CID=501345) | [54](http://www.ncbi.nih.gov/entrez/query.fcgi?tool=bioconductor&cmd=Retrieve&db=PubMed&list_uids=7479821%2C9479502%2C9724649%2C9858600%2C10359772%2C10438528%2C10567582%2C10756197%2C10764811%2C11163272%2C11504872%2C11864595%2C12477932%2C12535528%2C12556451%2C12711682%2C12714599%2C12867035%2C15060175%2C15146197%2C15489334%2C15542832%2C16189514%2C16356938%2C16385451%2C16702210%2C16787403%2C17023432%2C17546044%2C18184656%2C18264096%2C18794092%2C19240061%2C19318432%2C19366831%2C19423541%2C19486893%2C19506021%2C19549807%2C19668232%2C19754958%2C19798104%2C19866473%2C19900942%2C19902474%2C20379614%2C20450899%2C20460480%2C20523059%2C20564319%2C20690139%2C20717903%2C20878950%2C21071540) | [protein binding](http://amigo.geneontology.org/cgi-bin/amigo/go.cgi?view=details&query=GO:0005515)  [nucleus](http://amigo.geneontology.org/cgi-bin/amigo/go.cgi?view=details&query=GO:0005634)  [negative regulation of cell proliferation](http://amigo.geneontology.org/cgi-bin/amigo/go.cgi?view=details&query=GO:0008285)  [negative regulation of transcription](http://amigo.geneontology.org/cgi-bin/amigo/go.cgi?view=details&query=GO:0016481)  [oxidoreductase activity](http://amigo.geneontology.org/cgi-bin/amigo/go.cgi?view=details&query=GO:0016491)  [transcription repressor activity](http://amigo.geneontology.org/cgi-bin/amigo/go.cgi?view=details&query=GO:0016564)  [oxidoreductase activity, acting on the CH-OH group of donors, NAD or NADP as acceptor](http://amigo.geneontology.org/cgi-bin/amigo/go.cgi?view=details&query=GO:0016616)  [transcriptional repressor complex](http://amigo.geneontology.org/cgi-bin/amigo/go.cgi?view=details&query=GO:0017053)  [viral genome replication](http://amigo.geneontology.org/cgi-bin/amigo/go.cgi?view=details&query=GO:0019079)  [cell junction](http://amigo.geneontology.org/cgi-bin/amigo/go.cgi?view=details&query=GO:0030054)  [cell differentiation](http://amigo.geneontology.org/cgi-bin/amigo/go.cgi?view=details&query=GO:0030154)  [synapse](http://amigo.geneontology.org/cgi-bin/amigo/go.cgi?view=details&query=GO:0045202)  [cofactor binding](http://amigo.geneontology.org/cgi-bin/amigo/go.cgi?view=details&query=GO:0048037)  [white fat cell differentiation](http://amigo.geneontology.org/cgi-bin/amigo/go.cgi?view=details&query=GO:0050872)  [NAD binding](http://amigo.geneontology.org/cgi-bin/amigo/go.cgi?view=details&query=GO:0051287)  [oxidation-reduction process](http://amigo.geneontology.org/cgi-bin/amigo/go.cgi?view=details&query=GO:0055114) | [Wnt signaling pathway](http://www.genome.ad.jp/kegg/pathway/hsa/hsa04310.html)  [Notch signaling pathway](http://www.genome.ad.jp/kegg/pathway/hsa/hsa04330.html)  [Pathways in cancer](http://www.genome.ad.jp/kegg/pathway/hsa/hsa05200.html)  [Chronic myeloid leukemia](http://www.genome.ad.jp/kegg/pathway/hsa/hsa05220.html) |
| [211024_s_at](https://www.affymetrix.com/LinkServlet?&probeset=211024_s_at) | NKX2-1 | NK2 homeobox 1 | 14 | -36985605, -36985605 | [BC006221](http://www.ncbi.nlm.nih.gov/entrez/query.fcgi?cmd=search&db=nucleotide&term=BC006221%5BACCN%5D&doptcmdl=GenBank) | [7080](http://www.ncbi.nlm.nih.gov/sites/entrez?Db=gene&Cmd=DetailsSearch&Term=7080) | [14q13](http://www.ncbi.nlm.nih.gov/mapview/map_search.cgi?direct=on&idtype=gene&id=7080) | [Hs.94367](http://www.ncbi.nlm.nih.gov/UniGene/clust.cgi?ORG=Hs&CID=94367) | [108](http://www.ncbi.nih.gov/entrez/query.fcgi?tool=bioconductor&cmd=Retrieve&db=PubMed&list_uids=1735431%2C1976511%2C7559607%2C7635972%2C7711079%2C7711080%2C7713914%2C8675988%2C8889548%2C9396717%2C9545595%2C9582279%2C9988700%2C10617585%2C10733581%2C11076796%2C11152647%2C11274148%2C11438542%2C11713256%2C11733512%2C11836702%2C11854318%2C11854319%2C11923479%2C11953175%2C11957142%2C11971878%2C12040027%2C12051643%2C12161428%2C12408771%2C12441357%2C12477932%2C12499091%2C12684771%2C12923324%2C14633512%2C14720435%2C14960358%2C14970209%2C15098009%2C15173172%2C15271884%2C15279903%2C15449938%2C15485815%2C15489334%2C15548547%2C15929662%2C15955952%2C16220345%2C16260629%2C16314749%2C16461352%2C16565516%2C16613858%2C16630564%2C16960125%2C16980598%2C17044090%2C17182767%2C17220277%2C17412341%2C17413979%2C17474147%2C17616654%2C17640327%2C17671725%2C17702043%2C17765926%2C18059234%2C18071837%2C18212743%2C18239190%2C18379122%2C18391950%2C18487360%2C18661567%2C18682709%2C18788921%2C18855882%2C18957494%2C18958156%2C18997617%2C19011567%2C19047914%2C19064983%2C19176457%2C19198613%2C19293183%2C19329538%2C19336474%2C19365834%2C19483637%2C19506552%2C19525896%2C19578049%2C19730683%2C19740516%2C19906647%2C20020530%2C20042854%2C20157192%2C20518411%2C20701785%2C20734064%2C20830690) | [negative regulation of transcription from RNA polymerase II promoter](http://amigo.geneontology.org/cgi-bin/amigo/go.cgi?view=details&query=GO:0000122)  [neuron migration](http://amigo.geneontology.org/cgi-bin/amigo/go.cgi?view=details&query=GO:0001764)  [regulation of blood volume by renin-angiotensin](http://amigo.geneontology.org/cgi-bin/amigo/go.cgi?view=details&query=GO:0002016)  [DNA binding](http://amigo.geneontology.org/cgi-bin/amigo/go.cgi?view=details&query=GO:0003677)  [sequence-specific DNA binding transcription factor activity](http://amigo.geneontology.org/cgi-bin/amigo/go.cgi?view=details&query=GO:0003700)  [specific RNA polymerase II transcription factor activity](http://amigo.geneontology.org/cgi-bin/amigo/go.cgi?view=details&query=GO:0003704)  [sequence-specific enhancer binding RNA polymerase II transcription factor activity](http://amigo.geneontology.org/cgi-bin/amigo/go.cgi?view=details&query=GO:0003705)  [protein binding](http://amigo.geneontology.org/cgi-bin/amigo/go.cgi?view=details&query=GO:0005515)  [soluble fraction](http://amigo.geneontology.org/cgi-bin/amigo/go.cgi?view=details&query=GO:0005625)  [nucleus](http://amigo.geneontology.org/cgi-bin/amigo/go.cgi?view=details&query=GO:0005634)  [nucleoplasm](http://amigo.geneontology.org/cgi-bin/amigo/go.cgi?view=details&query=GO:0005654)  [transcription factor complex](http://amigo.geneontology.org/cgi-bin/amigo/go.cgi?view=details&query=GO:0005667)  [transcription, DNA-dependent](http://amigo.geneontology.org/cgi-bin/amigo/go.cgi?view=details&query=GO:0006351)  [phospholipid metabolic process](http://amigo.geneontology.org/cgi-bin/amigo/go.cgi?view=details&query=GO:0006644)  [pattern specification process](http://amigo.geneontology.org/cgi-bin/amigo/go.cgi?view=details&query=GO:0007389)  [axon guidance](http://amigo.geneontology.org/cgi-bin/amigo/go.cgi?view=details&query=GO:0007411)  [brain development](http://amigo.geneontology.org/cgi-bin/amigo/go.cgi?view=details&query=GO:0007420)  [endoderm development](http://amigo.geneontology.org/cgi-bin/amigo/go.cgi?view=details&query=GO:0007492)  [locomotory behavior](http://amigo.geneontology.org/cgi-bin/amigo/go.cgi?view=details&query=GO:0007626)  [feeding behavior](http://amigo.geneontology.org/cgi-bin/amigo/go.cgi?view=details&query=GO:0007631)  [response to hormone stimulus](http://amigo.geneontology.org/cgi-bin/amigo/go.cgi?view=details&query=GO:0009725)  [organ morphogenesis](http://amigo.geneontology.org/cgi-bin/amigo/go.cgi?view=details&query=GO:0009887)  [positive regulation of gene-specific transcription from RNA polymerase II promoter](http://amigo.geneontology.org/cgi-bin/amigo/go.cgi?view=details&query=GO:0010552)  [positive regulation of gene expression](http://amigo.geneontology.org/cgi-bin/amigo/go.cgi?view=details&query=GO:0010628)  [negative regulation of epithelial to mesenchymal transition](http://amigo.geneontology.org/cgi-bin/amigo/go.cgi?view=details&query=GO:0010719)  [promoter binding](http://amigo.geneontology.org/cgi-bin/amigo/go.cgi?view=details&query=GO:0010843)  [transcription activator activity](http://amigo.geneontology.org/cgi-bin/amigo/go.cgi?view=details&query=GO:0016563)  [globus pallidus development](http://amigo.geneontology.org/cgi-bin/amigo/go.cgi?view=details&query=GO:0021759)  [hippocampus development](http://amigo.geneontology.org/cgi-bin/amigo/go.cgi?view=details&query=GO:0021766)  [cerebral cortex cell migration](http://amigo.geneontology.org/cgi-bin/amigo/go.cgi?view=details&query=GO:0021795)  [forebrain dorsal/ventral pattern formation](http://amigo.geneontology.org/cgi-bin/amigo/go.cgi?view=details&query=GO:0021798)  [forebrain neuron fate commitment](http://amigo.geneontology.org/cgi-bin/amigo/go.cgi?view=details&query=GO:0021877)  [cerebral cortex GABAergic interneuron differentiation](http://amigo.geneontology.org/cgi-bin/amigo/go.cgi?view=details&query=GO:0021892)  [pituitary gland development](http://amigo.geneontology.org/cgi-bin/amigo/go.cgi?view=details&query=GO:0021983)  [lung development](http://amigo.geneontology.org/cgi-bin/amigo/go.cgi?view=details&query=GO:0030324)  [negative regulation of cell migration](http://amigo.geneontology.org/cgi-bin/amigo/go.cgi?view=details&query=GO:0030336)  [negative regulation of transforming growth factor beta receptor signaling pathway](http://amigo.geneontology.org/cgi-bin/amigo/go.cgi?view=details&query=GO:0030512)  [thyroid gland development](http://amigo.geneontology.org/cgi-bin/amigo/go.cgi?view=details&query=GO:0030878)  [developmental induction](http://amigo.geneontology.org/cgi-bin/amigo/go.cgi?view=details&query=GO:0031128)  [response to lipopolysaccharide](http://amigo.geneontology.org/cgi-bin/amigo/go.cgi?view=details&query=GO:0032496)  [Leydig cell differentiation](http://amigo.geneontology.org/cgi-bin/amigo/go.cgi?view=details&query=GO:0033327)  [hyperosmotic salinity response](http://amigo.geneontology.org/cgi-bin/amigo/go.cgi?view=details&query=GO:0042538)  [menarche](http://amigo.geneontology.org/cgi-bin/amigo/go.cgi?view=details&query=GO:0042696)  [response to ethanol](http://amigo.geneontology.org/cgi-bin/amigo/go.cgi?view=details&query=GO:0045471)  [positive regulation of transcription, DNA-dependent](http://amigo.geneontology.org/cgi-bin/amigo/go.cgi?view=details&query=GO:0045893)  [positive regulation of transcription, DNA-dependent](http://amigo.geneontology.org/cgi-bin/amigo/go.cgi?view=details&query=GO:0045893)  [positive regulation of transcription from RNA polymerase II promoter](http://amigo.geneontology.org/cgi-bin/amigo/go.cgi?view=details&query=GO:0045944)  [development of primary female sexual characteristics](http://amigo.geneontology.org/cgi-bin/amigo/go.cgi?view=details&query=GO:0046545)  [anatomical structure formation involved in morphogenesis](http://amigo.geneontology.org/cgi-bin/amigo/go.cgi?view=details&query=GO:0048646)  [oligodendrocyte differentiation](http://amigo.geneontology.org/cgi-bin/amigo/go.cgi?view=details&query=GO:0048709)  [lung saccule development](http://amigo.geneontology.org/cgi-bin/amigo/go.cgi?view=details&query=GO:0060430)  [epithelial tube branching involved in lung morphogenesis](http://amigo.geneontology.org/cgi-bin/amigo/go.cgi?view=details&query=GO:0060441)  [Clara cell differentiation](http://amigo.geneontology.org/cgi-bin/amigo/go.cgi?view=details&query=GO:0060486)  [Type II pneumocyte differentiation](http://amigo.geneontology.org/cgi-bin/amigo/go.cgi?view=details&query=GO:0060510) |  |
| [211341_at](https://www.affymetrix.com/LinkServlet?&probeset=211341_at) | POU4F1 | POU class 4 homeobox 1 | 13 | -79173231 | [L20433](http://www.ncbi.nlm.nih.gov/entrez/query.fcgi?cmd=search&db=nucleotide&term=L20433%5BACCN%5D&doptcmdl=GenBank) | [5457](http://www.ncbi.nlm.nih.gov/sites/entrez?Db=gene&Cmd=DetailsSearch&Term=5457) | [13q31.1](http://www.ncbi.nlm.nih.gov/mapview/map_search.cgi?direct=on&idtype=gene&id=5457) | [Hs.654522](http://www.ncbi.nlm.nih.gov/UniGene/clust.cgi?ORG=Hs&CID=654522) | [23](http://www.ncbi.nih.gov/entrez/query.fcgi?tool=bioconductor&cmd=Retrieve&db=PubMed&list_uids=1357630%2C7623109%2C8234287%2C8248179%2C8889548%2C8941380%2C8995448%2C9448000%2C12427558%2C12432261%2C12441296%2C12477932%2C12810599%2C12893201%2C12911730%2C12934100%2C15021903%2C15272315%2C16247485%2C16276351%2C20348952%2C20376082%2C21116278) | [suckling behavior](http://amigo.geneontology.org/cgi-bin/amigo/go.cgi?view=details&query=GO:0001967)  [sequence-specific DNA binding transcription factor activity](http://amigo.geneontology.org/cgi-bin/amigo/go.cgi?view=details&query=GO:0003700)  [nucleus](http://amigo.geneontology.org/cgi-bin/amigo/go.cgi?view=details&query=GO:0005634)  [transcription, DNA-dependent](http://amigo.geneontology.org/cgi-bin/amigo/go.cgi?view=details&query=GO:0006351)  [regulation of transcription from RNA polymerase II promoter](http://amigo.geneontology.org/cgi-bin/amigo/go.cgi?view=details&query=GO:0006357)  [multicellular organismal development](http://amigo.geneontology.org/cgi-bin/amigo/go.cgi?view=details&query=GO:0007275)  [axonogenesis](http://amigo.geneontology.org/cgi-bin/amigo/go.cgi?view=details&query=GO:0007409)  [synapse assembly](http://amigo.geneontology.org/cgi-bin/amigo/go.cgi?view=details&query=GO:0007416)  [mesoderm development](http://amigo.geneontology.org/cgi-bin/amigo/go.cgi?view=details&query=GO:0007498)  [positive regulation of gene-specific transcription from RNA polymerase II promoter](http://amigo.geneontology.org/cgi-bin/amigo/go.cgi?view=details&query=GO:0010552)  [negative regulation of gene-specific transcription from RNA polymerase II promoter](http://amigo.geneontology.org/cgi-bin/amigo/go.cgi?view=details&query=GO:0010553)  [cell migration in hindbrain](http://amigo.geneontology.org/cgi-bin/amigo/go.cgi?view=details&query=GO:0021535)  [central nervous system neuron differentiation](http://amigo.geneontology.org/cgi-bin/amigo/go.cgi?view=details&query=GO:0021953)  [transcription regulator activity](http://amigo.geneontology.org/cgi-bin/amigo/go.cgi?view=details&query=GO:0030528)  [positive regulation of apoptosis](http://amigo.geneontology.org/cgi-bin/amigo/go.cgi?view=details&query=GO:0043065)  [sequence-specific DNA binding](http://amigo.geneontology.org/cgi-bin/amigo/go.cgi?view=details&query=GO:0043565)  [peripheral nervous system neuron differentiation](http://amigo.geneontology.org/cgi-bin/amigo/go.cgi?view=details&query=GO:0048934)  [regulation of neurogenesis](http://amigo.geneontology.org/cgi-bin/amigo/go.cgi?view=details&query=GO:0050767)  [proprioception involved in equilibrioception](http://amigo.geneontology.org/cgi-bin/amigo/go.cgi?view=details&query=GO:0051355) |  |
| [211673_s_at](https://www.affymetrix.com/LinkServlet?&probeset=211673_s_at) | MOCS1 | molybdenum cofactor synthesis 1 | 6 | -39872034, -39872034 | [AF034374](http://www.ncbi.nlm.nih.gov/entrez/query.fcgi?cmd=search&db=nucleotide&term=AF034374%5BACCN%5D&doptcmdl=GenBank) | [4337](http://www.ncbi.nlm.nih.gov/sites/entrez?Db=gene&Cmd=DetailsSearch&Term=4337) | [6p21.3](http://www.ncbi.nlm.nih.gov/mapview/map_search.cgi?direct=on&idtype=gene&id=4337) | [Hs.718492](http://www.ncbi.nlm.nih.gov/UniGene/clust.cgi?ORG=Hs&CID=718492) | [22](http://www.ncbi.nih.gov/entrez/query.fcgi?tool=bioconductor&cmd=Retrieve&db=PubMed&list_uids=8889548%2C9634514%2C9731530%2C9812897%2C9921896%2C10053004%2C10327149%2C10917590%2C11891227%2C12208140%2C12477932%2C12754701%2C14574404%2C14702039%2C15146197%2C15180982%2C15489334%2C16021469%2C16429380%2C17065069%2C19544009%2C19793632) | [nucleotide binding](http://amigo.geneontology.org/cgi-bin/amigo/go.cgi?view=details&query=GO:0000166)  [catalytic activity](http://amigo.geneontology.org/cgi-bin/amigo/go.cgi?view=details&query=GO:0003824)  [GTP binding](http://amigo.geneontology.org/cgi-bin/amigo/go.cgi?view=details&query=GO:0005525)  [nucleus](http://amigo.geneontology.org/cgi-bin/amigo/go.cgi?view=details&query=GO:0005634)  [cytosol](http://amigo.geneontology.org/cgi-bin/amigo/go.cgi?view=details&query=GO:0005829)  [vitamin metabolic process](http://amigo.geneontology.org/cgi-bin/amigo/go.cgi?view=details&query=GO:0006766)  [water-soluble vitamin metabolic process](http://amigo.geneontology.org/cgi-bin/amigo/go.cgi?view=details&query=GO:0006767)  [Mo-molybdopterin cofactor biosynthetic process](http://amigo.geneontology.org/cgi-bin/amigo/go.cgi?view=details&query=GO:0006777)  [Mo-molybdopterin cofactor biosynthetic process](http://amigo.geneontology.org/cgi-bin/amigo/go.cgi?view=details&query=GO:0006777)  [Mo-molybdopterin cofactor biosynthetic process](http://amigo.geneontology.org/cgi-bin/amigo/go.cgi?view=details&query=GO:0006777)  [molybdopterin synthase complex](http://amigo.geneontology.org/cgi-bin/amigo/go.cgi?view=details&query=GO:0019008)  [molybdopterin cofactor biosynthetic process](http://amigo.geneontology.org/cgi-bin/amigo/go.cgi?view=details&query=GO:0032324)  [metal ion binding](http://amigo.geneontology.org/cgi-bin/amigo/go.cgi?view=details&query=GO:0046872)  [4 iron, 4 sulfur cluster binding](http://amigo.geneontology.org/cgi-bin/amigo/go.cgi?view=details&query=GO:0051539)  [4 iron, 4 sulfur cluster binding](http://amigo.geneontology.org/cgi-bin/amigo/go.cgi?view=details&query=GO:0051539) | [Sulfur relay system](http://www.genome.ad.jp/kegg/pathway/hsa/hsa04122.html) |
| [212092_at](https://www.affymetrix.com/LinkServlet?&probeset=212092_at) | PEG10 | paternally expressed 10 | 7 | 94285636, 94285681 | [BE858180](http://www.ncbi.nlm.nih.gov/entrez/query.fcgi?cmd=search&db=nucleotide&term=BE858180%5BACCN%5D&doptcmdl=GenBank) | [23089](http://www.ncbi.nlm.nih.gov/sites/entrez?Db=gene&Cmd=DetailsSearch&Term=23089) | [7q21](http://www.ncbi.nlm.nih.gov/mapview/map_search.cgi?direct=on&idtype=gene&id=23089) | [Hs.147492](http://www.ncbi.nlm.nih.gov/UniGene/clust.cgi?ORG=Hs&CID=147492) | [28](http://www.ncbi.nih.gov/entrez/query.fcgi?tool=bioconductor&cmd=Retrieve&db=PubMed&list_uids=8619474%2C9110174%2C10470851%2C11158386%2C11318613%2C11574691%2C12477932%2C12620933%2C12810624%2C12853948%2C14576465%2C15342556%2C15611116%2C15716091%2C15767280%2C16053381%2C16093683%2C16225771%2C16344560%2C16423995%2C17369855%2C17621626%2C17942406%2C18550496%2C18625225%2C20084274%2C20362226%2C20460050) | [DNA binding](http://amigo.geneontology.org/cgi-bin/amigo/go.cgi?view=details&query=GO:0003677)  [protein binding](http://amigo.geneontology.org/cgi-bin/amigo/go.cgi?view=details&query=GO:0005515)  [nucleus](http://amigo.geneontology.org/cgi-bin/amigo/go.cgi?view=details&query=GO:0005634)  [cytoplasm](http://amigo.geneontology.org/cgi-bin/amigo/go.cgi?view=details&query=GO:0005737)  [apoptosis](http://amigo.geneontology.org/cgi-bin/amigo/go.cgi?view=details&query=GO:0006915)  [zinc ion binding](http://amigo.geneontology.org/cgi-bin/amigo/go.cgi?view=details&query=GO:0008270)  [cell differentiation](http://amigo.geneontology.org/cgi-bin/amigo/go.cgi?view=details&query=GO:0030154)  [negative regulation of transforming growth factor beta receptor signaling pathway](http://amigo.geneontology.org/cgi-bin/amigo/go.cgi?view=details&query=GO:0030512)  [metal ion binding](http://amigo.geneontology.org/cgi-bin/amigo/go.cgi?view=details&query=GO:0046872) |  |
| [212093_s_at](https://www.affymetrix.com/LinkServlet?&probeset=212093_s_at) | MTUS1 | microtubule associated tumor suppressor 1 | 8 | -17501303, -17501303, -17501303, -17501303 | [AI695017](http://www.ncbi.nlm.nih.gov/entrez/query.fcgi?cmd=search&db=nucleotide&term=AI695017%5BACCN%5D&doptcmdl=GenBank) | [57509](http://www.ncbi.nlm.nih.gov/sites/entrez?Db=gene&Cmd=DetailsSearch&Term=57509) | [8p22](http://www.ncbi.nlm.nih.gov/mapview/map_search.cgi?direct=on&idtype=gene&id=57509) | [Hs.7946](http://www.ncbi.nlm.nih.gov/UniGene/clust.cgi?ORG=Hs&CID=7946) | [16](http://www.ncbi.nih.gov/entrez/query.fcgi?tool=bioconductor&cmd=Retrieve&db=PubMed&list_uids=10574462%2C12477932%2C12692079%2C14702039%2C15123706%2C16344560%2C16650523%2C16887298%2C17301065%2C18519826%2C19344625%2C19545354%2C19794912%2C19956880%2C20379614%2C20687230) | [nucleus](http://amigo.geneontology.org/cgi-bin/amigo/go.cgi?view=details&query=GO:0005634)  [cytoplasm](http://amigo.geneontology.org/cgi-bin/amigo/go.cgi?view=details&query=GO:0005737)  [mitochondrion](http://amigo.geneontology.org/cgi-bin/amigo/go.cgi?view=details&query=GO:0005739)  [Golgi apparatus](http://amigo.geneontology.org/cgi-bin/amigo/go.cgi?view=details&query=GO:0005794)  [microtubule organizing center](http://amigo.geneontology.org/cgi-bin/amigo/go.cgi?view=details&query=GO:0005815)  [spindle](http://amigo.geneontology.org/cgi-bin/amigo/go.cgi?view=details&query=GO:0005819)  [cytoskeleton](http://amigo.geneontology.org/cgi-bin/amigo/go.cgi?view=details&query=GO:0005856)  [microtubule](http://amigo.geneontology.org/cgi-bin/amigo/go.cgi?view=details&query=GO:0005874)  [plasma membrane](http://amigo.geneontology.org/cgi-bin/amigo/go.cgi?view=details&query=GO:0005886) |  |
| [212096_s_at](https://www.affymetrix.com/LinkServlet?&probeset=212096_s_at) | MTUS1 | microtubule associated tumor suppressor 1 | 8 | -17501303, -17501303, -17501303, -17501303 | [AL096842](http://www.ncbi.nlm.nih.gov/entrez/query.fcgi?cmd=search&db=nucleotide&term=AL096842%5BACCN%5D&doptcmdl=GenBank) | [57509](http://www.ncbi.nlm.nih.gov/sites/entrez?Db=gene&Cmd=DetailsSearch&Term=57509) | [8p22](http://www.ncbi.nlm.nih.gov/mapview/map_search.cgi?direct=on&idtype=gene&id=57509) | [Hs.7946](http://www.ncbi.nlm.nih.gov/UniGene/clust.cgi?ORG=Hs&CID=7946) | [16](http://www.ncbi.nih.gov/entrez/query.fcgi?tool=bioconductor&cmd=Retrieve&db=PubMed&list_uids=10574462%2C12477932%2C12692079%2C14702039%2C15123706%2C16344560%2C16650523%2C16887298%2C17301065%2C18519826%2C19344625%2C19545354%2C19794912%2C19956880%2C20379614%2C20687230) | [nucleus](http://amigo.geneontology.org/cgi-bin/amigo/go.cgi?view=details&query=GO:0005634)  [cytoplasm](http://amigo.geneontology.org/cgi-bin/amigo/go.cgi?view=details&query=GO:0005737)  [mitochondrion](http://amigo.geneontology.org/cgi-bin/amigo/go.cgi?view=details&query=GO:0005739)  [Golgi apparatus](http://amigo.geneontology.org/cgi-bin/amigo/go.cgi?view=details&query=GO:0005794)  [microtubule organizing center](http://amigo.geneontology.org/cgi-bin/amigo/go.cgi?view=details&query=GO:0005815)  [spindle](http://amigo.geneontology.org/cgi-bin/amigo/go.cgi?view=details&query=GO:0005819)  [cytoskeleton](http://amigo.geneontology.org/cgi-bin/amigo/go.cgi?view=details&query=GO:0005856)  [microtubule](http://amigo.geneontology.org/cgi-bin/amigo/go.cgi?view=details&query=GO:0005874)  [plasma membrane](http://amigo.geneontology.org/cgi-bin/amigo/go.cgi?view=details&query=GO:0005886) |  |
| [212311_at](https://www.affymetrix.com/LinkServlet?&probeset=212311_at) | SEL1L3 | sel-1 suppressor of lin-12-like 3 (C. elegans) | 4 | -25749049 | [AA522514](http://www.ncbi.nlm.nih.gov/entrez/query.fcgi?cmd=search&db=nucleotide&term=AA522514%5BACCN%5D&doptcmdl=GenBank) | [23231](http://www.ncbi.nlm.nih.gov/sites/entrez?Db=gene&Cmd=DetailsSearch&Term=23231) | [4p15.2](http://www.ncbi.nlm.nih.gov/mapview/map_search.cgi?direct=on&idtype=gene&id=23231) | [Hs.479384](http://www.ncbi.nlm.nih.gov/UniGene/clust.cgi?ORG=Hs&CID=479384) | [7](http://www.ncbi.nih.gov/entrez/query.fcgi?tool=bioconductor&cmd=Retrieve&db=PubMed&list_uids=9847074%2C9872452%2C12477932%2C16169070%2C17457313%2C20379614%2C20708005) | [binding](http://amigo.geneontology.org/cgi-bin/amigo/go.cgi?view=details&query=GO:0005488)  [membrane](http://amigo.geneontology.org/cgi-bin/amigo/go.cgi?view=details&query=GO:0016020)  [integral to membrane](http://amigo.geneontology.org/cgi-bin/amigo/go.cgi?view=details&query=GO:0016021) |  |
| [212314_at](https://www.affymetrix.com/LinkServlet?&probeset=212314_at) | SEL1L3 | sel-1 suppressor of lin-12-like 3 (C. elegans) | 4 | -25749049 | [AB018289](http://www.ncbi.nlm.nih.gov/entrez/query.fcgi?cmd=search&db=nucleotide&term=AB018289%5BACCN%5D&doptcmdl=GenBank) | [23231](http://www.ncbi.nlm.nih.gov/sites/entrez?Db=gene&Cmd=DetailsSearch&Term=23231) | [4p15.2](http://www.ncbi.nlm.nih.gov/mapview/map_search.cgi?direct=on&idtype=gene&id=23231) | [Hs.479384](http://www.ncbi.nlm.nih.gov/UniGene/clust.cgi?ORG=Hs&CID=479384) | [7](http://www.ncbi.nih.gov/entrez/query.fcgi?tool=bioconductor&cmd=Retrieve&db=PubMed&list_uids=9847074%2C9872452%2C12477932%2C16169070%2C17457313%2C20379614%2C20708005) | [binding](http://amigo.geneontology.org/cgi-bin/amigo/go.cgi?view=details&query=GO:0005488)  [membrane](http://amigo.geneontology.org/cgi-bin/amigo/go.cgi?view=details&query=GO:0016020)  [integral to membrane](http://amigo.geneontology.org/cgi-bin/amigo/go.cgi?view=details&query=GO:0016021) |  |
| [212358_at](https://www.affymetrix.com/LinkServlet?&probeset=212358_at) | CLIP3 | CAP-GLY domain containing linker protein 3 | 19 | -36505562 | [AL117468](http://www.ncbi.nlm.nih.gov/entrez/query.fcgi?cmd=search&db=nucleotide&term=AL117468%5BACCN%5D&doptcmdl=GenBank) | [25999](http://www.ncbi.nlm.nih.gov/sites/entrez?Db=gene&Cmd=DetailsSearch&Term=25999) | [19q13.12](http://www.ncbi.nlm.nih.gov/mapview/map_search.cgi?direct=on&idtype=gene&id=25999) | [Hs.466539](http://www.ncbi.nlm.nih.gov/UniGene/clust.cgi?ORG=Hs&CID=466539) | [8](http://www.ncbi.nih.gov/entrez/query.fcgi?tool=bioconductor&cmd=Retrieve&db=PubMed&list_uids=11854307%2C12477932%2C14702039%2C15262990%2C15489334%2C16344560%2C19139280%2C20052288) | [positive regulation of protein phosphorylation](http://amigo.geneontology.org/cgi-bin/amigo/go.cgi?view=details&query=GO:0001934)  [cytoplasm](http://amigo.geneontology.org/cgi-bin/amigo/go.cgi?view=details&query=GO:0005737)  [microsome](http://amigo.geneontology.org/cgi-bin/amigo/go.cgi?view=details&query=GO:0005792)  [Golgi apparatus](http://amigo.geneontology.org/cgi-bin/amigo/go.cgi?view=details&query=GO:0005794)  [Golgi stack](http://amigo.geneontology.org/cgi-bin/amigo/go.cgi?view=details&query=GO:0005795)  [plasma membrane](http://amigo.geneontology.org/cgi-bin/amigo/go.cgi?view=details&query=GO:0005886)  [plasma membrane](http://amigo.geneontology.org/cgi-bin/amigo/go.cgi?view=details&query=GO:0005886)  [microtubule binding](http://amigo.geneontology.org/cgi-bin/amigo/go.cgi?view=details&query=GO:0008017)  [microtubule binding](http://amigo.geneontology.org/cgi-bin/amigo/go.cgi?view=details&query=GO:0008017)  [positive regulation of glucose transport](http://amigo.geneontology.org/cgi-bin/amigo/go.cgi?view=details&query=GO:0010828)  [peptidyl-L-cysteine S-palmitoylation](http://amigo.geneontology.org/cgi-bin/amigo/go.cgi?view=details&query=GO:0018230)  [negative regulation of microtubule polymerization](http://amigo.geneontology.org/cgi-bin/amigo/go.cgi?view=details&query=GO:0031115)  [early endosome membrane](http://amigo.geneontology.org/cgi-bin/amigo/go.cgi?view=details&query=GO:0031901)  [trans-Golgi network membrane](http://amigo.geneontology.org/cgi-bin/amigo/go.cgi?view=details&query=GO:0032588)  [trans-Golgi network membrane](http://amigo.geneontology.org/cgi-bin/amigo/go.cgi?view=details&query=GO:0032588)  [ganglioside binding](http://amigo.geneontology.org/cgi-bin/amigo/go.cgi?view=details&query=GO:0035594)  [positive regulation of apoptosis](http://amigo.geneontology.org/cgi-bin/amigo/go.cgi?view=details&query=GO:0043065)  [membrane biogenesis](http://amigo.geneontology.org/cgi-bin/amigo/go.cgi?view=details&query=GO:0044091)  [membrane raft](http://amigo.geneontology.org/cgi-bin/amigo/go.cgi?view=details&query=GO:0045121)  [fat cell differentiation](http://amigo.geneontology.org/cgi-bin/amigo/go.cgi?view=details&query=GO:0045444)  [positive regulation of endocytosis](http://amigo.geneontology.org/cgi-bin/amigo/go.cgi?view=details&query=GO:0045807)  [recycling endosome membrane](http://amigo.geneontology.org/cgi-bin/amigo/go.cgi?view=details&query=GO:0055038)  [chaperone-mediated protein transport](http://amigo.geneontology.org/cgi-bin/amigo/go.cgi?view=details&query=GO:0072321)  [positive regulation of establishment of protein localization in plasma membrane](http://amigo.geneontology.org/cgi-bin/amigo/go.cgi?view=details&query=GO:0090004) |  |
| [212526_at](https://www.affymetrix.com/LinkServlet?&probeset=212526_at) | SPG20 | spastic paraplegia 20 (Troyer syndrome) | 13 | -36875776, -36875776, -36875776 | [AK002207](http://www.ncbi.nlm.nih.gov/entrez/query.fcgi?cmd=search&db=nucleotide&term=AK002207%5BACCN%5D&doptcmdl=GenBank) | [23111](http://www.ncbi.nlm.nih.gov/sites/entrez?Db=gene&Cmd=DetailsSearch&Term=23111) | [13q13.3](http://www.ncbi.nlm.nih.gov/mapview/map_search.cgi?direct=on&idtype=gene&id=23111) | [Hs.440414](http://www.ncbi.nlm.nih.gov/UniGene/clust.cgi?ORG=Hs&CID=440414) | [26](http://www.ncbi.nih.gov/entrez/query.fcgi?tool=bioconductor&cmd=Retrieve&db=PubMed&list_uids=6022528%2C8889549%2C9628581%2C10463356%2C12134148%2C12477932%2C12676568%2C14702039%2C15057823%2C15188498%2C15231748%2C15489334%2C16036216%2C16055720%2C16344560%2C16945107%2C17332501%2C17420921%2C18413476%2C19307600%2C19580544%2C19620182%2C19765186%2C20301556%2C20504295%2C20719964) | [cytoplasm](http://amigo.geneontology.org/cgi-bin/amigo/go.cgi?view=details&query=GO:0005737)  [cell death](http://amigo.geneontology.org/cgi-bin/amigo/go.cgi?view=details&query=GO:0008219)  [ubiquitin protein ligase binding](http://amigo.geneontology.org/cgi-bin/amigo/go.cgi?view=details&query=GO:0031625) |  |
| [212538_at](https://www.affymetrix.com/LinkServlet?&probeset=212538_at) | DOCK9 | dedicator of cytokinesis 9 | 13 | -99512434, -99512434, -99445740, -99445740 | [AL576253](http://www.ncbi.nlm.nih.gov/entrez/query.fcgi?cmd=search&db=nucleotide&term=AL576253%5BACCN%5D&doptcmdl=GenBank) | [23348](http://www.ncbi.nlm.nih.gov/sites/entrez?Db=gene&Cmd=DetailsSearch&Term=23348) | [13q32.3](http://www.ncbi.nlm.nih.gov/mapview/map_search.cgi?direct=on&idtype=gene&id=23348) | [Hs.596105](http://www.ncbi.nlm.nih.gov/UniGene/clust.cgi?ORG=Hs&CID=596105) | [18](http://www.ncbi.nih.gov/entrez/query.fcgi?tool=bioconductor&cmd=Retrieve&db=PubMed&list_uids=8889548%2C10470851%2C11991713%2C12168954%2C12172552%2C12432077%2C12477932%2C14702039%2C15057823%2C15489334%2C16344560%2C17728666%2C17935486%2C18056264%2C18729074%2C19745154%2C19809089%2C20379614) | [guanyl-nucleotide exchange factor activity](http://amigo.geneontology.org/cgi-bin/amigo/go.cgi?view=details&query=GO:0005085)  [protein binding](http://amigo.geneontology.org/cgi-bin/amigo/go.cgi?view=details&query=GO:0005515)  [GTP binding](http://amigo.geneontology.org/cgi-bin/amigo/go.cgi?view=details&query=GO:0005525)  [cellular_component](http://amigo.geneontology.org/cgi-bin/amigo/go.cgi?view=details&query=GO:0005575)  [cytosol](http://amigo.geneontology.org/cgi-bin/amigo/go.cgi?view=details&query=GO:0005829)  [blood coagulation](http://amigo.geneontology.org/cgi-bin/amigo/go.cgi?view=details&query=GO:0007596)  [biological_process](http://amigo.geneontology.org/cgi-bin/amigo/go.cgi?view=details&query=GO:0008150)  [endomembrane system](http://amigo.geneontology.org/cgi-bin/amigo/go.cgi?view=details&query=GO:0012505)  [membrane](http://amigo.geneontology.org/cgi-bin/amigo/go.cgi?view=details&query=GO:0016020)  [GTPase binding](http://amigo.geneontology.org/cgi-bin/amigo/go.cgi?view=details&query=GO:0051020) |  |
| [212606_at](https://www.affymetrix.com/LinkServlet?&probeset=212606_at) | WDFY3 | WD repeat and FYVE domain containing 3 | 4 | -85590696 | [AL536319](http://www.ncbi.nlm.nih.gov/entrez/query.fcgi?cmd=search&db=nucleotide&term=AL536319%5BACCN%5D&doptcmdl=GenBank) | [23001](http://www.ncbi.nlm.nih.gov/sites/entrez?Db=gene&Cmd=DetailsSearch&Term=23001) | [4q21.23](http://www.ncbi.nlm.nih.gov/mapview/map_search.cgi?direct=on&idtype=gene&id=23001) | [Hs.480116](http://www.ncbi.nlm.nih.gov/UniGene/clust.cgi?ORG=Hs&CID=480116) [Hs.726339](http://www.ncbi.nlm.nih.gov/UniGene/clust.cgi?ORG=Hs&CID=726339) | [16](http://www.ncbi.nih.gov/entrez/query.fcgi?tool=bioconductor&cmd=Retrieve&db=PubMed&list_uids=8619474%2C8889548%2C9110174%2C9847074%2C10231032%2C12168954%2C12477932%2C14702039%2C15292400%2C15489334%2C17207965%2C17452356%2C20168092%2C20379614%2C20417604%2C20493804) | [protein binding](http://amigo.geneontology.org/cgi-bin/amigo/go.cgi?view=details&query=GO:0005515)  [1-phosphatidylinositol binding](http://amigo.geneontology.org/cgi-bin/amigo/go.cgi?view=details&query=GO:0005545)  [nuclear envelope](http://amigo.geneontology.org/cgi-bin/amigo/go.cgi?view=details&query=GO:0005635)  [autophagic vacuole](http://amigo.geneontology.org/cgi-bin/amigo/go.cgi?view=details&query=GO:0005776)  [zinc ion binding](http://amigo.geneontology.org/cgi-bin/amigo/go.cgi?view=details&query=GO:0008270)  [membrane](http://amigo.geneontology.org/cgi-bin/amigo/go.cgi?view=details&query=GO:0016020)  [extrinsic to membrane](http://amigo.geneontology.org/cgi-bin/amigo/go.cgi?view=details&query=GO:0019898)  [cytoplasmic part](http://amigo.geneontology.org/cgi-bin/amigo/go.cgi?view=details&query=GO:0044444)  [metal ion binding](http://amigo.geneontology.org/cgi-bin/amigo/go.cgi?view=details&query=GO:0046872) |  |
| [212651_at](https://www.affymetrix.com/LinkServlet?&probeset=212651_at) | RHOBTB1 | Rho-related BTB domain containing 1 | 10 | -62629199, -62629199, -62629199 | [AB018283](http://www.ncbi.nlm.nih.gov/entrez/query.fcgi?cmd=search&db=nucleotide&term=AB018283%5BACCN%5D&doptcmdl=GenBank) | [9886](http://www.ncbi.nlm.nih.gov/sites/entrez?Db=gene&Cmd=DetailsSearch&Term=9886) | [10q21.2](http://www.ncbi.nlm.nih.gov/mapview/map_search.cgi?direct=on&idtype=gene&id=9886) | [Hs.148670](http://www.ncbi.nlm.nih.gov/UniGene/clust.cgi?ORG=Hs&CID=148670) | [12](http://www.ncbi.nih.gov/entrez/query.fcgi?tool=bioconductor&cmd=Retrieve&db=PubMed&list_uids=9872452%2C11222756%2C12426103%2C12477932%2C14521508%2C14702039%2C15146197%2C15489334%2C16170569%2C16344560%2C16385451%2C19430483) | [nucleotide binding](http://amigo.geneontology.org/cgi-bin/amigo/go.cgi?view=details&query=GO:0000166)  [GTP binding](http://amigo.geneontology.org/cgi-bin/amigo/go.cgi?view=details&query=GO:0005525)  [intracellular](http://amigo.geneontology.org/cgi-bin/amigo/go.cgi?view=details&query=GO:0005622)  [cytosol](http://amigo.geneontology.org/cgi-bin/amigo/go.cgi?view=details&query=GO:0005829)  [plasma membrane](http://amigo.geneontology.org/cgi-bin/amigo/go.cgi?view=details&query=GO:0005886)  [small GTPase mediated signal transduction](http://amigo.geneontology.org/cgi-bin/amigo/go.cgi?view=details&query=GO:0007264)  [regulation of small GTPase mediated signal transduction](http://amigo.geneontology.org/cgi-bin/amigo/go.cgi?view=details&query=GO:0051056) | [Ubiquitin mediated proteolysis](http://www.genome.ad.jp/kegg/pathway/hsa/hsa04120.html) |
| [212736_at](https://www.affymetrix.com/LinkServlet?&probeset=212736_at) | C16orf45 | chromosome 16 open reading frame 45 | 16 | 15528324, 15596122 | [BE299456](http://www.ncbi.nlm.nih.gov/entrez/query.fcgi?cmd=search&db=nucleotide&term=BE299456%5BACCN%5D&doptcmdl=GenBank) | [89927](http://www.ncbi.nlm.nih.gov/sites/entrez?Db=gene&Cmd=DetailsSearch&Term=89927) | [16p13.11](http://www.ncbi.nlm.nih.gov/mapview/map_search.cgi?direct=on&idtype=gene&id=89927) | [Hs.401798](http://www.ncbi.nlm.nih.gov/UniGene/clust.cgi?ORG=Hs&CID=401798) | [6](http://www.ncbi.nih.gov/entrez/query.fcgi?tool=bioconductor&cmd=Retrieve&db=PubMed&list_uids=10493829%2C12477932%2C14702039%2C15489334%2C16169070%2C17207965) |  |  |
| [212816_s_at](https://www.affymetrix.com/LinkServlet?&probeset=212816_s_at) | CBS | cystathionine-beta-synthase | 21 | -44473300, -44473300 | [BE613178](http://www.ncbi.nlm.nih.gov/entrez/query.fcgi?cmd=search&db=nucleotide&term=BE613178%5BACCN%5D&doptcmdl=GenBank) | [875](http://www.ncbi.nlm.nih.gov/sites/entrez?Db=gene&Cmd=DetailsSearch&Term=875) | [21q22.3](http://www.ncbi.nlm.nih.gov/mapview/map_search.cgi?direct=on&idtype=gene&id=875) | [Hs.533013](http://www.ncbi.nlm.nih.gov/UniGene/clust.cgi?ORG=Hs&CID=533013) | [239](http://www.ncbi.nih.gov/entrez/query.fcgi?tool=bioconductor&cmd=Retrieve&db=PubMed&list_uids=404147%2C681363%2C840498%2C1301198%2C2894761%2C7506602%2C7564249%2C7598711%2C7611293%2C7635485%2C7762555%2C7849717%2C7903580%2C7929220%2C7967489%2C7981678%2C8022826%2C8353501%2C8528202%2C8755636%2C8803779%2C8990018%2C9156316%2C9266356%2C9361025%2C9383285%2C9466992%2C9590298%2C9790750%2C9889017%2C10215408%2C10338090%2C10408774%2C10434301%2C10462600%2C10791559%2C10830953%2C11013450%2C11074524%2C11149614%2C11173483%2C11204591%2C11292330%2C11310576%2C11341749%2C11359213%2C11359462%2C11434706%2C11457468%2C11483494%2C11528503%2C11575217%2C11672761%2C11748855%2C11758232%2C11872884%2C12007221%2C12015064%2C12020105%2C12082592%2C12124992%2C12154064%2C12173932%2C12180146%2C12186157%2C12228232%2C12269827%2C12379655%2C12393509%2C12413583%2C12427542%2C12439143%2C12477932%2C12529702%2C12642343%2C12649066%2C12725044%2C12815602%2C12855221%2C12889841%2C14670973%2C14977639%2C15009965%2C15082224%2C15228193%2C15354395%2C15365998%2C15489334%2C15503105%2C15520012%2C15544339%2C15554031%2C15719048%2C15748616%2C15755387%2C15772012%2C15889417%2C15922487%2C15972722%2C15975077%2C16007597%2C16013960%2C16115349%2C16189514%2C16205833%2C16259797%2C16274669%2C16328059%2C16363792%2C16375773%2C16422253%2C16470595%2C16479318%2C16505479%2C16541333%2C16601865%2C16709328%2C16780588%2C16791140%2C16792904%2C16941496%2C16953589%2C17035141%2C17087506%2C17119116%2C17160942%2C17311259%2C17311260%2C17319270%2C17327360%2C17352495%2C17436311%2C17540596%2C17548676%2C17553479%2C17601350%2C17621169%2C17686644%2C17726616%2C17891500%2C17956124%2C17993766%2C18029348%2C18060852%2C18203168%2C18278872%2C18398434%2C18427977%2C18447718%2C18454451%2C18614746%2C18620331%2C18622257%2C18635682%2C18636124%2C18676680%2C18708589%2C18776696%2C18785313%2C18792976%2C18799873%2C18818748%2C18830263%2C18839533%2C18936436%2C18950795%2C18977241%2C18977990%2C18988749%2C18992148%2C19010420%2C19019082%2C19019335%2C19019492%2C19048631%2C19064578%2C19074437%2C19112534%2C19161160%2C19166826%2C19170196%2C19238444%2C19267073%2C19370759%2C19424622%2C19429038%2C19447967%2C19493349%2C19527514%2C19531479%2C19559392%2C19593657%2C19625176%2C19657138%2C19657388%2C19683694%2C19692168%2C19722721%2C19729796%2C19737740%2C19906435%2C19913121%2C19948975%2C20031554%2C20031578%2C20056620%2C20066033%2C20082058%2C20140262%2C20160465%2C20217437%2C20237949%2C20301697%2C20308073%2C20346360%2C20379614%2C20453000%2C20458436%2C20506325%2C20544798%2C20559280%2C20565774%2C20601281%2C20615890%2C20628086%2C20634891%2C20638879%2C20670920%2C20707729%2C20717043%2C20718043%2C20737570%2C20883119%2C20890573%2C20939734%2C20948192%2C21045269%2C21055808%2C21062078) | [endochondral ossification](http://amigo.geneontology.org/cgi-bin/amigo/go.cgi?view=details&query=GO:0001958)  [cystathionine beta-synthase activity](http://amigo.geneontology.org/cgi-bin/amigo/go.cgi?view=details&query=GO:0004122)  [protein binding](http://amigo.geneontology.org/cgi-bin/amigo/go.cgi?view=details&query=GO:0005515)  [soluble fraction](http://amigo.geneontology.org/cgi-bin/amigo/go.cgi?view=details&query=GO:0005625)  [nucleus](http://amigo.geneontology.org/cgi-bin/amigo/go.cgi?view=details&query=GO:0005634)  [nucleolus](http://amigo.geneontology.org/cgi-bin/amigo/go.cgi?view=details&query=GO:0005730)  [cytoplasm](http://amigo.geneontology.org/cgi-bin/amigo/go.cgi?view=details&query=GO:0005737)  [cytosol](http://amigo.geneontology.org/cgi-bin/amigo/go.cgi?view=details&query=GO:0005829)  [cysteine biosynthetic process from serine](http://amigo.geneontology.org/cgi-bin/amigo/go.cgi?view=details&query=GO:0006535)  [L-serine metabolic process](http://amigo.geneontology.org/cgi-bin/amigo/go.cgi?view=details&query=GO:0006563)  [L-serine catabolic process](http://amigo.geneontology.org/cgi-bin/amigo/go.cgi?view=details&query=GO:0006565)  [cellular amino acid biosynthetic process](http://amigo.geneontology.org/cgi-bin/amigo/go.cgi?view=details&query=GO:0008652)  [lyase activity](http://amigo.geneontology.org/cgi-bin/amigo/go.cgi?view=details&query=GO:0016829)  [cysteine biosynthetic process via cystathionine](http://amigo.geneontology.org/cgi-bin/amigo/go.cgi?view=details&query=GO:0019343)  [transsulfuration](http://amigo.geneontology.org/cgi-bin/amigo/go.cgi?view=details&query=GO:0019346)  [L-cysteine catabolic process](http://amigo.geneontology.org/cgi-bin/amigo/go.cgi?view=details&query=GO:0019448)  [enzyme binding](http://amigo.geneontology.org/cgi-bin/amigo/go.cgi?view=details&query=GO:0019899)  [heme binding](http://amigo.geneontology.org/cgi-bin/amigo/go.cgi?view=details&query=GO:0020037)  [heme binding](http://amigo.geneontology.org/cgi-bin/amigo/go.cgi?view=details&query=GO:0020037)  [pyridoxal phosphate binding](http://amigo.geneontology.org/cgi-bin/amigo/go.cgi?view=details&query=GO:0030170)  [ubiquitin protein ligase binding](http://amigo.geneontology.org/cgi-bin/amigo/go.cgi?view=details&query=GO:0031625)  [response to nutrient levels](http://amigo.geneontology.org/cgi-bin/amigo/go.cgi?view=details&query=GO:0031667)  [identical protein binding](http://amigo.geneontology.org/cgi-bin/amigo/go.cgi?view=details&query=GO:0042802)  [protein homodimerization activity](http://amigo.geneontology.org/cgi-bin/amigo/go.cgi?view=details&query=GO:0042803)  [intracellular membrane-bounded organelle](http://amigo.geneontology.org/cgi-bin/amigo/go.cgi?view=details&query=GO:0043231)  [homocysteine catabolic process](http://amigo.geneontology.org/cgi-bin/amigo/go.cgi?view=details&query=GO:0043418)  [regulation of JUN kinase activity](http://amigo.geneontology.org/cgi-bin/amigo/go.cgi?view=details&query=GO:0043506)  [metal ion binding](http://amigo.geneontology.org/cgi-bin/amigo/go.cgi?view=details&query=GO:0046872)  [homocysteine metabolic process](http://amigo.geneontology.org/cgi-bin/amigo/go.cgi?view=details&query=GO:0050667)  [response to folic acid](http://amigo.geneontology.org/cgi-bin/amigo/go.cgi?view=details&query=GO:0051593)  [hydrogen sulfide biosynthetic process](http://amigo.geneontology.org/cgi-bin/amigo/go.cgi?view=details&query=GO:0070814) | [Glycine, serine and threonine metabolism](http://www.genome.ad.jp/dbget-bin/show_pathway?MAP00260+4.2.1.22)  [Cysteine and methionine metabolism](http://www.genome.ad.jp/dbget-bin/show_pathway?MAP00270+4.2.1.22)  [Metabolic pathways](http://www.genome.ad.jp/dbget-bin/show_pathway?MAP01100+4.2.1.22) |
| [212970_at](https://www.affymetrix.com/LinkServlet?&probeset=212970_at) | APBB2 | amyloid beta (A4) precursor protein-binding, family B, member 2 | 4 | -40812044, -40812044, -40812044 | [AI694303](http://www.ncbi.nlm.nih.gov/entrez/query.fcgi?cmd=search&db=nucleotide&term=AI694303%5BACCN%5D&doptcmdl=GenBank) | [323](http://www.ncbi.nlm.nih.gov/sites/entrez?Db=gene&Cmd=DetailsSearch&Term=323) | [4p13](http://www.ncbi.nlm.nih.gov/mapview/map_search.cgi?direct=on&idtype=gene&id=323) | [Hs.479602](http://www.ncbi.nlm.nih.gov/UniGene/clust.cgi?ORG=Hs&CID=479602) | [15](http://www.ncbi.nih.gov/entrez/query.fcgi?tool=bioconductor&cmd=Retrieve&db=PubMed&list_uids=8855266%2C8955346%2C9585438%2C12089154%2C12477932%2C14527950%2C15489334%2C15714520%2C15761153%2C16083851%2C16344560%2C17567906%2C18852029%2C19542221%2C20677014) | [beta-amyloid binding](http://amigo.geneontology.org/cgi-bin/amigo/go.cgi?view=details&query=GO:0001540)  [protein binding](http://amigo.geneontology.org/cgi-bin/amigo/go.cgi?view=details&query=GO:0005515)  [nucleus](http://amigo.geneontology.org/cgi-bin/amigo/go.cgi?view=details&query=GO:0005634)  [cell cycle arrest](http://amigo.geneontology.org/cgi-bin/amigo/go.cgi?view=details&query=GO:0007050)  [transcription factor binding](http://amigo.geneontology.org/cgi-bin/amigo/go.cgi?view=details&query=GO:0008134)  [membrane](http://amigo.geneontology.org/cgi-bin/amigo/go.cgi?view=details&query=GO:0016020)  [lamellipodium](http://amigo.geneontology.org/cgi-bin/amigo/go.cgi?view=details&query=GO:0030027)  [negative regulation of cell growth](http://amigo.geneontology.org/cgi-bin/amigo/go.cgi?view=details&query=GO:0030308)  [growth cone](http://amigo.geneontology.org/cgi-bin/amigo/go.cgi?view=details&query=GO:0030426)  [intracellular signal transduction](http://amigo.geneontology.org/cgi-bin/amigo/go.cgi?view=details&query=GO:0035556)  [synapse](http://amigo.geneontology.org/cgi-bin/amigo/go.cgi?view=details&query=GO:0045202)  [regulation of transcription](http://amigo.geneontology.org/cgi-bin/amigo/go.cgi?view=details&query=GO:0045449)  [negative regulation of S phase of mitotic cell cycle](http://amigo.geneontology.org/cgi-bin/amigo/go.cgi?view=details&query=GO:0045749) |  |
| [212985_at](https://www.affymetrix.com/LinkServlet?&probeset=212985_at) | APBB2 | amyloid beta (A4) precursor protein-binding, family B, member 2 | 4 | -40812044, -40812044, -40812044 | [BF115739](http://www.ncbi.nlm.nih.gov/entrez/query.fcgi?cmd=search&db=nucleotide&term=BF115739%5BACCN%5D&doptcmdl=GenBank) | [323](http://www.ncbi.nlm.nih.gov/sites/entrez?Db=gene&Cmd=DetailsSearch&Term=323) | [4p13](http://www.ncbi.nlm.nih.gov/mapview/map_search.cgi?direct=on&idtype=gene&id=323) | [Hs.479602](http://www.ncbi.nlm.nih.gov/UniGene/clust.cgi?ORG=Hs&CID=479602) | [15](http://www.ncbi.nih.gov/entrez/query.fcgi?tool=bioconductor&cmd=Retrieve&db=PubMed&list_uids=8855266%2C8955346%2C9585438%2C12089154%2C12477932%2C14527950%2C15489334%2C15714520%2C15761153%2C16083851%2C16344560%2C17567906%2C18852029%2C19542221%2C20677014) | [beta-amyloid binding](http://amigo.geneontology.org/cgi-bin/amigo/go.cgi?view=details&query=GO:0001540)  [protein binding](http://amigo.geneontology.org/cgi-bin/amigo/go.cgi?view=details&query=GO:0005515)  [nucleus](http://amigo.geneontology.org/cgi-bin/amigo/go.cgi?view=details&query=GO:0005634)  [cell cycle arrest](http://amigo.geneontology.org/cgi-bin/amigo/go.cgi?view=details&query=GO:0007050)  [transcription factor binding](http://amigo.geneontology.org/cgi-bin/amigo/go.cgi?view=details&query=GO:0008134)  [membrane](http://amigo.geneontology.org/cgi-bin/amigo/go.cgi?view=details&query=GO:0016020)  [lamellipodium](http://amigo.geneontology.org/cgi-bin/amigo/go.cgi?view=details&query=GO:0030027)  [negative regulation of cell growth](http://amigo.geneontology.org/cgi-bin/amigo/go.cgi?view=details&query=GO:0030308)  [growth cone](http://amigo.geneontology.org/cgi-bin/amigo/go.cgi?view=details&query=GO:0030426)  [intracellular signal transduction](http://amigo.geneontology.org/cgi-bin/amigo/go.cgi?view=details&query=GO:0035556)  [synapse](http://amigo.geneontology.org/cgi-bin/amigo/go.cgi?view=details&query=GO:0045202)  [regulation of transcription](http://amigo.geneontology.org/cgi-bin/amigo/go.cgi?view=details&query=GO:0045449)  [negative regulation of S phase of mitotic cell cycle](http://amigo.geneontology.org/cgi-bin/amigo/go.cgi?view=details&query=GO:0045749) |  |
| [213181_s_at](https://www.affymetrix.com/LinkServlet?&probeset=213181_s_at) | MOCS1 | molybdenum cofactor synthesis 1 | 6 | -39872034, -39872034 | [AL583528](http://www.ncbi.nlm.nih.gov/entrez/query.fcgi?cmd=search&db=nucleotide&term=AL583528%5BACCN%5D&doptcmdl=GenBank) | [4337](http://www.ncbi.nlm.nih.gov/sites/entrez?Db=gene&Cmd=DetailsSearch&Term=4337) | [6p21.3](http://www.ncbi.nlm.nih.gov/mapview/map_search.cgi?direct=on&idtype=gene&id=4337) | [Hs.718492](http://www.ncbi.nlm.nih.gov/UniGene/clust.cgi?ORG=Hs&CID=718492) | [22](http://www.ncbi.nih.gov/entrez/query.fcgi?tool=bioconductor&cmd=Retrieve&db=PubMed&list_uids=8889548%2C9634514%2C9731530%2C9812897%2C9921896%2C10053004%2C10327149%2C10917590%2C11891227%2C12208140%2C12477932%2C12754701%2C14574404%2C14702039%2C15146197%2C15180982%2C15489334%2C16021469%2C16429380%2C17065069%2C19544009%2C19793632) | [nucleotide binding](http://amigo.geneontology.org/cgi-bin/amigo/go.cgi?view=details&query=GO:0000166)  [catalytic activity](http://amigo.geneontology.org/cgi-bin/amigo/go.cgi?view=details&query=GO:0003824)  [GTP binding](http://amigo.geneontology.org/cgi-bin/amigo/go.cgi?view=details&query=GO:0005525)  [nucleus](http://amigo.geneontology.org/cgi-bin/amigo/go.cgi?view=details&query=GO:0005634)  [cytosol](http://amigo.geneontology.org/cgi-bin/amigo/go.cgi?view=details&query=GO:0005829)  [vitamin metabolic process](http://amigo.geneontology.org/cgi-bin/amigo/go.cgi?view=details&query=GO:0006766)  [water-soluble vitamin metabolic process](http://amigo.geneontology.org/cgi-bin/amigo/go.cgi?view=details&query=GO:0006767)  [Mo-molybdopterin cofactor biosynthetic process](http://amigo.geneontology.org/cgi-bin/amigo/go.cgi?view=details&query=GO:0006777)  [Mo-molybdopterin cofactor biosynthetic process](http://amigo.geneontology.org/cgi-bin/amigo/go.cgi?view=details&query=GO:0006777)  [Mo-molybdopterin cofactor biosynthetic process](http://amigo.geneontology.org/cgi-bin/amigo/go.cgi?view=details&query=GO:0006777)  [molybdopterin synthase complex](http://amigo.geneontology.org/cgi-bin/amigo/go.cgi?view=details&query=GO:0019008)  [molybdopterin cofactor biosynthetic process](http://amigo.geneontology.org/cgi-bin/amigo/go.cgi?view=details&query=GO:0032324)  [metal ion binding](http://amigo.geneontology.org/cgi-bin/amigo/go.cgi?view=details&query=GO:0046872)  [4 iron, 4 sulfur cluster binding](http://amigo.geneontology.org/cgi-bin/amigo/go.cgi?view=details&query=GO:0051539)  [4 iron, 4 sulfur cluster binding](http://amigo.geneontology.org/cgi-bin/amigo/go.cgi?view=details&query=GO:0051539) | [Sulfur relay system](http://www.genome.ad.jp/kegg/pathway/hsa/hsa04122.html) |
| [213193_x_at](https://www.affymetrix.com/LinkServlet?&probeset=213193_x_at) | TRBC1 | T cell receptor beta constant 1 | 7 |  | [AL559122](http://www.ncbi.nlm.nih.gov/entrez/query.fcgi?cmd=search&db=nucleotide&term=AL559122%5BACCN%5D&doptcmdl=GenBank) | [28639](http://www.ncbi.nlm.nih.gov/sites/entrez?Db=gene&Cmd=DetailsSearch&Term=28639) | [7q34](http://www.ncbi.nlm.nih.gov/mapview/map_search.cgi?direct=on&idtype=gene&id=28639) | [Hs.382212](http://www.ncbi.nlm.nih.gov/UniGene/clust.cgi?ORG=Hs&CID=382212) | [10](http://www.ncbi.nih.gov/entrez/query.fcgi?tool=bioconductor&cmd=Retrieve&db=PubMed&list_uids=3860845%2C6336315%2C8650574%2C11048639%2C11827988%2C12429093%2C12477932%2C15480765%2C15489916%2C17652306) | [receptor activity](http://amigo.geneontology.org/cgi-bin/amigo/go.cgi?view=details&query=GO:0004872)  [protein binding](http://amigo.geneontology.org/cgi-bin/amigo/go.cgi?view=details&query=GO:0005515)  [plasma membrane](http://amigo.geneontology.org/cgi-bin/amigo/go.cgi?view=details&query=GO:0005886)  [plasma membrane](http://amigo.geneontology.org/cgi-bin/amigo/go.cgi?view=details&query=GO:0005886)  [immune response](http://amigo.geneontology.org/cgi-bin/amigo/go.cgi?view=details&query=GO:0006955)  [membrane](http://amigo.geneontology.org/cgi-bin/amigo/go.cgi?view=details&query=GO:0016020)  [integral to membrane](http://amigo.geneontology.org/cgi-bin/amigo/go.cgi?view=details&query=GO:0016021)  [T cell costimulation](http://amigo.geneontology.org/cgi-bin/amigo/go.cgi?view=details&query=GO:0031295)  [regulation of immune response](http://amigo.geneontology.org/cgi-bin/amigo/go.cgi?view=details&query=GO:0050776)  [T cell receptor signaling pathway](http://amigo.geneontology.org/cgi-bin/amigo/go.cgi?view=details&query=GO:0050852) |  |
| [213194_at](https://www.affymetrix.com/LinkServlet?&probeset=213194_at) | ROBO1 | roundabout, axon guidance receptor, homolog 1 (Drosophila) | 3 | -78646387, -78646387 | [BF059159](http://www.ncbi.nlm.nih.gov/entrez/query.fcgi?cmd=search&db=nucleotide&term=BF059159%5BACCN%5D&doptcmdl=GenBank) | [6091](http://www.ncbi.nlm.nih.gov/sites/entrez?Db=gene&Cmd=DetailsSearch&Term=6091) | [3p12](http://www.ncbi.nlm.nih.gov/mapview/map_search.cgi?direct=on&idtype=gene&id=6091) | [Hs.13640](http://www.ncbi.nlm.nih.gov/UniGene/clust.cgi?ORG=Hs&CID=13640) | [51](http://www.ncbi.nih.gov/entrez/query.fcgi?tool=bioconductor&cmd=Retrieve&db=PubMed&list_uids=9458045%2C9608531%2C9796701%2C10102268%2C10433822%2C10892742%2C11672528%2C11748139%2C12082532%2C12471613%2C12477932%2C12504588%2C12892710%2C14702039%2C15207848%2C15342556%2C15489334%2C16169070%2C16254601%2C16636676%2C16641997%2C16685377%2C16740745%2C17062560%2C17081983%2C17671114%2C17671369%2C17848514%2C17968499%2C18270976%2C18387595%2C18464913%2C18566128%2C18829537%2C18948384%2C19023125%2C19104841%2C19351956%2C19706539%2C20029409%2C20068157%2C20071679%2C20298689%2C20300657%2C20379614%2C20438712%2C20471383%2C20816195%2C20936779%2C20949370%2C21041608) | [cell migration involved in sprouting angiogenesis](http://amigo.geneontology.org/cgi-bin/amigo/go.cgi?view=details&query=GO:0002042)  [receptor activity](http://amigo.geneontology.org/cgi-bin/amigo/go.cgi?view=details&query=GO:0004872)  [protein binding](http://amigo.geneontology.org/cgi-bin/amigo/go.cgi?view=details&query=GO:0005515)  [cytoplasm](http://amigo.geneontology.org/cgi-bin/amigo/go.cgi?view=details&query=GO:0005737)  [plasma membrane](http://amigo.geneontology.org/cgi-bin/amigo/go.cgi?view=details&query=GO:0005886)  [plasma membrane](http://amigo.geneontology.org/cgi-bin/amigo/go.cgi?view=details&query=GO:0005886)  [integral to plasma membrane](http://amigo.geneontology.org/cgi-bin/amigo/go.cgi?view=details&query=GO:0005887)  [activation of caspase activity](http://amigo.geneontology.org/cgi-bin/amigo/go.cgi?view=details&query=GO:0006919)  [cell adhesion](http://amigo.geneontology.org/cgi-bin/amigo/go.cgi?view=details&query=GO:0007155)  [homophilic cell adhesion](http://amigo.geneontology.org/cgi-bin/amigo/go.cgi?view=details&query=GO:0007156)  [multicellular organismal development](http://amigo.geneontology.org/cgi-bin/amigo/go.cgi?view=details&query=GO:0007275)  [nervous system development](http://amigo.geneontology.org/cgi-bin/amigo/go.cgi?view=details&query=GO:0007399)  [axon guidance](http://amigo.geneontology.org/cgi-bin/amigo/go.cgi?view=details&query=GO:0007411)  [axon guidance receptor activity](http://amigo.geneontology.org/cgi-bin/amigo/go.cgi?view=details&query=GO:0008046)  [cell surface](http://amigo.geneontology.org/cgi-bin/amigo/go.cgi?view=details&query=GO:0009986)  [axon midline choice point recognition](http://amigo.geneontology.org/cgi-bin/amigo/go.cgi?view=details&query=GO:0016199)  [chemorepulsion involved in postnatal olfactory bulb interneuron migration](http://amigo.geneontology.org/cgi-bin/amigo/go.cgi?view=details&query=GO:0021836)  [cell differentiation](http://amigo.geneontology.org/cgi-bin/amigo/go.cgi?view=details&query=GO:0030154)  [LRR domain binding](http://amigo.geneontology.org/cgi-bin/amigo/go.cgi?view=details&query=GO:0030275)  [negative regulation of mammary gland epithelial cell proliferation](http://amigo.geneontology.org/cgi-bin/amigo/go.cgi?view=details&query=GO:0033600)  [Roundabout signaling pathway](http://amigo.geneontology.org/cgi-bin/amigo/go.cgi?view=details&query=GO:0035385)  [identical protein binding](http://amigo.geneontology.org/cgi-bin/amigo/go.cgi?view=details&query=GO:0042802)  [identical protein binding](http://amigo.geneontology.org/cgi-bin/amigo/go.cgi?view=details&query=GO:0042802)  [positive regulation of axonogenesis](http://amigo.geneontology.org/cgi-bin/amigo/go.cgi?view=details&query=GO:0050772)  [negative regulation of negative chemotaxis](http://amigo.geneontology.org/cgi-bin/amigo/go.cgi?view=details&query=GO:0050925)  [negative regulation of chemokine-mediated signaling pathway](http://amigo.geneontology.org/cgi-bin/amigo/go.cgi?view=details&query=GO:0070100) | [Axon guidance](http://www.genome.ad.jp/kegg/pathway/hsa/hsa04360.html) |
| [213340_s_at](https://www.affymetrix.com/LinkServlet?&probeset=213340_s_at) | KIAA0495 | KIAA0495 | 1 | -3652549, -3652549 | [AI073551](http://www.ncbi.nlm.nih.gov/entrez/query.fcgi?cmd=search&db=nucleotide&term=AI073551%5BACCN%5D&doptcmdl=GenBank) | [57212](http://www.ncbi.nlm.nih.gov/sites/entrez?Db=gene&Cmd=DetailsSearch&Term=57212) | [1p36.32](http://www.ncbi.nlm.nih.gov/mapview/map_search.cgi?direct=on&idtype=gene&id=57212) | [Hs.49658](http://www.ncbi.nlm.nih.gov/UniGene/clust.cgi?ORG=Hs&CID=49658) | [3](http://www.ncbi.nih.gov/entrez/query.fcgi?tool=bioconductor&cmd=Retrieve&db=PubMed&list_uids=9455484%2C12477932%2C20477830) | [extracellular region](http://amigo.geneontology.org/cgi-bin/amigo/go.cgi?view=details&query=GO:0005576) |  |
| [213419_at](https://www.affymetrix.com/LinkServlet?&probeset=213419_at) | APBB2 | amyloid beta (A4) precursor protein-binding, family B, member 2 | 4 | -40812044, -40812044, -40812044 | [U62325](http://www.ncbi.nlm.nih.gov/entrez/query.fcgi?cmd=search&db=nucleotide&term=U62325%5BACCN%5D&doptcmdl=GenBank) | [323](http://www.ncbi.nlm.nih.gov/sites/entrez?Db=gene&Cmd=DetailsSearch&Term=323) | [4p13](http://www.ncbi.nlm.nih.gov/mapview/map_search.cgi?direct=on&idtype=gene&id=323) | [Hs.479602](http://www.ncbi.nlm.nih.gov/UniGene/clust.cgi?ORG=Hs&CID=479602) | [15](http://www.ncbi.nih.gov/entrez/query.fcgi?tool=bioconductor&cmd=Retrieve&db=PubMed&list_uids=8855266%2C8955346%2C9585438%2C12089154%2C12477932%2C14527950%2C15489334%2C15714520%2C15761153%2C16083851%2C16344560%2C17567906%2C18852029%2C19542221%2C20677014) | [beta-amyloid binding](http://amigo.geneontology.org/cgi-bin/amigo/go.cgi?view=details&query=GO:0001540)  [protein binding](http://amigo.geneontology.org/cgi-bin/amigo/go.cgi?view=details&query=GO:0005515)  [nucleus](http://amigo.geneontology.org/cgi-bin/amigo/go.cgi?view=details&query=GO:0005634)  [cell cycle arrest](http://amigo.geneontology.org/cgi-bin/amigo/go.cgi?view=details&query=GO:0007050)  [transcription factor binding](http://amigo.geneontology.org/cgi-bin/amigo/go.cgi?view=details&query=GO:0008134)  [membrane](http://amigo.geneontology.org/cgi-bin/amigo/go.cgi?view=details&query=GO:0016020)  [lamellipodium](http://amigo.geneontology.org/cgi-bin/amigo/go.cgi?view=details&query=GO:0030027)  [negative regulation of cell growth](http://amigo.geneontology.org/cgi-bin/amigo/go.cgi?view=details&query=GO:0030308)  [growth cone](http://amigo.geneontology.org/cgi-bin/amigo/go.cgi?view=details&query=GO:0030426)  [intracellular signal transduction](http://amigo.geneontology.org/cgi-bin/amigo/go.cgi?view=details&query=GO:0035556)  [synapse](http://amigo.geneontology.org/cgi-bin/amigo/go.cgi?view=details&query=GO:0045202)  [regulation of transcription](http://amigo.geneontology.org/cgi-bin/amigo/go.cgi?view=details&query=GO:0045449)  [negative regulation of S phase of mitotic cell cycle](http://amigo.geneontology.org/cgi-bin/amigo/go.cgi?view=details&query=GO:0045749) |  |
| [213423_x_at](https://www.affymetrix.com/LinkServlet?&probeset=213423_x_at) | TUSC3 | tumor suppressor candidate 3 | 8 | 15397729 | [AI884858](http://www.ncbi.nlm.nih.gov/entrez/query.fcgi?cmd=search&db=nucleotide&term=AI884858%5BACCN%5D&doptcmdl=GenBank) | [7991](http://www.ncbi.nlm.nih.gov/sites/entrez?Db=gene&Cmd=DetailsSearch&Term=7991) | [8p22](http://www.ncbi.nlm.nih.gov/mapview/map_search.cgi?direct=on&idtype=gene&id=7991) | [Hs.591845](http://www.ncbi.nlm.nih.gov/UniGene/clust.cgi?ORG=Hs&CID=591845) [Hs.613699](http://www.ncbi.nlm.nih.gov/UniGene/clust.cgi?ORG=Hs&CID=613699) | [15](http://www.ncbi.nih.gov/entrez/query.fcgi?tool=bioconductor&cmd=Retrieve&db=PubMed&list_uids=8661104%2C9778121%2C10097140%2C12477932%2C12887896%2C14718574%2C15231748%2C15489334%2C15835887%2C16270321%2C16344560%2C17641416%2C18452889%2C18455129%2C19717468) | [dolichyl-diphosphooligosaccharide-protein glycotransferase activity](http://amigo.geneontology.org/cgi-bin/amigo/go.cgi?view=details&query=GO:0004579)  [endoplasmic reticulum](http://amigo.geneontology.org/cgi-bin/amigo/go.cgi?view=details&query=GO:0005783)  [endoplasmic reticulum membrane](http://amigo.geneontology.org/cgi-bin/amigo/go.cgi?view=details&query=GO:0005789)  [oligosaccharyltransferase complex](http://amigo.geneontology.org/cgi-bin/amigo/go.cgi?view=details&query=GO:0008250)  [membrane](http://amigo.geneontology.org/cgi-bin/amigo/go.cgi?view=details&query=GO:0016020)  [integral to membrane](http://amigo.geneontology.org/cgi-bin/amigo/go.cgi?view=details&query=GO:0016021)  [protein N-linked glycosylation via asparagine](http://amigo.geneontology.org/cgi-bin/amigo/go.cgi?view=details&query=GO:0018279)  [post-translational protein modification](http://amigo.geneontology.org/cgi-bin/amigo/go.cgi?view=details&query=GO:0043687)  [cellular protein metabolic process](http://amigo.geneontology.org/cgi-bin/amigo/go.cgi?view=details&query=GO:0044267)  [cell redox homeostasis](http://amigo.geneontology.org/cgi-bin/amigo/go.cgi?view=details&query=GO:0045454) | [N-Glycan biosynthesis](http://www.genome.ad.jp/kegg/pathway/hsa/hsa00510.html)  [Metabolic pathways](http://www.genome.ad.jp/kegg/pathway/hsa/hsa01100.html)  [Protein processing in endoplasmic reticulum](http://www.genome.ad.jp/kegg/pathway/hsa/hsa04141.html) |
| [213568_at](https://www.affymetrix.com/LinkServlet?&probeset=213568_at) | OSR2 | odd-skipped related 2 (Drosophila) | 8 | 99956630 | [AI811298](http://www.ncbi.nlm.nih.gov/entrez/query.fcgi?cmd=search&db=nucleotide&term=AI811298%5BACCN%5D&doptcmdl=GenBank) | [116039](http://www.ncbi.nlm.nih.gov/sites/entrez?Db=gene&Cmd=DetailsSearch&Term=116039) | [8q22.2](http://www.ncbi.nlm.nih.gov/mapview/map_search.cgi?direct=on&idtype=gene&id=116039) | [Hs.253247](http://www.ncbi.nlm.nih.gov/UniGene/clust.cgi?ORG=Hs&CID=253247) | [7](http://www.ncbi.nih.gov/entrez/query.fcgi?tool=bioconductor&cmd=Retrieve&db=PubMed&list_uids=11520675%2C12477932%2C14702039%2C15175245%2C15489334%2C15670784%2C20634891) | [nucleic acid binding](http://amigo.geneontology.org/cgi-bin/amigo/go.cgi?view=details&query=GO:0003676)  [intracellular](http://amigo.geneontology.org/cgi-bin/amigo/go.cgi?view=details&query=GO:0005622)  [nucleus](http://amigo.geneontology.org/cgi-bin/amigo/go.cgi?view=details&query=GO:0005634)  [zinc ion binding](http://amigo.geneontology.org/cgi-bin/amigo/go.cgi?view=details&query=GO:0008270)  [transcription activator activity](http://amigo.geneontology.org/cgi-bin/amigo/go.cgi?view=details&query=GO:0016563)  [osteoblast proliferation](http://amigo.geneontology.org/cgi-bin/amigo/go.cgi?view=details&query=GO:0033687)  [middle ear morphogenesis](http://amigo.geneontology.org/cgi-bin/amigo/go.cgi?view=details&query=GO:0042474)  [odontogenesis](http://amigo.geneontology.org/cgi-bin/amigo/go.cgi?view=details&query=GO:0042476)  [sequence-specific DNA binding](http://amigo.geneontology.org/cgi-bin/amigo/go.cgi?view=details&query=GO:0043565)  [metal ion binding](http://amigo.geneontology.org/cgi-bin/amigo/go.cgi?view=details&query=GO:0046872)  [embryonic skeletal system morphogenesis](http://amigo.geneontology.org/cgi-bin/amigo/go.cgi?view=details&query=GO:0048704)  [positive regulation of epithelial cell proliferation](http://amigo.geneontology.org/cgi-bin/amigo/go.cgi?view=details&query=GO:0050679)  [palate development](http://amigo.geneontology.org/cgi-bin/amigo/go.cgi?view=details&query=GO:0060021)  [bone morphogenesis](http://amigo.geneontology.org/cgi-bin/amigo/go.cgi?view=details&query=GO:0060349)  [eyelid development in camera-type eye](http://amigo.geneontology.org/cgi-bin/amigo/go.cgi?view=details&query=GO:0061029) |  |
| [213578_at](https://www.affymetrix.com/LinkServlet?&probeset=213578_at) | BMPR1A | bone morphogenetic protein receptor, type IA | 10 | 88516395 | [AI678679](http://www.ncbi.nlm.nih.gov/entrez/query.fcgi?cmd=search&db=nucleotide&term=AI678679%5BACCN%5D&doptcmdl=GenBank) | [657](http://www.ncbi.nlm.nih.gov/sites/entrez?Db=gene&Cmd=DetailsSearch&Term=657) | [10q22.3](http://www.ncbi.nlm.nih.gov/mapview/map_search.cgi?direct=on&idtype=gene&id=657) | [Hs.524477](http://www.ncbi.nlm.nih.gov/UniGene/clust.cgi?ORG=Hs&CID=524477) | [87](http://www.ncbi.nih.gov/entrez/query.fcgi?tool=bioconductor&cmd=Retrieve&db=PubMed&list_uids=7644468%2C7791754%2C8006002%2C8397373%2C8592941%2C8605097%2C8702914%2C8707881%2C9389648%2C9525338%2C9547239%2C9663660%2C9730621%2C9738003%2C9950587%2C10051328%2C10504300%2C10692589%2C10712517%2C10850425%2C10880444%2C10881198%2C11139569%2C11241215%2C11263668%2C11278302%2C11282024%2C11381269%2C11401330%2C11438941%2C11536076%2C11580864%2C12065756%2C12135884%2C12136244%2C12417513%2C12477932%2C12620973%2C12630959%2C15064755%2C15148321%2C15235019%2C15252450%2C15351706%2C15489334%2C15621726%2C15657086%2C15940369%2C16226113%2C16341674%2C16385451%2C16436528%2C16436638%2C16525031%2C16613856%2C16672363%2C16886151%2C17081983%2C17101085%2C17356069%2C17513295%2C17573831%2C17624341%2C17873119%2C18160401%2C18178612%2C18184661%2C18262054%2C18436533%2C18510548%2C18667463%2C18823382%2C18937504%2C19244313%2C19438883%2C19453261%2C19463221%2C19502417%2C19773747%2C20301642%2C20346360%2C20587070%2C20634891%2C20693682%2C20734064%2C20927405%2C21152263) | [nucleotide binding](http://amigo.geneontology.org/cgi-bin/amigo/go.cgi?view=details&query=GO:0000166)  [protein serine/threonine kinase activity](http://amigo.geneontology.org/cgi-bin/amigo/go.cgi?view=details&query=GO:0004674)  [receptor activity](http://amigo.geneontology.org/cgi-bin/amigo/go.cgi?view=details&query=GO:0004872)  [transforming growth factor beta receptor activity](http://amigo.geneontology.org/cgi-bin/amigo/go.cgi?view=details&query=GO:0005024)  [protein binding](http://amigo.geneontology.org/cgi-bin/amigo/go.cgi?view=details&query=GO:0005515)  [ATP binding](http://amigo.geneontology.org/cgi-bin/amigo/go.cgi?view=details&query=GO:0005524)  [plasma membrane](http://amigo.geneontology.org/cgi-bin/amigo/go.cgi?view=details&query=GO:0005886)  [plasma membrane](http://amigo.geneontology.org/cgi-bin/amigo/go.cgi?view=details&query=GO:0005886)  [plasma membrane](http://amigo.geneontology.org/cgi-bin/amigo/go.cgi?view=details&query=GO:0005886)  [caveola](http://amigo.geneontology.org/cgi-bin/amigo/go.cgi?view=details&query=GO:0005901)  [protein phosphorylation](http://amigo.geneontology.org/cgi-bin/amigo/go.cgi?view=details&query=GO:0006468)  [immune response](http://amigo.geneontology.org/cgi-bin/amigo/go.cgi?view=details&query=GO:0006955)  [transforming growth factor beta receptor signaling pathway](http://amigo.geneontology.org/cgi-bin/amigo/go.cgi?view=details&query=GO:0007179)  [positive regulation of pathway-restricted SMAD protein phosphorylation](http://amigo.geneontology.org/cgi-bin/amigo/go.cgi?view=details&query=GO:0010862)  [integral to membrane](http://amigo.geneontology.org/cgi-bin/amigo/go.cgi?view=details&query=GO:0016021)  [positive regulation of bone mineralization](http://amigo.geneontology.org/cgi-bin/amigo/go.cgi?view=details&query=GO:0030501)  [BMP signaling pathway](http://amigo.geneontology.org/cgi-bin/amigo/go.cgi?view=details&query=GO:0030509)  [BMP signaling pathway](http://amigo.geneontology.org/cgi-bin/amigo/go.cgi?view=details&query=GO:0030509)  [protein homodimerization activity](http://amigo.geneontology.org/cgi-bin/amigo/go.cgi?view=details&query=GO:0042803)  [positive regulation of osteoblast differentiation](http://amigo.geneontology.org/cgi-bin/amigo/go.cgi?view=details&query=GO:0045669)  [SMAD binding](http://amigo.geneontology.org/cgi-bin/amigo/go.cgi?view=details&query=GO:0046332)  [metal ion binding](http://amigo.geneontology.org/cgi-bin/amigo/go.cgi?view=details&query=GO:0046872)  [positive regulation of SMAD protein import into nucleus](http://amigo.geneontology.org/cgi-bin/amigo/go.cgi?view=details&query=GO:0060391) | [Cytokine-cytokine receptor interaction](http://www.genome.ad.jp/dbget-bin/show_pathway?MAP04060+2.7.11.30)  [TGF-beta signaling pathway](http://www.genome.ad.jp/dbget-bin/show_pathway?MAP04350+2.7.11.30) |
| [213929_at](https://www.affymetrix.com/LinkServlet?&probeset=213929_at) | EXPH5 | exophilin 5 | 11 | -108376158, -108376158, -108376158 | [AL050204](http://www.ncbi.nlm.nih.gov/entrez/query.fcgi?cmd=search&db=nucleotide&term=AL050204%5BACCN%5D&doptcmdl=GenBank) | [23086](http://www.ncbi.nlm.nih.gov/sites/entrez?Db=gene&Cmd=DetailsSearch&Term=23086) | [11q22.3](http://www.ncbi.nlm.nih.gov/mapview/map_search.cgi?direct=on&idtype=gene&id=23086) | [Hs.28540](http://www.ncbi.nlm.nih.gov/UniGene/clust.cgi?ORG=Hs&CID=28540) | [10](http://www.ncbi.nih.gov/entrez/query.fcgi?tool=bioconductor&cmd=Retrieve&db=PubMed&list_uids=9734811%2C11773082%2C12062444%2C12189142%2C12477932%2C12590134%2C15231748%2C15342556%2C15489334%2C20936779) | [intracellular protein transport](http://amigo.geneontology.org/cgi-bin/amigo/go.cgi?view=details&query=GO:0006886)  [Rab GTPase binding](http://amigo.geneontology.org/cgi-bin/amigo/go.cgi?view=details&query=GO:0017137) |  |
| [213943_at](https://www.affymetrix.com/LinkServlet?&probeset=213943_at) | TWIST1 | twist homolog 1 (Drosophila) | 7 | -19155092 | [X99268](http://www.ncbi.nlm.nih.gov/entrez/query.fcgi?cmd=search&db=nucleotide&term=X99268%5BACCN%5D&doptcmdl=GenBank) | [7291](http://www.ncbi.nlm.nih.gov/sites/entrez?Db=gene&Cmd=DetailsSearch&Term=7291) | [7p21.2](http://www.ncbi.nlm.nih.gov/mapview/map_search.cgi?direct=on&idtype=gene&id=7291) | [Hs.66744](http://www.ncbi.nlm.nih.gov/UniGene/clust.cgi?ORG=Hs&CID=66744) | [134](http://www.ncbi.nih.gov/entrez/query.fcgi?tool=bioconductor&cmd=Retrieve&db=PubMed&list_uids=1433226%2C7296937%2C7987323%2C8968762%2C8988166%2C8988167%2C8995765%2C9073070%2C9215678%2C9259286%2C9343420%2C9934984%2C10025406%2C10465122%2C10485844%2C10749989%2C11062344%2C11474656%2C11748846%2C11754069%2C11854168%2C11948912%2C11977182%2C12015302%2C12142027%2C12218784%2C12270142%2C12477932%2C12553906%2C12690205%2C12791045%2C12853948%2C14513358%2C14724576%2C15210113%2C15313894%2C15489334%2C15545268%2C15555546%2C15607966%2C15735646%2C15880747%2C15900593%2C15958559%2C16229805%2C16293629%2C16322226%2C16412561%2C16502419%2C16540516%2C16737925%2C16831897%2C16888803%2C17003487%2C17070479%2C17157810%2C17236203%2C17332324%2C17332325%2C17343269%2C17394502%2C17403902%2C17414280%2C17487558%2C17512904%2C17690110%2C17785550%2C17886095%2C17893140%2C17925286%2C17967182%2C17987801%2C18062917%2C18172301%2C18231738%2C18255367%2C18297062%2C18349132%2C18353781%2C18391499%2C18440840%2C18480983%2C18504427%2C18519689%2C18549475%2C18598946%2C18663125%2C18974133%2C19051271%2C19064546%2C19094228%2C19276370%2C19345188%2C19373776%2C19381684%2C19412634%2C19453261%2C19470737%2C19513566%2C19534813%2C19597909%2C19609939%2C19644484%2C19802001%2C19816777%2C19821482%2C19851501%2C19860490%2C19863427%2C19893041%2C20007935%2C20019840%2C20025748%2C20140954%2C20301368%2C20400975%2C20400976%2C20496080%2C20505321%2C20562331%2C20564230%2C20628624%2C20634891%2C20643727%2C20646316%2C20696219%2C20804746%2C20818389%2C20838384%2C20857420%2C20920982%2C20943789%2C20970163%2C21199805) | [negative regulation of transcription from RNA polymerase II promoter](http://amigo.geneontology.org/cgi-bin/amigo/go.cgi?view=details&query=GO:0000122)  [skeletal system development](http://amigo.geneontology.org/cgi-bin/amigo/go.cgi?view=details&query=GO:0001501)  [neuron migration](http://amigo.geneontology.org/cgi-bin/amigo/go.cgi?view=details&query=GO:0001764)  [neural tube closure](http://amigo.geneontology.org/cgi-bin/amigo/go.cgi?view=details&query=GO:0001843)  [DNA binding](http://amigo.geneontology.org/cgi-bin/amigo/go.cgi?view=details&query=GO:0003677)  [sequence-specific DNA binding transcription factor activity](http://amigo.geneontology.org/cgi-bin/amigo/go.cgi?view=details&query=GO:0003700)  [enzyme inhibitor activity](http://amigo.geneontology.org/cgi-bin/amigo/go.cgi?view=details&query=GO:0004857)  [protein binding](http://amigo.geneontology.org/cgi-bin/amigo/go.cgi?view=details&query=GO:0005515)  [nucleus](http://amigo.geneontology.org/cgi-bin/amigo/go.cgi?view=details&query=GO:0005634)  [apoptosis](http://amigo.geneontology.org/cgi-bin/amigo/go.cgi?view=details&query=GO:0006915)  [multicellular organismal development](http://amigo.geneontology.org/cgi-bin/amigo/go.cgi?view=details&query=GO:0007275)  [muscle organ development](http://amigo.geneontology.org/cgi-bin/amigo/go.cgi?view=details&query=GO:0007517)  [transcription factor binding](http://amigo.geneontology.org/cgi-bin/amigo/go.cgi?view=details&query=GO:0008134)  [anatomical structure morphogenesis](http://amigo.geneontology.org/cgi-bin/amigo/go.cgi?view=details&query=GO:0009653)  [transcription regulator activity](http://amigo.geneontology.org/cgi-bin/amigo/go.cgi?view=details&query=GO:0030528)  [embryonic forelimb morphogenesis](http://amigo.geneontology.org/cgi-bin/amigo/go.cgi?view=details&query=GO:0035115)  [hindlimb morphogenesis](http://amigo.geneontology.org/cgi-bin/amigo/go.cgi?view=details&query=GO:0035137)  [protein homodimerization activity](http://amigo.geneontology.org/cgi-bin/amigo/go.cgi?view=details&query=GO:0042803)  [negative regulation of cell differentiation](http://amigo.geneontology.org/cgi-bin/amigo/go.cgi?view=details&query=GO:0045596)  [negative regulation of striated muscle tissue development](http://amigo.geneontology.org/cgi-bin/amigo/go.cgi?view=details&query=GO:0045843)  [positive regulation of transcription from RNA polymerase II promoter](http://amigo.geneontology.org/cgi-bin/amigo/go.cgi?view=details&query=GO:0045944)  [protein heterodimerization activity](http://amigo.geneontology.org/cgi-bin/amigo/go.cgi?view=details&query=GO:0046982)  [cranial suture morphogenesis](http://amigo.geneontology.org/cgi-bin/amigo/go.cgi?view=details&query=GO:0060363)  [E-box binding](http://amigo.geneontology.org/cgi-bin/amigo/go.cgi?view=details&query=GO:0070888) |  |
| [214039_s_at](https://www.affymetrix.com/LinkServlet?&probeset=214039_s_at) | LAPTM4B | lysosomal protein transmembrane 4 beta | 8 | 98787808 | [T15777](http://www.ncbi.nlm.nih.gov/entrez/query.fcgi?cmd=search&db=nucleotide&term=T15777%5BACCN%5D&doptcmdl=GenBank) | [55353](http://www.ncbi.nlm.nih.gov/sites/entrez?Db=gene&Cmd=DetailsSearch&Term=55353) | [8q22.1](http://www.ncbi.nlm.nih.gov/mapview/map_search.cgi?direct=on&idtype=gene&id=55353) | [Hs.492314](http://www.ncbi.nlm.nih.gov/UniGene/clust.cgi?ORG=Hs&CID=492314) | [24](http://www.ncbi.nih.gov/entrez/query.fcgi?tool=bioconductor&cmd=Retrieve&db=PubMed&list_uids=11076863%2C11230166%2C12477932%2C12640618%2C12902989%2C15162524%2C15489334%2C15489336%2C15911104%2C15968325%2C16303743%2C16381901%2C16769693%2C17074969%2C17965115%2C18334282%2C18949404%2C19690886%2C19843073%2C20358632%2C20379614%2C20583413%2C20711237%2C20881850) | [protein binding](http://amigo.geneontology.org/cgi-bin/amigo/go.cgi?view=details&query=GO:0005515)  [transport](http://amigo.geneontology.org/cgi-bin/amigo/go.cgi?view=details&query=GO:0006810)  [endomembrane system](http://amigo.geneontology.org/cgi-bin/amigo/go.cgi?view=details&query=GO:0012505)  [membrane](http://amigo.geneontology.org/cgi-bin/amigo/go.cgi?view=details&query=GO:0016020)  [integral to membrane](http://amigo.geneontology.org/cgi-bin/amigo/go.cgi?view=details&query=GO:0016021) | [Lysosome](http://www.genome.ad.jp/kegg/pathway/hsa/hsa04142.html) |
| [214097_at](https://www.affymetrix.com/LinkServlet?&probeset=214097_at) | RPS21 | ribosomal protein S21 | 20 | 60962120 | [AW024383](http://www.ncbi.nlm.nih.gov/entrez/query.fcgi?cmd=search&db=nucleotide&term=AW024383%5BACCN%5D&doptcmdl=GenBank) | [6227](http://www.ncbi.nlm.nih.gov/sites/entrez?Db=gene&Cmd=DetailsSearch&Term=6227) | [20q13.3](http://www.ncbi.nlm.nih.gov/mapview/map_search.cgi?direct=on&idtype=gene&id=6227) | [Hs.190968](http://www.ncbi.nlm.nih.gov/UniGene/clust.cgi?ORG=Hs&CID=190968) | [19](http://www.ncbi.nih.gov/entrez/query.fcgi?tool=bioconductor&cmd=Retrieve&db=PubMed&list_uids=7786314%2C7805865%2C8332502%2C8706699%2C8722009%2C8889548%2C9582194%2C10079194%2C10900511%2C11780052%2C11875025%2C12477932%2C12588972%2C15189156%2C15883184%2C16169070%2C16189514%2C16341674%2C18029348) | [endonucleolytic cleavage in ITS1 to separate SSU-rRNA from 5.8S rRNA and LSU-rRNA from tricistronic rRNA transcript (SSU-rRNA, 5.8S rRNA, LSU-rRNA)](http://amigo.geneontology.org/cgi-bin/amigo/go.cgi?view=details&query=GO:0000447)  [endonucleolytic cleavage to generate mature 3'-end of SSU-rRNA from (SSU-rRNA, 5.8S rRNA, LSU-rRNA)](http://amigo.geneontology.org/cgi-bin/amigo/go.cgi?view=details&query=GO:0000461)  [structural constituent of ribosome](http://amigo.geneontology.org/cgi-bin/amigo/go.cgi?view=details&query=GO:0003735)  [intracellular](http://amigo.geneontology.org/cgi-bin/amigo/go.cgi?view=details&query=GO:0005622)  [cytoplasm](http://amigo.geneontology.org/cgi-bin/amigo/go.cgi?view=details&query=GO:0005737)  [cytosol](http://amigo.geneontology.org/cgi-bin/amigo/go.cgi?view=details&query=GO:0005829)  [cytosol](http://amigo.geneontology.org/cgi-bin/amigo/go.cgi?view=details&query=GO:0005829)  [translation](http://amigo.geneontology.org/cgi-bin/amigo/go.cgi?view=details&query=GO:0006412)  [translation](http://amigo.geneontology.org/cgi-bin/amigo/go.cgi?view=details&query=GO:0006412)  [translation](http://amigo.geneontology.org/cgi-bin/amigo/go.cgi?view=details&query=GO:0006412)  [translational elongation](http://amigo.geneontology.org/cgi-bin/amigo/go.cgi?view=details&query=GO:0006414)  [translational termination](http://amigo.geneontology.org/cgi-bin/amigo/go.cgi?view=details&query=GO:0006415)  [gene expression](http://amigo.geneontology.org/cgi-bin/amigo/go.cgi?view=details&query=GO:0010467)  [small ribosomal subunit](http://amigo.geneontology.org/cgi-bin/amigo/go.cgi?view=details&query=GO:0015935)  [viral reproduction](http://amigo.geneontology.org/cgi-bin/amigo/go.cgi?view=details&query=GO:0016032)  [viral infectious cycle](http://amigo.geneontology.org/cgi-bin/amigo/go.cgi?view=details&query=GO:0019058)  [viral transcription](http://amigo.geneontology.org/cgi-bin/amigo/go.cgi?view=details&query=GO:0019083)  [cytosolic small ribosomal subunit](http://amigo.geneontology.org/cgi-bin/amigo/go.cgi?view=details&query=GO:0022627)  [cytosolic small ribosomal subunit](http://amigo.geneontology.org/cgi-bin/amigo/go.cgi?view=details&query=GO:0022627)  [endocrine pancreas development](http://amigo.geneontology.org/cgi-bin/amigo/go.cgi?view=details&query=GO:0031018)  [ribosomal small subunit biogenesis](http://amigo.geneontology.org/cgi-bin/amigo/go.cgi?view=details&query=GO:0042274)  [cellular protein metabolic process](http://amigo.geneontology.org/cgi-bin/amigo/go.cgi?view=details&query=GO:0044267)  [protein N-terminus binding](http://amigo.geneontology.org/cgi-bin/amigo/go.cgi?view=details&query=GO:0047485) | [Ribosome](http://www.genome.ad.jp/kegg/pathway/hsa/hsa03010.html) |
| [214436_at](https://www.affymetrix.com/LinkServlet?&probeset=214436_at) | FBXL2 | F-box and leucine-rich repeat protein 2 | 3 | 33318933, 33318936 | [AF176518](http://www.ncbi.nlm.nih.gov/entrez/query.fcgi?cmd=search&db=nucleotide&term=AF176518%5BACCN%5D&doptcmdl=GenBank) | [25827](http://www.ncbi.nlm.nih.gov/sites/entrez?Db=gene&Cmd=DetailsSearch&Term=25827) | [3p22.3](http://www.ncbi.nlm.nih.gov/mapview/map_search.cgi?direct=on&idtype=gene&id=25827) | [Hs.475872](http://www.ncbi.nlm.nih.gov/UniGene/clust.cgi?ORG=Hs&CID=475872) | [11](http://www.ncbi.nih.gov/entrez/query.fcgi?tool=bioconductor&cmd=Retrieve&db=PubMed&list_uids=10508920%2C10531035%2C10531037%2C10945468%2C12477932%2C14702039%2C15489334%2C15893726%2C16169070%2C16344560%2C17784784) | [ubiquitin-protein ligase activity](http://amigo.geneontology.org/cgi-bin/amigo/go.cgi?view=details&query=GO:0004842)  [cytoplasm](http://amigo.geneontology.org/cgi-bin/amigo/go.cgi?view=details&query=GO:0005737)  [protein modification process](http://amigo.geneontology.org/cgi-bin/amigo/go.cgi?view=details&query=GO:0006464)  [proteolysis](http://amigo.geneontology.org/cgi-bin/amigo/go.cgi?view=details&query=GO:0006508)  [membrane](http://amigo.geneontology.org/cgi-bin/amigo/go.cgi?view=details&query=GO:0016020)  [protein ubiquitination](http://amigo.geneontology.org/cgi-bin/amigo/go.cgi?view=details&query=GO:0016567)  [interspecies interaction between organisms](http://amigo.geneontology.org/cgi-bin/amigo/go.cgi?view=details&query=GO:0044419) |  |
| [214734_at](https://www.affymetrix.com/LinkServlet?&probeset=214734_at) | EXPH5 | exophilin 5 | 11 | -108376158, -108376158, -108376158 | [AB014524](http://www.ncbi.nlm.nih.gov/entrez/query.fcgi?cmd=search&db=nucleotide&term=AB014524%5BACCN%5D&doptcmdl=GenBank) | [23086](http://www.ncbi.nlm.nih.gov/sites/entrez?Db=gene&Cmd=DetailsSearch&Term=23086) | [11q22.3](http://www.ncbi.nlm.nih.gov/mapview/map_search.cgi?direct=on&idtype=gene&id=23086) | [Hs.28540](http://www.ncbi.nlm.nih.gov/UniGene/clust.cgi?ORG=Hs&CID=28540) | [10](http://www.ncbi.nih.gov/entrez/query.fcgi?tool=bioconductor&cmd=Retrieve&db=PubMed&list_uids=9734811%2C11773082%2C12062444%2C12189142%2C12477932%2C12590134%2C15231748%2C15342556%2C15489334%2C20936779) | [intracellular protein transport](http://amigo.geneontology.org/cgi-bin/amigo/go.cgi?view=details&query=GO:0006886)  [Rab GTPase binding](http://amigo.geneontology.org/cgi-bin/amigo/go.cgi?view=details&query=GO:0017137) |  |
| [214829_at](https://www.affymetrix.com/LinkServlet?&probeset=214829_at) | AASS | aminoadipate-semialdehyde synthase | 7 | -121713599 | [AK023446](http://www.ncbi.nlm.nih.gov/entrez/query.fcgi?cmd=search&db=nucleotide&term=AK023446%5BACCN%5D&doptcmdl=GenBank) | [10157](http://www.ncbi.nlm.nih.gov/sites/entrez?Db=gene&Cmd=DetailsSearch&Term=10157) | [7q31.3](http://www.ncbi.nlm.nih.gov/mapview/map_search.cgi?direct=on&idtype=gene&id=10157) | [Hs.156738](http://www.ncbi.nlm.nih.gov/UniGene/clust.cgi?ORG=Hs&CID=156738) | [13](http://www.ncbi.nih.gov/entrez/query.fcgi?tool=bioconductor&cmd=Retrieve&db=PubMed&list_uids=8125298%2C8889548%2C9373149%2C9847074%2C10567240%2C10775527%2C12477932%2C12690205%2C12853948%2C14702039%2C16344560%2C18029348%2C20877624) | [binding](http://amigo.geneontology.org/cgi-bin/amigo/go.cgi?view=details&query=GO:0005488)  [cytoplasm](http://amigo.geneontology.org/cgi-bin/amigo/go.cgi?view=details&query=GO:0005737)  [mitochondrion](http://amigo.geneontology.org/cgi-bin/amigo/go.cgi?view=details&query=GO:0005739)  [mitochondrion](http://amigo.geneontology.org/cgi-bin/amigo/go.cgi?view=details&query=GO:0005739)  [mitochondrial matrix](http://amigo.geneontology.org/cgi-bin/amigo/go.cgi?view=details&query=GO:0005759)  [lysine catabolic process](http://amigo.geneontology.org/cgi-bin/amigo/go.cgi?view=details&query=GO:0006554)  [lysine catabolic process](http://amigo.geneontology.org/cgi-bin/amigo/go.cgi?view=details&query=GO:0006554)  [oxidoreductase activity](http://amigo.geneontology.org/cgi-bin/amigo/go.cgi?view=details&query=GO:0016491)  [L-lysine catabolic process](http://amigo.geneontology.org/cgi-bin/amigo/go.cgi?view=details&query=GO:0019477)  [cellular nitrogen compound metabolic process](http://amigo.geneontology.org/cgi-bin/amigo/go.cgi?view=details&query=GO:0034641)  [intracellular membrane-bounded organelle](http://amigo.geneontology.org/cgi-bin/amigo/go.cgi?view=details&query=GO:0043231)  [saccharopine dehydrogenase (NADP+, L-lysine-forming) activity](http://amigo.geneontology.org/cgi-bin/amigo/go.cgi?view=details&query=GO:0047130)  [saccharopine dehydrogenase (NAD+, L-glutamate-forming) activity](http://amigo.geneontology.org/cgi-bin/amigo/go.cgi?view=details&query=GO:0047131)  [saccharopine dehydrogenase (NAD+, L-glutamate-forming) activity](http://amigo.geneontology.org/cgi-bin/amigo/go.cgi?view=details&query=GO:0047131)  [protein tetramerization](http://amigo.geneontology.org/cgi-bin/amigo/go.cgi?view=details&query=GO:0051262)  [oxidation-reduction process](http://amigo.geneontology.org/cgi-bin/amigo/go.cgi?view=details&query=GO:0055114) | [Lysine biosynthesis](http://www.genome.ad.jp/dbget-bin/show_pathway?MAP00300+1.5.1.8)  [Lysine degradation](http://www.genome.ad.jp/dbget-bin/show_pathway?MAP00310+1.5.1.8)  [Metabolic pathways](http://www.genome.ad.jp/dbget-bin/show_pathway?MAP01100+1.5.1.8) |
| [216191_s_at](https://www.affymetrix.com/LinkServlet?&probeset=216191_s_at) |  |  |  |  | [X72501](http://www.ncbi.nlm.nih.gov/entrez/query.fcgi?cmd=search&db=nucleotide&term=X72501%5BACCN%5D&doptcmdl=GenBank) |  |  |  |  |  |  |
| [216620_s_at](https://www.affymetrix.com/LinkServlet?&probeset=216620_s_at) | ARHGEF10 | Rho guanine nucleotide exchange factor (GEF) 10 | 8 | 1772148 | [AF009205](http://www.ncbi.nlm.nih.gov/entrez/query.fcgi?cmd=search&db=nucleotide&term=AF009205%5BACCN%5D&doptcmdl=GenBank) | [9639](http://www.ncbi.nlm.nih.gov/sites/entrez?Db=gene&Cmd=DetailsSearch&Term=9639) | [8p23](http://www.ncbi.nlm.nih.gov/mapview/map_search.cgi?direct=on&idtype=gene&id=9639) | [Hs.98594](http://www.ncbi.nlm.nih.gov/UniGene/clust.cgi?ORG=Hs&CID=98594) | [13](http://www.ncbi.nih.gov/entrez/query.fcgi?tool=bioconductor&cmd=Retrieve&db=PubMed&list_uids=9205841%2C9314494%2C12168954%2C12477932%2C14508709%2C15146197%2C15489334%2C16896804%2C17893707%2C19615732%2C19635168%2C20042462%2C20379614) | [guanyl-nucleotide exchange factor activity](http://amigo.geneontology.org/cgi-bin/amigo/go.cgi?view=details&query=GO:0005085)  [Rho guanyl-nucleotide exchange factor activity](http://amigo.geneontology.org/cgi-bin/amigo/go.cgi?view=details&query=GO:0005089)  [intracellular](http://amigo.geneontology.org/cgi-bin/amigo/go.cgi?view=details&query=GO:0005622)  [regulation of Rho protein signal transduction](http://amigo.geneontology.org/cgi-bin/amigo/go.cgi?view=details&query=GO:0035023) |  |
| [216733_s_at](https://www.affymetrix.com/LinkServlet?&probeset=216733_s_at) | GATM | glycine amidinotransferase (L-arginine:glycine amidinotransferase) | 15 | -45653323 | [X86401](http://www.ncbi.nlm.nih.gov/entrez/query.fcgi?cmd=search&db=nucleotide&term=X86401%5BACCN%5D&doptcmdl=GenBank) | [2628](http://www.ncbi.nlm.nih.gov/sites/entrez?Db=gene&Cmd=DetailsSearch&Term=2628) | [15q21.1](http://www.ncbi.nlm.nih.gov/mapview/map_search.cgi?direct=on&idtype=gene&id=2628) | [Hs.75335](http://www.ncbi.nlm.nih.gov/UniGene/clust.cgi?ORG=Hs&CID=75335) | [27](http://www.ncbi.nih.gov/entrez/query.fcgi?tool=bioconductor&cmd=Retrieve&db=PubMed&list_uids=3800397%2C8125298%2C8313955%2C9148748%2C9165070%2C9218780%2C9266688%2C9373149%2C9915841%2C10893433%2C11555793%2C12324495%2C12468279%2C12477932%2C12701824%2C12709373%2C14702039%2C15489334%2C15978539%2C16614068%2C16769397%2C16820567%2C17101918%2C19430482%2C20383146%2C20682460%2C20877624) | [protein binding](http://amigo.geneontology.org/cgi-bin/amigo/go.cgi?view=details&query=GO:0005515)  [cytoplasm](http://amigo.geneontology.org/cgi-bin/amigo/go.cgi?view=details&query=GO:0005737)  [mitochondrion](http://amigo.geneontology.org/cgi-bin/amigo/go.cgi?view=details&query=GO:0005739)  [mitochondrial inner membrane](http://amigo.geneontology.org/cgi-bin/amigo/go.cgi?view=details&query=GO:0005743)  [mitochondrial intermembrane space](http://amigo.geneontology.org/cgi-bin/amigo/go.cgi?view=details&query=GO:0005758)  [mitochondrial intermembrane space](http://amigo.geneontology.org/cgi-bin/amigo/go.cgi?view=details&query=GO:0005758)  [creatine metabolic process](http://amigo.geneontology.org/cgi-bin/amigo/go.cgi?view=details&query=GO:0006600)  [creatine biosynthetic process](http://amigo.geneontology.org/cgi-bin/amigo/go.cgi?view=details&query=GO:0006601)  [glycine amidinotransferase activity](http://amigo.geneontology.org/cgi-bin/amigo/go.cgi?view=details&query=GO:0015068)  [glycine amidinotransferase activity](http://amigo.geneontology.org/cgi-bin/amigo/go.cgi?view=details&query=GO:0015068)  [membrane](http://amigo.geneontology.org/cgi-bin/amigo/go.cgi?view=details&query=GO:0016020)  [transferase activity](http://amigo.geneontology.org/cgi-bin/amigo/go.cgi?view=details&query=GO:0016740)  [cellular nitrogen compound metabolic process](http://amigo.geneontology.org/cgi-bin/amigo/go.cgi?view=details&query=GO:0034641) | [Glycine, serine and threonine metabolism](http://www.genome.ad.jp/dbget-bin/show_pathway?MAP00260+2.1.4.1)  [Arginine and proline metabolism](http://www.genome.ad.jp/dbget-bin/show_pathway?MAP00330+2.1.4.1)  [Metabolic pathways](http://www.genome.ad.jp/dbget-bin/show_pathway?MAP01100+2.1.4.1) |
| [218100_s_at](https://www.affymetrix.com/LinkServlet?&probeset=218100_s_at) | IFT57 | intraflagellar transport 57 homolog (Chlamydomonas) | 3 | -107879659 | [NM_018010](http://www.ncbi.nlm.nih.gov/entrez/query.fcgi?cmd=search&db=nucleotide&term=NM_018010%5BACCN%5D&doptcmdl=GenBank) | [55081](http://www.ncbi.nlm.nih.gov/sites/entrez?Db=gene&Cmd=DetailsSearch&Term=55081) | [3q13.13](http://www.ncbi.nlm.nih.gov/mapview/map_search.cgi?direct=on&idtype=gene&id=55081) | [Hs.412196](http://www.ncbi.nlm.nih.gov/UniGene/clust.cgi?ORG=Hs&CID=412196) | [14](http://www.ncbi.nih.gov/entrez/query.fcgi?tool=bioconductor&cmd=Retrieve&db=PubMed&list_uids=9140394%2C11788820%2C11807533%2C12477932%2C12745083%2C12821668%2C14702039%2C15314642%2C16169070%2C16344560%2C16364650%2C17142908%2C17173859%2C18188704) | [DNA binding](http://amigo.geneontology.org/cgi-bin/amigo/go.cgi?view=details&query=GO:0003677)  [protein binding](http://amigo.geneontology.org/cgi-bin/amigo/go.cgi?view=details&query=GO:0005515)  [cytoplasm](http://amigo.geneontology.org/cgi-bin/amigo/go.cgi?view=details&query=GO:0005737)  [Golgi apparatus](http://amigo.geneontology.org/cgi-bin/amigo/go.cgi?view=details&query=GO:0005794)  [cytoskeleton](http://amigo.geneontology.org/cgi-bin/amigo/go.cgi?view=details&query=GO:0005856)  [cilium](http://amigo.geneontology.org/cgi-bin/amigo/go.cgi?view=details&query=GO:0005929)  [microtubule basal body](http://amigo.geneontology.org/cgi-bin/amigo/go.cgi?view=details&query=GO:0005932)  [apoptosis](http://amigo.geneontology.org/cgi-bin/amigo/go.cgi?view=details&query=GO:0006915)  [activation of caspase activity](http://amigo.geneontology.org/cgi-bin/amigo/go.cgi?view=details&query=GO:0006919)  [regulation of apoptosis](http://amigo.geneontology.org/cgi-bin/amigo/go.cgi?view=details&query=GO:0042981)  [regulation of transcription](http://amigo.geneontology.org/cgi-bin/amigo/go.cgi?view=details&query=GO:0045449) | [Huntington's disease](http://www.genome.ad.jp/kegg/pathway/hsa/hsa05016.html) |
| [218266_s_at](https://www.affymetrix.com/LinkServlet?&probeset=218266_s_at) | NCS1 | neuronal calcium sensor 1 | 9 | 132934856, 132962871 | [NM_014286](http://www.ncbi.nlm.nih.gov/entrez/query.fcgi?cmd=search&db=nucleotide&term=NM_014286%5BACCN%5D&doptcmdl=GenBank) | [23413](http://www.ncbi.nlm.nih.gov/sites/entrez?Db=gene&Cmd=DetailsSearch&Term=23413) | [9q34](http://www.ncbi.nlm.nih.gov/mapview/map_search.cgi?direct=on&idtype=gene&id=23413) | [Hs.642946](http://www.ncbi.nlm.nih.gov/UniGene/clust.cgi?ORG=Hs&CID=642946) | [31](http://www.ncbi.nih.gov/entrez/query.fcgi?tool=bioconductor&cmd=Retrieve&db=PubMed&list_uids=7488079%2C8889548%2C11092894%2C11115393%2C11526106%2C11606724%2C11825672%2C11836243%2C12006624%2C12244129%2C12351722%2C12477932%2C12496348%2C12783849%2C12928444%2C14512421%2C14607934%2C14702039%2C14726528%2C14760944%2C15164053%2C15489334%2C16189514%2C16344560%2C16402081%2C16837555%2C17672918%2C18029348%2C19091302%2C20468064%2C20479890) | [calcium ion binding](http://amigo.geneontology.org/cgi-bin/amigo/go.cgi?view=details&query=GO:0005509)  [protein binding](http://amigo.geneontology.org/cgi-bin/amigo/go.cgi?view=details&query=GO:0005515)  [cytoplasm](http://amigo.geneontology.org/cgi-bin/amigo/go.cgi?view=details&query=GO:0005737)  [Golgi apparatus](http://amigo.geneontology.org/cgi-bin/amigo/go.cgi?view=details&query=GO:0005794)  [plasma membrane](http://amigo.geneontology.org/cgi-bin/amigo/go.cgi?view=details&query=GO:0005886)  [regulation of neuron projection development](http://amigo.geneontology.org/cgi-bin/amigo/go.cgi?view=details&query=GO:0010975)  [postsynaptic density](http://amigo.geneontology.org/cgi-bin/amigo/go.cgi?view=details&query=GO:0014069)  [cell junction](http://amigo.geneontology.org/cgi-bin/amigo/go.cgi?view=details&query=GO:0030054)  [Golgi cisterna membrane](http://amigo.geneontology.org/cgi-bin/amigo/go.cgi?view=details&query=GO:0032580)  [intracellular membrane-bounded organelle](http://amigo.geneontology.org/cgi-bin/amigo/go.cgi?view=details&query=GO:0043231)  [synapse](http://amigo.geneontology.org/cgi-bin/amigo/go.cgi?view=details&query=GO:0045202)  [postsynaptic membrane](http://amigo.geneontology.org/cgi-bin/amigo/go.cgi?view=details&query=GO:0045211)  [perinuclear region of cytoplasm](http://amigo.geneontology.org/cgi-bin/amigo/go.cgi?view=details&query=GO:0048471)  [negative regulation of calcium ion transport via voltage-gated calcium channel activity](http://amigo.geneontology.org/cgi-bin/amigo/go.cgi?view=details&query=GO:0051927) |  |
| [218613_at](https://www.affymetrix.com/LinkServlet?&probeset=218613_at) | PSD3 | pleckstrin and Sec7 domain containing 3 | 8 | -18384813, -18384813 | [NM_018422](http://www.ncbi.nlm.nih.gov/entrez/query.fcgi?cmd=search&db=nucleotide&term=NM_018422%5BACCN%5D&doptcmdl=GenBank) | [23362](http://www.ncbi.nlm.nih.gov/sites/entrez?Db=gene&Cmd=DetailsSearch&Term=23362) | [8p21.3](http://www.ncbi.nlm.nih.gov/mapview/map_search.cgi?direct=on&idtype=gene&id=23362) | [Hs.434255](http://www.ncbi.nlm.nih.gov/UniGene/clust.cgi?ORG=Hs&CID=434255) | [13](http://www.ncbi.nih.gov/entrez/query.fcgi?tool=bioconductor&cmd=Retrieve&db=PubMed&list_uids=10231032%2C12097419%2C12168954%2C12477932%2C14702039%2C15489334%2C16270321%2C16344560%2C17148452%2C18649358%2C20080650%2C20379614%2C20736409) | [ARF guanyl-nucleotide exchange factor activity](http://amigo.geneontology.org/cgi-bin/amigo/go.cgi?view=details&query=GO:0005086)  [intracellular](http://amigo.geneontology.org/cgi-bin/amigo/go.cgi?view=details&query=GO:0005622)  [cytoplasm](http://amigo.geneontology.org/cgi-bin/amigo/go.cgi?view=details&query=GO:0005737)  [plasma membrane](http://amigo.geneontology.org/cgi-bin/amigo/go.cgi?view=details&query=GO:0005886)  [postsynaptic density](http://amigo.geneontology.org/cgi-bin/amigo/go.cgi?view=details&query=GO:0014069)  [cell junction](http://amigo.geneontology.org/cgi-bin/amigo/go.cgi?view=details&query=GO:0030054)  [regulation of ARF protein signal transduction](http://amigo.geneontology.org/cgi-bin/amigo/go.cgi?view=details&query=GO:0032012)  [synapse](http://amigo.geneontology.org/cgi-bin/amigo/go.cgi?view=details&query=GO:0045202)  [postsynaptic membrane](http://amigo.geneontology.org/cgi-bin/amigo/go.cgi?view=details&query=GO:0045211) | [Endocytosis](http://www.genome.ad.jp/kegg/pathway/hsa/hsa04144.html) |
| [218651_s_at](https://www.affymetrix.com/LinkServlet?&probeset=218651_s_at) | LARP6 | La ribonucleoprotein domain family, member 6 | 15 | -71143769, -71123890 | [NM_018357](http://www.ncbi.nlm.nih.gov/entrez/query.fcgi?cmd=search&db=nucleotide&term=NM_018357%5BACCN%5D&doptcmdl=GenBank) | [55323](http://www.ncbi.nlm.nih.gov/sites/entrez?Db=gene&Cmd=DetailsSearch&Term=55323) | [15q23](http://www.ncbi.nlm.nih.gov/mapview/map_search.cgi?direct=on&idtype=gene&id=55323) | [Hs.416755](http://www.ncbi.nlm.nih.gov/UniGene/clust.cgi?ORG=Hs&CID=416755) | [8](http://www.ncbi.nih.gov/entrez/query.fcgi?tool=bioconductor&cmd=Retrieve&db=PubMed&list_uids=12477932%2C14702039%2C15489334%2C17081983%2C17383118%2C18029348%2C19889961%2C19917293) | [nucleotide binding](http://amigo.geneontology.org/cgi-bin/amigo/go.cgi?view=details&query=GO:0000166)  [RNA binding](http://amigo.geneontology.org/cgi-bin/amigo/go.cgi?view=details&query=GO:0003723)  [nucleus](http://amigo.geneontology.org/cgi-bin/amigo/go.cgi?view=details&query=GO:0005634)  [cytoplasm](http://amigo.geneontology.org/cgi-bin/amigo/go.cgi?view=details&query=GO:0005737)  [Golgi apparatus](http://amigo.geneontology.org/cgi-bin/amigo/go.cgi?view=details&query=GO:0005794)  [RNA processing](http://amigo.geneontology.org/cgi-bin/amigo/go.cgi?view=details&query=GO:0006396)  [ribonucleoprotein complex](http://amigo.geneontology.org/cgi-bin/amigo/go.cgi?view=details&query=GO:0030529)  [intracellular membrane-bounded organelle](http://amigo.geneontology.org/cgi-bin/amigo/go.cgi?view=details&query=GO:0043231) |  |
| [218793_s_at](https://www.affymetrix.com/LinkServlet?&probeset=218793_s_at) | SCML1 | sex comb on midleg-like 1 (Drosophila) | X | 17755591 | [NM_006746](http://www.ncbi.nlm.nih.gov/entrez/query.fcgi?cmd=search&db=nucleotide&term=NM_006746%5BACCN%5D&doptcmdl=GenBank) | [6322](http://www.ncbi.nlm.nih.gov/sites/entrez?Db=gene&Cmd=DetailsSearch&Term=6322) | [Xp22](http://www.ncbi.nlm.nih.gov/mapview/map_search.cgi?direct=on&idtype=gene&id=6322) | [Hs.109655](http://www.ncbi.nlm.nih.gov/UniGene/clust.cgi?ORG=Hs&CID=109655) | [5](http://www.ncbi.nih.gov/entrez/query.fcgi?tool=bioconductor&cmd=Retrieve&db=PubMed&list_uids=9570953%2C12477932%2C15489334%2C15772651%2C18601738) | [DNA binding](http://amigo.geneontology.org/cgi-bin/amigo/go.cgi?view=details&query=GO:0003677)  [sequence-specific DNA binding transcription factor activity](http://amigo.geneontology.org/cgi-bin/amigo/go.cgi?view=details&query=GO:0003700)  [nucleus](http://amigo.geneontology.org/cgi-bin/amigo/go.cgi?view=details&query=GO:0005634)  [transcription, DNA-dependent](http://amigo.geneontology.org/cgi-bin/amigo/go.cgi?view=details&query=GO:0006351)  [anatomical structure morphogenesis](http://amigo.geneontology.org/cgi-bin/amigo/go.cgi?view=details&query=GO:0009653)  [regulation of transcription](http://amigo.geneontology.org/cgi-bin/amigo/go.cgi?view=details&query=GO:0045449) |  |
| [218820_at](https://www.affymetrix.com/LinkServlet?&probeset=218820_at) | C14orf132 | chromosome 14 open reading frame 132 | 14 | 96505661 | [NM_020215](http://www.ncbi.nlm.nih.gov/entrez/query.fcgi?cmd=search&db=nucleotide&term=NM_020215%5BACCN%5D&doptcmdl=GenBank) | [56967](http://www.ncbi.nlm.nih.gov/sites/entrez?Db=gene&Cmd=DetailsSearch&Term=56967) | [14q32.2](http://www.ncbi.nlm.nih.gov/mapview/map_search.cgi?direct=on&idtype=gene&id=56967) | [Hs.6434](http://www.ncbi.nlm.nih.gov/UniGene/clust.cgi?ORG=Hs&CID=6434) | [6](http://www.ncbi.nih.gov/entrez/query.fcgi?tool=bioconductor&cmd=Retrieve&db=PubMed&list_uids=8619474%2C9110174%2C12477932%2C14702039%2C15489334%2C16344560) | [membrane](http://amigo.geneontology.org/cgi-bin/amigo/go.cgi?view=details&query=GO:0016020)  [integral to membrane](http://amigo.geneontology.org/cgi-bin/amigo/go.cgi?view=details&query=GO:0016021) |  |
| [218919_at](https://www.affymetrix.com/LinkServlet?&probeset=218919_at) | ZFAND1 | zinc finger, AN1-type domain 1 | 8 | -82615149, -82613567 | [NM_024699](http://www.ncbi.nlm.nih.gov/entrez/query.fcgi?cmd=search&db=nucleotide&term=NM_024699%5BACCN%5D&doptcmdl=GenBank) | [79752](http://www.ncbi.nlm.nih.gov/sites/entrez?Db=gene&Cmd=DetailsSearch&Term=79752) | [8q21.13](http://www.ncbi.nlm.nih.gov/mapview/map_search.cgi?direct=on&idtype=gene&id=79752) | [Hs.655453](http://www.ncbi.nlm.nih.gov/UniGene/clust.cgi?ORG=Hs&CID=655453) | [7](http://www.ncbi.nih.gov/entrez/query.fcgi?tool=bioconductor&cmd=Retrieve&db=PubMed&list_uids=8125298%2C8889548%2C9373149%2C12477932%2C14702039%2C15489334%2C17207965) | [zinc ion binding](http://amigo.geneontology.org/cgi-bin/amigo/go.cgi?view=details&query=GO:0008270)  [metal ion binding](http://amigo.geneontology.org/cgi-bin/amigo/go.cgi?view=details&query=GO:0046872) |  |
| [219013_at](https://www.affymetrix.com/LinkServlet?&probeset=219013_at) | GALNT11 | UDP-N-acetyl-alpha-D-galactosamine:polypeptide N-acetylgalactosaminyltransferase 11 (GalNAc-T11) | 7 | 151722777 | [NM_022087](http://www.ncbi.nlm.nih.gov/entrez/query.fcgi?cmd=search&db=nucleotide&term=NM_022087%5BACCN%5D&doptcmdl=GenBank) | [63917](http://www.ncbi.nlm.nih.gov/sites/entrez?Db=gene&Cmd=DetailsSearch&Term=63917) | [7q36.1](http://www.ncbi.nlm.nih.gov/mapview/map_search.cgi?direct=on&idtype=gene&id=63917) | [Hs.647109](http://www.ncbi.nlm.nih.gov/UniGene/clust.cgi?ORG=Hs&CID=647109) | [11](http://www.ncbi.nih.gov/entrez/query.fcgi?tool=bioconductor&cmd=Retrieve&db=PubMed&list_uids=9847074%2C11925450%2C12477932%2C12690205%2C12853948%2C14702039%2C15146197%2C15489334%2C16344560%2C20422447%2C20547088) | [Golgi membrane](http://amigo.geneontology.org/cgi-bin/amigo/go.cgi?view=details&query=GO:0000139)  [polypeptide N-acetylgalactosaminyltransferase activity](http://amigo.geneontology.org/cgi-bin/amigo/go.cgi?view=details&query=GO:0004653)  [sugar binding](http://amigo.geneontology.org/cgi-bin/amigo/go.cgi?view=details&query=GO:0005529)  [Golgi apparatus](http://amigo.geneontology.org/cgi-bin/amigo/go.cgi?view=details&query=GO:0005794)  [membrane](http://amigo.geneontology.org/cgi-bin/amigo/go.cgi?view=details&query=GO:0016020)  [integral to membrane](http://amigo.geneontology.org/cgi-bin/amigo/go.cgi?view=details&query=GO:0016021)  [transferase activity, transferring glycosyl groups](http://amigo.geneontology.org/cgi-bin/amigo/go.cgi?view=details&query=GO:0016757) | [Mucin type O-Glycan biosynthesis](http://www.genome.ad.jp/dbget-bin/show_pathway?MAP00512+2.4.1.41)  [Metabolic pathways](http://www.genome.ad.jp/dbget-bin/show_pathway?MAP01100+2.4.1.41) |
| [219143_s_at](https://www.affymetrix.com/LinkServlet?&probeset=219143_s_at) | RPP25 | ribonuclease P/MRP 25kDa subunit | 15 | -75247442 | [NM_017793](http://www.ncbi.nlm.nih.gov/entrez/query.fcgi?cmd=search&db=nucleotide&term=NM_017793%5BACCN%5D&doptcmdl=GenBank) | [54913](http://www.ncbi.nlm.nih.gov/sites/entrez?Db=gene&Cmd=DetailsSearch&Term=54913) | [15q24.2](http://www.ncbi.nlm.nih.gov/mapview/map_search.cgi?direct=on&idtype=gene&id=54913) | [Hs.8562](http://www.ncbi.nlm.nih.gov/UniGene/clust.cgi?ORG=Hs&CID=8562) | [9](http://www.ncbi.nih.gov/entrez/query.fcgi?tool=bioconductor&cmd=Retrieve&db=PubMed&list_uids=12003489%2C12477932%2C14702039%2C15096576%2C15489334%2C16344560%2C17207965%2C20215441%2C20632321) | [RNA binding](http://amigo.geneontology.org/cgi-bin/amigo/go.cgi?view=details&query=GO:0003723)  [ribonuclease P activity](http://amigo.geneontology.org/cgi-bin/amigo/go.cgi?view=details&query=GO:0004526)  [protein binding](http://amigo.geneontology.org/cgi-bin/amigo/go.cgi?view=details&query=GO:0005515)  [nucleus](http://amigo.geneontology.org/cgi-bin/amigo/go.cgi?view=details&query=GO:0005634)  [tRNA processing](http://amigo.geneontology.org/cgi-bin/amigo/go.cgi?view=details&query=GO:0008033)  [hydrolase activity](http://amigo.geneontology.org/cgi-bin/amigo/go.cgi?view=details&query=GO:0016787) | [Ribosome biogenesis in eukaryotes](http://www.genome.ad.jp/dbget-bin/show_pathway?MAP03008+3.1.26.5)  [RNA transport](http://www.genome.ad.jp/dbget-bin/show_pathway?MAP03013+3.1.26.5) |
| [219355_at](https://www.affymetrix.com/LinkServlet?&probeset=219355_at) | CXorf57 | chromosome X open reading frame 57 | X | 105855159 | [NM_018015](http://www.ncbi.nlm.nih.gov/entrez/query.fcgi?cmd=search&db=nucleotide&term=NM_018015%5BACCN%5D&doptcmdl=GenBank) | [55086](http://www.ncbi.nlm.nih.gov/sites/entrez?Db=gene&Cmd=DetailsSearch&Term=55086) | [Xq22.3](http://www.ncbi.nlm.nih.gov/mapview/map_search.cgi?direct=on&idtype=gene&id=55086) | [Hs.274267](http://www.ncbi.nlm.nih.gov/UniGene/clust.cgi?ORG=Hs&CID=274267) | [5](http://www.ncbi.nih.gov/entrez/query.fcgi?tool=bioconductor&cmd=Retrieve&db=PubMed&list_uids=12477932%2C14702039%2C15489334%2C15772651%2C16344560) |  |  |
| [219469_at](https://www.affymetrix.com/LinkServlet?&probeset=219469_at) | DYNC2H1 | dynein, cytoplasmic 2, heavy chain 1 | 11 | 102980159 | [NM_024606](http://www.ncbi.nlm.nih.gov/entrez/query.fcgi?cmd=search&db=nucleotide&term=NM_024606%5BACCN%5D&doptcmdl=GenBank) | [79659](http://www.ncbi.nlm.nih.gov/sites/entrez?Db=gene&Cmd=DetailsSearch&Term=79659) | [11q21-q22.1](http://www.ncbi.nlm.nih.gov/mapview/map_search.cgi?direct=on&idtype=gene&id=79659) | [Hs.503721](http://www.ncbi.nlm.nih.gov/UniGene/clust.cgi?ORG=Hs&CID=503721) | [18](http://www.ncbi.nih.gov/entrez/query.fcgi?tool=bioconductor&cmd=Retrieve&db=PubMed&list_uids=8186465%2C8666668%2C8832411%2C9325061%2C9373155%2C9763680%2C11907264%2C12056414%2C12432068%2C12477932%2C14702039%2C16320026%2C16440056%2C19361615%2C19442771%2C19615732%2C20379614%2C21118971) | [nucleotide binding](http://amigo.geneontology.org/cgi-bin/amigo/go.cgi?view=details&query=GO:0000166)  [motor activity](http://amigo.geneontology.org/cgi-bin/amigo/go.cgi?view=details&query=GO:0003774)  [microtubule motor activity](http://amigo.geneontology.org/cgi-bin/amigo/go.cgi?view=details&query=GO:0003777)  [ATP binding](http://amigo.geneontology.org/cgi-bin/amigo/go.cgi?view=details&query=GO:0005524)  [cytoplasm](http://amigo.geneontology.org/cgi-bin/amigo/go.cgi?view=details&query=GO:0005737)  [Golgi apparatus](http://amigo.geneontology.org/cgi-bin/amigo/go.cgi?view=details&query=GO:0005794)  [cytoskeleton](http://amigo.geneontology.org/cgi-bin/amigo/go.cgi?view=details&query=GO:0005856)  [microtubule](http://amigo.geneontology.org/cgi-bin/amigo/go.cgi?view=details&query=GO:0005874)  [plasma membrane](http://amigo.geneontology.org/cgi-bin/amigo/go.cgi?view=details&query=GO:0005886)  [cilium](http://amigo.geneontology.org/cgi-bin/amigo/go.cgi?view=details&query=GO:0005929)  [microtubule-based movement](http://amigo.geneontology.org/cgi-bin/amigo/go.cgi?view=details&query=GO:0007018)  [Golgi organization](http://amigo.geneontology.org/cgi-bin/amigo/go.cgi?view=details&query=GO:0007030)  [multicellular organismal development](http://amigo.geneontology.org/cgi-bin/amigo/go.cgi?view=details&query=GO:0007275)  [ATPase activity](http://amigo.geneontology.org/cgi-bin/amigo/go.cgi?view=details&query=GO:0016887)  [cell projection organization](http://amigo.geneontology.org/cgi-bin/amigo/go.cgi?view=details&query=GO:0030030)  [dynein complex](http://amigo.geneontology.org/cgi-bin/amigo/go.cgi?view=details&query=GO:0030286)  [cilium axoneme](http://amigo.geneontology.org/cgi-bin/amigo/go.cgi?view=details&query=GO:0035085) | [Phagosome](http://www.genome.ad.jp/kegg/pathway/hsa/hsa04145.html)  [Vasopressin-regulated water reabsorption](http://www.genome.ad.jp/kegg/pathway/hsa/hsa04962.html) |
| [219570_at](https://www.affymetrix.com/LinkServlet?&probeset=219570_at) | KIF16B | kinesin family member 16B | 20 | -16252748 | [NM_024704](http://www.ncbi.nlm.nih.gov/entrez/query.fcgi?cmd=search&db=nucleotide&term=NM_024704%5BACCN%5D&doptcmdl=GenBank) | [55614](http://www.ncbi.nlm.nih.gov/sites/entrez?Db=gene&Cmd=DetailsSearch&Term=55614) | [20p11.23](http://www.ncbi.nlm.nih.gov/mapview/map_search.cgi?direct=on&idtype=gene&id=55614) | [Hs.101774](http://www.ncbi.nlm.nih.gov/UniGene/clust.cgi?ORG=Hs&CID=101774) | [11](http://www.ncbi.nih.gov/entrez/query.fcgi?tool=bioconductor&cmd=Retrieve&db=PubMed&list_uids=10997877%2C11780052%2C12461558%2C12477932%2C14702039%2C15489334%2C15882625%2C16084724%2C16782399%2C17203973%2C21139019) | [nucleotide binding](http://amigo.geneontology.org/cgi-bin/amigo/go.cgi?view=details&query=GO:0000166)  [microtubule motor activity](http://amigo.geneontology.org/cgi-bin/amigo/go.cgi?view=details&query=GO:0003777)  [ATP binding](http://amigo.geneontology.org/cgi-bin/amigo/go.cgi?view=details&query=GO:0005524)  [cytoplasm](http://amigo.geneontology.org/cgi-bin/amigo/go.cgi?view=details&query=GO:0005737)  [cytoskeleton](http://amigo.geneontology.org/cgi-bin/amigo/go.cgi?view=details&query=GO:0005856)  [microtubule](http://amigo.geneontology.org/cgi-bin/amigo/go.cgi?view=details&query=GO:0005874)  [microtubule-based movement](http://amigo.geneontology.org/cgi-bin/amigo/go.cgi?view=details&query=GO:0007018)  [cell communication](http://amigo.geneontology.org/cgi-bin/amigo/go.cgi?view=details&query=GO:0007154)  [phosphatidylinositol binding](http://amigo.geneontology.org/cgi-bin/amigo/go.cgi?view=details&query=GO:0035091) |  |
| [219737_s_at](https://www.affymetrix.com/LinkServlet?&probeset=219737_s_at) | PCDH9 | protocadherin 9 | 13 | -66876966 | [AI524125](http://www.ncbi.nlm.nih.gov/entrez/query.fcgi?cmd=search&db=nucleotide&term=AI524125%5BACCN%5D&doptcmdl=GenBank) | [5101](http://www.ncbi.nlm.nih.gov/sites/entrez?Db=gene&Cmd=DetailsSearch&Term=5101) | [13q21.32](http://www.ncbi.nlm.nih.gov/mapview/map_search.cgi?direct=on&idtype=gene&id=5101) | [Hs.654709](http://www.ncbi.nlm.nih.gov/UniGene/clust.cgi?ORG=Hs&CID=654709) | [11](http://www.ncbi.nih.gov/entrez/query.fcgi?tool=bioconductor&cmd=Retrieve&db=PubMed&list_uids=9787079%2C10716726%2C10817752%2C10835267%2C12477932%2C14702039%2C15057823%2C16344560%2C19913121%2C20379614%2C20628086) | [calcium ion binding](http://amigo.geneontology.org/cgi-bin/amigo/go.cgi?view=details&query=GO:0005509)  [plasma membrane](http://amigo.geneontology.org/cgi-bin/amigo/go.cgi?view=details&query=GO:0005886)  [cell adhesion](http://amigo.geneontology.org/cgi-bin/amigo/go.cgi?view=details&query=GO:0007155)  [homophilic cell adhesion](http://amigo.geneontology.org/cgi-bin/amigo/go.cgi?view=details&query=GO:0007156)  [integral to membrane](http://amigo.geneontology.org/cgi-bin/amigo/go.cgi?view=details&query=GO:0016021) |  |
| [219938_s_at](https://www.affymetrix.com/LinkServlet?&probeset=219938_s_at) | PSTPIP2 | proline-serine-threonine phosphatase interacting protein 2 | 18 | -43563502 | [NM_024430](http://www.ncbi.nlm.nih.gov/entrez/query.fcgi?cmd=search&db=nucleotide&term=NM_024430%5BACCN%5D&doptcmdl=GenBank) | [9050](http://www.ncbi.nlm.nih.gov/sites/entrez?Db=gene&Cmd=DetailsSearch&Term=9050) | [18q12](http://www.ncbi.nlm.nih.gov/mapview/map_search.cgi?direct=on&idtype=gene&id=9050) | [Hs.567384](http://www.ncbi.nlm.nih.gov/UniGene/clust.cgi?ORG=Hs&CID=567384) | [10](http://www.ncbi.nih.gov/entrez/query.fcgi?tool=bioconductor&cmd=Retrieve&db=PubMed&list_uids=8889549%2C9804817%2C9804836%2C12477932%2C14702039%2C15342556%2C15489334%2C15862967%2C16344560%2C20032092) | [cytoplasm](http://amigo.geneontology.org/cgi-bin/amigo/go.cgi?view=details&query=GO:0005737)  [membrane](http://amigo.geneontology.org/cgi-bin/amigo/go.cgi?view=details&query=GO:0016020) |  |
| [219945_at](https://www.affymetrix.com/LinkServlet?&probeset=219945_at) | DDX25 | DEAD (Asp-Glu-Ala-Asp) box polypeptide 25 | 11 | 125774271 | [NM_013264](http://www.ncbi.nlm.nih.gov/entrez/query.fcgi?cmd=search&db=nucleotide&term=NM_013264%5BACCN%5D&doptcmdl=GenBank) | [29118](http://www.ncbi.nlm.nih.gov/sites/entrez?Db=gene&Cmd=DetailsSearch&Term=29118) | [11q24](http://www.ncbi.nlm.nih.gov/mapview/map_search.cgi?direct=on&idtype=gene&id=29118) | [Hs.420263](http://www.ncbi.nlm.nih.gov/UniGene/clust.cgi?ORG=Hs&CID=420263) | [10](http://www.ncbi.nih.gov/entrez/query.fcgi?tool=bioconductor&cmd=Retrieve&db=PubMed&list_uids=10608860%2C12477932%2C15094194%2C15489334%2C16293649%2C17848414%2C17889551%2C19536092%2C19875492%2C20378615) | [nucleotide binding](http://amigo.geneontology.org/cgi-bin/amigo/go.cgi?view=details&query=GO:0000166)  [RNA binding](http://amigo.geneontology.org/cgi-bin/amigo/go.cgi?view=details&query=GO:0003723)  [ATP-dependent RNA helicase activity](http://amigo.geneontology.org/cgi-bin/amigo/go.cgi?view=details&query=GO:0004004)  [ATP binding](http://amigo.geneontology.org/cgi-bin/amigo/go.cgi?view=details&query=GO:0005524)  [nucleus](http://amigo.geneontology.org/cgi-bin/amigo/go.cgi?view=details&query=GO:0005634)  [cytoplasm](http://amigo.geneontology.org/cgi-bin/amigo/go.cgi?view=details&query=GO:0005737)  [mRNA export from nucleus](http://amigo.geneontology.org/cgi-bin/amigo/go.cgi?view=details&query=GO:0006406)  [regulation of translation](http://amigo.geneontology.org/cgi-bin/amigo/go.cgi?view=details&query=GO:0006417)  [transport](http://amigo.geneontology.org/cgi-bin/amigo/go.cgi?view=details&query=GO:0006810)  [multicellular organismal development](http://amigo.geneontology.org/cgi-bin/amigo/go.cgi?view=details&query=GO:0007275)  [spermatogenesis](http://amigo.geneontology.org/cgi-bin/amigo/go.cgi?view=details&query=GO:0007283)  [spermatid development](http://amigo.geneontology.org/cgi-bin/amigo/go.cgi?view=details&query=GO:0007286)  [hydrolase activity](http://amigo.geneontology.org/cgi-bin/amigo/go.cgi?view=details&query=GO:0016787)  [ATPase activity](http://amigo.geneontology.org/cgi-bin/amigo/go.cgi?view=details&query=GO:0016887)  [cell differentiation](http://amigo.geneontology.org/cgi-bin/amigo/go.cgi?view=details&query=GO:0030154)  [chromatoid body](http://amigo.geneontology.org/cgi-bin/amigo/go.cgi?view=details&query=GO:0033391) |  |
| [219983_at](https://www.affymetrix.com/LinkServlet?&probeset=219983_at) | HRASLS | HRAS-like suppressor | 3 | 192958917 | [NM_020386](http://www.ncbi.nlm.nih.gov/entrez/query.fcgi?cmd=search&db=nucleotide&term=NM_020386%5BACCN%5D&doptcmdl=GenBank) | [57110](http://www.ncbi.nlm.nih.gov/sites/entrez?Db=gene&Cmd=DetailsSearch&Term=57110) | [3q29](http://www.ncbi.nlm.nih.gov/mapview/map_search.cgi?direct=on&idtype=gene&id=57110) | [Hs.36761](http://www.ncbi.nlm.nih.gov/UniGene/clust.cgi?ORG=Hs&CID=36761) | [5](http://www.ncbi.nih.gov/entrez/query.fcgi?tool=bioconductor&cmd=Retrieve&db=PubMed&list_uids=9771974%2C10542256%2C11474175%2C12477932%2C20837014) | [nuclear envelope lumen](http://amigo.geneontology.org/cgi-bin/amigo/go.cgi?view=details&query=GO:0005641)  [cytoplasm](http://amigo.geneontology.org/cgi-bin/amigo/go.cgi?view=details&query=GO:0005737) |  |
| [219984_s_at](https://www.affymetrix.com/LinkServlet?&probeset=219984_s_at) | HRASLS | HRAS-like suppressor | 3 | 192958917 | [NM_020386](http://www.ncbi.nlm.nih.gov/entrez/query.fcgi?cmd=search&db=nucleotide&term=NM_020386%5BACCN%5D&doptcmdl=GenBank) | [57110](http://www.ncbi.nlm.nih.gov/sites/entrez?Db=gene&Cmd=DetailsSearch&Term=57110) | [3q29](http://www.ncbi.nlm.nih.gov/mapview/map_search.cgi?direct=on&idtype=gene&id=57110) | [Hs.36761](http://www.ncbi.nlm.nih.gov/UniGene/clust.cgi?ORG=Hs&CID=36761) | [5](http://www.ncbi.nih.gov/entrez/query.fcgi?tool=bioconductor&cmd=Retrieve&db=PubMed&list_uids=9771974%2C10542256%2C11474175%2C12477932%2C20837014) | [nuclear envelope lumen](http://amigo.geneontology.org/cgi-bin/amigo/go.cgi?view=details&query=GO:0005641)  [cytoplasm](http://amigo.geneontology.org/cgi-bin/amigo/go.cgi?view=details&query=GO:0005737) |  |
| [220150_s_at](https://www.affymetrix.com/LinkServlet?&probeset=220150_s_at) | FAM184A | family with sequence similarity 184, member A | 6 | -119280997, -119280997 | [NM_024581](http://www.ncbi.nlm.nih.gov/entrez/query.fcgi?cmd=search&db=nucleotide&term=NM_024581%5BACCN%5D&doptcmdl=GenBank) | [79632](http://www.ncbi.nlm.nih.gov/sites/entrez?Db=gene&Cmd=DetailsSearch&Term=79632) | [6q22.31](http://www.ncbi.nlm.nih.gov/mapview/map_search.cgi?direct=on&idtype=gene&id=79632) | [Hs.443789](http://www.ncbi.nlm.nih.gov/UniGene/clust.cgi?ORG=Hs&CID=443789) | [6](http://www.ncbi.nih.gov/entrez/query.fcgi?tool=bioconductor&cmd=Retrieve&db=PubMed&list_uids=11230166%2C12477932%2C14702039%2C15489334%2C16344560%2C20379614) | [molecular_function](http://amigo.geneontology.org/cgi-bin/amigo/go.cgi?view=details&query=GO:0003674)  [cellular_component](http://amigo.geneontology.org/cgi-bin/amigo/go.cgi?view=details&query=GO:0005575)  [biological_process](http://amigo.geneontology.org/cgi-bin/amigo/go.cgi?view=details&query=GO:0008150) |  |
| [220892_s_at](https://www.affymetrix.com/LinkServlet?&probeset=220892_s_at) | PSAT1 | phosphoserine aminotransferase 1 | 9 | 80912058 | [NM_021154](http://www.ncbi.nlm.nih.gov/entrez/query.fcgi?cmd=search&db=nucleotide&term=NM_021154%5BACCN%5D&doptcmdl=GenBank) | [29968](http://www.ncbi.nlm.nih.gov/sites/entrez?Db=gene&Cmd=DetailsSearch&Term=29968) | [9q21.2](http://www.ncbi.nlm.nih.gov/mapview/map_search.cgi?direct=on&idtype=gene&id=29968) | [Hs.494261](http://www.ncbi.nlm.nih.gov/UniGene/clust.cgi?ORG=Hs&CID=494261) [Hs.592595](http://www.ncbi.nlm.nih.gov/UniGene/clust.cgi?ORG=Hs&CID=592595) | [6](http://www.ncbi.nih.gov/entrez/query.fcgi?tool=bioconductor&cmd=Retrieve&db=PubMed&list_uids=3651428%2C10637769%2C12477932%2C12633500%2C15489334%2C18221502) | [O-phospho-L-serine:2-oxoglutarate aminotransferase activity](http://amigo.geneontology.org/cgi-bin/amigo/go.cgi?view=details&query=GO:0004648)  [L-serine biosynthetic process](http://amigo.geneontology.org/cgi-bin/amigo/go.cgi?view=details&query=GO:0006564)  [pyridoxine biosynthetic process](http://amigo.geneontology.org/cgi-bin/amigo/go.cgi?view=details&query=GO:0008615)  [cellular amino acid biosynthetic process](http://amigo.geneontology.org/cgi-bin/amigo/go.cgi?view=details&query=GO:0008652)  [transferase activity](http://amigo.geneontology.org/cgi-bin/amigo/go.cgi?view=details&query=GO:0016740)  [pyridoxal phosphate binding](http://amigo.geneontology.org/cgi-bin/amigo/go.cgi?view=details&query=GO:0030170) | [Glycine, serine and threonine metabolism](http://www.genome.ad.jp/dbget-bin/show_pathway?MAP00260+2.6.1.52)  [Vitamin B6 metabolism](http://www.genome.ad.jp/dbget-bin/show_pathway?MAP00750+2.6.1.52)  [Metabolic pathways](http://www.genome.ad.jp/dbget-bin/show_pathway?MAP01100+2.6.1.52) |
| [221059_s_at](https://www.affymetrix.com/LinkServlet?&probeset=221059_s_at) |  |  |  |  | [NM_021615](http://www.ncbi.nlm.nih.gov/entrez/query.fcgi?cmd=search&db=nucleotide&term=NM_021615%5BACCN%5D&doptcmdl=GenBank) |  |  |  |  | [Golgi membrane](http://amigo.geneontology.org/cgi-bin/amigo/go.cgi?view=details&query=GO:0000139)  [N-acetylglucosamine 6-O-sulfotransferase activity](http://amigo.geneontology.org/cgi-bin/amigo/go.cgi?view=details&query=GO:0001517)  [N-acetylglucosamine 6-O-sulfotransferase activity](http://amigo.geneontology.org/cgi-bin/amigo/go.cgi?view=details&query=GO:0001517)  [actin binding](http://amigo.geneontology.org/cgi-bin/amigo/go.cgi?view=details&query=GO:0003779)  [protein binding](http://amigo.geneontology.org/cgi-bin/amigo/go.cgi?view=details&query=GO:0005515)  [cellular_component](http://amigo.geneontology.org/cgi-bin/amigo/go.cgi?view=details&query=GO:0005575)  [intracellular](http://amigo.geneontology.org/cgi-bin/amigo/go.cgi?view=details&query=GO:0005622)  [cytoplasm](http://amigo.geneontology.org/cgi-bin/amigo/go.cgi?view=details&query=GO:0005737)  [Golgi apparatus](http://amigo.geneontology.org/cgi-bin/amigo/go.cgi?view=details&query=GO:0005794)  [cytoskeleton](http://amigo.geneontology.org/cgi-bin/amigo/go.cgi?view=details&query=GO:0005856)  [carbohydrate metabolic process](http://amigo.geneontology.org/cgi-bin/amigo/go.cgi?view=details&query=GO:0005975)  [N-acetylglucosamine metabolic process](http://amigo.geneontology.org/cgi-bin/amigo/go.cgi?view=details&query=GO:0006044)  [N-acetylglucosamine metabolic process](http://amigo.geneontology.org/cgi-bin/amigo/go.cgi?view=details&query=GO:0006044)  [sulfur compound metabolic process](http://amigo.geneontology.org/cgi-bin/amigo/go.cgi?view=details&query=GO:0006790)  [biological_process](http://amigo.geneontology.org/cgi-bin/amigo/go.cgi?view=details&query=GO:0008150)  [membrane](http://amigo.geneontology.org/cgi-bin/amigo/go.cgi?view=details&query=GO:0016020)  [integral to membrane](http://amigo.geneontology.org/cgi-bin/amigo/go.cgi?view=details&query=GO:0016021)  [transferase activity](http://amigo.geneontology.org/cgi-bin/amigo/go.cgi?view=details&query=GO:0016740)  [keratan sulfate biosynthetic process](http://amigo.geneontology.org/cgi-bin/amigo/go.cgi?view=details&query=GO:0018146)  [enzyme binding](http://amigo.geneontology.org/cgi-bin/amigo/go.cgi?view=details&query=GO:0019899)  [defense response to fungus](http://amigo.geneontology.org/cgi-bin/amigo/go.cgi?view=details&query=GO:0050832) |  |
| [221215_s_at](https://www.affymetrix.com/LinkServlet?&probeset=221215_s_at) | RIPK4 | receptor-interacting serine-threonine kinase 4 | 21 | -43159528 | [NM_020639](http://www.ncbi.nlm.nih.gov/entrez/query.fcgi?cmd=search&db=nucleotide&term=NM_020639%5BACCN%5D&doptcmdl=GenBank) | [54101](http://www.ncbi.nlm.nih.gov/sites/entrez?Db=gene&Cmd=DetailsSearch&Term=54101) | [21q22.3](http://www.ncbi.nlm.nih.gov/mapview/map_search.cgi?direct=on&idtype=gene&id=54101) | [Hs.517310](http://www.ncbi.nlm.nih.gov/UniGene/clust.cgi?ORG=Hs&CID=517310) | [15](http://www.ncbi.nih.gov/entrez/query.fcgi?tool=bioconductor&cmd=Retrieve&db=PubMed&list_uids=8889548%2C9199174%2C10830953%2C10948194%2C11278382%2C12446564%2C12477932%2C12676934%2C12747765%2C14702039%2C17039240%2C18025152%2C18511573%2C19818768%2C20379614) | [nucleotide binding](http://amigo.geneontology.org/cgi-bin/amigo/go.cgi?view=details&query=GO:0000166)  [protein serine/threonine kinase activity](http://amigo.geneontology.org/cgi-bin/amigo/go.cgi?view=details&query=GO:0004674)  [receptor activity](http://amigo.geneontology.org/cgi-bin/amigo/go.cgi?view=details&query=GO:0004872)  [ATP binding](http://amigo.geneontology.org/cgi-bin/amigo/go.cgi?view=details&query=GO:0005524)  [cytoplasm](http://amigo.geneontology.org/cgi-bin/amigo/go.cgi?view=details&query=GO:0005737)  [protein phosphorylation](http://amigo.geneontology.org/cgi-bin/amigo/go.cgi?view=details&query=GO:0006468)  [membrane](http://amigo.geneontology.org/cgi-bin/amigo/go.cgi?view=details&query=GO:0016020)  [transferase activity](http://amigo.geneontology.org/cgi-bin/amigo/go.cgi?view=details&query=GO:0016740) |  |
| [221261_x_at](https://www.affymetrix.com/LinkServlet?&probeset=221261_x_at) |  |  |  |  | [NM_030801](http://www.ncbi.nlm.nih.gov/entrez/query.fcgi?cmd=search&db=nucleotide&term=NM_030801%5BACCN%5D&doptcmdl=GenBank) |  |  |  |  |  |  |
| [221593_s_at](https://www.affymetrix.com/LinkServlet?&probeset=221593_s_at) | RPL31 | ribosomal protein L31 | 2 | 101618690, 101618690 | [BC001663](http://www.ncbi.nlm.nih.gov/entrez/query.fcgi?cmd=search&db=nucleotide&term=BC001663%5BACCN%5D&doptcmdl=GenBank) | [6160](http://www.ncbi.nlm.nih.gov/sites/entrez?Db=gene&Cmd=DetailsSearch&Term=6160) | [2q11.2](http://www.ncbi.nlm.nih.gov/mapview/map_search.cgi?direct=on&idtype=gene&id=6160) | [Hs.469473](http://www.ncbi.nlm.nih.gov/UniGene/clust.cgi?ORG=Hs&CID=469473) | [21](http://www.ncbi.nih.gov/entrez/query.fcgi?tool=bioconductor&cmd=Retrieve&db=PubMed&list_uids=2597680%2C2780320%2C7821789%2C8722009%2C8889548%2C9582194%2C11746496%2C11790298%2C11875025%2C12477932%2C12962325%2C14567916%2C15146197%2C15189156%2C15489334%2C15592455%2C15815621%2C16189514%2C16344560%2C17081983%2C17353931) | [RNA binding](http://amigo.geneontology.org/cgi-bin/amigo/go.cgi?view=details&query=GO:0003723)  [structural constituent of ribosome](http://amigo.geneontology.org/cgi-bin/amigo/go.cgi?view=details&query=GO:0003735)  [protein binding](http://amigo.geneontology.org/cgi-bin/amigo/go.cgi?view=details&query=GO:0005515)  [intracellular](http://amigo.geneontology.org/cgi-bin/amigo/go.cgi?view=details&query=GO:0005622)  [cytosol](http://amigo.geneontology.org/cgi-bin/amigo/go.cgi?view=details&query=GO:0005829)  [cytosol](http://amigo.geneontology.org/cgi-bin/amigo/go.cgi?view=details&query=GO:0005829)  [ribosome](http://amigo.geneontology.org/cgi-bin/amigo/go.cgi?view=details&query=GO:0005840)  [translation](http://amigo.geneontology.org/cgi-bin/amigo/go.cgi?view=details&query=GO:0006412)  [translational elongation](http://amigo.geneontology.org/cgi-bin/amigo/go.cgi?view=details&query=GO:0006414)  [translational termination](http://amigo.geneontology.org/cgi-bin/amigo/go.cgi?view=details&query=GO:0006415)  [gene expression](http://amigo.geneontology.org/cgi-bin/amigo/go.cgi?view=details&query=GO:0010467)  [viral reproduction](http://amigo.geneontology.org/cgi-bin/amigo/go.cgi?view=details&query=GO:0016032)  [viral infectious cycle](http://amigo.geneontology.org/cgi-bin/amigo/go.cgi?view=details&query=GO:0019058)  [viral transcription](http://amigo.geneontology.org/cgi-bin/amigo/go.cgi?view=details&query=GO:0019083)  [cytosolic large ribosomal subunit](http://amigo.geneontology.org/cgi-bin/amigo/go.cgi?view=details&query=GO:0022625)  [endocrine pancreas development](http://amigo.geneontology.org/cgi-bin/amigo/go.cgi?view=details&query=GO:0031018)  [cellular protein metabolic process](http://amigo.geneontology.org/cgi-bin/amigo/go.cgi?view=details&query=GO:0044267) | [Ribosome](http://www.genome.ad.jp/kegg/pathway/hsa/hsa03010.html) |
| [221802_s_at](https://www.affymetrix.com/LinkServlet?&probeset=221802_s_at) | KIAA1598 | KIAA1598 | 10 | -118644307 | [AU157109](http://www.ncbi.nlm.nih.gov/entrez/query.fcgi?cmd=search&db=nucleotide&term=AU157109%5BACCN%5D&doptcmdl=GenBank) | [57698](http://www.ncbi.nlm.nih.gov/sites/entrez?Db=gene&Cmd=DetailsSearch&Term=57698) | [10q25.3](http://www.ncbi.nlm.nih.gov/mapview/map_search.cgi?direct=on&idtype=gene&id=57698) | [Hs.501140](http://www.ncbi.nlm.nih.gov/UniGene/clust.cgi?ORG=Hs&CID=501140) | [12](http://www.ncbi.nih.gov/entrez/query.fcgi?tool=bioconductor&cmd=Retrieve&db=PubMed&list_uids=10997877%2C12421765%2C12477932%2C14702039%2C16344560%2C16385451%2C17030985%2C17903304%2C18519736%2C20023658%2C20098747%2C20379614) | [multicellular organismal development](http://amigo.geneontology.org/cgi-bin/amigo/go.cgi?view=details&query=GO:0007275)  [axon guidance](http://amigo.geneontology.org/cgi-bin/amigo/go.cgi?view=details&query=GO:0007411)  [axon](http://amigo.geneontology.org/cgi-bin/amigo/go.cgi?view=details&query=GO:0030424)  [cell projection](http://amigo.geneontology.org/cgi-bin/amigo/go.cgi?view=details&query=GO:0042995) |  |
| [222347_at](https://www.affymetrix.com/LinkServlet?&probeset=222347_at) | LOC644450 | hypothetical LOC644450 | 1 |  | [AI050036](http://www.ncbi.nlm.nih.gov/entrez/query.fcgi?cmd=search&db=nucleotide&term=AI050036%5BACCN%5D&doptcmdl=GenBank) | [644450](http://www.ncbi.nlm.nih.gov/sites/entrez?Db=gene&Cmd=DetailsSearch&Term=644450) | [1q12](http://www.ncbi.nlm.nih.gov/mapview/map_search.cgi?direct=on&idtype=gene&id=644450) | [Hs.652926](http://www.ncbi.nlm.nih.gov/UniGene/clust.cgi?ORG=Hs&CID=652926) | [1](http://www.ncbi.nih.gov/entrez/query.fcgi?tool=bioconductor&cmd=Retrieve&db=PubMed&list_uids=12477932) |  |  |
| [222662_at](https://www.affymetrix.com/LinkServlet?&probeset=222662_at) | PPP1R3B | protein phosphatase 1, regulatory (inhibitor) subunit 3B | 8 | -8993773 | [W60806](http://www.ncbi.nlm.nih.gov/entrez/query.fcgi?cmd=search&db=nucleotide&term=W60806%5BACCN%5D&doptcmdl=GenBank) | [79660](http://www.ncbi.nlm.nih.gov/sites/entrez?Db=gene&Cmd=DetailsSearch&Term=79660) | [8p23.1](http://www.ncbi.nlm.nih.gov/mapview/map_search.cgi?direct=on&idtype=gene&id=79660) | [Hs.458513](http://www.ncbi.nlm.nih.gov/UniGene/clust.cgi?ORG=Hs&CID=458513) | [11](http://www.ncbi.nih.gov/entrez/query.fcgi?tool=bioconductor&cmd=Retrieve&db=PubMed&list_uids=7498521%2C11872655%2C11948623%2C12477932%2C14702039%2C15231748%2C16344560%2C16907705%2C17207965%2C17555403%2C20864672) | [carbohydrate metabolic process](http://amigo.geneontology.org/cgi-bin/amigo/go.cgi?view=details&query=GO:0005975)  [glycogen metabolic process](http://amigo.geneontology.org/cgi-bin/amigo/go.cgi?view=details&query=GO:0005977) | [Insulin signaling pathway](http://www.genome.ad.jp/kegg/pathway/hsa/hsa04910.html) |
| [222747_s_at](https://www.affymetrix.com/LinkServlet?&probeset=222747_s_at) | SCML1 | sex comb on midleg-like 1 (Drosophila) | X | 17755591 | [BF001786](http://www.ncbi.nlm.nih.gov/entrez/query.fcgi?cmd=search&db=nucleotide&term=BF001786%5BACCN%5D&doptcmdl=GenBank) | [6322](http://www.ncbi.nlm.nih.gov/sites/entrez?Db=gene&Cmd=DetailsSearch&Term=6322) | [Xp22](http://www.ncbi.nlm.nih.gov/mapview/map_search.cgi?direct=on&idtype=gene&id=6322) | [Hs.109655](http://www.ncbi.nlm.nih.gov/UniGene/clust.cgi?ORG=Hs&CID=109655) | [5](http://www.ncbi.nih.gov/entrez/query.fcgi?tool=bioconductor&cmd=Retrieve&db=PubMed&list_uids=9570953%2C12477932%2C15489334%2C15772651%2C18601738) | [DNA binding](http://amigo.geneontology.org/cgi-bin/amigo/go.cgi?view=details&query=GO:0003677)  [sequence-specific DNA binding transcription factor activity](http://amigo.geneontology.org/cgi-bin/amigo/go.cgi?view=details&query=GO:0003700)  [nucleus](http://amigo.geneontology.org/cgi-bin/amigo/go.cgi?view=details&query=GO:0005634)  [transcription, DNA-dependent](http://amigo.geneontology.org/cgi-bin/amigo/go.cgi?view=details&query=GO:0006351)  [anatomical structure morphogenesis](http://amigo.geneontology.org/cgi-bin/amigo/go.cgi?view=details&query=GO:0009653)  [regulation of transcription](http://amigo.geneontology.org/cgi-bin/amigo/go.cgi?view=details&query=GO:0045449) |  |
| [223062_s_at](https://www.affymetrix.com/LinkServlet?&probeset=223062_s_at) | PSAT1 | phosphoserine aminotransferase 1 | 9 | 80912058 | [BC004863](http://www.ncbi.nlm.nih.gov/entrez/query.fcgi?cmd=search&db=nucleotide&term=BC004863%5BACCN%5D&doptcmdl=GenBank) | [29968](http://www.ncbi.nlm.nih.gov/sites/entrez?Db=gene&Cmd=DetailsSearch&Term=29968) | [9q21.2](http://www.ncbi.nlm.nih.gov/mapview/map_search.cgi?direct=on&idtype=gene&id=29968) | [Hs.494261](http://www.ncbi.nlm.nih.gov/UniGene/clust.cgi?ORG=Hs&CID=494261) [Hs.592595](http://www.ncbi.nlm.nih.gov/UniGene/clust.cgi?ORG=Hs&CID=592595) | [6](http://www.ncbi.nih.gov/entrez/query.fcgi?tool=bioconductor&cmd=Retrieve&db=PubMed&list_uids=3651428%2C10637769%2C12477932%2C12633500%2C15489334%2C18221502) | [O-phospho-L-serine:2-oxoglutarate aminotransferase activity](http://amigo.geneontology.org/cgi-bin/amigo/go.cgi?view=details&query=GO:0004648)  [L-serine biosynthetic process](http://amigo.geneontology.org/cgi-bin/amigo/go.cgi?view=details&query=GO:0006564)  [pyridoxine biosynthetic process](http://amigo.geneontology.org/cgi-bin/amigo/go.cgi?view=details&query=GO:0008615)  [cellular amino acid biosynthetic process](http://amigo.geneontology.org/cgi-bin/amigo/go.cgi?view=details&query=GO:0008652)  [transferase activity](http://amigo.geneontology.org/cgi-bin/amigo/go.cgi?view=details&query=GO:0016740)  [pyridoxal phosphate binding](http://amigo.geneontology.org/cgi-bin/amigo/go.cgi?view=details&query=GO:0030170) | [Glycine, serine and threonine metabolism](http://www.genome.ad.jp/dbget-bin/show_pathway?MAP00260+2.6.1.52)  [Vitamin B6 metabolism](http://www.genome.ad.jp/dbget-bin/show_pathway?MAP00750+2.6.1.52)  [Metabolic pathways](http://www.genome.ad.jp/dbget-bin/show_pathway?MAP01100+2.6.1.52) |
| [223168_at](https://www.affymetrix.com/LinkServlet?&probeset=223168_at) | RHOU | ras homolog gene family, member U | 1 | 228870868 | [AL096776](http://www.ncbi.nlm.nih.gov/entrez/query.fcgi?cmd=search&db=nucleotide&term=AL096776%5BACCN%5D&doptcmdl=GenBank) | [58480](http://www.ncbi.nlm.nih.gov/sites/entrez?Db=gene&Cmd=DetailsSearch&Term=58480) | [1q42.11-q42.3](http://www.ncbi.nlm.nih.gov/mapview/map_search.cgi?direct=on&idtype=gene&id=58480) | [Hs.647774](http://www.ncbi.nlm.nih.gov/UniGene/clust.cgi?ORG=Hs&CID=647774) | [16](http://www.ncbi.nih.gov/entrez/query.fcgi?tool=bioconductor&cmd=Retrieve&db=PubMed&list_uids=11459829%2C11894124%2C12477932%2C14702039%2C14731133%2C15350535%2C15556869%2C16344560%2C16751668%2C17504809%2C18086875%2C18849993%2C19064640%2C19135548%2C20547754%2C20844546) | [G1/S transition of mitotic cell cycle](http://amigo.geneontology.org/cgi-bin/amigo/go.cgi?view=details&query=GO:0000082)  [Golgi membrane](http://amigo.geneontology.org/cgi-bin/amigo/go.cgi?view=details&query=GO:0000139)  [nucleotide binding](http://amigo.geneontology.org/cgi-bin/amigo/go.cgi?view=details&query=GO:0000166)  [podosome](http://amigo.geneontology.org/cgi-bin/amigo/go.cgi?view=details&query=GO:0002102)  [GTPase activity](http://amigo.geneontology.org/cgi-bin/amigo/go.cgi?view=details&query=GO:0003924)  [protein binding](http://amigo.geneontology.org/cgi-bin/amigo/go.cgi?view=details&query=GO:0005515)  [GTP binding](http://amigo.geneontology.org/cgi-bin/amigo/go.cgi?view=details&query=GO:0005525)  [intracellular](http://amigo.geneontology.org/cgi-bin/amigo/go.cgi?view=details&query=GO:0005622)  [Golgi apparatus](http://amigo.geneontology.org/cgi-bin/amigo/go.cgi?view=details&query=GO:0005794)  [cytosol](http://amigo.geneontology.org/cgi-bin/amigo/go.cgi?view=details&query=GO:0005829)  [plasma membrane](http://amigo.geneontology.org/cgi-bin/amigo/go.cgi?view=details&query=GO:0005886)  [focal adhesion](http://amigo.geneontology.org/cgi-bin/amigo/go.cgi?view=details&query=GO:0005925)  [small GTPase mediated signal transduction](http://amigo.geneontology.org/cgi-bin/amigo/go.cgi?view=details&query=GO:0007264)  [regulation of cell shape](http://amigo.geneontology.org/cgi-bin/amigo/go.cgi?view=details&query=GO:0008360)  [Rac protein signal transduction](http://amigo.geneontology.org/cgi-bin/amigo/go.cgi?view=details&query=GO:0016601)  [actin cytoskeleton organization](http://amigo.geneontology.org/cgi-bin/amigo/go.cgi?view=details&query=GO:0030036)  [cell junction](http://amigo.geneontology.org/cgi-bin/amigo/go.cgi?view=details&query=GO:0030054)  [cell projection](http://amigo.geneontology.org/cgi-bin/amigo/go.cgi?view=details&query=GO:0042995)  [metal ion binding](http://amigo.geneontology.org/cgi-bin/amigo/go.cgi?view=details&query=GO:0046872)  [regulation of small GTPase mediated signal transduction](http://amigo.geneontology.org/cgi-bin/amigo/go.cgi?view=details&query=GO:0051056) |  |
| [223170_at](https://www.affymetrix.com/LinkServlet?&probeset=223170_at) | TMEM98 | transmembrane protein 98 | 17 | 31254927 | [AF132000](http://www.ncbi.nlm.nih.gov/entrez/query.fcgi?cmd=search&db=nucleotide&term=AF132000%5BACCN%5D&doptcmdl=GenBank) | [26022](http://www.ncbi.nlm.nih.gov/sites/entrez?Db=gene&Cmd=DetailsSearch&Term=26022) | [17q11.2](http://www.ncbi.nlm.nih.gov/mapview/map_search.cgi?direct=on&idtype=gene&id=26022) | [Hs.3447](http://www.ncbi.nlm.nih.gov/UniGene/clust.cgi?ORG=Hs&CID=3447) | [8](http://www.ncbi.nih.gov/entrez/query.fcgi?tool=bioconductor&cmd=Retrieve&db=PubMed&list_uids=11076863%2C11230166%2C11256614%2C12477932%2C12975309%2C15489334%2C15489336%2C16381901) | [endoplasmic reticulum](http://amigo.geneontology.org/cgi-bin/amigo/go.cgi?view=details&query=GO:0005783)  [membrane](http://amigo.geneontology.org/cgi-bin/amigo/go.cgi?view=details&query=GO:0016020)  [integral to membrane](http://amigo.geneontology.org/cgi-bin/amigo/go.cgi?view=details&query=GO:0016021) |  |
| [223313_s_at](https://www.affymetrix.com/LinkServlet?&probeset=223313_s_at) |  |  |  |  | [BC001207](http://www.ncbi.nlm.nih.gov/entrez/query.fcgi?cmd=search&db=nucleotide&term=BC001207%5BACCN%5D&doptcmdl=GenBank) |  |  |  |  |  |  |
| [223593_at](https://www.affymetrix.com/LinkServlet?&probeset=223593_at) | AADAT | aminoadipate aminotransferase | 4 | -170981373, -170981373 | [AF097994](http://www.ncbi.nlm.nih.gov/entrez/query.fcgi?cmd=search&db=nucleotide&term=AF097994%5BACCN%5D&doptcmdl=GenBank) | [51166](http://www.ncbi.nlm.nih.gov/sites/entrez?Db=gene&Cmd=DetailsSearch&Term=51166) | [4q33](http://www.ncbi.nlm.nih.gov/mapview/map_search.cgi?direct=on&idtype=gene&id=51166) | [Hs.529735](http://www.ncbi.nlm.nih.gov/UniGene/clust.cgi?ORG=Hs&CID=529735) | [12](http://www.ncbi.nih.gov/entrez/query.fcgi?tool=bioconductor&cmd=Retrieve&db=PubMed&list_uids=1798692%2C8087205%2C10441733%2C12126930%2C12477932%2C14702039%2C15489334%2C17207965%2C18056995%2C18056996%2C18620547%2C20877624) | [mitochondrion](http://amigo.geneontology.org/cgi-bin/amigo/go.cgi?view=details&query=GO:0005739)  [mitochondrial matrix](http://amigo.geneontology.org/cgi-bin/amigo/go.cgi?view=details&query=GO:0005759)  [2-oxoglutarate metabolic process](http://amigo.geneontology.org/cgi-bin/amigo/go.cgi?view=details&query=GO:0006103)  [glutamate metabolic process](http://amigo.geneontology.org/cgi-bin/amigo/go.cgi?view=details&query=GO:0006536)  [lysine catabolic process](http://amigo.geneontology.org/cgi-bin/amigo/go.cgi?view=details&query=GO:0006554)  [tryptophan catabolic process](http://amigo.geneontology.org/cgi-bin/amigo/go.cgi?view=details&query=GO:0006569)  [biosynthetic process](http://amigo.geneontology.org/cgi-bin/amigo/go.cgi?view=details&query=GO:0009058)  [kynurenine-oxoglutarate transaminase activity](http://amigo.geneontology.org/cgi-bin/amigo/go.cgi?view=details&query=GO:0016212)  [kynurenine-oxoglutarate transaminase activity](http://amigo.geneontology.org/cgi-bin/amigo/go.cgi?view=details&query=GO:0016212)  [pyridoxal phosphate binding](http://amigo.geneontology.org/cgi-bin/amigo/go.cgi?view=details&query=GO:0030170)  [cellular nitrogen compound metabolic process](http://amigo.geneontology.org/cgi-bin/amigo/go.cgi?view=details&query=GO:0034641)  [protein homodimerization activity](http://amigo.geneontology.org/cgi-bin/amigo/go.cgi?view=details&query=GO:0042803)  [2-aminoadipate transaminase activity](http://amigo.geneontology.org/cgi-bin/amigo/go.cgi?view=details&query=GO:0047536)  [2-aminoadipate transaminase activity](http://amigo.geneontology.org/cgi-bin/amigo/go.cgi?view=details&query=GO:0047536)  [kynurenine metabolic process](http://amigo.geneontology.org/cgi-bin/amigo/go.cgi?view=details&query=GO:0070189) | [Lysine biosynthesis](http://www.genome.ad.jp/dbget-bin/show_pathway?MAP00300+2.6.1.39)  [Lysine degradation](http://www.genome.ad.jp/dbget-bin/show_pathway?MAP00310+2.6.1.39)  [Tryptophan metabolism](http://www.genome.ad.jp/dbget-bin/show_pathway?MAP00380+2.6.1.39)  [Metabolic pathways](http://www.genome.ad.jp/dbget-bin/show_pathway?MAP01100+2.6.1.39) |
| [223614_at](https://www.affymetrix.com/LinkServlet?&probeset=223614_at) | MMP16 | matrix metallopeptidase 16 (membrane-inserted) | 8 | -89081460, -89049461 | [AL136588](http://www.ncbi.nlm.nih.gov/entrez/query.fcgi?cmd=search&db=nucleotide&term=AL136588%5BACCN%5D&doptcmdl=GenBank) | [4325](http://www.ncbi.nlm.nih.gov/sites/entrez?Db=gene&Cmd=DetailsSearch&Term=4325) | [8q21.3](http://www.ncbi.nlm.nih.gov/mapview/map_search.cgi?direct=on&idtype=gene&id=4325) | [Hs.492187](http://www.ncbi.nlm.nih.gov/UniGene/clust.cgi?ORG=Hs&CID=492187) [Hs.546267](http://www.ncbi.nlm.nih.gov/UniGene/clust.cgi?ORG=Hs&CID=546267) | [33](http://www.ncbi.nih.gov/entrez/query.fcgi?tool=bioconductor&cmd=Retrieve&db=PubMed&list_uids=7559440%2C8619474%2C9070935%2C9092507%2C9110174%2C9119382%2C9396633%2C10419448%2C10949161%2C11230166%2C11278606%2C12477932%2C12661033%2C12879005%2C14645246%2C14741217%2C15044209%2C15380516%2C15489334%2C16344560%2C16983145%2C17217338%2C17419254%2C18784838%2C19240061%2C19265686%2C19913121%2C20207250%2C20452482%2C20587546%2C20628086%2C20673868%2C21048031) | [metalloendopeptidase activity](http://amigo.geneontology.org/cgi-bin/amigo/go.cgi?view=details&query=GO:0004222)  [calcium ion binding](http://amigo.geneontology.org/cgi-bin/amigo/go.cgi?view=details&query=GO:0005509)  [extracellular region](http://amigo.geneontology.org/cgi-bin/amigo/go.cgi?view=details&query=GO:0005576)  [proteinaceous extracellular matrix](http://amigo.geneontology.org/cgi-bin/amigo/go.cgi?view=details&query=GO:0005578)  [plasma membrane](http://amigo.geneontology.org/cgi-bin/amigo/go.cgi?view=details&query=GO:0005886)  [integral to plasma membrane](http://amigo.geneontology.org/cgi-bin/amigo/go.cgi?view=details&query=GO:0005887)  [proteolysis](http://amigo.geneontology.org/cgi-bin/amigo/go.cgi?view=details&query=GO:0006508)  [enzyme activator activity](http://amigo.geneontology.org/cgi-bin/amigo/go.cgi?view=details&query=GO:0008047)  [metabolic process](http://amigo.geneontology.org/cgi-bin/amigo/go.cgi?view=details&query=GO:0008152)  [peptidase activity](http://amigo.geneontology.org/cgi-bin/amigo/go.cgi?view=details&query=GO:0008233)  [zinc ion binding](http://amigo.geneontology.org/cgi-bin/amigo/go.cgi?view=details&query=GO:0008270)  [cell surface](http://amigo.geneontology.org/cgi-bin/amigo/go.cgi?view=details&query=GO:0009986)  [collagen catabolic process](http://amigo.geneontology.org/cgi-bin/amigo/go.cgi?view=details&query=GO:0030574) |  |
| [223839_s_at](https://www.affymetrix.com/LinkServlet?&probeset=223839_s_at) | SCD | stearoyl-CoA desaturase (delta-9-desaturase) | 10 | 102106771 | [AF132203](http://www.ncbi.nlm.nih.gov/entrez/query.fcgi?cmd=search&db=nucleotide&term=AF132203%5BACCN%5D&doptcmdl=GenBank) | [6319](http://www.ncbi.nlm.nih.gov/sites/entrez?Db=gene&Cmd=DetailsSearch&Term=6319) | [10q24.31](http://www.ncbi.nlm.nih.gov/mapview/map_search.cgi?direct=on&idtype=gene&id=6319) | [Hs.558396](http://www.ncbi.nlm.nih.gov/UniGene/clust.cgi?ORG=Hs&CID=558396) [Hs.597496](http://www.ncbi.nlm.nih.gov/UniGene/clust.cgi?ORG=Hs&CID=597496) | [55](http://www.ncbi.nih.gov/entrez/query.fcgi?tool=bioconductor&cmd=Retrieve&db=PubMed&list_uids=21148%2C6102994%2C7909540%2C8125298%2C9362069%2C9373149%2C10229681%2C10922050%2C11181995%2C11397803%2C11415448%2C11677241%2C12061775%2C12401889%2C12477932%2C14683458%2C14967817%2C14967823%2C15164054%2C15489334%2C15609334%2C15610069%2C15662557%2C15708362%2C15851470%2C15855323%2C15907797%2C16213227%2C16385451%2C16723740%2C17614770%2C17636091%2C17852835%2C18029348%2C18030445%2C18286258%2C18340007%2C18499418%2C18660489%2C18697866%2C18813799%2C18832746%2C18952834%2C19130493%2C19154947%2C19478146%2C19710915%2C19913121%2C20032470%2C20395685%2C20565855%2C20579763%2C20599700%2C20628086%2C21045174) | [stearoyl-CoA 9-desaturase activity](http://amigo.geneontology.org/cgi-bin/amigo/go.cgi?view=details&query=GO:0004768)  [iron ion binding](http://amigo.geneontology.org/cgi-bin/amigo/go.cgi?view=details&query=GO:0005506)  [endoplasmic reticulum](http://amigo.geneontology.org/cgi-bin/amigo/go.cgi?view=details&query=GO:0005783)  [endoplasmic reticulum membrane](http://amigo.geneontology.org/cgi-bin/amigo/go.cgi?view=details&query=GO:0005789)  [fatty acid biosynthetic process](http://amigo.geneontology.org/cgi-bin/amigo/go.cgi?view=details&query=GO:0006633)  [membrane](http://amigo.geneontology.org/cgi-bin/amigo/go.cgi?view=details&query=GO:0016020)  [integral to membrane](http://amigo.geneontology.org/cgi-bin/amigo/go.cgi?view=details&query=GO:0016021)  [oxidoreductase activity](http://amigo.geneontology.org/cgi-bin/amigo/go.cgi?view=details&query=GO:0016491)  [oxidoreductase activity, acting on paired donors, with oxidation of a pair of donors resulting in the reduction of molecular oxygen to two molecules of water](http://amigo.geneontology.org/cgi-bin/amigo/go.cgi?view=details&query=GO:0016717)  [oxidation-reduction process](http://amigo.geneontology.org/cgi-bin/amigo/go.cgi?view=details&query=GO:0055114) | [Biosynthesis of unsaturated fatty acids](http://www.genome.ad.jp/dbget-bin/show_pathway?MAP01040+1.14.19.1)  [PPAR signaling pathway](http://www.genome.ad.jp/dbget-bin/show_pathway?MAP03320+1.14.19.1) |
| [224583_at](https://www.affymetrix.com/LinkServlet?&probeset=224583_at) | COTL1 | coactosin-like 1 (Dictyostelium) | 16 | -84599205 | [AL565621](http://www.ncbi.nlm.nih.gov/entrez/query.fcgi?cmd=search&db=nucleotide&term=AL565621%5BACCN%5D&doptcmdl=GenBank) | [23406](http://www.ncbi.nlm.nih.gov/sites/entrez?Db=gene&Cmd=DetailsSearch&Term=23406) | [16q24.1](http://www.ncbi.nlm.nih.gov/mapview/map_search.cgi?direct=on&idtype=gene&id=23406) | [Hs.289092](http://www.ncbi.nlm.nih.gov/UniGene/clust.cgi?ORG=Hs&CID=289092) | [19](http://www.ncbi.nih.gov/entrez/query.fcgi?tool=bioconductor&cmd=Retrieve&db=PubMed&list_uids=7690594%2C9326934%2C10051563%2C11297527%2C11583571%2C11870627%2C12477932%2C12665801%2C14702039%2C15213466%2C15333945%2C15459340%2C15489334%2C15583396%2C16097034%2C16924104%2C17070122%2C19307756%2C19807693) | [actin binding](http://amigo.geneontology.org/cgi-bin/amigo/go.cgi?view=details&query=GO:0003779)  [protein binding](http://amigo.geneontology.org/cgi-bin/amigo/go.cgi?view=details&query=GO:0005515)  [cellular_component](http://amigo.geneontology.org/cgi-bin/amigo/go.cgi?view=details&query=GO:0005575)  [intracellular](http://amigo.geneontology.org/cgi-bin/amigo/go.cgi?view=details&query=GO:0005622)  [cytoplasm](http://amigo.geneontology.org/cgi-bin/amigo/go.cgi?view=details&query=GO:0005737)  [cytoskeleton](http://amigo.geneontology.org/cgi-bin/amigo/go.cgi?view=details&query=GO:0005856)  [biological_process](http://amigo.geneontology.org/cgi-bin/amigo/go.cgi?view=details&query=GO:0008150)  [enzyme binding](http://amigo.geneontology.org/cgi-bin/amigo/go.cgi?view=details&query=GO:0019899)  [defense response to fungus](http://amigo.geneontology.org/cgi-bin/amigo/go.cgi?view=details&query=GO:0050832) |  |
| [224839_s_at](https://www.affymetrix.com/LinkServlet?&probeset=224839_s_at) | GPT2 | glutamic pyruvate transaminase (alanine aminotransferase) 2 | 16 | 46918307, 46919101 | [BG328998](http://www.ncbi.nlm.nih.gov/entrez/query.fcgi?cmd=search&db=nucleotide&term=BG328998%5BACCN%5D&doptcmdl=GenBank) | [84706](http://www.ncbi.nlm.nih.gov/sites/entrez?Db=gene&Cmd=DetailsSearch&Term=84706) | [16q12.1](http://www.ncbi.nlm.nih.gov/mapview/map_search.cgi?direct=on&idtype=gene&id=84706) | [Hs.460693](http://www.ncbi.nlm.nih.gov/UniGene/clust.cgi?ORG=Hs&CID=460693) | [11](http://www.ncbi.nih.gov/entrez/query.fcgi?tool=bioconductor&cmd=Retrieve&db=PubMed&list_uids=11863375%2C12477932%2C14702039%2C15489334%2C15616553%2C16344560%2C17109502%2C17596883%2C19360321%2C19615732%2C20877624) | [L-alanine:2-oxoglutarate aminotransferase activity](http://amigo.geneontology.org/cgi-bin/amigo/go.cgi?view=details&query=GO:0004021)  [L-alanine:2-oxoglutarate aminotransferase activity](http://amigo.geneontology.org/cgi-bin/amigo/go.cgi?view=details&query=GO:0004021)  [mitochondrial matrix](http://amigo.geneontology.org/cgi-bin/amigo/go.cgi?view=details&query=GO:0005759)  [2-oxoglutarate metabolic process](http://amigo.geneontology.org/cgi-bin/amigo/go.cgi?view=details&query=GO:0006103)  [transaminase activity](http://amigo.geneontology.org/cgi-bin/amigo/go.cgi?view=details&query=GO:0008483)  [cellular amino acid biosynthetic process](http://amigo.geneontology.org/cgi-bin/amigo/go.cgi?view=details&query=GO:0008652)  [biosynthetic process](http://amigo.geneontology.org/cgi-bin/amigo/go.cgi?view=details&query=GO:0009058)  [pyridoxal phosphate binding](http://amigo.geneontology.org/cgi-bin/amigo/go.cgi?view=details&query=GO:0030170)  [cellular nitrogen compound metabolic process](http://amigo.geneontology.org/cgi-bin/amigo/go.cgi?view=details&query=GO:0034641)  [L-alanine metabolic process](http://amigo.geneontology.org/cgi-bin/amigo/go.cgi?view=details&query=GO:0042851) | [Alanine, aspartate and glutamate metabolism](http://www.genome.ad.jp/dbget-bin/show_pathway?MAP00250+2.6.1.2)  [Metabolic pathways](http://www.genome.ad.jp/dbget-bin/show_pathway?MAP01100+2.6.1.2) |
| [225270_at](https://www.affymetrix.com/LinkServlet?&probeset=225270_at) | NEO1 | neogenin 1 | 15 | 73344824 | [AL355708](http://www.ncbi.nlm.nih.gov/entrez/query.fcgi?cmd=search&db=nucleotide&term=AL355708%5BACCN%5D&doptcmdl=GenBank) | [4756](http://www.ncbi.nlm.nih.gov/sites/entrez?Db=gene&Cmd=DetailsSearch&Term=4756) | [15q22.3-q23](http://www.ncbi.nlm.nih.gov/mapview/map_search.cgi?direct=on&idtype=gene&id=4756) | [Hs.388613](http://www.ncbi.nlm.nih.gov/UniGene/clust.cgi?ORG=Hs&CID=388613) | [23](http://www.ncbi.nih.gov/entrez/query.fcgi?tool=bioconductor&cmd=Retrieve&db=PubMed&list_uids=8861902%2C9121761%2C9169140%2C10366627%2C12477932%2C12636918%2C12833147%2C15258591%2C15489334%2C15494733%2C15923648%2C16244667%2C16324219%2C16335952%2C18029348%2C18287331%2C18326817%2C18391016%2C18445598%2C18583991%2C19564337%2C20237496%2C20575069) | [receptor activity](http://amigo.geneontology.org/cgi-bin/amigo/go.cgi?view=details&query=GO:0004872)  [nucleus](http://amigo.geneontology.org/cgi-bin/amigo/go.cgi?view=details&query=GO:0005634)  [cytoplasm](http://amigo.geneontology.org/cgi-bin/amigo/go.cgi?view=details&query=GO:0005737)  [Golgi apparatus](http://amigo.geneontology.org/cgi-bin/amigo/go.cgi?view=details&query=GO:0005794)  [plasma membrane](http://amigo.geneontology.org/cgi-bin/amigo/go.cgi?view=details&query=GO:0005886)  [plasma membrane](http://amigo.geneontology.org/cgi-bin/amigo/go.cgi?view=details&query=GO:0005886)  [integral to plasma membrane](http://amigo.geneontology.org/cgi-bin/amigo/go.cgi?view=details&query=GO:0005887)  [cell adhesion](http://amigo.geneontology.org/cgi-bin/amigo/go.cgi?view=details&query=GO:0007155)  [axon guidance](http://amigo.geneontology.org/cgi-bin/amigo/go.cgi?view=details&query=GO:0007411)  [myoblast fusion](http://amigo.geneontology.org/cgi-bin/amigo/go.cgi?view=details&query=GO:0007520)  [transcription regulator activity](http://amigo.geneontology.org/cgi-bin/amigo/go.cgi?view=details&query=GO:0030528)  [muscle cell differentiation](http://amigo.geneontology.org/cgi-bin/amigo/go.cgi?view=details&query=GO:0042692)  [cadherin binding](http://amigo.geneontology.org/cgi-bin/amigo/go.cgi?view=details&query=GO:0045296)  [positive regulation of muscle cell differentiation](http://amigo.geneontology.org/cgi-bin/amigo/go.cgi?view=details&query=GO:0051149) | [Cell adhesion molecules (CAMs)](http://www.genome.ad.jp/kegg/pathway/hsa/hsa04514.html) |
| [225464_at](https://www.affymetrix.com/LinkServlet?&probeset=225464_at) | FRMD6 | FERM domain containing 6 | 14 | 51955854, 52118575 | [N30138](http://www.ncbi.nlm.nih.gov/entrez/query.fcgi?cmd=search&db=nucleotide&term=N30138%5BACCN%5D&doptcmdl=GenBank) | [122786](http://www.ncbi.nlm.nih.gov/sites/entrez?Db=gene&Cmd=DetailsSearch&Term=122786) | [14q22.1](http://www.ncbi.nlm.nih.gov/mapview/map_search.cgi?direct=on&idtype=gene&id=122786) | [Hs.434914](http://www.ncbi.nlm.nih.gov/UniGene/clust.cgi?ORG=Hs&CID=434914) | [12](http://www.ncbi.nih.gov/entrez/query.fcgi?tool=bioconductor&cmd=Retrieve&db=PubMed&list_uids=12477932%2C12508121%2C14702039%2C15324660%2C15489334%2C16137681%2C16189514%2C16341207%2C18029348%2C19668339%2C20171287%2C21116278) | [binding](http://amigo.geneontology.org/cgi-bin/amigo/go.cgi?view=details&query=GO:0005488)  [cytoplasm](http://amigo.geneontology.org/cgi-bin/amigo/go.cgi?view=details&query=GO:0005737)  [mitochondrion](http://amigo.geneontology.org/cgi-bin/amigo/go.cgi?view=details&query=GO:0005739)  [cytoskeleton](http://amigo.geneontology.org/cgi-bin/amigo/go.cgi?view=details&query=GO:0005856)  [plasma membrane](http://amigo.geneontology.org/cgi-bin/amigo/go.cgi?view=details&query=GO:0005886) |  |
| [225481_at](https://www.affymetrix.com/LinkServlet?&probeset=225481_at) | FRMD6 | FERM domain containing 6 | 14 | 51955854, 52118575 | [AL040051](http://www.ncbi.nlm.nih.gov/entrez/query.fcgi?cmd=search&db=nucleotide&term=AL040051%5BACCN%5D&doptcmdl=GenBank) | [122786](http://www.ncbi.nlm.nih.gov/sites/entrez?Db=gene&Cmd=DetailsSearch&Term=122786) | [14q22.1](http://www.ncbi.nlm.nih.gov/mapview/map_search.cgi?direct=on&idtype=gene&id=122786) | [Hs.434914](http://www.ncbi.nlm.nih.gov/UniGene/clust.cgi?ORG=Hs&CID=434914) | [12](http://www.ncbi.nih.gov/entrez/query.fcgi?tool=bioconductor&cmd=Retrieve&db=PubMed&list_uids=12477932%2C12508121%2C14702039%2C15324660%2C15489334%2C16137681%2C16189514%2C16341207%2C18029348%2C19668339%2C20171287%2C21116278) | [binding](http://amigo.geneontology.org/cgi-bin/amigo/go.cgi?view=details&query=GO:0005488)  [cytoplasm](http://amigo.geneontology.org/cgi-bin/amigo/go.cgi?view=details&query=GO:0005737)  [mitochondrion](http://amigo.geneontology.org/cgi-bin/amigo/go.cgi?view=details&query=GO:0005739)  [cytoskeleton](http://amigo.geneontology.org/cgi-bin/amigo/go.cgi?view=details&query=GO:0005856)  [plasma membrane](http://amigo.geneontology.org/cgi-bin/amigo/go.cgi?view=details&query=GO:0005886) |  |
| [225541_at](https://www.affymetrix.com/LinkServlet?&probeset=225541_at) | RPL22L1 | ribosomal protein L22-like 1 | 3 | -170582666 | [BE274422](http://www.ncbi.nlm.nih.gov/entrez/query.fcgi?cmd=search&db=nucleotide&term=BE274422%5BACCN%5D&doptcmdl=GenBank) | [200916](http://www.ncbi.nlm.nih.gov/sites/entrez?Db=gene&Cmd=DetailsSearch&Term=200916) | [3q26.2](http://www.ncbi.nlm.nih.gov/mapview/map_search.cgi?direct=on&idtype=gene&id=200916) | [Hs.380933](http://www.ncbi.nlm.nih.gov/UniGene/clust.cgi?ORG=Hs&CID=380933) | [5](http://www.ncbi.nih.gov/entrez/query.fcgi?tool=bioconductor&cmd=Retrieve&db=PubMed&list_uids=8076819%2C12477932%2C15489334%2C19615732%2C20379614) | [structural constituent of ribosome](http://amigo.geneontology.org/cgi-bin/amigo/go.cgi?view=details&query=GO:0003735)  [intracellular](http://amigo.geneontology.org/cgi-bin/amigo/go.cgi?view=details&query=GO:0005622)  [ribosome](http://amigo.geneontology.org/cgi-bin/amigo/go.cgi?view=details&query=GO:0005840)  [translation](http://amigo.geneontology.org/cgi-bin/amigo/go.cgi?view=details&query=GO:0006412) | [Ribosome](http://www.genome.ad.jp/kegg/pathway/hsa/hsa03010.html) |
| [226027_at](https://www.affymetrix.com/LinkServlet?&probeset=226027_at) | C9orf119 | chromosome 9 open reading frame 119 | 9 | 131038424 | [BG541668](http://www.ncbi.nlm.nih.gov/entrez/query.fcgi?cmd=search&db=nucleotide&term=BG541668%5BACCN%5D&doptcmdl=GenBank) | [375757](http://www.ncbi.nlm.nih.gov/sites/entrez?Db=gene&Cmd=DetailsSearch&Term=375757) | [9q34.11](http://www.ncbi.nlm.nih.gov/mapview/map_search.cgi?direct=on&idtype=gene&id=375757) | [Hs.259594](http://www.ncbi.nlm.nih.gov/UniGene/clust.cgi?ORG=Hs&CID=259594) | [2](http://www.ncbi.nih.gov/entrez/query.fcgi?tool=bioconductor&cmd=Retrieve&db=PubMed&list_uids=12477932%2C21252223) | [double-strand break repair via homologous recombination](http://amigo.geneontology.org/cgi-bin/amigo/go.cgi?view=details&query=GO:0000724)  [protein binding](http://amigo.geneontology.org/cgi-bin/amigo/go.cgi?view=details&query=GO:0005515)  [nucleus](http://amigo.geneontology.org/cgi-bin/amigo/go.cgi?view=details&query=GO:0005634)  [Swi5-Sfr1 complex](http://amigo.geneontology.org/cgi-bin/amigo/go.cgi?view=details&query=GO:0032798) |  |
| [226245_at](https://www.affymetrix.com/LinkServlet?&probeset=226245_at) | KCTD1 | potassium channel tetramerisation domain containing 1 | 18 | -24034876, -24034876, -24034876 | [AA199881](http://www.ncbi.nlm.nih.gov/entrez/query.fcgi?cmd=search&db=nucleotide&term=AA199881%5BACCN%5D&doptcmdl=GenBank) | [284252](http://www.ncbi.nlm.nih.gov/sites/entrez?Db=gene&Cmd=DetailsSearch&Term=284252) | [18q11.2](http://www.ncbi.nlm.nih.gov/mapview/map_search.cgi?direct=on&idtype=gene&id=284252) | [Hs.526630](http://www.ncbi.nlm.nih.gov/UniGene/clust.cgi?ORG=Hs&CID=526630) | [9](http://www.ncbi.nih.gov/entrez/query.fcgi?tool=bioconductor&cmd=Retrieve&db=PubMed&list_uids=11181995%2C12477932%2C15146197%2C15345747%2C15489334%2C15561718%2C17207965%2C19115315%2C20379614) | [voltage-gated potassium channel activity](http://amigo.geneontology.org/cgi-bin/amigo/go.cgi?view=details&query=GO:0005249)  [nucleus](http://amigo.geneontology.org/cgi-bin/amigo/go.cgi?view=details&query=GO:0005634)  [potassium ion transport](http://amigo.geneontology.org/cgi-bin/amigo/go.cgi?view=details&query=GO:0006813)  [voltage-gated potassium channel complex](http://amigo.geneontology.org/cgi-bin/amigo/go.cgi?view=details&query=GO:0008076)  [membrane](http://amigo.geneontology.org/cgi-bin/amigo/go.cgi?view=details&query=GO:0016020)  [negative regulation of transcription](http://amigo.geneontology.org/cgi-bin/amigo/go.cgi?view=details&query=GO:0016481) |  |
| [226246_at](https://www.affymetrix.com/LinkServlet?&probeset=226246_at) | KCTD1 | potassium channel tetramerisation domain containing 1 | 18 | -24034876, -24034876, -24034876 | [AA115278](http://www.ncbi.nlm.nih.gov/entrez/query.fcgi?cmd=search&db=nucleotide&term=AA115278%5BACCN%5D&doptcmdl=GenBank) | [284252](http://www.ncbi.nlm.nih.gov/sites/entrez?Db=gene&Cmd=DetailsSearch&Term=284252) | [18q11.2](http://www.ncbi.nlm.nih.gov/mapview/map_search.cgi?direct=on&idtype=gene&id=284252) | [Hs.526630](http://www.ncbi.nlm.nih.gov/UniGene/clust.cgi?ORG=Hs&CID=526630) | [9](http://www.ncbi.nih.gov/entrez/query.fcgi?tool=bioconductor&cmd=Retrieve&db=PubMed&list_uids=11181995%2C12477932%2C15146197%2C15345747%2C15489334%2C15561718%2C17207965%2C19115315%2C20379614) | [voltage-gated potassium channel activity](http://amigo.geneontology.org/cgi-bin/amigo/go.cgi?view=details&query=GO:0005249)  [nucleus](http://amigo.geneontology.org/cgi-bin/amigo/go.cgi?view=details&query=GO:0005634)  [potassium ion transport](http://amigo.geneontology.org/cgi-bin/amigo/go.cgi?view=details&query=GO:0006813)  [voltage-gated potassium channel complex](http://amigo.geneontology.org/cgi-bin/amigo/go.cgi?view=details&query=GO:0008076)  [membrane](http://amigo.geneontology.org/cgi-bin/amigo/go.cgi?view=details&query=GO:0016020)  [negative regulation of transcription](http://amigo.geneontology.org/cgi-bin/amigo/go.cgi?view=details&query=GO:0016481) |  |
| [226324_s_at](https://www.affymetrix.com/LinkServlet?&probeset=226324_s_at) | IFT172 | intraflagellar transport 172 homolog (Chlamydomonas) | 2 | -27667240 | [AB033005](http://www.ncbi.nlm.nih.gov/entrez/query.fcgi?cmd=search&db=nucleotide&term=AB033005%5BACCN%5D&doptcmdl=GenBank) | [26160](http://www.ncbi.nlm.nih.gov/sites/entrez?Db=gene&Cmd=DetailsSearch&Term=26160) | [2p23.3](http://www.ncbi.nlm.nih.gov/mapview/map_search.cgi?direct=on&idtype=gene&id=26160) | [Hs.127401](http://www.ncbi.nlm.nih.gov/UniGene/clust.cgi?ORG=Hs&CID=127401) | [7](http://www.ncbi.nih.gov/entrez/query.fcgi?tool=bioconductor&cmd=Retrieve&db=PubMed&list_uids=10574461%2C10788441%2C12477932%2C14702039%2C20379614%2C20383146%2C20705733) | [neural tube formation](http://amigo.geneontology.org/cgi-bin/amigo/go.cgi?view=details&query=GO:0001841)  [binding](http://amigo.geneontology.org/cgi-bin/amigo/go.cgi?view=details&query=GO:0005488)  [cilium](http://amigo.geneontology.org/cgi-bin/amigo/go.cgi?view=details&query=GO:0005929)  [smoothened signaling pathway](http://amigo.geneontology.org/cgi-bin/amigo/go.cgi?view=details&query=GO:0007224)  [multicellular organismal development](http://amigo.geneontology.org/cgi-bin/amigo/go.cgi?view=details&query=GO:0007275)  [determination of left/right symmetry](http://amigo.geneontology.org/cgi-bin/amigo/go.cgi?view=details&query=GO:0007368)  [dorsal/ventral pattern formation](http://amigo.geneontology.org/cgi-bin/amigo/go.cgi?view=details&query=GO:0009953)  [protein processing](http://amigo.geneontology.org/cgi-bin/amigo/go.cgi?view=details&query=GO:0016485)  [cilium assembly](http://amigo.geneontology.org/cgi-bin/amigo/go.cgi?view=details&query=GO:0042384) |  |
| [226433_at](https://www.affymetrix.com/LinkServlet?&probeset=226433_at) | RNF157 | ring finger protein 157 | 17 | -74138534 | [BF056204](http://www.ncbi.nlm.nih.gov/entrez/query.fcgi?cmd=search&db=nucleotide&term=BF056204%5BACCN%5D&doptcmdl=GenBank) | [114804](http://www.ncbi.nlm.nih.gov/sites/entrez?Db=gene&Cmd=DetailsSearch&Term=114804) | [17q25.1](http://www.ncbi.nlm.nih.gov/mapview/map_search.cgi?direct=on&idtype=gene&id=114804) | [Hs.500643](http://www.ncbi.nlm.nih.gov/UniGene/clust.cgi?ORG=Hs&CID=500643) | [8](http://www.ncbi.nih.gov/entrez/query.fcgi?tool=bioconductor&cmd=Retrieve&db=PubMed&list_uids=11572484%2C12477932%2C14702039%2C19690564%2C19913121%2C20379614%2C20628086%2C20800603) | [zinc ion binding](http://amigo.geneontology.org/cgi-bin/amigo/go.cgi?view=details&query=GO:0008270)  [metal ion binding](http://amigo.geneontology.org/cgi-bin/amigo/go.cgi?view=details&query=GO:0046872) |  |
| [226571_s_at](https://www.affymetrix.com/LinkServlet?&probeset=226571_s_at) | PTPRS | protein tyrosine phosphatase, receptor type, S | 19 | -5205519 | [N38920](http://www.ncbi.nlm.nih.gov/entrez/query.fcgi?cmd=search&db=nucleotide&term=N38920%5BACCN%5D&doptcmdl=GenBank) | [5802](http://www.ncbi.nlm.nih.gov/sites/entrez?Db=gene&Cmd=DetailsSearch&Term=5802) | [19p13.3](http://www.ncbi.nlm.nih.gov/mapview/map_search.cgi?direct=on&idtype=gene&id=5802) | [Hs.728875](http://www.ncbi.nlm.nih.gov/UniGene/clust.cgi?ORG=Hs&CID=728875) | [26](http://www.ncbi.nih.gov/entrez/query.fcgi?tool=bioconductor&cmd=Retrieve&db=PubMed&list_uids=1370651%2C8524829%2C8954782%2C8992885%2C9357975%2C9566880%2C9624153%2C9790732%2C10080191%2C10080192%2C10435588%2C10777529%2C12376545%2C12477932%2C15057824%2C15146197%2C16169070%2C16273344%2C16335952%2C16552719%2C17353931%2C17614280%2C17893260%2C19000305%2C20139422%2C20179269) | [receptor activity](http://amigo.geneontology.org/cgi-bin/amigo/go.cgi?view=details&query=GO:0004872)  [transmembrane receptor protein tyrosine phosphatase activity](http://amigo.geneontology.org/cgi-bin/amigo/go.cgi?view=details&query=GO:0005001)  [protein binding](http://amigo.geneontology.org/cgi-bin/amigo/go.cgi?view=details&query=GO:0005515)  [integral to plasma membrane](http://amigo.geneontology.org/cgi-bin/amigo/go.cgi?view=details&query=GO:0005887)  [protein dephosphorylation](http://amigo.geneontology.org/cgi-bin/amigo/go.cgi?view=details&query=GO:0006470)  [cell adhesion](http://amigo.geneontology.org/cgi-bin/amigo/go.cgi?view=details&query=GO:0007155)  [membrane](http://amigo.geneontology.org/cgi-bin/amigo/go.cgi?view=details&query=GO:0016020)  [hydrolase activity](http://amigo.geneontology.org/cgi-bin/amigo/go.cgi?view=details&query=GO:0016787) |  |
| [226586_at](https://www.affymetrix.com/LinkServlet?&probeset=226586_at) | ANKS6 | ankyrin repeat and sterile alpha motif domain containing 6 | 9 | -101494291 | [AW130559](http://www.ncbi.nlm.nih.gov/entrez/query.fcgi?cmd=search&db=nucleotide&term=AW130559%5BACCN%5D&doptcmdl=GenBank) | [203286](http://www.ncbi.nlm.nih.gov/sites/entrez?Db=gene&Cmd=DetailsSearch&Term=203286) | [9q22.33](http://www.ncbi.nlm.nih.gov/mapview/map_search.cgi?direct=on&idtype=gene&id=203286) | [Hs.406890](http://www.ncbi.nlm.nih.gov/UniGene/clust.cgi?ORG=Hs&CID=406890) | [7](http://www.ncbi.nih.gov/entrez/query.fcgi?tool=bioconductor&cmd=Retrieve&db=PubMed&list_uids=12477932%2C14702039%2C15164053%2C15489334%2C18029348%2C18434273%2C18978678) | [cytoplasm](http://amigo.geneontology.org/cgi-bin/amigo/go.cgi?view=details&query=GO:0005737)  [protein homodimerization activity](http://amigo.geneontology.org/cgi-bin/amigo/go.cgi?view=details&query=GO:0042803) |  |
| [226609_at](https://www.affymetrix.com/LinkServlet?&probeset=226609_at) | DCBLD1 | discoidin, CUB and LCCL domain containing 1 | 6 | 117803819 | [N22751](http://www.ncbi.nlm.nih.gov/entrez/query.fcgi?cmd=search&db=nucleotide&term=N22751%5BACCN%5D&doptcmdl=GenBank) | [285761](http://www.ncbi.nlm.nih.gov/sites/entrez?Db=gene&Cmd=DetailsSearch&Term=285761) | [6q22.1](http://www.ncbi.nlm.nih.gov/mapview/map_search.cgi?direct=on&idtype=gene&id=285761) | [Hs.658304](http://www.ncbi.nlm.nih.gov/UniGene/clust.cgi?ORG=Hs&CID=658304) | [5](http://www.ncbi.nih.gov/entrez/query.fcgi?tool=bioconductor&cmd=Retrieve&db=PubMed&list_uids=12477932%2C14574404%2C14702039%2C15489334%2C20379614) | [cell adhesion](http://amigo.geneontology.org/cgi-bin/amigo/go.cgi?view=details&query=GO:0007155)  [membrane](http://amigo.geneontology.org/cgi-bin/amigo/go.cgi?view=details&query=GO:0016020)  [integral to membrane](http://amigo.geneontology.org/cgi-bin/amigo/go.cgi?view=details&query=GO:0016021) |  |
| [226641_at](https://www.affymetrix.com/LinkServlet?&probeset=226641_at) | ANKRD44 | ankyrin repeat domain 44 | 2 | -197859249 | [AU157224](http://www.ncbi.nlm.nih.gov/entrez/query.fcgi?cmd=search&db=nucleotide&term=AU157224%5BACCN%5D&doptcmdl=GenBank) | [91526](http://www.ncbi.nlm.nih.gov/sites/entrez?Db=gene&Cmd=DetailsSearch&Term=91526) | [2q33.1](http://www.ncbi.nlm.nih.gov/mapview/map_search.cgi?direct=on&idtype=gene&id=91526) | [Hs.432706](http://www.ncbi.nlm.nih.gov/UniGene/clust.cgi?ORG=Hs&CID=432706) | [8](http://www.ncbi.nih.gov/entrez/query.fcgi?tool=bioconductor&cmd=Retrieve&db=PubMed&list_uids=12477932%2C14702039%2C15489334%2C16555005%2C18186651%2C19240061%2C19596235%2C20379614) | [protein binding](http://amigo.geneontology.org/cgi-bin/amigo/go.cgi?view=details&query=GO:0005515) |  |
| [226653_at](https://www.affymetrix.com/LinkServlet?&probeset=226653_at) | MARK1 | MAP/microtubule affinity-regulating kinase 1 | 1 | 220701567 | [AB040910](http://www.ncbi.nlm.nih.gov/entrez/query.fcgi?cmd=search&db=nucleotide&term=AB040910%5BACCN%5D&doptcmdl=GenBank) | [4139](http://www.ncbi.nlm.nih.gov/sites/entrez?Db=gene&Cmd=DetailsSearch&Term=4139) | [1q41](http://www.ncbi.nlm.nih.gov/mapview/map_search.cgi?direct=on&idtype=gene&id=4139) | [Hs.497806](http://www.ncbi.nlm.nih.gov/UniGene/clust.cgi?ORG=Hs&CID=497806) | [30](http://www.ncbi.nih.gov/entrez/query.fcgi?tool=bioconductor&cmd=Retrieve&db=PubMed&list_uids=7706316%2C7931292%2C8631898%2C8999860%2C9108484%2C9735171%2C9771888%2C9832145%2C10090741%2C10737616%2C10819331%2C12387894%2C12435421%2C12477932%2C14517247%2C14594945%2C14702039%2C14976552%2C15489334%2C15778465%2C16344560%2C16710414%2C16803889%2C17192257%2C18254724%2C18492799%2C19090997%2C19615732%2C20158304%2C20379614) | [nucleotide binding](http://amigo.geneontology.org/cgi-bin/amigo/go.cgi?view=details&query=GO:0000166)  [microtubule cytoskeleton organization](http://amigo.geneontology.org/cgi-bin/amigo/go.cgi?view=details&query=GO:0000226)  [magnesium ion binding](http://amigo.geneontology.org/cgi-bin/amigo/go.cgi?view=details&query=GO:0000287)  [protein serine/threonine kinase activity](http://amigo.geneontology.org/cgi-bin/amigo/go.cgi?view=details&query=GO:0004674)  [ATP binding](http://amigo.geneontology.org/cgi-bin/amigo/go.cgi?view=details&query=GO:0005524)  [cytoplasm](http://amigo.geneontology.org/cgi-bin/amigo/go.cgi?view=details&query=GO:0005737)  [cytoskeleton](http://amigo.geneontology.org/cgi-bin/amigo/go.cgi?view=details&query=GO:0005856)  [protein phosphorylation](http://amigo.geneontology.org/cgi-bin/amigo/go.cgi?view=details&query=GO:0006468)  [cytoskeleton organization](http://amigo.geneontology.org/cgi-bin/amigo/go.cgi?view=details&query=GO:0007010)  [intracellular protein kinase cascade](http://amigo.geneontology.org/cgi-bin/amigo/go.cgi?view=details&query=GO:0007243)  [microtubule cytoskeleton](http://amigo.geneontology.org/cgi-bin/amigo/go.cgi?view=details&query=GO:0015630)  [transferase activity](http://amigo.geneontology.org/cgi-bin/amigo/go.cgi?view=details&query=GO:0016740) |  |
| [226733_at](https://www.affymetrix.com/LinkServlet?&probeset=226733_at) | PFKFB2 | 6-phosphofructo-2-kinase/fructose-2,6-biphosphatase 2 | 1 | 207226619, 207226619 | [AA587884](http://www.ncbi.nlm.nih.gov/entrez/query.fcgi?cmd=search&db=nucleotide&term=AA587884%5BACCN%5D&doptcmdl=GenBank) | [5208](http://www.ncbi.nlm.nih.gov/sites/entrez?Db=gene&Cmd=DetailsSearch&Term=5208) | [1q31](http://www.ncbi.nlm.nih.gov/mapview/map_search.cgi?direct=on&idtype=gene&id=5208) | [Hs.282702](http://www.ncbi.nlm.nih.gov/UniGene/clust.cgi?ORG=Hs&CID=282702) | [24](http://www.ncbi.nih.gov/entrez/query.fcgi?tool=bioconductor&cmd=Retrieve&db=PubMed&list_uids=1322130%2C1655632%2C7574501%2C7904197%2C9464277%2C9652401%2C11129574%2C11245921%2C11374908%2C11522786%2C12065600%2C12477932%2C12853467%2C14702039%2C15324660%2C15489334%2C15925437%2C16025159%2C16710414%2C18039179%2C19423540%2C20406964%2C20438785%2C20958264) | [nucleotide binding](http://amigo.geneontology.org/cgi-bin/amigo/go.cgi?view=details&query=GO:0000166)  [6-phosphofructo-2-kinase activity](http://amigo.geneontology.org/cgi-bin/amigo/go.cgi?view=details&query=GO:0003873)  [fructose-2,6-bisphosphate 2-phosphatase activity](http://amigo.geneontology.org/cgi-bin/amigo/go.cgi?view=details&query=GO:0004331)  [ATP binding](http://amigo.geneontology.org/cgi-bin/amigo/go.cgi?view=details&query=GO:0005524)  [cytoplasm](http://amigo.geneontology.org/cgi-bin/amigo/go.cgi?view=details&query=GO:0005737)  [cytosol](http://amigo.geneontology.org/cgi-bin/amigo/go.cgi?view=details&query=GO:0005829)  [carbohydrate metabolic process](http://amigo.geneontology.org/cgi-bin/amigo/go.cgi?view=details&query=GO:0005975)  [fructose metabolic process](http://amigo.geneontology.org/cgi-bin/amigo/go.cgi?view=details&query=GO:0006000)  [fructose 2,6-bisphosphate metabolic process](http://amigo.geneontology.org/cgi-bin/amigo/go.cgi?view=details&query=GO:0006003)  [glucose metabolic process](http://amigo.geneontology.org/cgi-bin/amigo/go.cgi?view=details&query=GO:0006006)  [lactate metabolic process](http://amigo.geneontology.org/cgi-bin/amigo/go.cgi?view=details&query=GO:0006089)  [pyruvate metabolic process](http://amigo.geneontology.org/cgi-bin/amigo/go.cgi?view=details&query=GO:0006090)  [glycolysis](http://amigo.geneontology.org/cgi-bin/amigo/go.cgi?view=details&query=GO:0006096)  [response to glucose stimulus](http://amigo.geneontology.org/cgi-bin/amigo/go.cgi?view=details&query=GO:0009749)  [kinase activity](http://amigo.geneontology.org/cgi-bin/amigo/go.cgi?view=details&query=GO:0016301)  [transferase activity](http://amigo.geneontology.org/cgi-bin/amigo/go.cgi?view=details&query=GO:0016740)  [hydrolase activity](http://amigo.geneontology.org/cgi-bin/amigo/go.cgi?view=details&query=GO:0016787)  [kinase binding](http://amigo.geneontology.org/cgi-bin/amigo/go.cgi?view=details&query=GO:0019900)  [positive regulation of insulin secretion](http://amigo.geneontology.org/cgi-bin/amigo/go.cgi?view=details&query=GO:0032024)  [positive regulation of glucokinase activity](http://amigo.geneontology.org/cgi-bin/amigo/go.cgi?view=details&query=GO:0033133) | [Fructose and mannose metabolism](http://www.genome.ad.jp/dbget-bin/show_pathway?MAP00051+2.7.1.105) |
| [226837_at](https://www.affymetrix.com/LinkServlet?&probeset=226837_at) | SPRED1 | sprouty-related, EVH1 domain containing 1 | 15 | 38545051 | [BE967019](http://www.ncbi.nlm.nih.gov/entrez/query.fcgi?cmd=search&db=nucleotide&term=BE967019%5BACCN%5D&doptcmdl=GenBank) | [161742](http://www.ncbi.nlm.nih.gov/sites/entrez?Db=gene&Cmd=DetailsSearch&Term=161742) | [15q14](http://www.ncbi.nlm.nih.gov/mapview/map_search.cgi?direct=on&idtype=gene&id=161742) | [Hs.525781](http://www.ncbi.nlm.nih.gov/UniGene/clust.cgi?ORG=Hs&CID=525781) | [23](http://www.ncbi.nih.gov/entrez/query.fcgi?tool=bioconductor&cmd=Retrieve&db=PubMed&list_uids=11493923%2C12477932%2C12646235%2C14702039%2C15231748%2C15465815%2C15683364%2C16115197%2C16344560%2C16652141%2C17094949%2C17672918%2C17704776%2C17974561%2C18216281%2C19120036%2C19366998%2C19443465%2C19913121%2C19920235%2C20339110%2C20628086%2C20945555) | [inactivation of MAPK activity](http://amigo.geneontology.org/cgi-bin/amigo/go.cgi?view=details&query=GO:0000188)  [stem cell factor receptor binding](http://amigo.geneontology.org/cgi-bin/amigo/go.cgi?view=details&query=GO:0005173)  [nucleus](http://amigo.geneontology.org/cgi-bin/amigo/go.cgi?view=details&query=GO:0005634)  [plasma membrane](http://amigo.geneontology.org/cgi-bin/amigo/go.cgi?view=details&query=GO:0005886)  [caveola](http://amigo.geneontology.org/cgi-bin/amigo/go.cgi?view=details&query=GO:0005901)  [multicellular organismal development](http://amigo.geneontology.org/cgi-bin/amigo/go.cgi?view=details&query=GO:0007275)  [regulation of signal transduction](http://amigo.geneontology.org/cgi-bin/amigo/go.cgi?view=details&query=GO:0009966) | [Jak-STAT signaling pathway](http://www.genome.ad.jp/kegg/pathway/hsa/hsa04630.html) |
| [226889_at](https://www.affymetrix.com/LinkServlet?&probeset=226889_at) | WDR35 | WD repeat domain 35 | 2 | -20110030 | [AU151732](http://www.ncbi.nlm.nih.gov/entrez/query.fcgi?cmd=search&db=nucleotide&term=AU151732%5BACCN%5D&doptcmdl=GenBank) | [57539](http://www.ncbi.nlm.nih.gov/sites/entrez?Db=gene&Cmd=DetailsSearch&Term=57539) | [2p24.1](http://www.ncbi.nlm.nih.gov/mapview/map_search.cgi?direct=on&idtype=gene&id=57539) | [Hs.205427](http://www.ncbi.nlm.nih.gov/UniGene/clust.cgi?ORG=Hs&CID=205427) | [7](http://www.ncbi.nih.gov/entrez/query.fcgi?tool=bioconductor&cmd=Retrieve&db=PubMed&list_uids=10718198%2C12168954%2C12477932%2C14702039%2C15489334%2C20193664%2C20817137) |  |  |
| [226989_at](https://www.affymetrix.com/LinkServlet?&probeset=226989_at) | RGMB | RGM domain family, member B | 5 | 98104998 | [BE855765](http://www.ncbi.nlm.nih.gov/entrez/query.fcgi?cmd=search&db=nucleotide&term=BE855765%5BACCN%5D&doptcmdl=GenBank) | [285704](http://www.ncbi.nlm.nih.gov/sites/entrez?Db=gene&Cmd=DetailsSearch&Term=285704) | [5q15](http://www.ncbi.nlm.nih.gov/mapview/map_search.cgi?direct=on&idtype=gene&id=285704) | [Hs.526902](http://www.ncbi.nlm.nih.gov/UniGene/clust.cgi?ORG=Hs&CID=526902) | [6](http://www.ncbi.nih.gov/entrez/query.fcgi?tool=bioconductor&cmd=Retrieve&db=PubMed&list_uids=12477932%2C14702039%2C14985445%2C15489334%2C15671031%2C21182207) | [ER-Golgi intermediate compartment](http://amigo.geneontology.org/cgi-bin/amigo/go.cgi?view=details&query=GO:0005793)  [plasma membrane](http://amigo.geneontology.org/cgi-bin/amigo/go.cgi?view=details&query=GO:0005886)  [cell adhesion](http://amigo.geneontology.org/cgi-bin/amigo/go.cgi?view=details&query=GO:0007155)  [signal transduction](http://amigo.geneontology.org/cgi-bin/amigo/go.cgi?view=details&query=GO:0007165)  [axon guidance](http://amigo.geneontology.org/cgi-bin/amigo/go.cgi?view=details&query=GO:0007411)  [BMP signaling pathway](http://amigo.geneontology.org/cgi-bin/amigo/go.cgi?view=details&query=GO:0030509)  [identical protein binding](http://amigo.geneontology.org/cgi-bin/amigo/go.cgi?view=details&query=GO:0042802)  [membrane raft](http://amigo.geneontology.org/cgi-bin/amigo/go.cgi?view=details&query=GO:0045121)  [positive regulation of transcription](http://amigo.geneontology.org/cgi-bin/amigo/go.cgi?view=details&query=GO:0045941)  [anchored to plasma membrane](http://amigo.geneontology.org/cgi-bin/amigo/go.cgi?view=details&query=GO:0046658) |  |
| [227034_at](https://www.affymetrix.com/LinkServlet?&probeset=227034_at) | ANKRD57 | ankyrin repeat domain 57 | 2 | 110371910 | [BE669553](http://www.ncbi.nlm.nih.gov/entrez/query.fcgi?cmd=search&db=nucleotide&term=BE669553%5BACCN%5D&doptcmdl=GenBank) | [65124](http://www.ncbi.nlm.nih.gov/sites/entrez?Db=gene&Cmd=DetailsSearch&Term=65124) | [2q13](http://www.ncbi.nlm.nih.gov/mapview/map_search.cgi?direct=on&idtype=gene&id=65124) | [Hs.355455](http://www.ncbi.nlm.nih.gov/UniGene/clust.cgi?ORG=Hs&CID=355455) | [6](http://www.ncbi.nih.gov/entrez/query.fcgi?tool=bioconductor&cmd=Retrieve&db=PubMed&list_uids=12477932%2C14702039%2C15489334%2C15815621%2C16964243%2C20211142) |  |  |
| [227180_at](https://www.affymetrix.com/LinkServlet?&probeset=227180_at) | ELOVL7 | ELOVL family member 7, elongation of long chain fatty acids (yeast) | 5 | -60047616 | [AW138767](http://www.ncbi.nlm.nih.gov/entrez/query.fcgi?cmd=search&db=nucleotide&term=AW138767%5BACCN%5D&doptcmdl=GenBank) | [79993](http://www.ncbi.nlm.nih.gov/sites/entrez?Db=gene&Cmd=DetailsSearch&Term=79993) | [5q12.1](http://www.ncbi.nlm.nih.gov/mapview/map_search.cgi?direct=on&idtype=gene&id=79993) | [Hs.274256](http://www.ncbi.nlm.nih.gov/UniGene/clust.cgi?ORG=Hs&CID=274256) | [5](http://www.ncbi.nih.gov/entrez/query.fcgi?tool=bioconductor&cmd=Retrieve&db=PubMed&list_uids=8944226%2C12477932%2C16344560%2C19826053%2C20379614) | [protein binding](http://amigo.geneontology.org/cgi-bin/amigo/go.cgi?view=details&query=GO:0005515)  [endoplasmic reticulum](http://amigo.geneontology.org/cgi-bin/amigo/go.cgi?view=details&query=GO:0005783)  [endoplasmic reticulum membrane](http://amigo.geneontology.org/cgi-bin/amigo/go.cgi?view=details&query=GO:0005789)  [fatty acid elongase activity](http://amigo.geneontology.org/cgi-bin/amigo/go.cgi?view=details&query=GO:0009922)  [membrane](http://amigo.geneontology.org/cgi-bin/amigo/go.cgi?view=details&query=GO:0016020)  [integral to membrane](http://amigo.geneontology.org/cgi-bin/amigo/go.cgi?view=details&query=GO:0016021)  [transferase activity](http://amigo.geneontology.org/cgi-bin/amigo/go.cgi?view=details&query=GO:0016740)  [transferase activity, transferring acyl groups other than amino-acyl groups](http://amigo.geneontology.org/cgi-bin/amigo/go.cgi?view=details&query=GO:0016747)  [triglyceride biosynthetic process](http://amigo.geneontology.org/cgi-bin/amigo/go.cgi?view=details&query=GO:0019432)  [fatty acid elongation, polyunsaturated fatty acid](http://amigo.geneontology.org/cgi-bin/amigo/go.cgi?view=details&query=GO:0034626)  [long-chain fatty-acyl-CoA biosynthetic process](http://amigo.geneontology.org/cgi-bin/amigo/go.cgi?view=details&query=GO:0035338)  [very long-chain fatty acid biosynthetic process](http://amigo.geneontology.org/cgi-bin/amigo/go.cgi?view=details&query=GO:0042761)  [cellular lipid metabolic process](http://amigo.geneontology.org/cgi-bin/amigo/go.cgi?view=details&query=GO:0044255) |  |
| [227197_at](https://www.affymetrix.com/LinkServlet?&probeset=227197_at) | ARHGEF26 | Rho guanine nucleotide exchange factor (GEF) 26 | 3 | 153839148 | [AI989530](http://www.ncbi.nlm.nih.gov/entrez/query.fcgi?cmd=search&db=nucleotide&term=AI989530%5BACCN%5D&doptcmdl=GenBank) | [26084](http://www.ncbi.nlm.nih.gov/sites/entrez?Db=gene&Cmd=DetailsSearch&Term=26084) | [3q25.2](http://www.ncbi.nlm.nih.gov/mapview/map_search.cgi?direct=on&idtype=gene&id=26084) | [Hs.240845](http://www.ncbi.nlm.nih.gov/UniGene/clust.cgi?ORG=Hs&CID=240845) | [6](http://www.ncbi.nih.gov/entrez/query.fcgi?tool=bioconductor&cmd=Retrieve&db=PubMed&list_uids=12477932%2C12697679%2C14702039%2C15133129%2C15221005%2C16344560) |  | [Bacterial invasion of epithelial cells](http://www.genome.ad.jp/kegg/pathway/hsa/hsa05100.html) |
| [227273_at](https://www.affymetrix.com/LinkServlet?&probeset=227273_at) |  |  |  |  | [AI126798](http://www.ncbi.nlm.nih.gov/entrez/query.fcgi?cmd=search&db=nucleotide&term=AI126798%5BACCN%5D&doptcmdl=GenBank) |  |  |  |  |  |  |
| [227377_at](https://www.affymetrix.com/LinkServlet?&probeset=227377_at) | IGF2BP1 | insulin-like growth factor 2 mRNA binding protein 1 | 17 | 47074773 | [AK022784](http://www.ncbi.nlm.nih.gov/entrez/query.fcgi?cmd=search&db=nucleotide&term=AK022784%5BACCN%5D&doptcmdl=GenBank) | [10642](http://www.ncbi.nlm.nih.gov/sites/entrez?Db=gene&Cmd=DetailsSearch&Term=10642) | [17q21.32](http://www.ncbi.nlm.nih.gov/mapview/map_search.cgi?direct=on&idtype=gene&id=10642) | [Hs.144936](http://www.ncbi.nlm.nih.gov/UniGene/clust.cgi?ORG=Hs&CID=144936) | [52](http://www.ncbi.nih.gov/entrez/query.fcgi?tool=bioconductor&cmd=Retrieve&db=PubMed&list_uids=8132663%2C9801297%2C9891060%2C10850408%2C10875929%2C11973350%2C11992722%2C12024010%2C12532419%2C12921532%2C14702039%2C14767552%2C15121863%2C15159028%2C15282548%2C15314207%2C15342556%2C15355996%2C15601260%2C15769738%2C16049158%2C16306994%2C16344560%2C16356927%2C16541107%2C16778892%2C16964243%2C17081983%2C17101699%2C17212783%2C17255263%2C17289661%2C17296566%2C17353931%2C17546046%2C17643375%2C18029348%2C18252897%2C18385235%2C18454174%2C18490442%2C19029303%2C19038974%2C19541769%2C19647520%2C19661680%2C19726068%2C19887615%2C20195514%2C20308539%2C20627640%2C20819778) | [nucleotide binding](http://amigo.geneontology.org/cgi-bin/amigo/go.cgi?view=details&query=GO:0000166)  [mRNA binding](http://amigo.geneontology.org/cgi-bin/amigo/go.cgi?view=details&query=GO:0003729)  [mRNA 3'-UTR binding](http://amigo.geneontology.org/cgi-bin/amigo/go.cgi?view=details&query=GO:0003730)  [protein binding](http://amigo.geneontology.org/cgi-bin/amigo/go.cgi?view=details&query=GO:0005515)  [nucleus](http://amigo.geneontology.org/cgi-bin/amigo/go.cgi?view=details&query=GO:0005634)  [cytoplasm](http://amigo.geneontology.org/cgi-bin/amigo/go.cgi?view=details&query=GO:0005737)  [cytosol](http://amigo.geneontology.org/cgi-bin/amigo/go.cgi?view=details&query=GO:0005829)  [plasma membrane](http://amigo.geneontology.org/cgi-bin/amigo/go.cgi?view=details&query=GO:0005886)  [RNA localization](http://amigo.geneontology.org/cgi-bin/amigo/go.cgi?view=details&query=GO:0006403)  [stress granule](http://amigo.geneontology.org/cgi-bin/amigo/go.cgi?view=details&query=GO:0010494)  [regulation of mRNA stability involved in response to stress](http://amigo.geneontology.org/cgi-bin/amigo/go.cgi?view=details&query=GO:0010610)  [negative regulation of translation](http://amigo.geneontology.org/cgi-bin/amigo/go.cgi?view=details&query=GO:0017148)  [lamellipodium](http://amigo.geneontology.org/cgi-bin/amigo/go.cgi?view=details&query=GO:0030027)  [ribonucleoprotein complex](http://amigo.geneontology.org/cgi-bin/amigo/go.cgi?view=details&query=GO:0030529)  [regulation of cytokine biosynthetic process](http://amigo.geneontology.org/cgi-bin/amigo/go.cgi?view=details&query=GO:0042035)  [dendritic spine](http://amigo.geneontology.org/cgi-bin/amigo/go.cgi?view=details&query=GO:0043197)  [intracellular membrane-bounded organelle](http://amigo.geneontology.org/cgi-bin/amigo/go.cgi?view=details&query=GO:0043231)  [translation regulator activity](http://amigo.geneontology.org/cgi-bin/amigo/go.cgi?view=details&query=GO:0045182)  [mRNA 5'-UTR binding](http://amigo.geneontology.org/cgi-bin/amigo/go.cgi?view=details&query=GO:0048027)  [CRD-mediated mRNA stabilization](http://amigo.geneontology.org/cgi-bin/amigo/go.cgi?view=details&query=GO:0070934)  [CRD-mediated mRNA stability complex](http://amigo.geneontology.org/cgi-bin/amigo/go.cgi?view=details&query=GO:0070937) |  |
| [227424_x_at](https://www.affymetrix.com/LinkServlet?&probeset=227424_x_at) | C21orf119 | chromosome 21 open reading frame 119 | 21 | 33765441 | [AI800837](http://www.ncbi.nlm.nih.gov/entrez/query.fcgi?cmd=search&db=nucleotide&term=AI800837%5BACCN%5D&doptcmdl=GenBank) | [84996](http://www.ncbi.nlm.nih.gov/sites/entrez?Db=gene&Cmd=DetailsSearch&Term=84996) | [21q22.11](http://www.ncbi.nlm.nih.gov/mapview/map_search.cgi?direct=on&idtype=gene&id=84996) | [Hs.58149](http://www.ncbi.nlm.nih.gov/UniGene/clust.cgi?ORG=Hs&CID=58149) | [2](http://www.ncbi.nih.gov/entrez/query.fcgi?tool=bioconductor&cmd=Retrieve&db=PubMed&list_uids=12477932%2C15489334) |  |  |
| [227444_at](https://www.affymetrix.com/LinkServlet?&probeset=227444_at) | ARMCX4 | armadillo repeat containing, X-linked 4 | X | 100673265 | [AW519141](http://www.ncbi.nlm.nih.gov/entrez/query.fcgi?cmd=search&db=nucleotide&term=AW519141%5BACCN%5D&doptcmdl=GenBank) | [100131755](http://www.ncbi.nlm.nih.gov/sites/entrez?Db=gene&Cmd=DetailsSearch&Term=100131755) | [Xq22.1](http://www.ncbi.nlm.nih.gov/mapview/map_search.cgi?direct=on&idtype=gene&id=100131755) | [Hs.729062](http://www.ncbi.nlm.nih.gov/UniGene/clust.cgi?ORG=Hs&CID=729062) | [6](http://www.ncbi.nih.gov/entrez/query.fcgi?tool=bioconductor&cmd=Retrieve&db=PubMed&list_uids=11181995%2C12477932%2C14702039%2C15772651%2C16344560%2C17974005) | [membrane](http://amigo.geneontology.org/cgi-bin/amigo/go.cgi?view=details&query=GO:0016020)  [integral to membrane](http://amigo.geneontology.org/cgi-bin/amigo/go.cgi?view=details&query=GO:0016021) |  |
| [227875_at](https://www.affymetrix.com/LinkServlet?&probeset=227875_at) | KLHL13 | kelch-like 13 (Drosophila) | X | -117031776, -117031776, -117031776, -117031776, -117031776 | [AB037730](http://www.ncbi.nlm.nih.gov/entrez/query.fcgi?cmd=search&db=nucleotide&term=AB037730%5BACCN%5D&doptcmdl=GenBank) | [90293](http://www.ncbi.nlm.nih.gov/sites/entrez?Db=gene&Cmd=DetailsSearch&Term=90293) | [Xq23-q24](http://www.ncbi.nlm.nih.gov/mapview/map_search.cgi?direct=on&idtype=gene&id=90293) | [Hs.348262](http://www.ncbi.nlm.nih.gov/UniGene/clust.cgi?ORG=Hs&CID=348262) | [11](http://www.ncbi.nih.gov/entrez/query.fcgi?tool=bioconductor&cmd=Retrieve&db=PubMed&list_uids=10718198%2C12477932%2C14702039%2C15342556%2C15489334%2C15772651%2C16344560%2C17543862%2C18075312%2C19084217%2C19615732) | [cytokinesis](http://amigo.geneontology.org/cgi-bin/amigo/go.cgi?view=details&query=GO:0000910)  [ubiquitin-protein ligase activity](http://amigo.geneontology.org/cgi-bin/amigo/go.cgi?view=details&query=GO:0004842)  [cell cycle](http://amigo.geneontology.org/cgi-bin/amigo/go.cgi?view=details&query=GO:0007049)  [mitosis](http://amigo.geneontology.org/cgi-bin/amigo/go.cgi?view=details&query=GO:0007067)  [protein ubiquitination](http://amigo.geneontology.org/cgi-bin/amigo/go.cgi?view=details&query=GO:0016567)  [Cul3-RING ubiquitin ligase complex](http://amigo.geneontology.org/cgi-bin/amigo/go.cgi?view=details&query=GO:0031463) | [Ubiquitin mediated proteolysis](http://www.genome.ad.jp/kegg/pathway/hsa/hsa04120.html) |
| [227966_s_at](https://www.affymetrix.com/LinkServlet?&probeset=227966_s_at) |  |  |  |  | [AA524895](http://www.ncbi.nlm.nih.gov/entrez/query.fcgi?cmd=search&db=nucleotide&term=AA524895%5BACCN%5D&doptcmdl=GenBank) |  |  |  |  |  |  |
| [228208_x_at](https://www.affymetrix.com/LinkServlet?&probeset=228208_x_at) | ZNF354C | zinc finger protein 354C | 5 | 178487606 | [AL134573](http://www.ncbi.nlm.nih.gov/entrez/query.fcgi?cmd=search&db=nucleotide&term=AL134573%5BACCN%5D&doptcmdl=GenBank) | [30832](http://www.ncbi.nlm.nih.gov/sites/entrez?Db=gene&Cmd=DetailsSearch&Term=30832) | [5q35](http://www.ncbi.nlm.nih.gov/mapview/map_search.cgi?direct=on&idtype=gene&id=30832) | [Hs.272328](http://www.ncbi.nlm.nih.gov/UniGene/clust.cgi?ORG=Hs&CID=272328) | [4](http://www.ncbi.nih.gov/entrez/query.fcgi?tool=bioconductor&cmd=Retrieve&db=PubMed&list_uids=10786630%2C12477932%2C15489334%2C15555547) | [DNA binding](http://amigo.geneontology.org/cgi-bin/amigo/go.cgi?view=details&query=GO:0003677)  [intracellular](http://amigo.geneontology.org/cgi-bin/amigo/go.cgi?view=details&query=GO:0005622)  [nucleus](http://amigo.geneontology.org/cgi-bin/amigo/go.cgi?view=details&query=GO:0005634)  [regulation of transcription, DNA-dependent](http://amigo.geneontology.org/cgi-bin/amigo/go.cgi?view=details&query=GO:0006355)  [zinc ion binding](http://amigo.geneontology.org/cgi-bin/amigo/go.cgi?view=details&query=GO:0008270)  [metal ion binding](http://amigo.geneontology.org/cgi-bin/amigo/go.cgi?view=details&query=GO:0046872) |  |
| [228240_at](https://www.affymetrix.com/LinkServlet?&probeset=228240_at) |  |  |  |  | [AW952320](http://www.ncbi.nlm.nih.gov/entrez/query.fcgi?cmd=search&db=nucleotide&term=AW952320%5BACCN%5D&doptcmdl=GenBank) |  |  |  |  |  |  |
| [228353_x_at](https://www.affymetrix.com/LinkServlet?&probeset=228353_x_at) | UBASH3B | ubiquitin associated and SH3 domain containing B | 11 | 122526397 | [AA233308](http://www.ncbi.nlm.nih.gov/entrez/query.fcgi?cmd=search&db=nucleotide&term=AA233308%5BACCN%5D&doptcmdl=GenBank) | [84959](http://www.ncbi.nlm.nih.gov/sites/entrez?Db=gene&Cmd=DetailsSearch&Term=84959) | [11q24.1](http://www.ncbi.nlm.nih.gov/mapview/map_search.cgi?direct=on&idtype=gene&id=84959) | [Hs.444075](http://www.ncbi.nlm.nih.gov/UniGene/clust.cgi?ORG=Hs&CID=444075) | [18](http://www.ncbi.nih.gov/entrez/query.fcgi?tool=bioconductor&cmd=Retrieve&db=PubMed&list_uids=8125298%2C8889548%2C9373149%2C11853319%2C12370296%2C12477932%2C12665801%2C14702039%2C15159412%2C15388581%2C15489334%2C16303743%2C16344560%2C16429130%2C17588522%2C18189269%2C20379614%2C20585042) | [protein tyrosine phosphatase activity](http://amigo.geneontology.org/cgi-bin/amigo/go.cgi?view=details&query=GO:0004725)  [nucleus](http://amigo.geneontology.org/cgi-bin/amigo/go.cgi?view=details&query=GO:0005634)  [cytoplasm](http://amigo.geneontology.org/cgi-bin/amigo/go.cgi?view=details&query=GO:0005737)  [hydrolase activity](http://amigo.geneontology.org/cgi-bin/amigo/go.cgi?view=details&query=GO:0016787) |  |
| [228359_at](https://www.affymetrix.com/LinkServlet?&probeset=228359_at) | UBASH3B | ubiquitin associated and SH3 domain containing B | 11 | 122526397 | [AA037664](http://www.ncbi.nlm.nih.gov/entrez/query.fcgi?cmd=search&db=nucleotide&term=AA037664%5BACCN%5D&doptcmdl=GenBank) | [84959](http://www.ncbi.nlm.nih.gov/sites/entrez?Db=gene&Cmd=DetailsSearch&Term=84959) | [11q24.1](http://www.ncbi.nlm.nih.gov/mapview/map_search.cgi?direct=on&idtype=gene&id=84959) | [Hs.444075](http://www.ncbi.nlm.nih.gov/UniGene/clust.cgi?ORG=Hs&CID=444075) | [18](http://www.ncbi.nih.gov/entrez/query.fcgi?tool=bioconductor&cmd=Retrieve&db=PubMed&list_uids=8125298%2C8889548%2C9373149%2C11853319%2C12370296%2C12477932%2C12665801%2C14702039%2C15159412%2C15388581%2C15489334%2C16303743%2C16344560%2C16429130%2C17588522%2C18189269%2C20379614%2C20585042) | [protein tyrosine phosphatase activity](http://amigo.geneontology.org/cgi-bin/amigo/go.cgi?view=details&query=GO:0004725)  [nucleus](http://amigo.geneontology.org/cgi-bin/amigo/go.cgi?view=details&query=GO:0005634)  [cytoplasm](http://amigo.geneontology.org/cgi-bin/amigo/go.cgi?view=details&query=GO:0005737)  [hydrolase activity](http://amigo.geneontology.org/cgi-bin/amigo/go.cgi?view=details&query=GO:0016787) |  |
| [228476_at](https://www.affymetrix.com/LinkServlet?&probeset=228476_at) | KIAA1407 | KIAA1407 | 3 | -113682985 | [AW193515](http://www.ncbi.nlm.nih.gov/entrez/query.fcgi?cmd=search&db=nucleotide&term=AW193515%5BACCN%5D&doptcmdl=GenBank) | [57577](http://www.ncbi.nlm.nih.gov/sites/entrez?Db=gene&Cmd=DetailsSearch&Term=57577) | [3q13.31](http://www.ncbi.nlm.nih.gov/mapview/map_search.cgi?direct=on&idtype=gene&id=57577) | [Hs.477159](http://www.ncbi.nlm.nih.gov/UniGene/clust.cgi?ORG=Hs&CID=477159) | [2](http://www.ncbi.nih.gov/entrez/query.fcgi?tool=bioconductor&cmd=Retrieve&db=PubMed&list_uids=10718198%2C12477932) |  |  |
| [228574_at](https://www.affymetrix.com/LinkServlet?&probeset=228574_at) | TMTC2 | transmembrane and tetratricopeptide repeat containing 2 | 12 | 83080933 | [AI862551](http://www.ncbi.nlm.nih.gov/entrez/query.fcgi?cmd=search&db=nucleotide&term=AI862551%5BACCN%5D&doptcmdl=GenBank) | [160335](http://www.ncbi.nlm.nih.gov/sites/entrez?Db=gene&Cmd=DetailsSearch&Term=160335) | [12q21.31](http://www.ncbi.nlm.nih.gov/mapview/map_search.cgi?direct=on&idtype=gene&id=160335) | [Hs.577775](http://www.ncbi.nlm.nih.gov/UniGene/clust.cgi?ORG=Hs&CID=577775) | [13](http://www.ncbi.nih.gov/entrez/query.fcgi?tool=bioconductor&cmd=Retrieve&db=PubMed&list_uids=11076863%2C11230166%2C11256614%2C12477932%2C14702039%2C15489334%2C15489336%2C16381901%2C18519826%2C19386601%2C19625618%2C20379614%2C20662065) | [binding](http://amigo.geneontology.org/cgi-bin/amigo/go.cgi?view=details&query=GO:0005488)  [endoplasmic reticulum](http://amigo.geneontology.org/cgi-bin/amigo/go.cgi?view=details&query=GO:0005783)  [membrane](http://amigo.geneontology.org/cgi-bin/amigo/go.cgi?view=details&query=GO:0016020)  [integral to membrane](http://amigo.geneontology.org/cgi-bin/amigo/go.cgi?view=details&query=GO:0016021) |  |
| [228749_at](https://www.affymetrix.com/LinkServlet?&probeset=228749_at) | ZDBF2 | zinc finger, DBF-type containing 2 | 2 | 207139522 | [AV734793](http://www.ncbi.nlm.nih.gov/entrez/query.fcgi?cmd=search&db=nucleotide&term=AV734793%5BACCN%5D&doptcmdl=GenBank) | [57683](http://www.ncbi.nlm.nih.gov/sites/entrez?Db=gene&Cmd=DetailsSearch&Term=57683) | [2q33.3](http://www.ncbi.nlm.nih.gov/mapview/map_search.cgi?direct=on&idtype=gene&id=57683) | [Hs.110489](http://www.ncbi.nlm.nih.gov/UniGene/clust.cgi?ORG=Hs&CID=110489) | [3](http://www.ncbi.nih.gov/entrez/query.fcgi?tool=bioconductor&cmd=Retrieve&db=PubMed&list_uids=10997877%2C14702039%2C19200453) | [nucleic acid binding](http://amigo.geneontology.org/cgi-bin/amigo/go.cgi?view=details&query=GO:0003676)  [zinc ion binding](http://amigo.geneontology.org/cgi-bin/amigo/go.cgi?view=details&query=GO:0008270)  [metal ion binding](http://amigo.geneontology.org/cgi-bin/amigo/go.cgi?view=details&query=GO:0046872) |  |
| [228783_at](https://www.affymetrix.com/LinkServlet?&probeset=228783_at) | BVES | blood vessel epicardial substance | 6 | -105544699, -105544699 | [AA993518](http://www.ncbi.nlm.nih.gov/entrez/query.fcgi?cmd=search&db=nucleotide&term=AA993518%5BACCN%5D&doptcmdl=GenBank) | [11149](http://www.ncbi.nlm.nih.gov/sites/entrez?Db=gene&Cmd=DetailsSearch&Term=11149) | [6q21](http://www.ncbi.nlm.nih.gov/mapview/map_search.cgi?direct=on&idtype=gene&id=11149) | [Hs.221660](http://www.ncbi.nlm.nih.gov/UniGene/clust.cgi?ORG=Hs&CID=221660) | [11](http://www.ncbi.nih.gov/entrez/query.fcgi?tool=bioconductor&cmd=Retrieve&db=PubMed&list_uids=10441744%2C10882522%2C12477932%2C15489334%2C16188940%2C17207965%2C18349282%2C18391951%2C19448622%2C20546612%2C20627872) | [positive regulation of receptor recycling](http://amigo.geneontology.org/cgi-bin/amigo/go.cgi?view=details&query=GO:0001921)  [structural molecule activity](http://amigo.geneontology.org/cgi-bin/amigo/go.cgi?view=details&query=GO:0005198)  [plasma membrane](http://amigo.geneontology.org/cgi-bin/amigo/go.cgi?view=details&query=GO:0005886)  [tight junction](http://amigo.geneontology.org/cgi-bin/amigo/go.cgi?view=details&query=GO:0005923)  [cell adhesion](http://amigo.geneontology.org/cgi-bin/amigo/go.cgi?view=details&query=GO:0007155)  [multicellular organismal development](http://amigo.geneontology.org/cgi-bin/amigo/go.cgi?view=details&query=GO:0007275)  [muscle organ development](http://amigo.geneontology.org/cgi-bin/amigo/go.cgi?view=details&query=GO:0007517)  [regulation of cell shape](http://amigo.geneontology.org/cgi-bin/amigo/go.cgi?view=details&query=GO:0008360)  [integral to membrane](http://amigo.geneontology.org/cgi-bin/amigo/go.cgi?view=details&query=GO:0016021)  [integral to membrane](http://amigo.geneontology.org/cgi-bin/amigo/go.cgi?view=details&query=GO:0016021)  [vesicle-mediated transport](http://amigo.geneontology.org/cgi-bin/amigo/go.cgi?view=details&query=GO:0016192)  [lateral plasma membrane](http://amigo.geneontology.org/cgi-bin/amigo/go.cgi?view=details&query=GO:0016328)  [cell junction](http://amigo.geneontology.org/cgi-bin/amigo/go.cgi?view=details&query=GO:0030054)  [regulation of Rac GTPase activity](http://amigo.geneontology.org/cgi-bin/amigo/go.cgi?view=details&query=GO:0032314)  [substrate adhesion-dependent cell spreading](http://amigo.geneontology.org/cgi-bin/amigo/go.cgi?view=details&query=GO:0034446)  [positive regulation of locomotion](http://amigo.geneontology.org/cgi-bin/amigo/go.cgi?view=details&query=GO:0040017)  [regulation of Cdc42 GTPase activity](http://amigo.geneontology.org/cgi-bin/amigo/go.cgi?view=details&query=GO:0043088)  [epithelial cell-cell adhesion](http://amigo.geneontology.org/cgi-bin/amigo/go.cgi?view=details&query=GO:0090136) |  |
| [229332_at](https://www.affymetrix.com/LinkServlet?&probeset=229332_at) | HPDL | 4-hydroxyphenylpyruvate dioxygenase-like | 1 | 45792544 | [AI653050](http://www.ncbi.nlm.nih.gov/entrez/query.fcgi?cmd=search&db=nucleotide&term=AI653050%5BACCN%5D&doptcmdl=GenBank) | [84842](http://www.ncbi.nlm.nih.gov/sites/entrez?Db=gene&Cmd=DetailsSearch&Term=84842) | [1p34.1](http://www.ncbi.nlm.nih.gov/mapview/map_search.cgi?direct=on&idtype=gene&id=84842) | [Hs.162717](http://www.ncbi.nlm.nih.gov/UniGene/clust.cgi?ORG=Hs&CID=162717) | [3](http://www.ncbi.nih.gov/entrez/query.fcgi?tool=bioconductor&cmd=Retrieve&db=PubMed&list_uids=12477932%2C15489334%2C16710414) | [4-hydroxyphenylpyruvate dioxygenase activity](http://amigo.geneontology.org/cgi-bin/amigo/go.cgi?view=details&query=GO:0003868)  [aromatic amino acid family metabolic process](http://amigo.geneontology.org/cgi-bin/amigo/go.cgi?view=details&query=GO:0009072)  [oxidoreductase activity](http://amigo.geneontology.org/cgi-bin/amigo/go.cgi?view=details&query=GO:0016491)  [metal ion binding](http://amigo.geneontology.org/cgi-bin/amigo/go.cgi?view=details&query=GO:0046872)  [oxidation-reduction process](http://amigo.geneontology.org/cgi-bin/amigo/go.cgi?view=details&query=GO:0055114) |  |
| [229465_s_at](https://www.affymetrix.com/LinkServlet?&probeset=229465_s_at) | PTPRS | protein tyrosine phosphatase, receptor type, S | 19 | -5205519 | [BF433071](http://www.ncbi.nlm.nih.gov/entrez/query.fcgi?cmd=search&db=nucleotide&term=BF433071%5BACCN%5D&doptcmdl=GenBank) | [5802](http://www.ncbi.nlm.nih.gov/sites/entrez?Db=gene&Cmd=DetailsSearch&Term=5802) | [19p13.3](http://www.ncbi.nlm.nih.gov/mapview/map_search.cgi?direct=on&idtype=gene&id=5802) | [Hs.728875](http://www.ncbi.nlm.nih.gov/UniGene/clust.cgi?ORG=Hs&CID=728875) | [26](http://www.ncbi.nih.gov/entrez/query.fcgi?tool=bioconductor&cmd=Retrieve&db=PubMed&list_uids=1370651%2C8524829%2C8954782%2C8992885%2C9357975%2C9566880%2C9624153%2C9790732%2C10080191%2C10080192%2C10435588%2C10777529%2C12376545%2C12477932%2C15057824%2C15146197%2C16169070%2C16273344%2C16335952%2C16552719%2C17353931%2C17614280%2C17893260%2C19000305%2C20139422%2C20179269) | [receptor activity](http://amigo.geneontology.org/cgi-bin/amigo/go.cgi?view=details&query=GO:0004872)  [transmembrane receptor protein tyrosine phosphatase activity](http://amigo.geneontology.org/cgi-bin/amigo/go.cgi?view=details&query=GO:0005001)  [protein binding](http://amigo.geneontology.org/cgi-bin/amigo/go.cgi?view=details&query=GO:0005515)  [integral to plasma membrane](http://amigo.geneontology.org/cgi-bin/amigo/go.cgi?view=details&query=GO:0005887)  [protein dephosphorylation](http://amigo.geneontology.org/cgi-bin/amigo/go.cgi?view=details&query=GO:0006470)  [cell adhesion](http://amigo.geneontology.org/cgi-bin/amigo/go.cgi?view=details&query=GO:0007155)  [membrane](http://amigo.geneontology.org/cgi-bin/amigo/go.cgi?view=details&query=GO:0016020)  [hydrolase activity](http://amigo.geneontology.org/cgi-bin/amigo/go.cgi?view=details&query=GO:0016787) |  |
| [229629_at](https://www.affymetrix.com/LinkServlet?&probeset=229629_at) |  |  |  |  | [AI923633](http://www.ncbi.nlm.nih.gov/entrez/query.fcgi?cmd=search&db=nucleotide&term=AI923633%5BACCN%5D&doptcmdl=GenBank) |  |  |  |  |  |  |
| [230083_at](https://www.affymetrix.com/LinkServlet?&probeset=230083_at) | USP53 | ubiquitin specific peptidase 53 | 4 | 120133781 | [AW188464](http://www.ncbi.nlm.nih.gov/entrez/query.fcgi?cmd=search&db=nucleotide&term=AW188464%5BACCN%5D&doptcmdl=GenBank) | [54532](http://www.ncbi.nlm.nih.gov/sites/entrez?Db=gene&Cmd=DetailsSearch&Term=54532) | [4q26](http://www.ncbi.nlm.nih.gov/mapview/map_search.cgi?direct=on&idtype=gene&id=54532) | [Hs.431081](http://www.ncbi.nlm.nih.gov/UniGene/clust.cgi?ORG=Hs&CID=431081) [Hs.595368](http://www.ncbi.nlm.nih.gov/UniGene/clust.cgi?ORG=Hs&CID=595368) | [11](http://www.ncbi.nih.gov/entrez/query.fcgi?tool=bioconductor&cmd=Retrieve&db=PubMed&list_uids=10718198%2C12477932%2C14702039%2C14715245%2C15489334%2C15851553%2C16189514%2C16344560%2C19615732%2C20332099%2C20936779) | [cellular_component](http://amigo.geneontology.org/cgi-bin/amigo/go.cgi?view=details&query=GO:0005575)  [ubiquitin-dependent protein catabolic process](http://amigo.geneontology.org/cgi-bin/amigo/go.cgi?view=details&query=GO:0006511)  [biological_process](http://amigo.geneontology.org/cgi-bin/amigo/go.cgi?view=details&query=GO:0008150) |  |
| [230271_at](https://www.affymetrix.com/LinkServlet?&probeset=230271_at) | ONECUT2 | one cut homeobox 2 | 18 | 55102916 | [BG150301](http://www.ncbi.nlm.nih.gov/entrez/query.fcgi?cmd=search&db=nucleotide&term=BG150301%5BACCN%5D&doptcmdl=GenBank) | [9480](http://www.ncbi.nlm.nih.gov/sites/entrez?Db=gene&Cmd=DetailsSearch&Term=9480) | [18q21.31](http://www.ncbi.nlm.nih.gov/mapview/map_search.cgi?direct=on&idtype=gene&id=9480) | [Hs.194725](http://www.ncbi.nlm.nih.gov/UniGene/clust.cgi?ORG=Hs&CID=194725) | [8](http://www.ncbi.nih.gov/entrez/query.fcgi?tool=bioconductor&cmd=Retrieve&db=PubMed&list_uids=9915796%2C11478782%2C12477932%2C14702039%2C16912168%2C18288132%2C18418398%2C19336002) | [liver development](http://amigo.geneontology.org/cgi-bin/amigo/go.cgi?view=details&query=GO:0001889)  [regulation of cell-matrix adhesion](http://amigo.geneontology.org/cgi-bin/amigo/go.cgi?view=details&query=GO:0001952)  [epithelial cell development](http://amigo.geneontology.org/cgi-bin/amigo/go.cgi?view=details&query=GO:0002064)  [sequence-specific DNA binding transcription factor activity](http://amigo.geneontology.org/cgi-bin/amigo/go.cgi?view=details&query=GO:0003700)  [RNA polymerase II transcription factor activity](http://amigo.geneontology.org/cgi-bin/amigo/go.cgi?view=details&query=GO:0003702)  [nucleus](http://amigo.geneontology.org/cgi-bin/amigo/go.cgi?view=details&query=GO:0005634)  [regulation of transcription, DNA-dependent](http://amigo.geneontology.org/cgi-bin/amigo/go.cgi?view=details&query=GO:0006355)  [organ morphogenesis](http://amigo.geneontology.org/cgi-bin/amigo/go.cgi?view=details&query=GO:0009887)  [positive regulation of cell migration](http://amigo.geneontology.org/cgi-bin/amigo/go.cgi?view=details&query=GO:0030335)  [negative regulation of transforming growth factor beta receptor signaling pathway](http://amigo.geneontology.org/cgi-bin/amigo/go.cgi?view=details&query=GO:0030512)  [endocrine pancreas development](http://amigo.geneontology.org/cgi-bin/amigo/go.cgi?view=details&query=GO:0031018)  [cilium assembly](http://amigo.geneontology.org/cgi-bin/amigo/go.cgi?view=details&query=GO:0042384)  [sequence-specific DNA binding](http://amigo.geneontology.org/cgi-bin/amigo/go.cgi?view=details&query=GO:0043565)  [cell fate commitment](http://amigo.geneontology.org/cgi-bin/amigo/go.cgi?view=details&query=GO:0045165)  [positive regulation of transcription from RNA polymerase II promoter](http://amigo.geneontology.org/cgi-bin/amigo/go.cgi?view=details&query=GO:0045944)  [peripheral nervous system neuron development](http://amigo.geneontology.org/cgi-bin/amigo/go.cgi?view=details&query=GO:0048935) |  |
| [230351_at](https://www.affymetrix.com/LinkServlet?&probeset=230351_at) | LOC283481 | hypothetical LOC283481 | 13 | 103046927 | [AW070248](http://www.ncbi.nlm.nih.gov/entrez/query.fcgi?cmd=search&db=nucleotide&term=AW070248%5BACCN%5D&doptcmdl=GenBank) | [283481](http://www.ncbi.nlm.nih.gov/sites/entrez?Db=gene&Cmd=DetailsSearch&Term=283481) | [13q33.1](http://www.ncbi.nlm.nih.gov/mapview/map_search.cgi?direct=on&idtype=gene&id=283481) | [Hs.646604](http://www.ncbi.nlm.nih.gov/UniGene/clust.cgi?ORG=Hs&CID=646604) | [3](http://www.ncbi.nih.gov/entrez/query.fcgi?tool=bioconductor&cmd=Retrieve&db=PubMed&list_uids=8889548%2C11991713%2C12477932) |  |  |
| [230383_x_at](https://www.affymetrix.com/LinkServlet?&probeset=230383_x_at) |  |  |  |  | [AA133285](http://www.ncbi.nlm.nih.gov/entrez/query.fcgi?cmd=search&db=nucleotide&term=AA133285%5BACCN%5D&doptcmdl=GenBank) |  |  |  |  |  |  |
| [230421_at](https://www.affymetrix.com/LinkServlet?&probeset=230421_at) | ZNF879 | zinc finger protein 879 | 5 | 178450775 | [AI340241](http://www.ncbi.nlm.nih.gov/entrez/query.fcgi?cmd=search&db=nucleotide&term=AI340241%5BACCN%5D&doptcmdl=GenBank) | [345462](http://www.ncbi.nlm.nih.gov/sites/entrez?Db=gene&Cmd=DetailsSearch&Term=345462) | [5q35.3](http://www.ncbi.nlm.nih.gov/mapview/map_search.cgi?direct=on&idtype=gene&id=345462) | [Hs.445740](http://www.ncbi.nlm.nih.gov/UniGene/clust.cgi?ORG=Hs&CID=445740) |  | [DNA binding](http://amigo.geneontology.org/cgi-bin/amigo/go.cgi?view=details&query=GO:0003677)  [intracellular](http://amigo.geneontology.org/cgi-bin/amigo/go.cgi?view=details&query=GO:0005622)  [nucleus](http://amigo.geneontology.org/cgi-bin/amigo/go.cgi?view=details&query=GO:0005634)  [regulation of transcription, DNA-dependent](http://amigo.geneontology.org/cgi-bin/amigo/go.cgi?view=details&query=GO:0006355)  [zinc ion binding](http://amigo.geneontology.org/cgi-bin/amigo/go.cgi?view=details&query=GO:0008270)  [metal ion binding](http://amigo.geneontology.org/cgi-bin/amigo/go.cgi?view=details&query=GO:0046872) |  |
| [230423_at](https://www.affymetrix.com/LinkServlet?&probeset=230423_at) |  |  |  |  | [AI554075](http://www.ncbi.nlm.nih.gov/entrez/query.fcgi?cmd=search&db=nucleotide&term=AI554075%5BACCN%5D&doptcmdl=GenBank) |  |  |  |  |  |  |
| [230624_at](https://www.affymetrix.com/LinkServlet?&probeset=230624_at) | SLC25A27 | solute carrier family 25, member 27 | 6 | 46620678 | [AW779950](http://www.ncbi.nlm.nih.gov/entrez/query.fcgi?cmd=search&db=nucleotide&term=AW779950%5BACCN%5D&doptcmdl=GenBank) | [9481](http://www.ncbi.nlm.nih.gov/sites/entrez?Db=gene&Cmd=DetailsSearch&Term=9481) | [6p12.3](http://www.ncbi.nlm.nih.gov/mapview/map_search.cgi?direct=on&idtype=gene&id=9481) | [Hs.40510](http://www.ncbi.nlm.nih.gov/UniGene/clust.cgi?ORG=Hs&CID=40510) | [22](http://www.ncbi.nih.gov/entrez/query.fcgi?tool=bioconductor&cmd=Retrieve&db=PubMed&list_uids=8889548%2C10025957%2C10620491%2C10772343%2C10928996%2C11994670%2C12477932%2C12975309%2C14702039%2C15342556%2C16344560%2C16378686%2C16775390%2C17035241%2C17066476%2C17601350%2C18755175%2C18977241%2C19150400%2C19536655%2C20545631%2C20877624) | [mitochondrion](http://amigo.geneontology.org/cgi-bin/amigo/go.cgi?view=details&query=GO:0005739)  [mitochondrial inner membrane](http://amigo.geneontology.org/cgi-bin/amigo/go.cgi?view=details&query=GO:0005743)  [generation of precursor metabolites and energy](http://amigo.geneontology.org/cgi-bin/amigo/go.cgi?view=details&query=GO:0006091)  [transport](http://amigo.geneontology.org/cgi-bin/amigo/go.cgi?view=details&query=GO:0006810)  [membrane](http://amigo.geneontology.org/cgi-bin/amigo/go.cgi?view=details&query=GO:0016020)  [integral to membrane](http://amigo.geneontology.org/cgi-bin/amigo/go.cgi?view=details&query=GO:0016021) |  |
| [231152_at](https://www.affymetrix.com/LinkServlet?&probeset=231152_at) | INO80D | INO80 complex subunit D | 2 | -206858445 | [AW452971](http://www.ncbi.nlm.nih.gov/entrez/query.fcgi?cmd=search&db=nucleotide&term=AW452971%5BACCN%5D&doptcmdl=GenBank) | [54891](http://www.ncbi.nlm.nih.gov/sites/entrez?Db=gene&Cmd=DetailsSearch&Term=54891) | [2q33.3](http://www.ncbi.nlm.nih.gov/mapview/map_search.cgi?direct=on&idtype=gene&id=54891) | [Hs.445036](http://www.ncbi.nlm.nih.gov/UniGene/clust.cgi?ORG=Hs&CID=445036) | [8](http://www.ncbi.nih.gov/entrez/query.fcgi?tool=bioconductor&cmd=Retrieve&db=PubMed&list_uids=8125298%2C9373149%2C12477932%2C14702039%2C16230350%2C16344560%2C19553259%2C21303910) |  |  |
| [231315_at](https://www.affymetrix.com/LinkServlet?&probeset=231315_at) | NKX2-1 | NK2 homeobox 1 | 14 | -36985605, -36985605 | [AI807728](http://www.ncbi.nlm.nih.gov/entrez/query.fcgi?cmd=search&db=nucleotide&term=AI807728%5BACCN%5D&doptcmdl=GenBank) | [7080](http://www.ncbi.nlm.nih.gov/sites/entrez?Db=gene&Cmd=DetailsSearch&Term=7080) | [14q13](http://www.ncbi.nlm.nih.gov/mapview/map_search.cgi?direct=on&idtype=gene&id=7080) | [Hs.94367](http://www.ncbi.nlm.nih.gov/UniGene/clust.cgi?ORG=Hs&CID=94367) | [108](http://www.ncbi.nih.gov/entrez/query.fcgi?tool=bioconductor&cmd=Retrieve&db=PubMed&list_uids=1735431%2C1976511%2C7559607%2C7635972%2C7711079%2C7711080%2C7713914%2C8675988%2C8889548%2C9396717%2C9545595%2C9582279%2C9988700%2C10617585%2C10733581%2C11076796%2C11152647%2C11274148%2C11438542%2C11713256%2C11733512%2C11836702%2C11854318%2C11854319%2C11923479%2C11953175%2C11957142%2C11971878%2C12040027%2C12051643%2C12161428%2C12408771%2C12441357%2C12477932%2C12499091%2C12684771%2C12923324%2C14633512%2C14720435%2C14960358%2C14970209%2C15098009%2C15173172%2C15271884%2C15279903%2C15449938%2C15485815%2C15489334%2C15548547%2C15929662%2C15955952%2C16220345%2C16260629%2C16314749%2C16461352%2C16565516%2C16613858%2C16630564%2C16960125%2C16980598%2C17044090%2C17182767%2C17220277%2C17412341%2C17413979%2C17474147%2C17616654%2C17640327%2C17671725%2C17702043%2C17765926%2C18059234%2C18071837%2C18212743%2C18239190%2C18379122%2C18391950%2C18487360%2C18661567%2C18682709%2C18788921%2C18855882%2C18957494%2C18958156%2C18997617%2C19011567%2C19047914%2C19064983%2C19176457%2C19198613%2C19293183%2C19329538%2C19336474%2C19365834%2C19483637%2C19506552%2C19525896%2C19578049%2C19730683%2C19740516%2C19906647%2C20020530%2C20042854%2C20157192%2C20518411%2C20701785%2C20734064%2C20830690) | [negative regulation of transcription from RNA polymerase II promoter](http://amigo.geneontology.org/cgi-bin/amigo/go.cgi?view=details&query=GO:0000122)  [neuron migration](http://amigo.geneontology.org/cgi-bin/amigo/go.cgi?view=details&query=GO:0001764)  [regulation of blood volume by renin-angiotensin](http://amigo.geneontology.org/cgi-bin/amigo/go.cgi?view=details&query=GO:0002016)  [DNA binding](http://amigo.geneontology.org/cgi-bin/amigo/go.cgi?view=details&query=GO:0003677)  [sequence-specific DNA binding transcription factor activity](http://amigo.geneontology.org/cgi-bin/amigo/go.cgi?view=details&query=GO:0003700)  [specific RNA polymerase II transcription factor activity](http://amigo.geneontology.org/cgi-bin/amigo/go.cgi?view=details&query=GO:0003704)  [sequence-specific enhancer binding RNA polymerase II transcription factor activity](http://amigo.geneontology.org/cgi-bin/amigo/go.cgi?view=details&query=GO:0003705)  [protein binding](http://amigo.geneontology.org/cgi-bin/amigo/go.cgi?view=details&query=GO:0005515)  [soluble fraction](http://amigo.geneontology.org/cgi-bin/amigo/go.cgi?view=details&query=GO:0005625)  [nucleus](http://amigo.geneontology.org/cgi-bin/amigo/go.cgi?view=details&query=GO:0005634)  [nucleoplasm](http://amigo.geneontology.org/cgi-bin/amigo/go.cgi?view=details&query=GO:0005654)  [transcription factor complex](http://amigo.geneontology.org/cgi-bin/amigo/go.cgi?view=details&query=GO:0005667)  [transcription, DNA-dependent](http://amigo.geneontology.org/cgi-bin/amigo/go.cgi?view=details&query=GO:0006351)  [phospholipid metabolic process](http://amigo.geneontology.org/cgi-bin/amigo/go.cgi?view=details&query=GO:0006644)  [pattern specification process](http://amigo.geneontology.org/cgi-bin/amigo/go.cgi?view=details&query=GO:0007389)  [axon guidance](http://amigo.geneontology.org/cgi-bin/amigo/go.cgi?view=details&query=GO:0007411)  [brain development](http://amigo.geneontology.org/cgi-bin/amigo/go.cgi?view=details&query=GO:0007420)  [endoderm development](http://amigo.geneontology.org/cgi-bin/amigo/go.cgi?view=details&query=GO:0007492)  [locomotory behavior](http://amigo.geneontology.org/cgi-bin/amigo/go.cgi?view=details&query=GO:0007626)  [feeding behavior](http://amigo.geneontology.org/cgi-bin/amigo/go.cgi?view=details&query=GO:0007631)  [response to hormone stimulus](http://amigo.geneontology.org/cgi-bin/amigo/go.cgi?view=details&query=GO:0009725)  [organ morphogenesis](http://amigo.geneontology.org/cgi-bin/amigo/go.cgi?view=details&query=GO:0009887)  [positive regulation of gene-specific transcription from RNA polymerase II promoter](http://amigo.geneontology.org/cgi-bin/amigo/go.cgi?view=details&query=GO:0010552)  [positive regulation of gene expression](http://amigo.geneontology.org/cgi-bin/amigo/go.cgi?view=details&query=GO:0010628)  [negative regulation of epithelial to mesenchymal transition](http://amigo.geneontology.org/cgi-bin/amigo/go.cgi?view=details&query=GO:0010719)  [promoter binding](http://amigo.geneontology.org/cgi-bin/amigo/go.cgi?view=details&query=GO:0010843)  [transcription activator activity](http://amigo.geneontology.org/cgi-bin/amigo/go.cgi?view=details&query=GO:0016563)  [globus pallidus development](http://amigo.geneontology.org/cgi-bin/amigo/go.cgi?view=details&query=GO:0021759)  [hippocampus development](http://amigo.geneontology.org/cgi-bin/amigo/go.cgi?view=details&query=GO:0021766)  [cerebral cortex cell migration](http://amigo.geneontology.org/cgi-bin/amigo/go.cgi?view=details&query=GO:0021795)  [forebrain dorsal/ventral pattern formation](http://amigo.geneontology.org/cgi-bin/amigo/go.cgi?view=details&query=GO:0021798)  [forebrain neuron fate commitment](http://amigo.geneontology.org/cgi-bin/amigo/go.cgi?view=details&query=GO:0021877)  [cerebral cortex GABAergic interneuron differentiation](http://amigo.geneontology.org/cgi-bin/amigo/go.cgi?view=details&query=GO:0021892)  [pituitary gland development](http://amigo.geneontology.org/cgi-bin/amigo/go.cgi?view=details&query=GO:0021983)  [lung development](http://amigo.geneontology.org/cgi-bin/amigo/go.cgi?view=details&query=GO:0030324)  [negative regulation of cell migration](http://amigo.geneontology.org/cgi-bin/amigo/go.cgi?view=details&query=GO:0030336)  [negative regulation of transforming growth factor beta receptor signaling pathway](http://amigo.geneontology.org/cgi-bin/amigo/go.cgi?view=details&query=GO:0030512)  [thyroid gland development](http://amigo.geneontology.org/cgi-bin/amigo/go.cgi?view=details&query=GO:0030878)  [developmental induction](http://amigo.geneontology.org/cgi-bin/amigo/go.cgi?view=details&query=GO:0031128)  [response to lipopolysaccharide](http://amigo.geneontology.org/cgi-bin/amigo/go.cgi?view=details&query=GO:0032496)  [Leydig cell differentiation](http://amigo.geneontology.org/cgi-bin/amigo/go.cgi?view=details&query=GO:0033327)  [hyperosmotic salinity response](http://amigo.geneontology.org/cgi-bin/amigo/go.cgi?view=details&query=GO:0042538)  [menarche](http://amigo.geneontology.org/cgi-bin/amigo/go.cgi?view=details&query=GO:0042696)  [response to ethanol](http://amigo.geneontology.org/cgi-bin/amigo/go.cgi?view=details&query=GO:0045471)  [positive regulation of transcription, DNA-dependent](http://amigo.geneontology.org/cgi-bin/amigo/go.cgi?view=details&query=GO:0045893)  [positive regulation of transcription, DNA-dependent](http://amigo.geneontology.org/cgi-bin/amigo/go.cgi?view=details&query=GO:0045893)  [positive regulation of transcription from RNA polymerase II promoter](http://amigo.geneontology.org/cgi-bin/amigo/go.cgi?view=details&query=GO:0045944)  [development of primary female sexual characteristics](http://amigo.geneontology.org/cgi-bin/amigo/go.cgi?view=details&query=GO:0046545)  [anatomical structure formation involved in morphogenesis](http://amigo.geneontology.org/cgi-bin/amigo/go.cgi?view=details&query=GO:0048646)  [oligodendrocyte differentiation](http://amigo.geneontology.org/cgi-bin/amigo/go.cgi?view=details&query=GO:0048709)  [lung saccule development](http://amigo.geneontology.org/cgi-bin/amigo/go.cgi?view=details&query=GO:0060430)  [epithelial tube branching involved in lung morphogenesis](http://amigo.geneontology.org/cgi-bin/amigo/go.cgi?view=details&query=GO:0060441)  [Clara cell differentiation](http://amigo.geneontology.org/cgi-bin/amigo/go.cgi?view=details&query=GO:0060486)  [Type II pneumocyte differentiation](http://amigo.geneontology.org/cgi-bin/amigo/go.cgi?view=details&query=GO:0060510) |  |
| [231472_at](https://www.affymetrix.com/LinkServlet?&probeset=231472_at) | FBXO15 | F-box protein 15 | 18 | -71740587, -71740587 | [BE464323](http://www.ncbi.nlm.nih.gov/entrez/query.fcgi?cmd=search&db=nucleotide&term=BE464323%5BACCN%5D&doptcmdl=GenBank) | [201456](http://www.ncbi.nlm.nih.gov/sites/entrez?Db=gene&Cmd=DetailsSearch&Term=201456) | [18q22.3](http://www.ncbi.nlm.nih.gov/mapview/map_search.cgi?direct=on&idtype=gene&id=201456) | [Hs.664011](http://www.ncbi.nlm.nih.gov/UniGene/clust.cgi?ORG=Hs&CID=664011) | [8](http://www.ncbi.nih.gov/entrez/query.fcgi?tool=bioconductor&cmd=Retrieve&db=PubMed&list_uids=8889548%2C10531037%2C12477932%2C12665572%2C14702039%2C15489334%2C15520277%2C17207965) | [SCF ubiquitin ligase complex](http://amigo.geneontology.org/cgi-bin/amigo/go.cgi?view=details&query=GO:0019005) |  |
| [231822_at](https://www.affymetrix.com/LinkServlet?&probeset=231822_at) | CTTNBP2NL | CTTNBP2 N-terminal like | 1 | 112938799 | [AI921883](http://www.ncbi.nlm.nih.gov/entrez/query.fcgi?cmd=search&db=nucleotide&term=AI921883%5BACCN%5D&doptcmdl=GenBank) | [55917](http://www.ncbi.nlm.nih.gov/sites/entrez?Db=gene&Cmd=DetailsSearch&Term=55917) | [1p13.2](http://www.ncbi.nlm.nih.gov/mapview/map_search.cgi?direct=on&idtype=gene&id=55917) | [Hs.687503](http://www.ncbi.nlm.nih.gov/UniGene/clust.cgi?ORG=Hs&CID=687503) | [16](http://www.ncbi.nih.gov/entrez/query.fcgi?tool=bioconductor&cmd=Retrieve&db=PubMed&list_uids=10718198%2C11076863%2C11256614%2C12477932%2C14702039%2C15302935%2C15345747%2C15489334%2C15489336%2C16344560%2C16381901%2C16710414%2C16964243%2C18029348%2C18782753%2C19615732) | [protein binding](http://amigo.geneontology.org/cgi-bin/amigo/go.cgi?view=details&query=GO:0005515)  [actin cytoskeleton](http://amigo.geneontology.org/cgi-bin/amigo/go.cgi?view=details&query=GO:0015629) |  |
| [233446_at](https://www.affymetrix.com/LinkServlet?&probeset=233446_at) | ONECUT2 | one cut homeobox 2 | 18 | 55102916 | [AU145336](http://www.ncbi.nlm.nih.gov/entrez/query.fcgi?cmd=search&db=nucleotide&term=AU145336%5BACCN%5D&doptcmdl=GenBank) | [9480](http://www.ncbi.nlm.nih.gov/sites/entrez?Db=gene&Cmd=DetailsSearch&Term=9480) | [18q21.31](http://www.ncbi.nlm.nih.gov/mapview/map_search.cgi?direct=on&idtype=gene&id=9480) | [Hs.194725](http://www.ncbi.nlm.nih.gov/UniGene/clust.cgi?ORG=Hs&CID=194725) | [8](http://www.ncbi.nih.gov/entrez/query.fcgi?tool=bioconductor&cmd=Retrieve&db=PubMed&list_uids=9915796%2C11478782%2C12477932%2C14702039%2C16912168%2C18288132%2C18418398%2C19336002) | [liver development](http://amigo.geneontology.org/cgi-bin/amigo/go.cgi?view=details&query=GO:0001889)  [regulation of cell-matrix adhesion](http://amigo.geneontology.org/cgi-bin/amigo/go.cgi?view=details&query=GO:0001952)  [epithelial cell development](http://amigo.geneontology.org/cgi-bin/amigo/go.cgi?view=details&query=GO:0002064)  [sequence-specific DNA binding transcription factor activity](http://amigo.geneontology.org/cgi-bin/amigo/go.cgi?view=details&query=GO:0003700)  [RNA polymerase II transcription factor activity](http://amigo.geneontology.org/cgi-bin/amigo/go.cgi?view=details&query=GO:0003702)  [nucleus](http://amigo.geneontology.org/cgi-bin/amigo/go.cgi?view=details&query=GO:0005634)  [regulation of transcription, DNA-dependent](http://amigo.geneontology.org/cgi-bin/amigo/go.cgi?view=details&query=GO:0006355)  [organ morphogenesis](http://amigo.geneontology.org/cgi-bin/amigo/go.cgi?view=details&query=GO:0009887)  [positive regulation of cell migration](http://amigo.geneontology.org/cgi-bin/amigo/go.cgi?view=details&query=GO:0030335)  [negative regulation of transforming growth factor beta receptor signaling pathway](http://amigo.geneontology.org/cgi-bin/amigo/go.cgi?view=details&query=GO:0030512)  [endocrine pancreas development](http://amigo.geneontology.org/cgi-bin/amigo/go.cgi?view=details&query=GO:0031018)  [cilium assembly](http://amigo.geneontology.org/cgi-bin/amigo/go.cgi?view=details&query=GO:0042384)  [sequence-specific DNA binding](http://amigo.geneontology.org/cgi-bin/amigo/go.cgi?view=details&query=GO:0043565)  [cell fate commitment](http://amigo.geneontology.org/cgi-bin/amigo/go.cgi?view=details&query=GO:0045165)  [positive regulation of transcription from RNA polymerase II promoter](http://amigo.geneontology.org/cgi-bin/amigo/go.cgi?view=details&query=GO:0045944)  [peripheral nervous system neuron development](http://amigo.geneontology.org/cgi-bin/amigo/go.cgi?view=details&query=GO:0048935) |  |
| [233903_s_at](https://www.affymetrix.com/LinkServlet?&probeset=233903_s_at) | ARHGEF26 | Rho guanine nucleotide exchange factor (GEF) 26 | 3 | 153839148 | [AK022655](http://www.ncbi.nlm.nih.gov/entrez/query.fcgi?cmd=search&db=nucleotide&term=AK022655%5BACCN%5D&doptcmdl=GenBank) | [26084](http://www.ncbi.nlm.nih.gov/sites/entrez?Db=gene&Cmd=DetailsSearch&Term=26084) | [3q25.2](http://www.ncbi.nlm.nih.gov/mapview/map_search.cgi?direct=on&idtype=gene&id=26084) | [Hs.240845](http://www.ncbi.nlm.nih.gov/UniGene/clust.cgi?ORG=Hs&CID=240845) | [6](http://www.ncbi.nih.gov/entrez/query.fcgi?tool=bioconductor&cmd=Retrieve&db=PubMed&list_uids=12477932%2C12697679%2C14702039%2C15133129%2C15221005%2C16344560) |  | [Bacterial invasion of epithelial cells](http://www.genome.ad.jp/kegg/pathway/hsa/hsa05100.html) |
| [234107_s_at](https://www.affymetrix.com/LinkServlet?&probeset=234107_s_at) | DTD1 | D-tyrosyl-tRNA deacylase 1 homolog (S. cerevisiae) | 20 | 18568555 | [AL121780](http://www.ncbi.nlm.nih.gov/entrez/query.fcgi?cmd=search&db=nucleotide&term=AL121780%5BACCN%5D&doptcmdl=GenBank) | [92675](http://www.ncbi.nlm.nih.gov/sites/entrez?Db=gene&Cmd=DetailsSearch&Term=92675) | [20p11.23](http://www.ncbi.nlm.nih.gov/mapview/map_search.cgi?direct=on&idtype=gene&id=92675) | [Hs.659442](http://www.ncbi.nlm.nih.gov/UniGene/clust.cgi?ORG=Hs&CID=659442) | [10](http://www.ncbi.nih.gov/entrez/query.fcgi?tool=bioconductor&cmd=Retrieve&db=PubMed&list_uids=8889548%2C11780052%2C12392168%2C12477932%2C14702039%2C15489334%2C15653697%2C17207965%2C20065034%2C20379614) | [cytoplasm](http://amigo.geneontology.org/cgi-bin/amigo/go.cgi?view=details&query=GO:0005737)  [hydrolase activity, acting on ester bonds](http://amigo.geneontology.org/cgi-bin/amigo/go.cgi?view=details&query=GO:0016788)  [D-amino acid catabolic process](http://amigo.geneontology.org/cgi-bin/amigo/go.cgi?view=details&query=GO:0019478) |  |
| [235074_at](https://www.affymetrix.com/LinkServlet?&probeset=235074_at) | SPRED1 | sprouty-related, EVH1 domain containing 1 | 15 | 38545051 | [BF342524](http://www.ncbi.nlm.nih.gov/entrez/query.fcgi?cmd=search&db=nucleotide&term=BF342524%5BACCN%5D&doptcmdl=GenBank) | [161742](http://www.ncbi.nlm.nih.gov/sites/entrez?Db=gene&Cmd=DetailsSearch&Term=161742) | [15q14](http://www.ncbi.nlm.nih.gov/mapview/map_search.cgi?direct=on&idtype=gene&id=161742) | [Hs.525781](http://www.ncbi.nlm.nih.gov/UniGene/clust.cgi?ORG=Hs&CID=525781) | [23](http://www.ncbi.nih.gov/entrez/query.fcgi?tool=bioconductor&cmd=Retrieve&db=PubMed&list_uids=11493923%2C12477932%2C12646235%2C14702039%2C15231748%2C15465815%2C15683364%2C16115197%2C16344560%2C16652141%2C17094949%2C17672918%2C17704776%2C17974561%2C18216281%2C19120036%2C19366998%2C19443465%2C19913121%2C19920235%2C20339110%2C20628086%2C20945555) | [inactivation of MAPK activity](http://amigo.geneontology.org/cgi-bin/amigo/go.cgi?view=details&query=GO:0000188)  [stem cell factor receptor binding](http://amigo.geneontology.org/cgi-bin/amigo/go.cgi?view=details&query=GO:0005173)  [nucleus](http://amigo.geneontology.org/cgi-bin/amigo/go.cgi?view=details&query=GO:0005634)  [plasma membrane](http://amigo.geneontology.org/cgi-bin/amigo/go.cgi?view=details&query=GO:0005886)  [caveola](http://amigo.geneontology.org/cgi-bin/amigo/go.cgi?view=details&query=GO:0005901)  [multicellular organismal development](http://amigo.geneontology.org/cgi-bin/amigo/go.cgi?view=details&query=GO:0007275)  [regulation of signal transduction](http://amigo.geneontology.org/cgi-bin/amigo/go.cgi?view=details&query=GO:0009966) | [Jak-STAT signaling pathway](http://www.genome.ad.jp/kegg/pathway/hsa/hsa04630.html) |
| [237301_at](https://www.affymetrix.com/LinkServlet?&probeset=237301_at) |  |  |  |  | [BF433570](http://www.ncbi.nlm.nih.gov/entrez/query.fcgi?cmd=search&db=nucleotide&term=BF433570%5BACCN%5D&doptcmdl=GenBank) |  |  |  |  |  |  |
| [238271_x_at](https://www.affymetrix.com/LinkServlet?&probeset=238271_x_at) |  |  |  |  | [W90010](http://www.ncbi.nlm.nih.gov/entrez/query.fcgi?cmd=search&db=nucleotide&term=W90010%5BACCN%5D&doptcmdl=GenBank) |  |  |  |  |  |  |
| [238462_at](https://www.affymetrix.com/LinkServlet?&probeset=238462_at) | UBASH3B | ubiquitin associated and SH3 domain containing B | 11 | 122526397 | [AI418293](http://www.ncbi.nlm.nih.gov/entrez/query.fcgi?cmd=search&db=nucleotide&term=AI418293%5BACCN%5D&doptcmdl=GenBank) | [84959](http://www.ncbi.nlm.nih.gov/sites/entrez?Db=gene&Cmd=DetailsSearch&Term=84959) | [11q24.1](http://www.ncbi.nlm.nih.gov/mapview/map_search.cgi?direct=on&idtype=gene&id=84959) | [Hs.444075](http://www.ncbi.nlm.nih.gov/UniGene/clust.cgi?ORG=Hs&CID=444075) | [18](http://www.ncbi.nih.gov/entrez/query.fcgi?tool=bioconductor&cmd=Retrieve&db=PubMed&list_uids=8125298%2C8889548%2C9373149%2C11853319%2C12370296%2C12477932%2C12665801%2C14702039%2C15159412%2C15388581%2C15489334%2C16303743%2C16344560%2C16429130%2C17588522%2C18189269%2C20379614%2C20585042) | [protein tyrosine phosphatase activity](http://amigo.geneontology.org/cgi-bin/amigo/go.cgi?view=details&query=GO:0004725)  [nucleus](http://amigo.geneontology.org/cgi-bin/amigo/go.cgi?view=details&query=GO:0005634)  [cytoplasm](http://amigo.geneontology.org/cgi-bin/amigo/go.cgi?view=details&query=GO:0005737)  [hydrolase activity](http://amigo.geneontology.org/cgi-bin/amigo/go.cgi?view=details&query=GO:0016787) |  |
| [238497_at](https://www.affymetrix.com/LinkServlet?&probeset=238497_at) | TMEM136 | transmembrane protein 136 | 11 | 120196015 | [N51836](http://www.ncbi.nlm.nih.gov/entrez/query.fcgi?cmd=search&db=nucleotide&term=N51836%5BACCN%5D&doptcmdl=GenBank) | [219902](http://www.ncbi.nlm.nih.gov/sites/entrez?Db=gene&Cmd=DetailsSearch&Term=219902) | [11q23.3](http://www.ncbi.nlm.nih.gov/mapview/map_search.cgi?direct=on&idtype=gene&id=219902) | [Hs.643516](http://www.ncbi.nlm.nih.gov/UniGene/clust.cgi?ORG=Hs&CID=643516) | [2](http://www.ncbi.nih.gov/entrez/query.fcgi?tool=bioconductor&cmd=Retrieve&db=PubMed&list_uids=12477932%2C16344560) | [membrane](http://amigo.geneontology.org/cgi-bin/amigo/go.cgi?view=details&query=GO:0016020)  [integral to membrane](http://amigo.geneontology.org/cgi-bin/amigo/go.cgi?view=details&query=GO:0016021) |  |
| [238673_at](https://www.affymetrix.com/LinkServlet?&probeset=238673_at) | SAMD12 | sterile alpha motif domain containing 12 | 8 | -119390226, -119201694 | [AV724325](http://www.ncbi.nlm.nih.gov/entrez/query.fcgi?cmd=search&db=nucleotide&term=AV724325%5BACCN%5D&doptcmdl=GenBank) | [401474](http://www.ncbi.nlm.nih.gov/sites/entrez?Db=gene&Cmd=DetailsSearch&Term=401474) | [8q24.12](http://www.ncbi.nlm.nih.gov/mapview/map_search.cgi?direct=on&idtype=gene&id=401474) | [Hs.359393](http://www.ncbi.nlm.nih.gov/UniGene/clust.cgi?ORG=Hs&CID=359393) [Hs.701196](http://www.ncbi.nlm.nih.gov/UniGene/clust.cgi?ORG=Hs&CID=701196) | [6](http://www.ncbi.nih.gov/entrez/query.fcgi?tool=bioconductor&cmd=Retrieve&db=PubMed&list_uids=8889548%2C10737800%2C12477932%2C16344560%2C17568003%2C20379614) |  |  |
| [238716_at](https://www.affymetrix.com/LinkServlet?&probeset=238716_at) |  |  |  |  | [AL527331](http://www.ncbi.nlm.nih.gov/entrez/query.fcgi?cmd=search&db=nucleotide&term=AL527331%5BACCN%5D&doptcmdl=GenBank) |  |  |  |  |  |  |
| [238919_at](https://www.affymetrix.com/LinkServlet?&probeset=238919_at) |  |  |  |  | [R49295](http://www.ncbi.nlm.nih.gov/entrez/query.fcgi?cmd=search&db=nucleotide&term=R49295%5BACCN%5D&doptcmdl=GenBank) |  |  |  |  |  |  |
| [239624_at](https://www.affymetrix.com/LinkServlet?&probeset=239624_at) |  |  |  |  | [AA725362](http://www.ncbi.nlm.nih.gov/entrez/query.fcgi?cmd=search&db=nucleotide&term=AA725362%5BACCN%5D&doptcmdl=GenBank) |  |  |  |  |  |  |
| [239911_at](https://www.affymetrix.com/LinkServlet?&probeset=239911_at) | ONECUT2 | one cut homeobox 2 | 18 | 55102916 | [H49805](http://www.ncbi.nlm.nih.gov/entrez/query.fcgi?cmd=search&db=nucleotide&term=H49805%5BACCN%5D&doptcmdl=GenBank) | [9480](http://www.ncbi.nlm.nih.gov/sites/entrez?Db=gene&Cmd=DetailsSearch&Term=9480) | [18q21.31](http://www.ncbi.nlm.nih.gov/mapview/map_search.cgi?direct=on&idtype=gene&id=9480) | [Hs.194725](http://www.ncbi.nlm.nih.gov/UniGene/clust.cgi?ORG=Hs&CID=194725) | [8](http://www.ncbi.nih.gov/entrez/query.fcgi?tool=bioconductor&cmd=Retrieve&db=PubMed&list_uids=9915796%2C11478782%2C12477932%2C14702039%2C16912168%2C18288132%2C18418398%2C19336002) | [liver development](http://amigo.geneontology.org/cgi-bin/amigo/go.cgi?view=details&query=GO:0001889)  [regulation of cell-matrix adhesion](http://amigo.geneontology.org/cgi-bin/amigo/go.cgi?view=details&query=GO:0001952)  [epithelial cell development](http://amigo.geneontology.org/cgi-bin/amigo/go.cgi?view=details&query=GO:0002064)  [sequence-specific DNA binding transcription factor activity](http://amigo.geneontology.org/cgi-bin/amigo/go.cgi?view=details&query=GO:0003700)  [RNA polymerase II transcription factor activity](http://amigo.geneontology.org/cgi-bin/amigo/go.cgi?view=details&query=GO:0003702)  [nucleus](http://amigo.geneontology.org/cgi-bin/amigo/go.cgi?view=details&query=GO:0005634)  [regulation of transcription, DNA-dependent](http://amigo.geneontology.org/cgi-bin/amigo/go.cgi?view=details&query=GO:0006355)  [organ morphogenesis](http://amigo.geneontology.org/cgi-bin/amigo/go.cgi?view=details&query=GO:0009887)  [positive regulation of cell migration](http://amigo.geneontology.org/cgi-bin/amigo/go.cgi?view=details&query=GO:0030335)  [negative regulation of transforming growth factor beta receptor signaling pathway](http://amigo.geneontology.org/cgi-bin/amigo/go.cgi?view=details&query=GO:0030512)  [endocrine pancreas development](http://amigo.geneontology.org/cgi-bin/amigo/go.cgi?view=details&query=GO:0031018)  [cilium assembly](http://amigo.geneontology.org/cgi-bin/amigo/go.cgi?view=details&query=GO:0042384)  [sequence-specific DNA binding](http://amigo.geneontology.org/cgi-bin/amigo/go.cgi?view=details&query=GO:0043565)  [cell fate commitment](http://amigo.geneontology.org/cgi-bin/amigo/go.cgi?view=details&query=GO:0045165)  [positive regulation of transcription from RNA polymerase II promoter](http://amigo.geneontology.org/cgi-bin/amigo/go.cgi?view=details&query=GO:0045944)  [peripheral nervous system neuron development](http://amigo.geneontology.org/cgi-bin/amigo/go.cgi?view=details&query=GO:0048935) |  |
| [240069_at](https://www.affymetrix.com/LinkServlet?&probeset=240069_at) |  |  |  |  | [AI343600](http://www.ncbi.nlm.nih.gov/entrez/query.fcgi?cmd=search&db=nucleotide&term=AI343600%5BACCN%5D&doptcmdl=GenBank) |  |  |  |  |  |  |
| [240102_at](https://www.affymetrix.com/LinkServlet?&probeset=240102_at) |  |  |  |  | [AW024095](http://www.ncbi.nlm.nih.gov/entrez/query.fcgi?cmd=search&db=nucleotide&term=AW024095%5BACCN%5D&doptcmdl=GenBank) |  |  |  |  |  |  |
| [240546_at](https://www.affymetrix.com/LinkServlet?&probeset=240546_at) | LOC389043 | hypothetical LOC389043 | 2 | 132160473 | [AI653224](http://www.ncbi.nlm.nih.gov/entrez/query.fcgi?cmd=search&db=nucleotide&term=AI653224%5BACCN%5D&doptcmdl=GenBank) | [389043](http://www.ncbi.nlm.nih.gov/sites/entrez?Db=gene&Cmd=DetailsSearch&Term=389043) | [2q21.1](http://www.ncbi.nlm.nih.gov/mapview/map_search.cgi?direct=on&idtype=gene&id=389043) | [Hs.586791](http://www.ncbi.nlm.nih.gov/UniGene/clust.cgi?ORG=Hs&CID=586791) | [1](http://www.ncbi.nih.gov/entrez/query.fcgi?tool=bioconductor&cmd=Retrieve&db=PubMed&list_uids=12477932) |  |  |
| [240655_at](https://www.affymetrix.com/LinkServlet?&probeset=240655_at) |  |  |  |  | [BE502785](http://www.ncbi.nlm.nih.gov/entrez/query.fcgi?cmd=search&db=nucleotide&term=BE502785%5BACCN%5D&doptcmdl=GenBank) |  |  |  |  |  |  |
| [241387_at](https://www.affymetrix.com/LinkServlet?&probeset=241387_at) |  |  |  |  | [AW276701](http://www.ncbi.nlm.nih.gov/entrez/query.fcgi?cmd=search&db=nucleotide&term=AW276701%5BACCN%5D&doptcmdl=GenBank) |  |  |  |  |  |  |
| [241484_x_at](https://www.affymetrix.com/LinkServlet?&probeset=241484_x_at) |  |  |  |  | [AI668696](http://www.ncbi.nlm.nih.gov/entrez/query.fcgi?cmd=search&db=nucleotide&term=AI668696%5BACCN%5D&doptcmdl=GenBank) |  |  |  |  |  |  |
| [242245_at](https://www.affymetrix.com/LinkServlet?&probeset=242245_at) |  |  |  |  | [N90719](http://www.ncbi.nlm.nih.gov/entrez/query.fcgi?cmd=search&db=nucleotide&term=N90719%5BACCN%5D&doptcmdl=GenBank) |  |  |  |  |  |  |
| [242463_x_at](https://www.affymetrix.com/LinkServlet?&probeset=242463_x_at) | ZNF600 | zinc finger protein 600 | 19 | -53268749 | [AI620827](http://www.ncbi.nlm.nih.gov/entrez/query.fcgi?cmd=search&db=nucleotide&term=AI620827%5BACCN%5D&doptcmdl=GenBank) | [162966](http://www.ncbi.nlm.nih.gov/sites/entrez?Db=gene&Cmd=DetailsSearch&Term=162966) | [19q13.41](http://www.ncbi.nlm.nih.gov/mapview/map_search.cgi?direct=on&idtype=gene&id=162966) | [Hs.696848](http://www.ncbi.nlm.nih.gov/UniGene/clust.cgi?ORG=Hs&CID=696848) | [3](http://www.ncbi.nih.gov/entrez/query.fcgi?tool=bioconductor&cmd=Retrieve&db=PubMed&list_uids=12576331%2C14702039%2C20936779) | [DNA binding](http://amigo.geneontology.org/cgi-bin/amigo/go.cgi?view=details&query=GO:0003677)  [intracellular](http://amigo.geneontology.org/cgi-bin/amigo/go.cgi?view=details&query=GO:0005622)  [nucleus](http://amigo.geneontology.org/cgi-bin/amigo/go.cgi?view=details&query=GO:0005634)  [zinc ion binding](http://amigo.geneontology.org/cgi-bin/amigo/go.cgi?view=details&query=GO:0008270)  [regulation of transcription](http://amigo.geneontology.org/cgi-bin/amigo/go.cgi?view=details&query=GO:0045449)  [metal ion binding](http://amigo.geneontology.org/cgi-bin/amigo/go.cgi?view=details&query=GO:0046872) |  |
| [243469_at](https://www.affymetrix.com/LinkServlet?&probeset=243469_at) |  |  |  |  | [AI022066](http://www.ncbi.nlm.nih.gov/entrez/query.fcgi?cmd=search&db=nucleotide&term=AI022066%5BACCN%5D&doptcmdl=GenBank) |  |  |  |  |  |  |

209 Genes
